# Post‐Assembly Reactivity of *N*‐Aryl Iminoboronates: Reversible Radical Coupling and Unusual B−N Dynamic Covalent Chemistry

**DOI:** 10.1002/chem.201802790

**Published:** 2018-07-25

**Authors:** Evan N. Keyzer, Alexandru Sava, Tanya K. Ronson, Jonathan R. Nitschke, Anna J. McConnell

**Affiliations:** ^1^ Department of Chemistry University of Cambridge Lensfield Rd Cambridge CB2 1EW UK; ^2^ Otto Diels Institute of Organic Chemistry University of Kiel 24118 Kiel Germany

**Keywords:** dynamic covalent chemistry, iminoboronate, post-assembly, radical coupling, supramolecular chemistry

## Abstract

Post‐assembly reaction of a dynamic covalent iminoboronate system following addition of Cp_2_Co resulted in the formation of a series of new reductively coupled dianionic dimers via C−C bond formation. The dimers formed as a mixture of BN‐containing isomeric products: diastereomers ***rac***_**5**_ and ***meso***_**5,**_ with coupled five‐membered rings, and enantiomeric ***rac***_**6,**_ with a fused six‐membered ring bicyclic system from C−C bond formation and rearrangement of the B−N bonds. Each isomer was identified using ^1^H NMR spectroscopy in combination with single crystal X‐ray structure determination. Interestingly, interconversion between the coupled five‐membered rings (***rac***_**5**_) and fused bicyclic systems (***rac***_**6**_) was found to occur through an unprecedented breaking and reforming of the B−N covalent bond. Further, the coupled products could be converted quantitatively back to their iminoboronate precursors with addition of the electron abstractor Ph_3_C^+^.

## Introduction

**Scheme 1:**
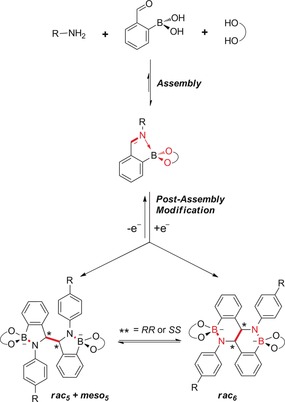
Assembly of iminoboronate esters and reversible post‐assembly reduction to give five‐ and six‐membered isomeric BN‐containing ring systems with two stereogenic centers (*).

Dynamic covalent chemistry (DCC)^1^, ^2^, ^3^ is a powerful tool for the self‐assembly of supramolecular structures, including macrocycles,^4^, ^5^ cryptates,^6^ interlocked structures,^7^, ^8^ and cages.^9^, ^10^, ^11^ Disulfide,^5^, ^12^ imine,^10^, ^13^, ^14^ boronate ester^15^, ^16^ exchange and more recently, orthoester exchange^6^, ^17^ are well‐established reversible covalent reactions in DCC. Iminoboronates are readily assembled using DCC from the condensation of three functionalizable building‐blocks: an amine, a 2‐formylphenyl boronic acid, and a diol (Scheme 1).^18^ They have been exploited in a variety of applications: fluorescence sensing to determine the enantiomeric excess of diols^19^ and amines,^20^, ^21^, ^22^ as intermediates in the bioconjugation of N‐terminal peptides,^23^, ^24^, ^25^ and as building blocks for the self‐assembly of macrocycles and cages.^16^ The ease with which these iminoboronates can assemble and the presence of multiple functional groups makes them an attractive starting platform for the development of more complex BN‐containing materials through post‐assembly modification. Interest in BN‐containing materials^26^ has increased in recent years due to their applications in fields from catalysis^27^ to materials science^28^, ^29^ and biological chemistry.^30^

Radical coupling reactions that result in the formation of carbon‐carbon bonds have been employed in many industrially relevant polymerization reactions (e.g. ATRP)^31^ and small molecule syntheses.^32^ Reductive coupling reactions involving carbonyls and related functional groups, such as imines, have received considerable attention for the enantioselective synthesis of fine chemicals and biologically active compounds, most notably the SmI_2_‐promoted pinacol coupling reaction.^33^, ^34^, ^35^, ^36^, ^37^, ^38^ Dostál and co‐workers recently studied the intermolecular reductive coupling of an iminochloroborane with potassium metal in toluene affording a neutral dimer as a mixture of the expected *rac*‐ (*RR/SS*) and *meso*‐ (*RS*) diastereomers.^39^ In supramolecular chemistry radical reductive coupling reactions have been exploited for the synthesis of macrocyles,^40^, ^41^, ^42^, ^43^ including large macrocyclic hydrocarbons based on spirobifluorenyl units,^44^ and stimuli‐responsive cyclophanes.^45^ Improving the versatility and utility of these radical coupling processes in complex chemical systems, as well as the discovery of new dynamic covalent chemistry, is key to the evolution of chemical synthesis to enable the discovery of new molecular and supramolecular architectures.

Here we report the post‐assembly reactivity of *N*‐aryl iminoboronate systems, wherein reductive C−C bond formation gives rise to new heterocyclic species which exhibit an unprecedented dynamic B−N covalent bond (Scheme 1).^18^ Post‐assembly reduction of a series of *N*‐aryl iminoborane catecholates resulted in two isomeric BN‐containing ring systems containing anionic tetrahedral boron centers and two stereogenic centers; one of these systems formed as a mixture of diastereomeric products (***meso***_**5**_ and ***rac***_**5**_) due to C−C bond formation between two five‐membered rings, whereas the other system formed as a mixture of enantiomers (***rac***_**6**_) based on two fused six‐membered rings as a consequence of C−C bond formation and B−N bond rearrangement. This latter structure represents a unique heterocyclic scaffold that has not been previously reported.

Solution‐state analysis of these structures indicated an unprecedented interconversion between the ***rac***_**5**_ and ***rac***_**6**_ isomers by way of a labile B−N covalent bond at room temperature, establishing a secondary dynamic covalent feature of the overall system. Additionally, the rate of interconversion between the two structures was influenced by manipulating the electronic character of the N‐containing fragment. Furthermore, the mixture of reductively coupled products could be converted quantitatively back to their iminoboronate precursors by the addition of single‐electron abstractor Ph_3_C^+^. Not only do these species display oxidative decoupling reactivity that is not known for analogous systems (pinacol coupling^33^, ^34^, ^35^, ^36^, ^37^, ^38^ etc.), this behaviour demonstrates further reversibility in this iminoboronate‐based system.

## Results and Discussion

**Scheme 2:**
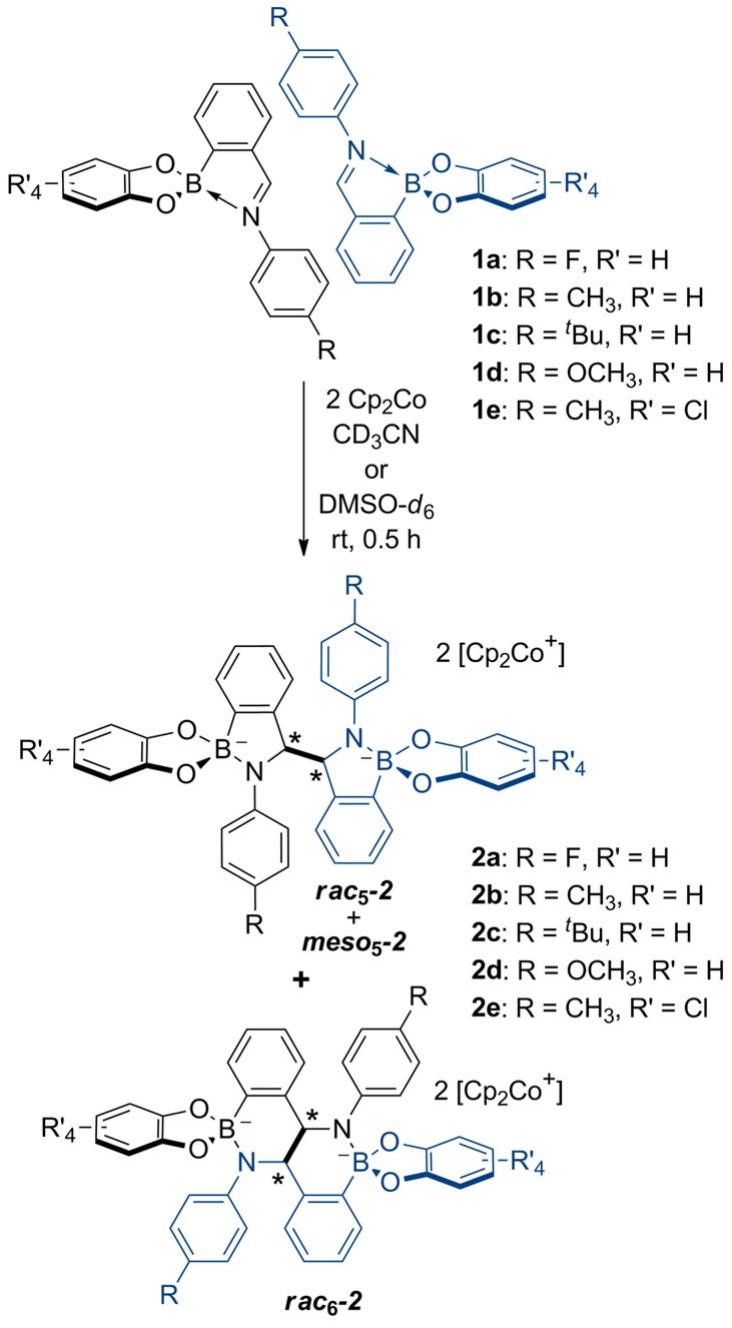
Reductive coupling of iminoboronates **1 a**–**e** in the presence of Cp_2_Co in CH_3_CN at room temperature, generating **2 a**–**e** as a mixture of isomers (* indicates stereogenic centers).

*N*‐Aryl iminoboronates **1 a**–**e** (Scheme 2) were synthesized in yields of greater than 95 % from the reaction of 2‐formylphenyl boronic acid, a 4‐substituted aniline (R=F, CH_3_, *t*Bu, OMe) and a catechol (R′=H, Cl) in CH_3_CN (Supporting Information, Scheme S1). Due to the water and air sensitivity of the reductively coupled products, all manipulations were carried out in a glovebox under a nitrogen atmosphere using dry solvent. Iminoboronate **1 a** was used in the initial investigations of iminoboronate coupling due to its convenient incorporation of an NMR‐active ^19^F nucleus. NMR spectroscopy supported the proposed formation of C−C coupled products from the reaction of iminoboronate **1 a** and one equivalent of Cp_2_Co in dry CD_3_CN (Scheme 2).

The ^1^H NMR spectrum of the reaction mixture after 30 minutes, although complex, is well‐defined (Figure S13); the formation of two isomers of a C−C coupled product **2 a** was indicated by the presence of two signals around *δ*=5.5 ppm corresponding to methine protons, as well as by the loss of the imine signal of the starting material. Two major species were also observed in the ^19^F NMR spectrum of the reaction mixture (Figure S14). These C−C coupled products were subsequently identified as the ***meso***_**5**_ **‐2 a** and ***rac***_**5**_ **‐2 a** isomers (vide infra). Additionally, the ^11^B NMR spectrum of the reaction mixture in CD_3_CN shows broad peaks at ca. *δ*=14 ppm (Figure S15), which is consistent with chemical shifts reported for other tetrahedral N‐coordinated boron complexes.^46^

**Figure 1:**
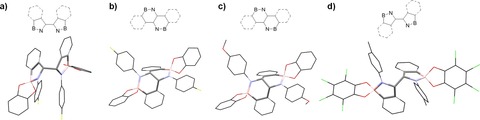
Crystal structures and schematics of atom connectivity of amidoboronates a) ***meso***_**5**_ **‐2 a**, b) ***rac***_**6**_ **‐2 a**, c) ***rac***_**6**_ **‐2 d**, and d) ***rac***_**5**_ **‐2 e**. Cp_2_Co^+^ counter ions, hydrogens, and disorder have been removed for clarity (C, grey; N, blue; O, red; B, pink; Cl, green; F, yellow).

X‐ray diffraction analysis of a deep‐red crystal grown by slow evaporation of the solvent under an inert atmosphere revealed the mesomeric C−C coupled product ***meso***_**5**_ **‐2 a**, containing two five‐membered rings joined at the formerly imine carbon (Figures 1 a, S82). The trigonal geometry around the nitrogen coordinated to a tetrahedral boron and the presence of two Cp_2_Co^+^ molecules per dimer verified the formation of two singly charged anionic boron centers.

Surprisingly, X‐ray diffraction analysis of crystals obtained from a similar but more concentrated reaction did not show either the ***meso***_**5**_ **‐2 a** or ***rac***_**5**_ **‐2 a** isomer. Instead the crystal structure showed a C−C coupled dimer composed of two fused six‐membered BN‐containing rings with racemic stereochemistry (***rac***_**6**_ **‐2 a**, Scheme 2, Figures 1 b, S83). In this fused ring system, the trigonal nitrogen of one coupling partner (shown in blue) interacts with the tetrahedral boron atom of the other coupling partner (shown in black), indicating that B−N bond rearrangement had occurred in addition to C−C bond formation.

**Figure 2:**
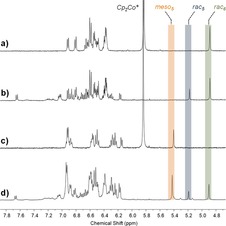
Comparison of ^1^H NMR ([D_6_]DMSO) spectra of a) freshly redissolved ***rac***_**6**_ **‐2 a** crystals, b) a mixture of ***rac***_**6**_ **‐2 a** and ***rac***_**5**_ **‐2 a** isomers 4 days after dissolution of ***rac***_**6**_ **‐2 a** crystals, c) redissolved ***meso***_**5**_ **‐2 a** crystals, and d) the **2 a** mixture containing ***rac***_**6**_, ***rac***_**5**_, and ***meso***_**5**_ isomers.

In order to study whether the ***rac***_**6**_ **‐2 a** isomer exists in solution or is only observed in the solid state, the reaction between iminoboronate **1 a** and one equivalent of Cp_2_Co was carried out in dry [D_6_]DMSO to prevent competing crystallisation (Figure S16). The ^1^H NMR spectrum of the reaction mixture showed the presence of three methine signals at *δ*=4.89, 5.19, and 5.43 ppm (Figure 2 d), as well as three major peaks in the ^19^F spectrum (Figure S17). These observations indicated that three distinct isomers were formed in [D_6_]DMSO, unlike the two isomers observed in solution in CD_3_CN. The signals corresponding to two of these isomers were identified by redissolving isolated crystals of ***meso***_**5**_ **‐2 a** (Figure S19) and ***rac***_**6**_ **‐2 a** (Figure S24) in dry [D_6_]DMSO; the single methine resonances at *δ*=5.43 and 4.89 ppm correspond to the ***meso***_**5**_ **‐2 a** and ***rac***_**6**_ **‐2 a** isomers, respectively (Figure 2 a and 2 c).

Interestingly, the ***rac***_**6**_ **‐2 a** species was observed to convert over a period of 4 days to a product with a methine signal at *δ*=5.18 ppm (Figure 2 b, Figure S30), which was also observed in the original reaction mixture in [D_6_]DMSO. Two signals were also observed in the ^19^F NMR spectrum (Figure S26). This species with the methine signal at *δ*=5.18 ppm was identified as the ***rac***_**5**_ **‐2 a** isomer based on the crystal structure obtained from the analogous reaction between iminoboronate **2 e** and Cp_2_Co (vide infra). Thus, ***meso***_**5**_ **‐2 a**, ***rac***_**5**_ **‐2 a** and ***rac***_**6**_ **‐2 a** were identified as the three coupled products in [D_6_]DMSO and they formed in a ratio of 1:0.27:0.63 based on integration of their ^19^F NMR signals in the equilibrated reaction mixture (Figure S17). Similar chemical shifts were observed for the ***meso***_**5**_ **‐2 a** and ***rac***_**5**_ **‐2 a** isomers in both [D_6_]DMSO and CD_3_CN, allowing the identification of these isomers as the two products from the reaction in CD_3_CN (Figure S18).

**Scheme 3:**
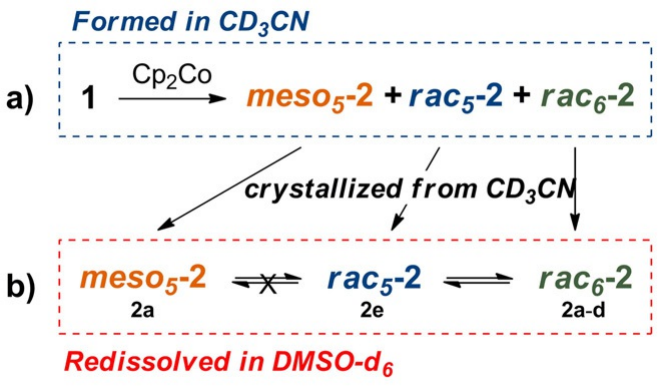
Summary of a) amidoboronate dimer formation, crystal isolation and b) isomerization upon redissolving crystals of **2 a**–**e** in [D_6_]DMSO.

Analogous reactions of iminoboronates **1 b**–**e** with one equivalent of Cp_2_Co in CD_3_CN or [D_6_]DMSO were carried out to investigate the influence of substituents (on the catechol or amine subcomponent) on the number and distribution of products (Scheme 1). As observed in the reductive coupling of iminoboronate **1 a**, the ^1^H NMR spectra of the reaction mixtures contained two and three distinct methine resonances in CD_3_CN and [D_6_]DMSO, respectively and crystals were obtained in all cases from the reactions in CD_3_CN (Scheme 3). Four crystal structures (three polymorphs of the Cp_2_Co^+^ complex and one structure of the Cp*_2_Co^+^ complex) were determined from the reaction of iminoboronate **1 e** with electron‐deficient tetrachlorocatechol. Despite varying the number of equivalents of Cp_2_Co and the reductant (Cp*_2_Co instead of Cp_2_Co) in the reaction, the crystal structures all displayed a racemic mixture of the dimer ***rac***_**5**_ **‐2 e**, composed of coupled five‐membered NB‐containing rings (Figure 1 d, Figures S87–S90).

In contrast, the solid‐state structures from the reactions of iminoboronates **1 b**–**d**, differing in the substitution of the amine subcomponent, were found to be the ***rac***_**6**_ **‐2** isomer (for example ***rac***_**6**_ **‐2 d**, Figures 1 c, S84–S86). In all cases, the redissolved crystals were observed to equilibrate with a second species over time in [D_6_]DMSO (Scheme 3, Figures S96‐S100). The redissolved crystals of ***rac***_**5**_ **‐2 e** also equilibrated with a second species (Figure S101). A comparison of the redissolved crystals from all the reactions (***rac***_**6**_ **‐2 a**–**d**, ***rac***_**5**_ **‐2 e**) revealed that the methine chemical shift of the two species did not vary significantly with substitution of the catechol and amine subcomponents (Figure S102), allowing identification of ***rac***_**5**_ **‐2 a**–**e** and ***rac***_**6**_ **‐2 a**–**e** as the species with methine signals around 5.2 and 4.9 ppm, respectively.

While ***meso***_***5***_ **‐2 a** was the only ***meso*** isomer observed to crystallize from the reaction mixture (Scheme 3), the ^1^H NMR spectrum of ***meso***_***5***_ **‐2 c** could be inferred from the reaction mixture in CD_3_CN following crystallization of ***rac***_**6**_ **‐2 c** (Figure S46). ***Meso***_***5***_ **‐2 b** and ***meso***_***5***_ **‐2 d** were characterized in CD_3_CN as a mixture with ***rac***_**5**_ **‐2** (Figures S31, S56). These spectra were similar to that of the redissolved ***meso***_***5***_ **‐2 a** crystals in [D_6_]DMSO with a methine signal around 5.5 ppm (Figure 2 c).

Having related the solid‐state structures to solution‐state NMR spectra, each isomer could be identified based on the chemical shift range where its methine resonance appears in both CD_3_CN and [D_6_]DMSO; the most downfield resonance around 5.5 ppm is attributed to the ***meso***_**5**_ isomer, the middle methine resonance around 5.2 ppm to the ***rac***_**5**_ isomer, and the most upfield resonance around 4.9 ppm to the ***rac***_**6**_ isomer (Figure 2). All three isomers were observed in the reactions in [D_6_]DMSO, while only the ***rac***_**5**_ and ***meso***_**5**_ isomers were observed as the major species in solution in CD_3_CN due to competing crystallization.

Intrigued by this unprecedented interconversion between the coupled five‐membered ring (***rac***_**5**_ **‐2**) and fused six‐membered ring (***rac***_***6***_ **‐2**) systems (Scheme 3) by way of a labile B−N covalent bond, we probed the formation of ***rac***_***6***_ **‐2** through time‐course ^1^H NMR studies. Although significant quantities of the ***rac***_**6**_ **‐2** isomer were not observed in the reactions carried out in CD_3_CN by ^1^H NMR spectroscopy, the intensity of the ***rac***_**5**_ **‐2** peaks diminished as it equilibrated with the precipitating ***rac***_**6**_ **‐2** species (Figure S91). The ***rac***_**6**_ **‐2** product, however, was observed if the reaction was conducted in [D_6_]DMSO as a thermodynamic product, converting from the kinetically formed ***rac***_**5**_ **‐2** species (Figures S92, S93). That no conversion of ***meso***_**5**_ **‐2** to the ***meso***_**6**_ **‐2** was observed, even at 90 °C (Figure S94), suggested that the formation of the *meso* fused six‐membered ring system is strongly disfavored. We postulate that the conversion between ***rac***_**5**_ **‐2** and ***rac***_**6**_ **‐2** involves the transit of a nitrogen anion from one boron to the opposite boron, facilitated by the polar aprotic solvent (Scheme S2).

We also investigated the influence of electronic effects on the rate of interconversion between the ***rac***_**5**_ **‐2** and ***rac***_**6**_ **‐2** isomers by redissolving isolated crystals of one isomer. Time‐course ^1^H NMR data qualitatively showed that the rate of conversion depended on the electron‐donating ability of the substituent on the aniline; ***rac***_**6**_ **‐2 d** with anisidine substituents converted most rapidly. Consequently, both ***rac***_**6**_ **‐2 d** and ***rac***_**5**_ **‐2 d** were observed in the initial NMR spectrum (Figures S99, S100). In contrast, ***rac***_**6**_ **‐2 a**–**c** predominated in the spectra immediately after dissolution of the crystals and converted more slowly than ***rac***_**6**_ **‐2 d**, which we attribute to their less electron‐rich substituents (Figures S96–98). This electron‐donor dependence is likely due to the build‐up of negative charge around nitrogen and boron, favoring bond cleavage (Scheme S2).

In contrast to the conversion between *rac* and *meso* species analogous to the case observed by Dostál and co‐workers,^39^ ***rac***_**5/6**_ **‐2 a** was not observed by ^1^H NMR spectroscopy to form from the isolated ***meso***_**5**_ **‐2 a** crystals in [D_6_]DMSO (Scheme 3 b, Figure S94). Furthermore, conversion of ***rac***_**5/6**_ **‐2** to ***meso***_**5**_ **‐2** was not observed during equilibration of redissolved crystals at room temperature (Figures S96–S101) or upon heating to 130 °C (Figure S95). The transition from a *meso* coupled species to one with *rac* stereocenters requires the cleavage of the C−C bond, a process not accessible in this system under the tested conditions (130 °C in DMSO).

**Scheme 4:**
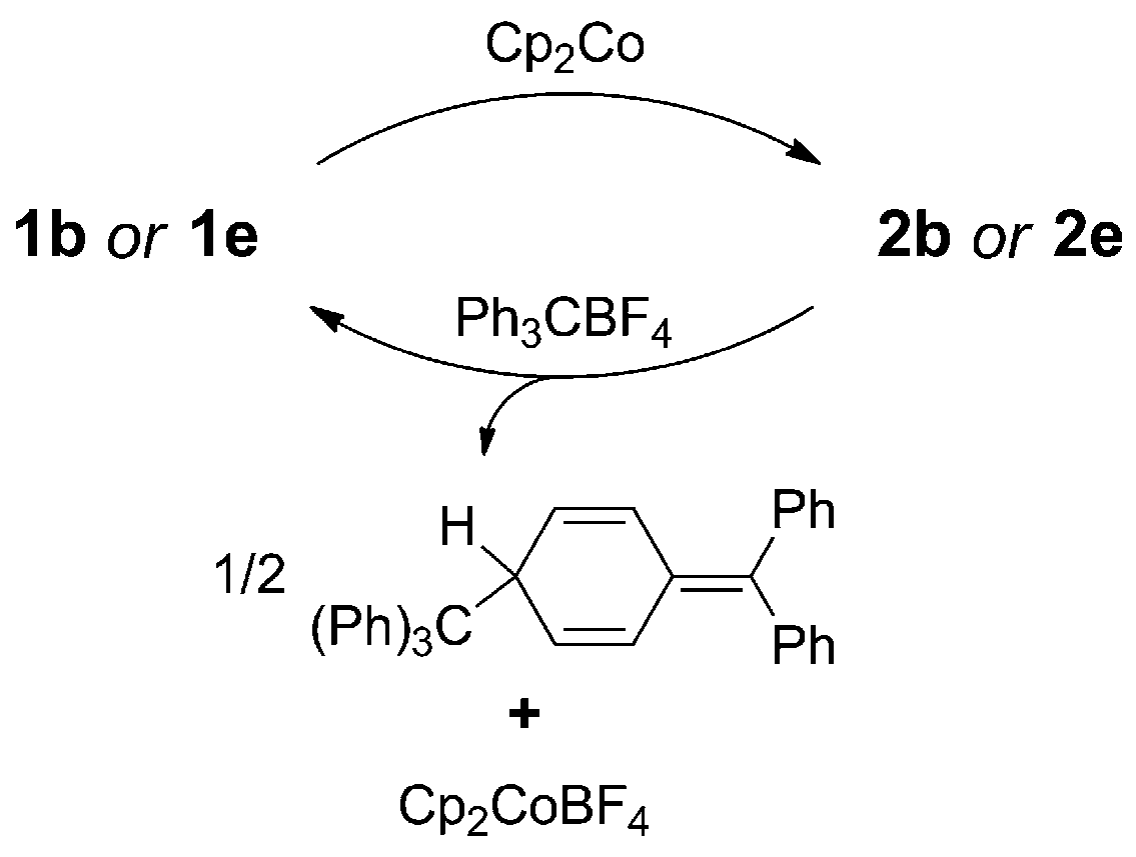
Reductive coupling of iminoboronates **1 b** and **1 e** and subsequent tritylium‐induced oxidative decoupling of dimers **2 b** and **2 e**.

While no interconversion between ***meso***_**5**_ **‐2** and ***rac***_**5**_ **‐2** species‐proceeding through homolytic C−C cleavage was observed by NMR spectroscopy, the mechanism of C−C bond coupling was probed using the tritylium cation (as Ph_3_CBF_4_) and the 2,2,6,6‐tetramethylpiperidine‐*N*‐oxyl radical (TEMPO^.^) to intercept radicals formed from the coupled dimers. The reaction of both the **2 b** and **2 e** reaction mixtures with tritylium tetrafluoroborate (Ph_3_CBF_4_, 1.1 equiv) resulted in quantitative conversion of the dimers to iminoboronates **1 b** and **1 e**, respectively, within 10 minutes (Scheme 4). ^1^H NMR analysis of these mixtures in CD_3_CN was in excellent agreement with the spectra of **1 b** and **1 e** and exhibited signals attributed to the trityl dimer, formed via radical coupling of the tritylium cation,^47^ as well as Cp_2_CoBF_4_ (Figures S103, S104). Through the addition of an electron abstractor the coupled products selectively and quantitatively reverted to the iminoboronate starting material, reactivity that is not known in related reductively coupled aldehyde or imine systems.^33^, ^34^, ^35^, ^36^, ^37^, ^38^ Using Ph_3_CBF_4_ for multiple cycles of coupling and decoupling was not possible due to the reaction of the trityl dimer with the iminoboronates upon subsequent additions of Cp_2_Co.

**Figure 3:**
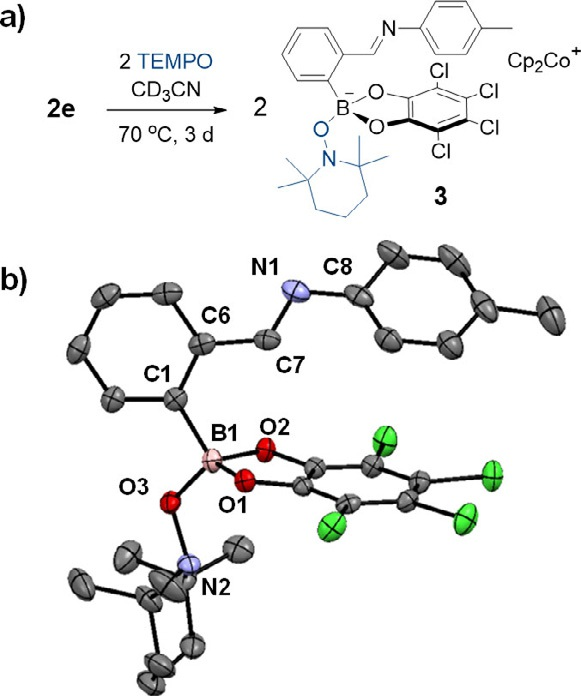
a) Formation of **3** via addition of TEMPO to **2 e**. b) Crystal structure of **3**. Thermal ellipsoids are displayed at 50 % probability; Cp_2_Co^+^ counter ion and hydrogens have been removed for clarity (C, grey; N, blue; O, red; B, pink; Cl, green).

The stable 2,2,6,6‐tetramethylpiperidine‐*N*‐oxyl radical (TEMPO^.^) was employed to further investigate the apparent lability of the coupled C−C bond. X‐ray analysis of yellow crystals retrieved from the reaction of **2 e** with two equivalents of TEMPO^.^ in CD_3_CN at 70 °C for three days revealed the formation of the anionic imine **3** with TEMPO, rather than nitrogen, bound to boron (Figures 3, S105, S106). The **2 e** mixture was chosen to study the reaction with TEMPO as crystallization of the reductively coupled product from the reaction mixture was observed to be the slowest for this reaction. This would ensure the reaction of the reductively coupled products with TEMPO prior to competing crystallization.

The TEMPO radical is known to react with alkylcatecholboranes, affording the trigonal TEMPO‐catecholboronate along with one equivalent of an alkyl radical via a S_H_2 mechanism, proceeding through a tetrahedral intermediate.^48^, ^49^, ^50^ However, the tetrahedral anionic boron of **2 e** as well as significant steric crowding around boron in the area opposite to nitrogen, make ejection of a nitrogen‐based radical from boron an unlikely mechanism. Accordingly, we hypothesize that the TEMPO radical abstracted an electron from the more accessible nitrogen lone pair, resulting in the nitrogen‐based radical cation and the TEMPO anion. Following this, the dimer could cleave homolytically and allow TEMPO^−^ to attack boron as a nucleophile.^51^

## Conclusion

In summary, the addition of a single‐electron reductant (Cp_2_Co) to a dynamic covalent iminoboronate system resulted in the generation of amidoboronate dimers which express new dynamic and stimuli‐responsive behavior. A combination of solid‐state X‐ray analysis and solution‐state NMR measurements showed that this reaction afforded a mixture of coupled five‐membered and fused six‐membered ring systems, ***rac***_**5**_ **‐2**, ***meso***_**5**_ **‐2** and ***rac***_**6**_ **‐2**, which were identified by their respective methine ^1^H resonances. Notably, the BN‐containing fused ring structure of ***rac***_**6**_ **‐2** represents a molecular scaffold that is unique to this coupled system. ^1^H NMR studies conducted on isolated crystals of the type ***rac***_**6**_ **‐2** as well as ***rac***_**5**_ **‐2** revealed that these two species are able to interconvert, with retention of stereochemistry, by way of a dynamic B−N bond. The dynamic behavior of these amidoboronates is unique among Lewis pairs, exhibiting B−N covalent bond lability at low temperatures, possibly providing access to new aspects of reactivity exhibited by Lewis pairs.^52^ Furthermore, these dimers were observed to quantitatively decouple back to their iminoboronate precursors in response to the addition of an electron abstractor (Ph_3_C^+^) — reactivity not observed thus far in related aldehyde and imine reductive coupling products. We are exploring the use of the dynamic covalent nature of this reductively coupled system for the self‐assembly of larger supramolecular architectures and functional materials.

## Experimental Section

For a detailed description of the experimental procedures, compound characterization, and X‐ray data and refinement, see the Supporting Information. https://summary.ccdc.cam.ac.uk/structure-summary?doi=10.1002/chem.201802790 1844532–1844541, contain the supplementary crystallographic data for this paper. These data are provided free of charge by http://www.ccdc.cam.ac.uk/

## Conflict of interest

The authors declare no conflict of interest.

## Supporting information

As a service to our authors and readers, this journal provides supporting information supplied by the authors. Such materials are peer reviewed and may be re‐organized for online delivery, but are not copy‐edited or typeset. Technical support issues arising from supporting information (other than missing files) should be addressed to the authors.

Supplementary
