# Supplementary material for: Post‐Assembly Reactivity of N‐Aryl Iminoboronates: Reversible Radical Coupling and Unusual B−N Dynamic Covalent Chemistry
Source: Chemistry. 2018 Jul 25;24(46):12000–5. doi: 10.1002/chem.201802790 (PMC6175077; doi:10.1002/chem.201802790)
Supplement: Supplementary file 1 — Supplementary [file CHEM-24-12000-s001.pdf]

CHEMISTRY
A European Journal

Supporting Information

Post-Assembly Reactivity of *N*-Aryl Iminoboronates: Reversible Radical Coupling and Unusual B—N Dynamic Covalent Chemistry

Evan N. Keyzer,[a] Alexandru Sava,[a] Tanya K. Ronson,[a] Jonathan R. Nitschke,[a] and Anna J. McConnell*[a, b]

chem_201802790_sm_miscellaneous_information.pdf

Table of Contents

1 Materials and Methods 3
1.1 NMR Spectroscopy 3
1.2 Mass Spectrometry 3
1.3 X-Ray Crystallography 3
2 Synthesis and Characterization of Iminoboronates 1a-e 4
2.1 *N*-(4-Fluorophenyl)iminoboronate pyrocatechol ester (1a) 4
2.2 *N*-Tolyliminoboronate pyrocatechol ester (1b) 7
2.3 *N*-(4-*tert*-Butylphenyl)iminoboronate pyrocatechol ester (1c) 8
2.4 *N*-(4-Methoxyphenyl)iminoboronate pyrocatechol ester (1d) 10
2.5 *N*-Tolyliminoboronate tetrachlorocatechol ester (1e) 11
3 Solution Characterisation of the Fluoroaniline-Pyrocatechol Reductively Coupled Dimer (2a) 13
3.1 *rac*$_5$-2a and *meso*$_5$-2a Mixture in CD_3CN 13
3.2 *rac*$_5$-2a, r*ac*$_6$-2a and *meso*$_5$-2a Mixture in DMSO-d_6 14
3.3 *meso*$_5$-2a 16
3.4 *rac*$_6$-2a 20
3.5 *rac*$_5$-2a 23
4 Solution Characterisation of the Toluidine-Pyrocatechol Reductively Coupled Dimer (2b).. 24
4.1 *rac*$_5$-2b and *meso*$_5$-2b Mixture in CD_3CN 24
4.2 *rac*$_5$-2b, r*ac*$_6$-2b and *meso*$_5$-2b Mixture in DMSO-d_6 29
4.3 *rac*$_6$-2b 30
4.4 *rac*$_5$-2b 33
5 Solution Characterisation of the *tert*-Butylaniline-Pyrocatechol Reductively Coupled Dimer (2c) 33
5.1 *rac*$_5$-2c and *meso*$_5$-2c Mixture in CD_3CN 33
5.2 *meso*$_5$-2c 34
5.3 *rac*$_6$-2c 37
5.4 *rac*$_5$-2c 40
6 Solution Characterisation of the Methoxyaniline-Pyrocatechol Reductively Coupled Dimer (2d) 41
6.1 *rac*$_5$-2d and *meso*$_5$-2d Mixture in CD_3CN 41
6.2 *rac*$_5$-2d, r*ac*$_6$-2d and *meso*$_5$-2d Mixture in DMSO-d_6 45
6.3 *rac*$_6$-2d and *rac*$_5$-2d 46
7 Solution Characterisation of the Toluidine-Tetrachlorocatechol Reductively Coupled Dimer (2e) 50
7.1 *rac*$_5$-2e and *meso*$_5$-2e Mixture in CD_3CN 50
7.2 *rac*$_5$-2e, r*ac*$_6$-2e and *meso*$_5$-2e Mixture in DMSO-d_6 53
7.3 *rac*$_5$-2e 54

7.4 *rac*$_6$-2e 57
8 X-Ray Crystal Structures of Reductively Coupled Products 58
8.1 *meso*$_5$-2a 58
8.2 *rac*$_6$-2a 59
8.3 *rac*$_6$-2b 60
8.4 *rac*$_6$-2c 61
8.5 *rac*$_6$-2d 62
8.6 *rac*$_5$-2e 63
9 Time-Course NMR Studies 67
9.1 Reductive Coupling of 1c in CD_3CN 67
9.2 Reductive Couplings in DMSO-d_6 68
9.3 NMR Studies of Interconversion between the *meso*$_5$-2 and *rac*$_{5/6}$ Isomers 69
10 Time-Course NMR Studies for *Rac*$_5$/*Rac*$_6$ Equilibration 70
10.1 2a 70
10.2 2b 71
10.3 2c 71
10.4 2d 71
10.5 2e 73
10.6 Comparison of Chemical Shifts 74
11 Reversible Radical Coupling with Ph_3CBF_4 74
11.1 2b 75
11.2 2e 75
12 Reaction of 2e with TEMPO 76
12.1 X-Ray Structure of 3 77
13 References 78

1 Materials and Methods

Reagents and solvents were purchased from commercial suppliers and used without further purification, unless otherwise specified. 2-formylphenylboronic acid was purchased from Arcos. Pyrocatechol, toluidine, tetrachlorocatechol monohydrate, bis(cyclopentadienyl) cobalt, bis(pentamethylcyclopentadienyl) cobalt, tritylium tetrafluoroborate, 2,2,6,6-tetramethylpiperidinyloxy were purchased from Sigma Aldrich. The solvents were purchased from Sigma Aldrich; prior to use, deuterated acetonitrile was distilled over calcium hydride and deuterated DMSO was dried over calcium hydride, filtered and stored over molecular sieves.

Due to the water and air sensitivity of the reductively coupled products, all manipulations were carried out in a glovebox under a nitrogen atmosphere using dry solvent.

1.1 NMR Spectroscopy

NMR spectra were recorded on a Bruker Avance DRX-400, Bruker Avance 500 BB ATM, Bruker DRX-500, or Bruker Avance 500 Cryo spectrometers. Chemical shifts for ^{1}H, ^{13}C, ^{19}F and ^{11}B spectra are expressed in parts per million (ppm) and coupling constants (*J*) are reported in Hertz (Hz). ^{1}H and ^{13}C were referenced to the solvent residual peak and ^{11}B was referenced to $BF_3{\cdot}Et_2O$ at 0.0 ppm. All measurements were carried out at 298 K unless reported otherwise. The following abbreviations are used to describe signal multiplicity for ^{1}H, ^{13}C and ^{11}B NMR spectra: s: singlet, d: doublet, t: triplet, m: multiplet, b: broad.

Each isomer of the reductively coupled products was fully characterised in solution, where possible, using ^{1}H NMR spectroscopy. Mixtures of two isomers were characterised in cases where one of the products could not be obtained as a single isomer by crystallisation (*e.g.* ***rac***$_5$**-2a-d**, ***rac*6-2e**, ***meso***$_5$**-2b**) or due to fast interconversion between isomers (*e.g.* ***rac***$_6$**-2d** and ***rac***$_5$**-2d**). In some instances, unambiguous assignment of all signals in these mixtures was not possible due to the number of overlapping signals. The broadness of the ^{13}C NMR signal for the carbon directly attached to the boron atom (C_a) often precluded assignment of this signal.

1.2 Mass Spectrometry

The mass spectra of the iminoboronates were acquired on a Jeol AccuTOF mass spectrometer. It was not possible to obtain mass spectra of the reductively coupled dimers in all cases due to their air and water sensitivity as well as fragmentation under mass spectrometry conditions. Where reported, nanospray ionisation (NSI) mass spectra provided by the EPSRC National MS Service Centre at Swansea were acquired on a Thermofisher LTQ Orbitrap XL.

1.3 X-Ray Crystallography

Data were with collected using a Bruker D8 VENTURE equipped with high-brilliance IμS Cu-Kα radiation (1.54178 Å), with ω and *ψ* scans at 180(2) K or at Beamline I19 of Diamond Light Source[1] employing silicon double crystal monochromated synchrotron radiation (0.6889 Å) with ω scans at 100(2) K. Data integration and reduction were undertaken with SAINT and XPREP.[2] Subsequent computations were carried out using the WinGX-32 graphical user interface.[3] Multi-scan empirical absorption corrections were applied to the data using SADABS.[2] Structures were solved by direct methods using SHELXT[4] or charge-flipping using SUPERFLIP[5] then refined and extended with SHELXL.[6] In general, non-hydrogen atoms with occupancies greater than 0.5 were refined anisotropically. Some disordered solvent molecules were refined with isotropic thermal parameters. Carbon-bound hydrogen atoms were included in idealised positions and refined using a riding model. Disorder was modelled using standard crystallographic methods including constraints, restraints and rigid bodies where necessary. Crystallographic data have been deposited with the CCDC (1844532-1844541).

2 Synthesis and Characterization of Iminoboronates 1a-e

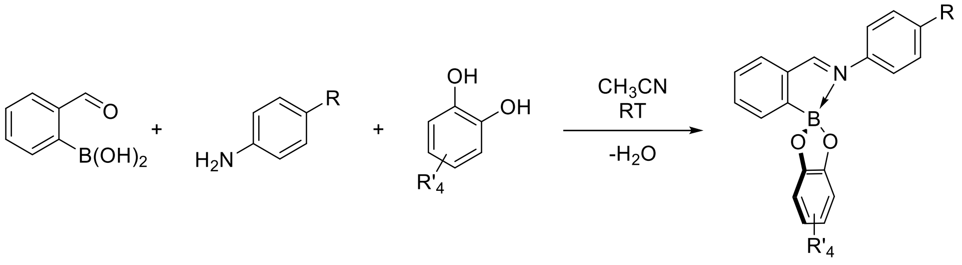

1a: R = F, R' = H
1b: R = CH_3, R' = H
1c: R = ^{t}Bu, R' = H
1d: R = OCH_3, R' = H
1e: R = CH_3, R' = Cl

Scheme S1. Formation of *N*-aryl iminoborane catecholates **1a-e**.

2.1 *N*-(4-Fluorophenyl)iminoboronate pyrocatechol ester (1a)

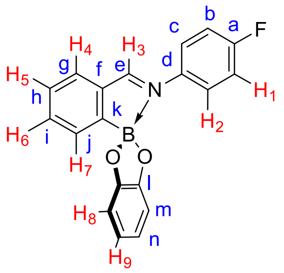

4-Fluoroaniline (15 µL, 0.158 mmol) 2-formylphenylboronic acid (23.75 mg, 0.158 mmol) and pyrocatechol (17.48 mg, 0.158 mmol) were dissolved in CH_3CN (2 mL) and stirred at room temperature for 2.5 hours. The solvent was removed *in vacuo* to afford the desired product as an orange solid (50.3 mg, >95%).

^{1}H NMR (400 MHz, 298.0 K, $CDCl_3$): δ 8.51 (1H, s, H_3), 7.70 (1H, d, ^3J = 7.4 Hz, H_7), 7.67 (1H, d, ^3J = 7.4 Hz, H_4), 7.61 (1H, td, ^3J = 7.4 Hz, ^4J = 0.9 Hz, H_6), 7.43 (1H, td, ^3J = 7.4 Hz, ^4J = 1.1 Hz, H_5), 7.37-7.32 (2H, m, H_2), 7.01-6.95 (2H, m, H_1), 7.01-6.95 (2H, m, $H_{8/9}$), 6.86-6.81 (2H, m, $H_{8/9}$)

^{13}C NMR (126 MHz, 298.0 K, $CDCl_3$): δ 166.7 (C_e), 162.7 (d, $^1J_{CF}$ = 250 Hz, C_a), 151.8 (C_l), 137.2 (d, $^4J_{CF}$ = 2 Hz, C_d), 137.0 (C_f), 135.0 (C_i), 131.3 (C_j), 129.1 (C_h), 127.6 (C_g), 123.9 (d, $^3J_{CF}$ = 9 Hz, C_c), 119.6 ($C_{m/n}$), 116.6 (d, $^2J_{CF}$ = 23 Hz, C_b), 110.2 ($C_{m/n}$)

^{19}F NMR (376 MHz, 298.0 K, $CDCl_3$): δ -111.17

^{11}B NMR (128 MHz, 298.0 K, CD_3CN): δ 15.62 (bs)

HRMS-EI: *m/z* calcd for $C_{19}H_{13}{}^{11}B^{19}FNO_2$ $[M]^+$ 317.10234, found $[M]^+$ 317.10187 (-1.47 ppm)

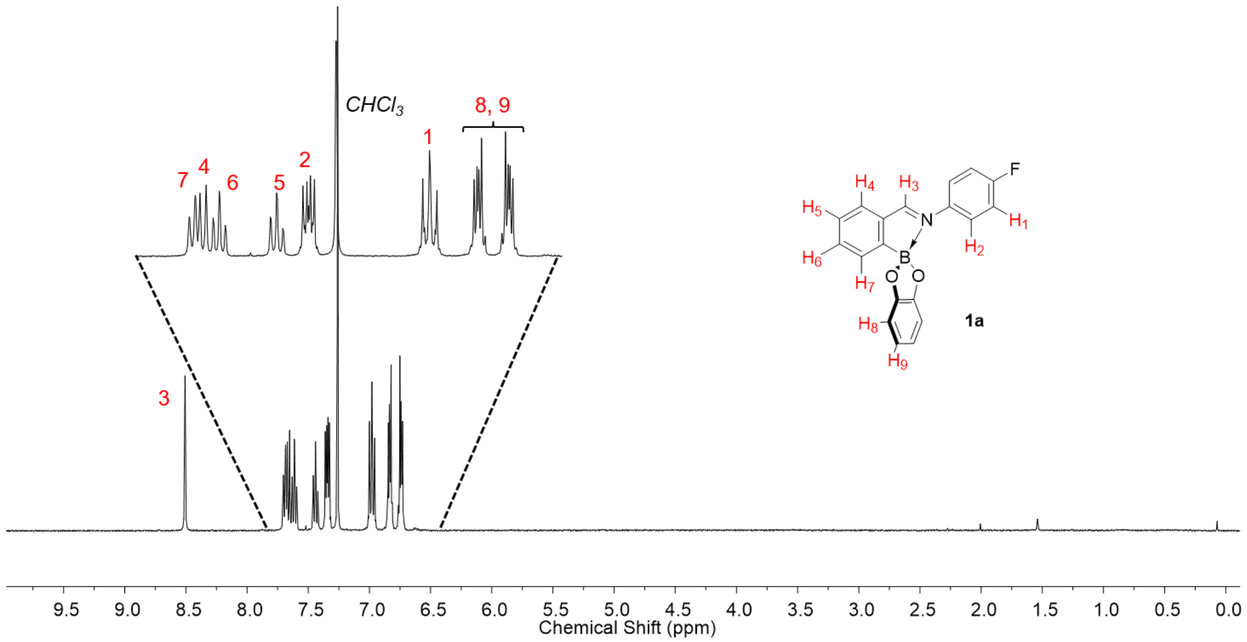

Figure S1. ^{1}H NMR spectrum (400 MHz, $CDCl_3$) of **1a**.

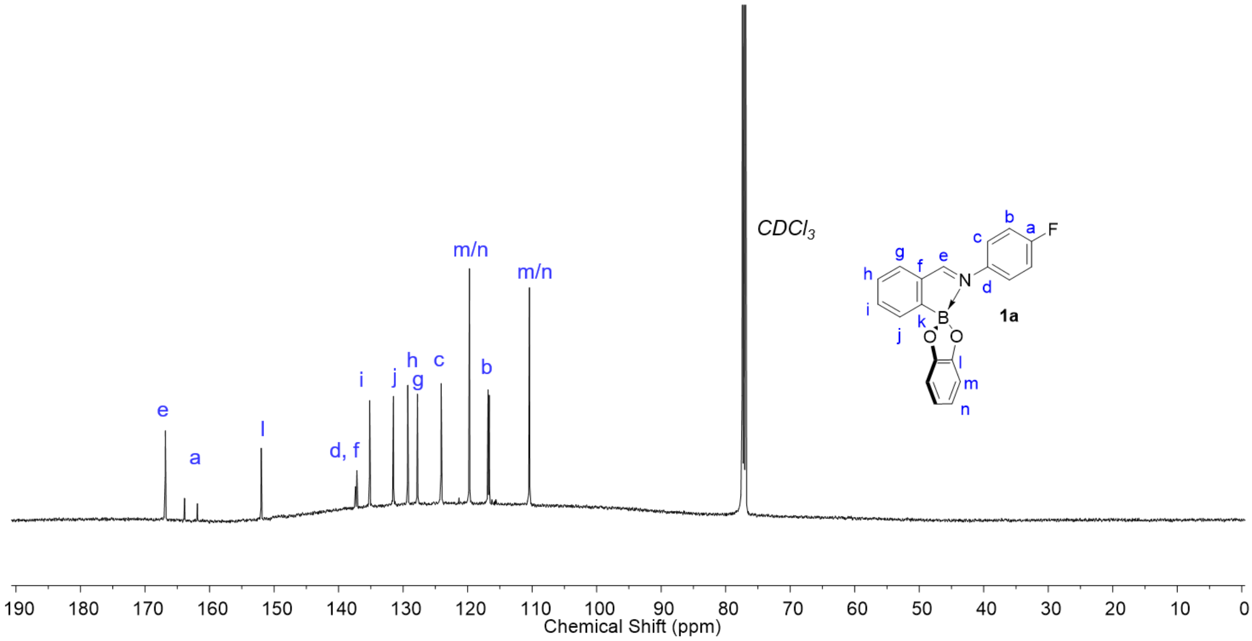

Figure S2. ^{13}C NMR spectrum (126 MHz, $CDCl_3$) of **1a**.

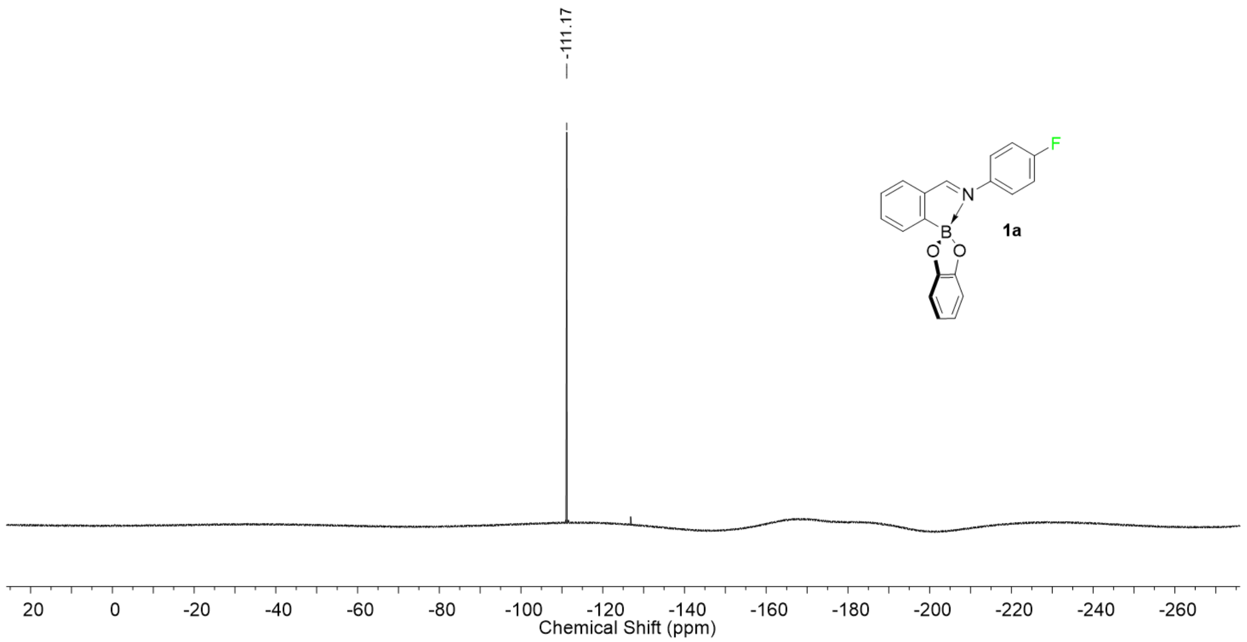

Figure S3. ^{19}F NMR spectrum (376 MHz, $CDCl_3$) of **1a**.

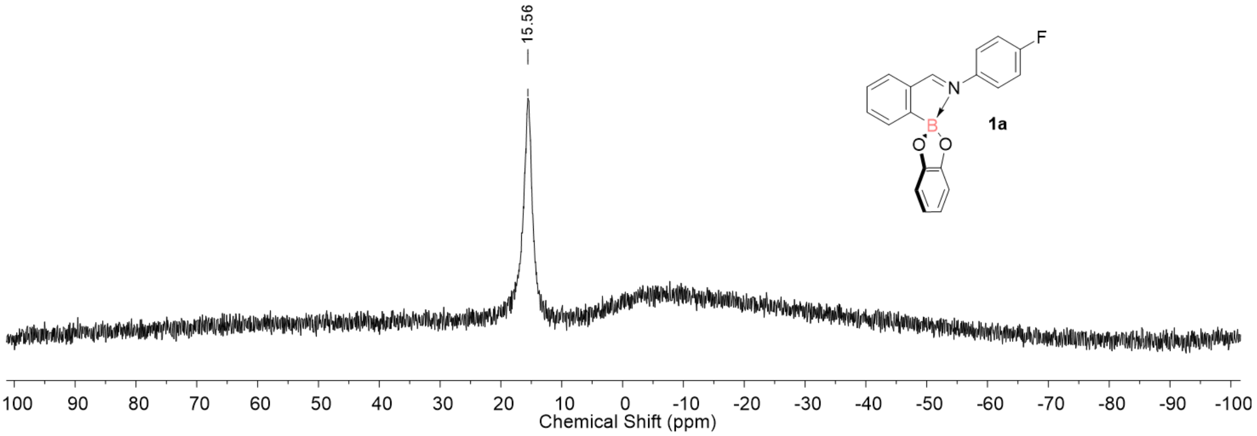

Figure S4. ^{11}B NMR spectrum (128 MHz, CD_3CN) of **1a**.

2.2 *N*-Tolyliminoboronate pyrocatechol ester (1b)

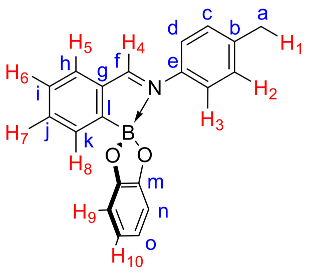

2-Formylphenylboronic acid (10.00 mg, 0.0667 mmol), toluidine (7.15 mg, 0.0667 mmol), and pyrocatechol (7.34 mg, 0.0667 mmol) were dissolved in CH_3CN (2 mL) and stirred at room temperature for 2 hours. The solvent was removed *in vacuo* to afford the desired product as an orange solid (20.5 mg, >95%).

^{1}H NMR (400 MHz, 298.0 K, $CDCl_3$): δ 8.52 (1H, s, *H4*), 7.68 (1H, d, ^3J = 7.3 Hz, *H8*), 7.63 (1H, d, ^3J = 7.3 Hz, *H5*), 7.59 (1H, t, ^3J = 7.3 Hz, *H7*), 7.42 (1H, t, ^3J = 7.3 Hz, *H6*), 7.24 (2H, d, ^3J = 8.4 Hz, *H3*), 7.08 (2H, d, ^3J = 8.4 Hz, *H2*), 6.86-6.81 (2H, m, *H9/10*), 6.77-6.71 (2H, m, *H9/10*), 2.29 (3H, s, *H1*)

^{13}C NMR (101 MHz, 298.0 K, $CDCl_3$): δ 166.2 (C_f), 152.2 (C_m), 139.6 (C_b), 138.6 (C_e), 137.3 (C_g), 134.8 (C_j), 131.3 (C_k), 130.3 (C_c), 129.1 (C_i), 127.5 (C_h), 121.9 (C_d), 119.5 ($C_{n/o}$), 110.3 ($C_{n/o}$), 21.2 (C_a)

^{11}B NMR (128 MHz, 298.0 K, CD_3CN): δ 15.05 (bs)

HRMS-EI: *m/z* calcd for $C_{20}H_{16}{}^{11}BNO_2$ $[M]^+$ 313.12741, found $[M]^+$ 313.12721 (-0.62 ppm)

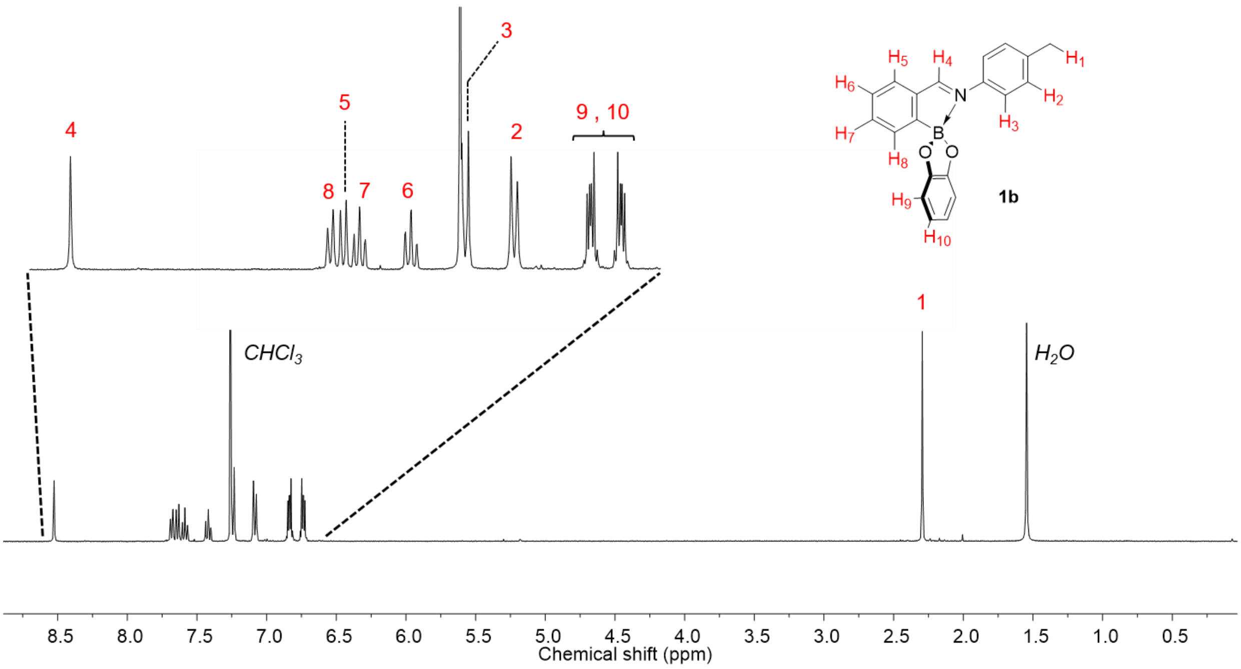

Figure S5. ^{1}H NMR spectrum (400 MHz, $CDCl_3$) of **1b**.

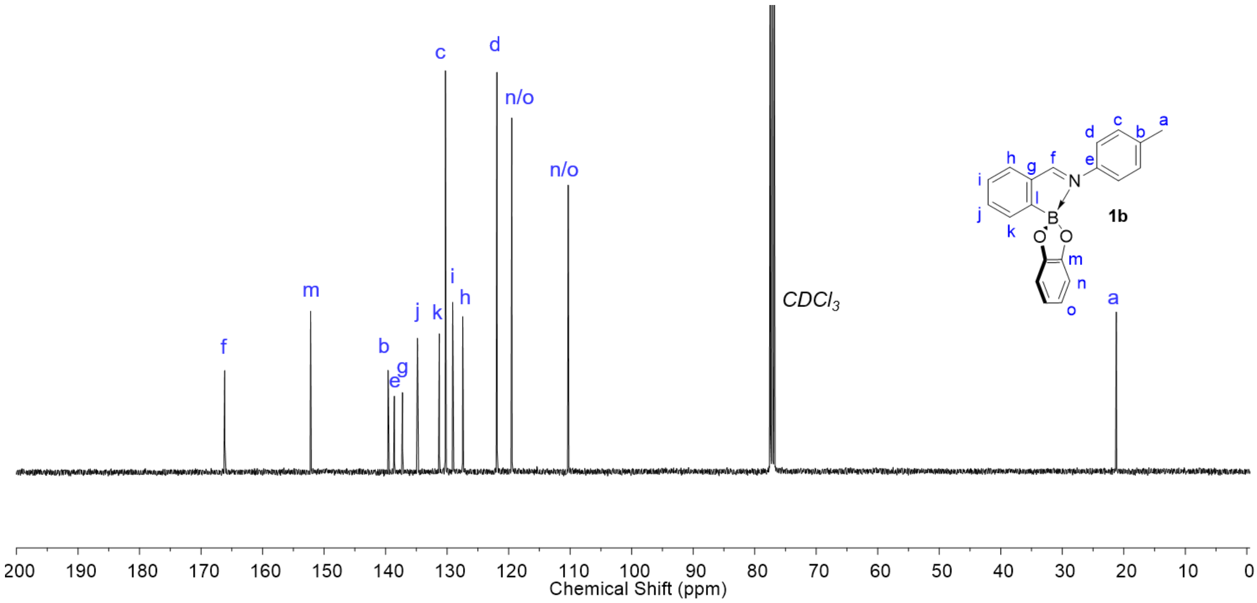

Figure S6. ^{13}C NMR spectrum (101 MHz, $CDCl_3$) of **1b**.

2.3 *N*-(4-*tert*-Butylphenyl)iminoboronate pyrocatechol ester (1c)

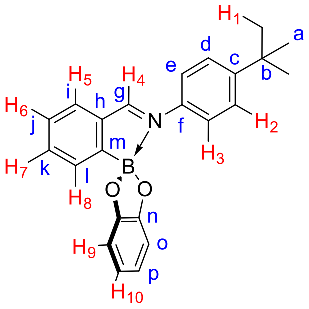

4-*tert*-Butylaniline (20 µL, 0.125 mmol) 2-formylphenylboronic acid (18.78 mg, 0.125 mmol) and pyrocatechol (13.81 mg, 0.125 mmol) were dissolved in CH_3CN (2 mL) and stirred at room temperature for 2 hours, forming a yellow precipitate. The resulting solid and brown solution was left still overnight and the solvent evaporated under nitrogen flow. The product was collected as a yellow solid and dried *in vacuo* (48.1 mg, >95%). Despite numerous attempts to dry the solid sample, residual CH_3CN could not be removed.

^{1}H NMR (500 MHz, 298.0 K, $CDCl_3$): δ 8.58 (1H, s, H_4), 7.67 (1H, dd, ^3J = 7.5 Hz, ^4J = 1.1 Hz, H_8), 7.64 (1H, dt, ^3J = 7.5 Hz, ^4J = 1.0 Hz, H_5), 7.58 (1H, td, ^3J = 7.5 Hz, ^4J = 1.0 Hz, H_7), 7.42 (1H, td, ^3J = 7.5 Hz, ^4J = 1.1 Hz, H_6), 7.32-7.27 (4H, m, $H_{2/3}$), 6.87-6.84 (2H, m, $H_{9/10}$), 6.77-6.74 (2H, m, $H_{9/10}$), 1.26 (9H, s, H_1)

^{13}C NMR (126 MHz, 298.0 K, $CDCl_3$): δ 166.0 (C_g), 152.7 (C_c), 152.1 (C_n), 149.6 (b, C_m), 138.2 (C_f), 137.0 (C_h), 134.8 (C_k), 131.1 (C_l), 129.0 (C_j), 127.3 (C_i), 126.6 (C_d), 121.5 (C_e), 119.3 ($C_{o/p}$), 110.3 ($C_{o/p}$), 34.7 (C_b), 31.1(C_a)

^{11}B NMR (128 MHz, 298.0 K, CD_3CN): δ 15.55 (bs)

EI: *m/z* 340.15 $[M-CH_3]^{\cdot+}$, 355.17 $[M]^+$

HRMS-EI: *m/z* calcd for $C_{23}H_{22}{}^{11}BNO_2$ $[M]^+$ 355.17436, found $[M]^+$ 355.17433 (-0.09 ppm)

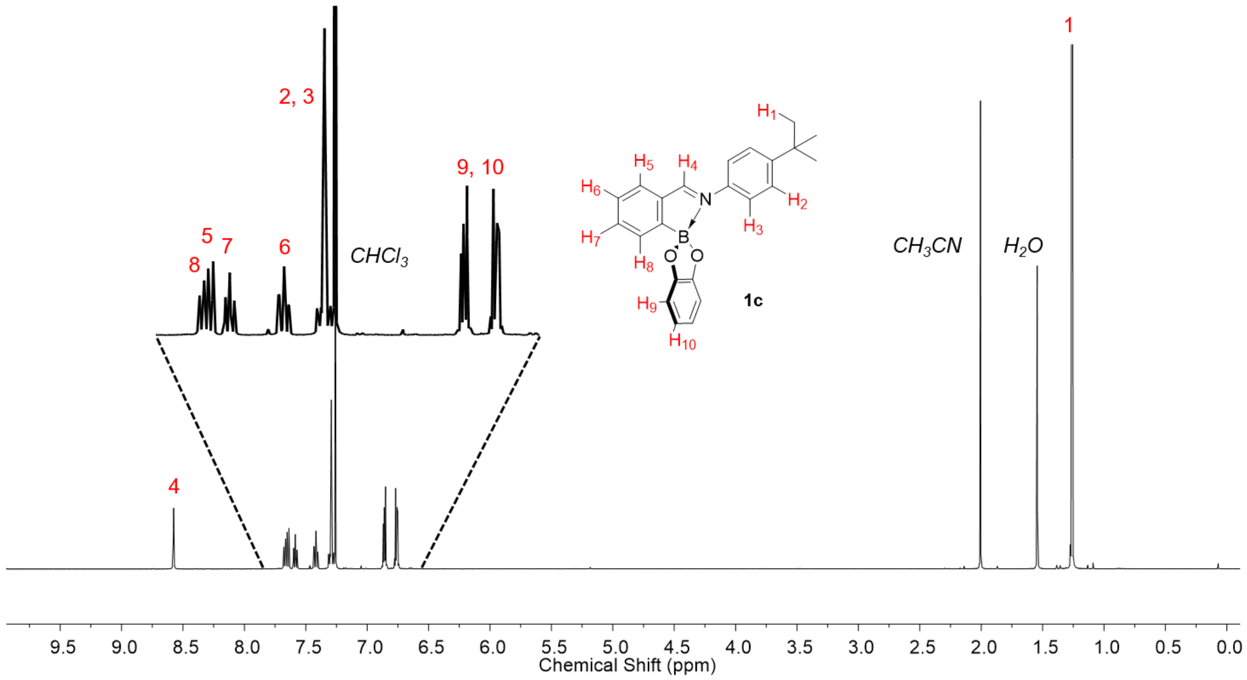

Figure S7. ^{1}H NMR spectrum (500 MHz, $CDCl_3$) of **1c**.

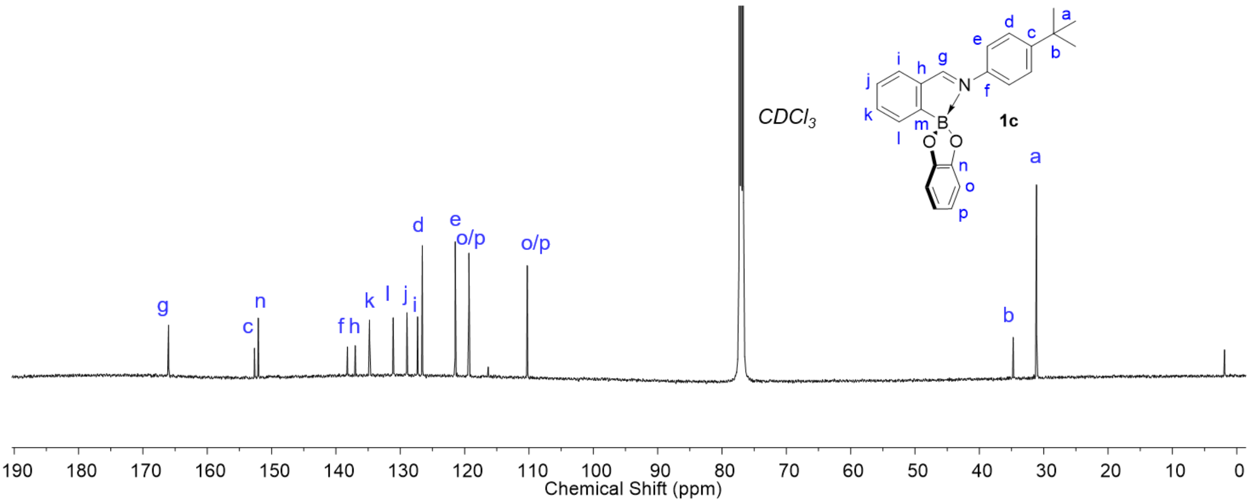

Figure S8. ^{13}C NMR spectrum (126 MHz, $CDCl_3$) of **1c**.

2.4 *N*-(4-Methoxyphenyl)iminoboronate pyrocatechol ester (1d)

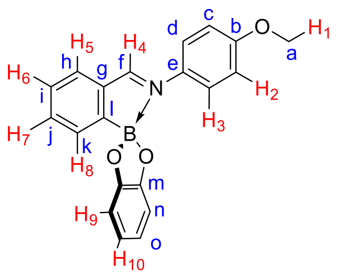

p-Anisidine (16.40 mg, 0.133 mmol) 2-formylphenylboronic acid (19.97 mg, 0.133 mmol) and pyrocatechol (14.69 mg, 0.133 mmol) were dissolved in CH_3CN (2 mL) and stirred at room temperature for 2.5 hours. The solvent was removed *in vacuo* to afford the desired product as a yellow brown solid (42.8 mg, >95%).

^{1}H NMR (500 MHz, 298.0 K, $CDCl_3$): δ 8.50 (1H, s, H_4), 7.67 (1H, d, ^3J = 7.5 Hz, H_8), 7.63 (1H, d, ^3J = 7.5 Hz, H_5), 7.58 (1H, td, ^3J = 7.5 Hz, ^4J = 1.1 Hz, H_7), 7.41 (1H, td, ^3J = 7.5 Hz, ^4J = 1.1 Hz, H_6), 7.31 (2H, d, ^3J = 9.0 Hz, H_2), 6.87-6.82 (2H, m, $H_{9/10}$), 6.79 (2H, d, ^3J = 9.1 Hz, H_3), 6.77-6.72 (2H, m, $H_{9/10}$), 3.76 (3H, s, H_1)

^{13}C NMR (126 MHz, 298.0 K, $CDCl_3$): δ 164.9 (C_f), 160.2 (C_b), 152.0 (C_m), 137.2 (C_g), 134.5 (C_j), 134.0 (C_e), 131.1 (C_k), 129.0 (C_i), 127.1 (C_h), 123.2 (C_c), 119.4 ($C_{n/o}$), 114.7 (C_d), 110.2 ($C_{n/o}$), 55.5 (C_a)

^{11}B NMR (128 MHz, 298.0 K, CD_3CN): δ 15.34 (bs)

EI: *m/z* 314.10 $[M\text{-}CH_3]^{\cdot+}$, 329.12 $[M]^+$

HRMS-EI: *m/z* calcd for $C_{20}H_{16}{}^{11}BNO_3$ $[M]^+$ 329.12232, found $[M]^+$ 329.12229 (-0.10 ppm)

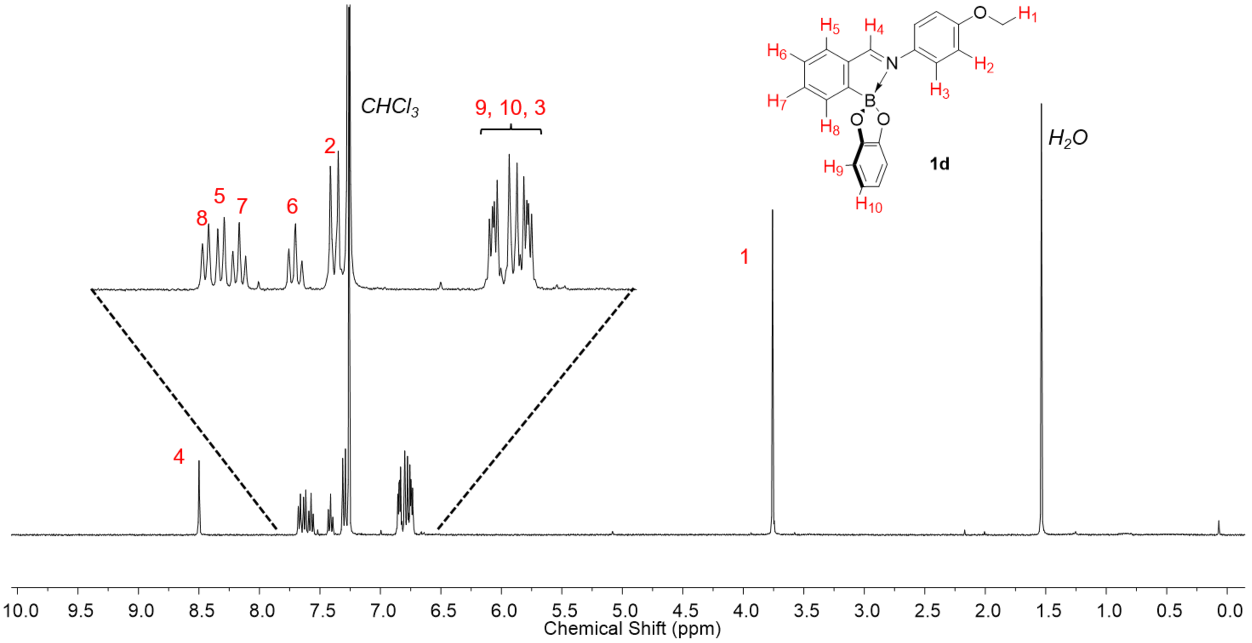

Figure S9. ^{1}H NMR spectrum (500 MHz, $CDCl_3$) of **1d**.

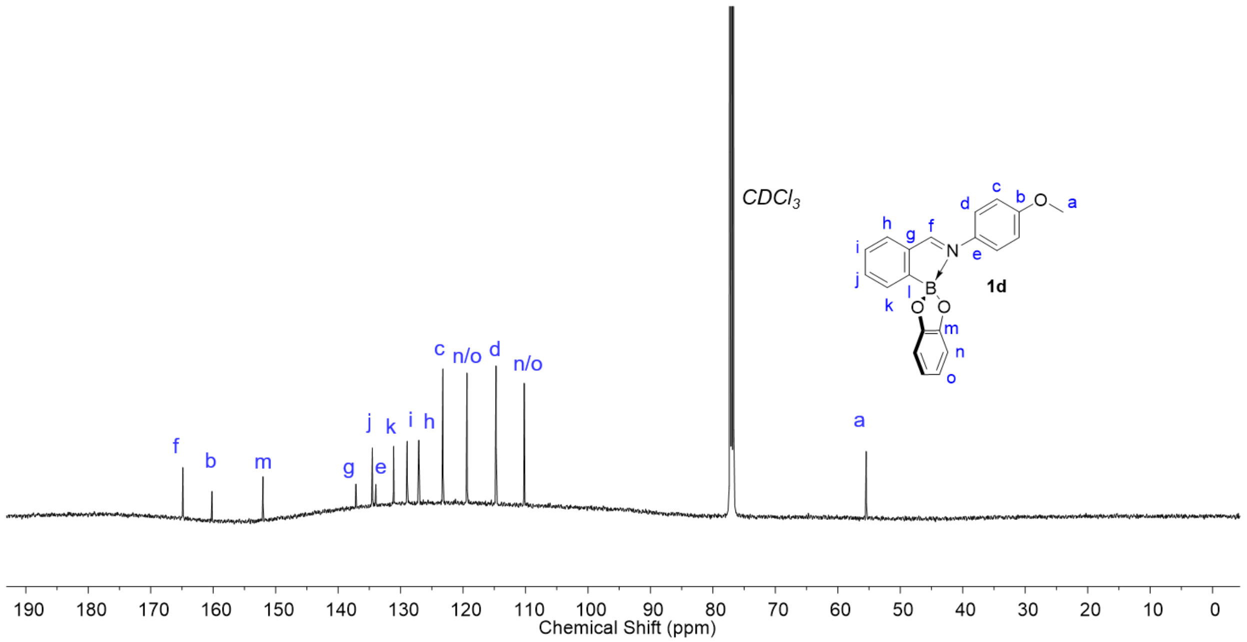

Figure S10. ^{13}C NMR spectrum (126 MHz, $CDCl_3$) of **1d**.

2.5 *N*-Tolyliminoboronate tetrachlorocatechol ester (1e)

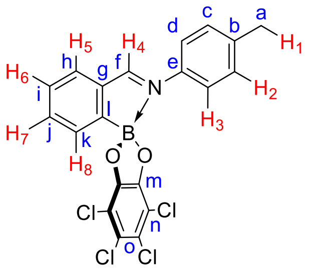

2-Formylphenylboronic acid (10.00 mg, 0.0667 mmol), toluidine (7.15 mg, 0.0667 mmol), and tetrachlorocatechol monohydrate (17.73 mg, 0.0667 mmol) were dissolved in CH_3CN (2 mL) and stirred at room temperature for 2 hours. The solvent was removed *in vacuo* to afford the desired product as a yellow solid (26.2 mg, 87%).

^{1}H NMR (400 MHz, 298.0 K, $CDCl_3$): δ 8.54 (1H, s, H_4), 7.69-7.64 (2H, m, $H_{5/8}$), 7.64 (1H, t, ^3J = 7.3 Hz, H_7), 7.47 (1H, td, ^3J = 7.5 Hz, ^4J = 1.2 Hz, H_6), 7.23-7.16 (4H, m, $H_{2/3}$), 2.37 (3H, s, H_1)

^{13}C NMR (101 MHz, 298.0 K, $CDCl_3$): δ 168.9 (C_f), 148.9 (C_m), 140.3 (C_b), 137.9 (C_e), 137.2 (C_g), 135.4 (C_j), 131.7 (C_k), 130.7 (C_c), 129.7 (C_i), 127.9 (C_h), 122.3 ($C_{n/o}$), 121.9 (C_d), 113.6 ($C_{n/o}$), 21.3 (C_a)

^{11}B NMR (128 MHz, 298.0 K, CD_3CN): δ 14.81 (bs)

HRMS-EI: *m/z* calcd for $C_{20}H_{12}{}^{11}B^{35}Cl_4NO_2$ $[M]^+$ 450.96857, found $[M]^+$ 450.96785 (-1.59 ppm)

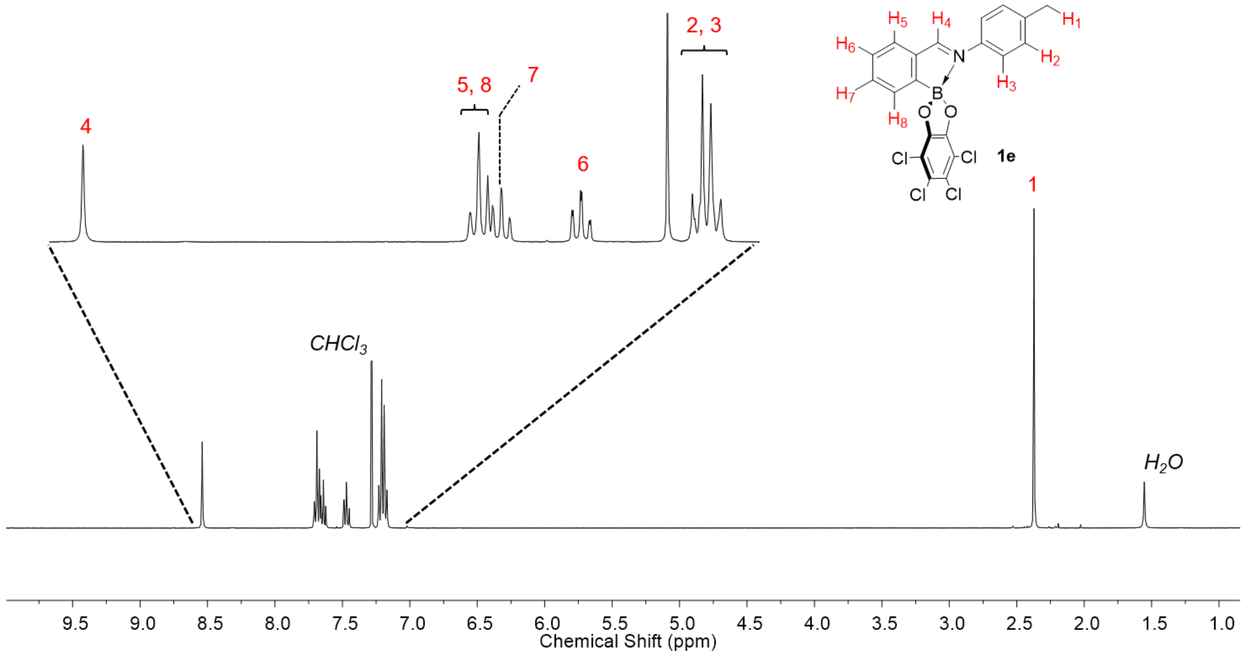

Figure S11. ^{1}H NMR spectrum (400 MHz, $CDCl_3$) of **1e**.

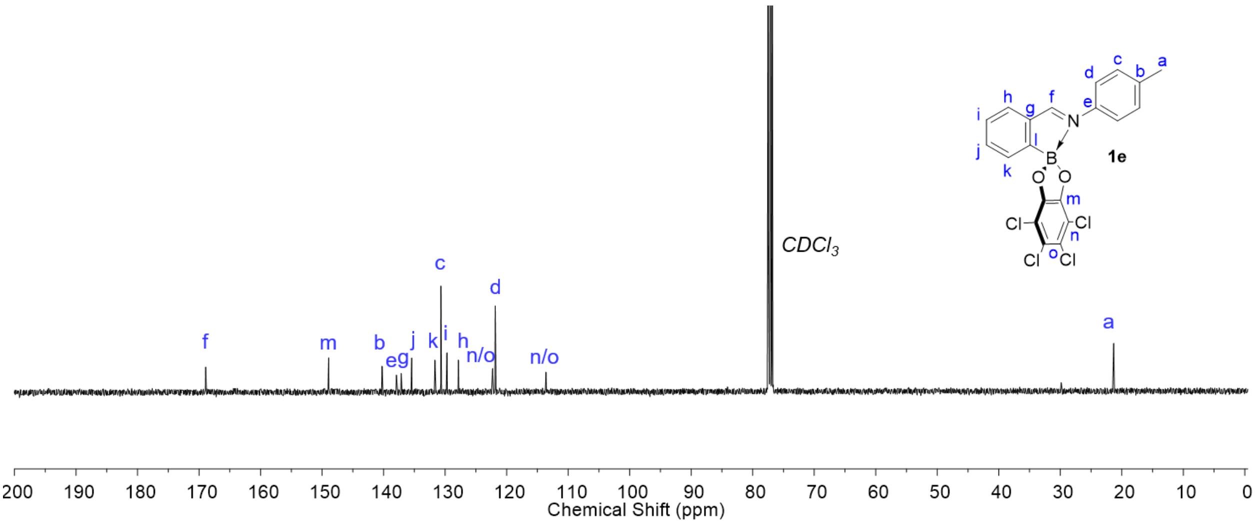

Figure S12. ^{13}C NMR spectrum (101 MHz, $CDCl_3$) of **1e**.

3 Solution Characterisation of the Fluoroaniline-Pyrocatechol Reductively Coupled Dimer (2a)

3.1 *rac*$_5$-2a and *meso*$_5$-2a Mixture in CD_3CN

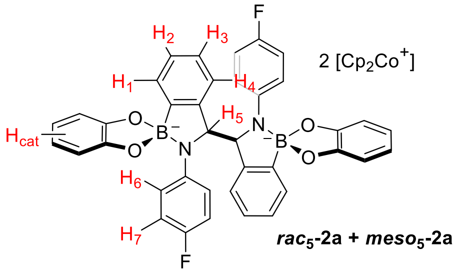

In a nitrogen atmosphere glove box, Cp_2Co (3.00 mg, 0.016 mmol) was dissolved in 0.5 mL of CD_3CN. This solution was then agitated with **1a** (5.00 mg, 0.016 mmol) until the solid was fully dissolved and transferred to a J. Young NMR tube.

Reaction progress was monitored by NMR spectroscopy. A loss of the imine signal and the appearance of the two methine signals 5 and 5' around 5.5 ppm were observed in the ^{1}H NMR spectrum (Figure S13). The ***rac*$_5$-2a** (protons 1-7) and ***meso*$_5$-2a** (protons 1'-7') products were subsequently identified and characterised through a combination of X-ray crystallography and NMR spectroscopy (Sections 3.2-3.5). Full assignment of the signals for each isomer was not carried out due to the overlapping signals.

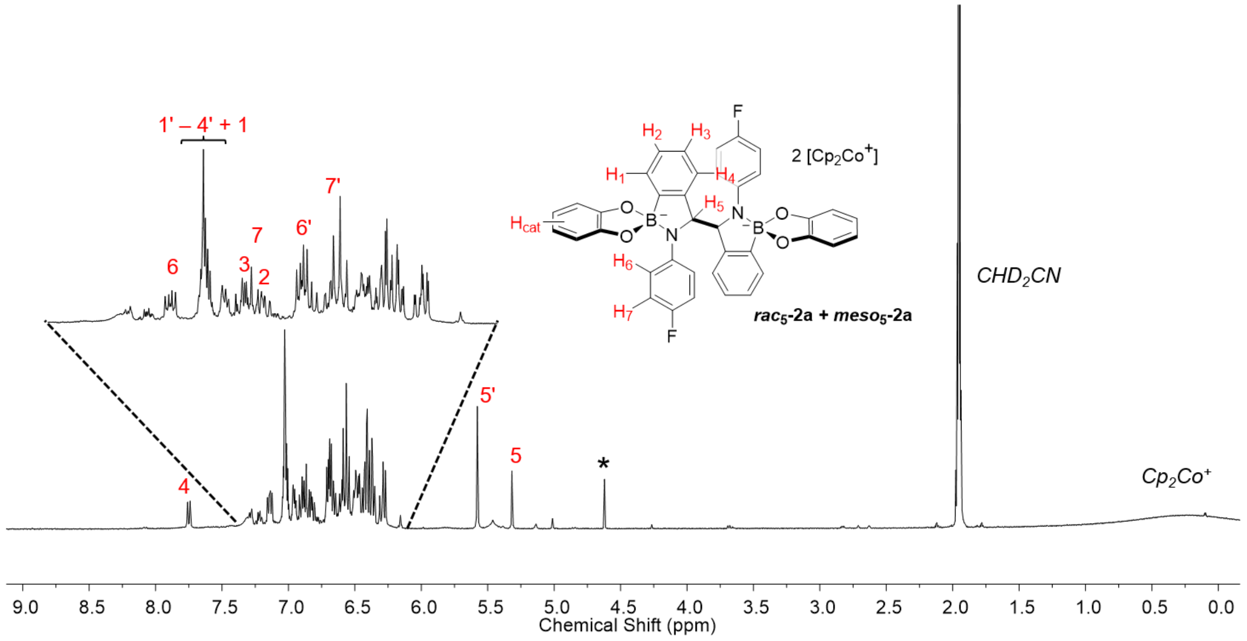

Figure S13. ^{1}H NMR spectrum (400 MHz, CD_3CN) of the reaction mixture from the reductive coupling of **1a**. Protons 1-7 correspond to ***rac*$_5$-2a** and protons 1'-7' to ***meso*$_5$-2a**. * is attributed to a transient Cp_2Co^+ species.

The ^{19}F NMR spectrum showed the loss of the fluorine signal for **1a** at -111.17 ppm and the appearance of several new fluorine-containing species between -130 ppm and -140 ppm (Figure S14). The two major species were subsequently identified as the ***rac*$_5$-2a** and ***meso*$_5$-2a** products.

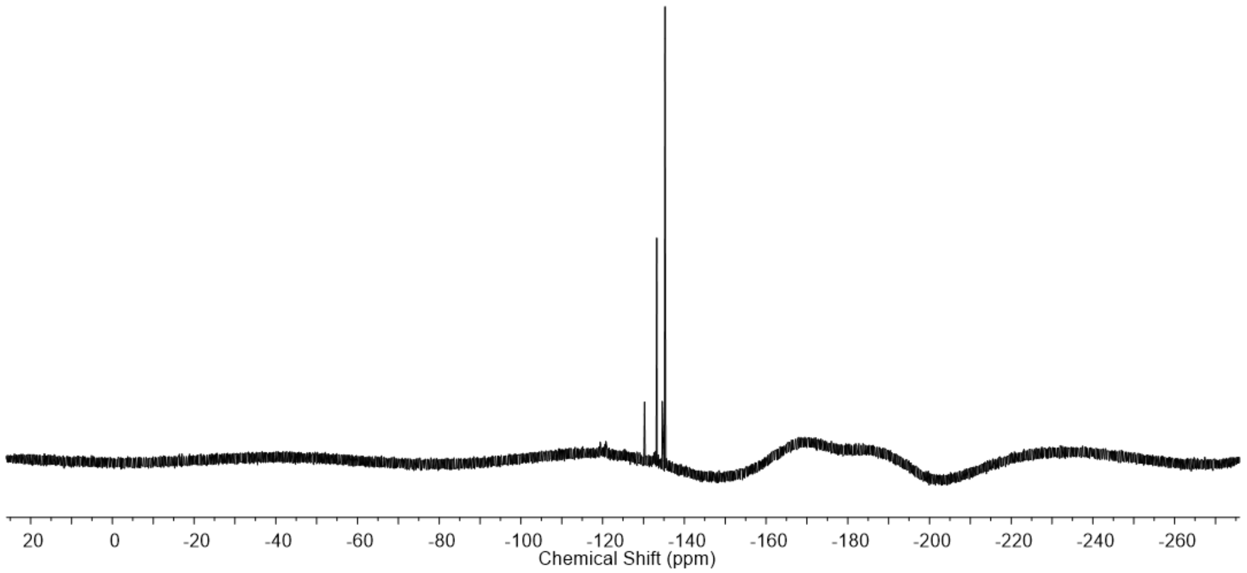

Figure S14. ^{19}F NMR spectrum (376 MHz, CD_3CN) of the reaction mixture from the reductive coupling of **1a**.

The ^{11}B NMR spectrum showed broad peaks around 14 ppm, consistent with the formation of a tetrahedrally coordinated boron complex (Figure S15).

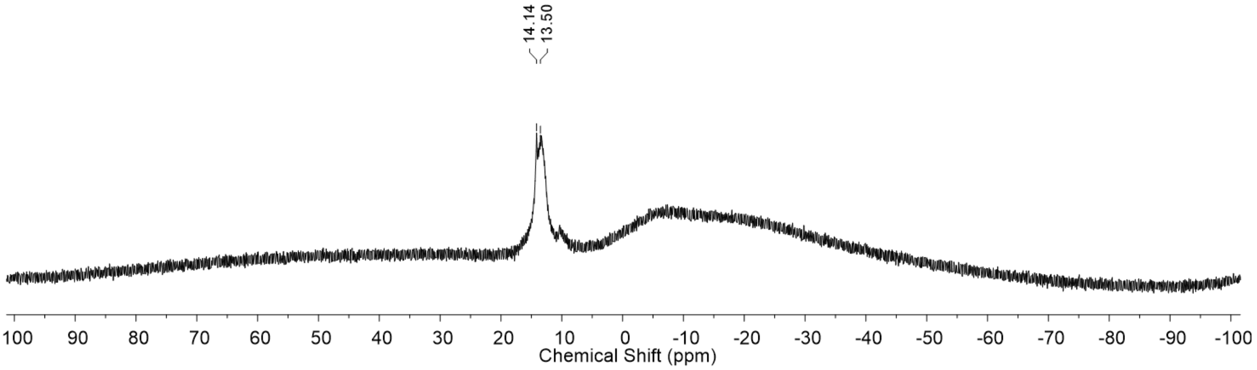

Figure S15. ^{11}B NMR spectrum (128 MHz, CD_3CN) of the reaction mixture from the reductive coupling of **1a**.

3.2 ***rac*₅-2a, r*ac*₆-2a and *meso*₅-2a Mixture in DMSO-*d*₆**

In a nitrogen atmosphere glove box, Cp_2Co (3.00 mg, 0.016 mmol) was dissolved in 0.5 mL of DMSO-d_6. This solution was then agitated with **1a** (5.03 mg, 0.016 mmol) until the solid was fully dissolved and transferred to a J. Young NMR tube.

***rac*$_6$-2a** (Section 3.3) and ***meso*$_5$-2a** (Section 3.4) were subsequently characterised by dissolving isolated crystals in DMSO-d_6, allowing identification of these isomers in the reaction mixture (Figures S16, S17). Furthermore, the redissolved ***rac*$_6$-2a** crystals were observed to interconvert to ***rac*$_5$-2a** and thus, all three products were characterised in DMSO-d_6. Due to the similarity of the chemical shifts of the coupled products in DMSO-d_6 and CD_3CN, the two products of the reaction in CD_3CN were identified as ***meso*$_5$-2a** and ***rac*$_5$-2a** (Figure S18).

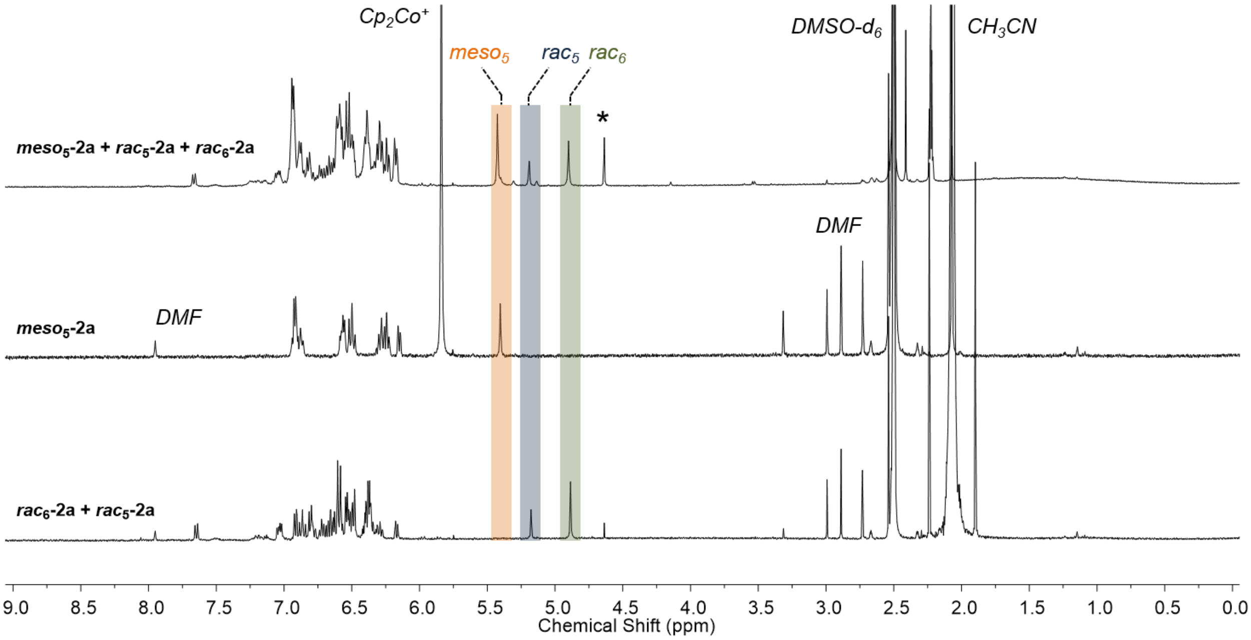

Figure S16. Stacked ^{1}H NMR spectra (400 MHz, DMSO-d_6) of: the **2a** mixture formed in DMSO-d_6 (top); ***meso*₅-2a** (middle); ***rac*₆-2a** and ***rac*₅-2a** after partial interconversion (bottom). * is attributed to a transient Cp_2Co^+ species.

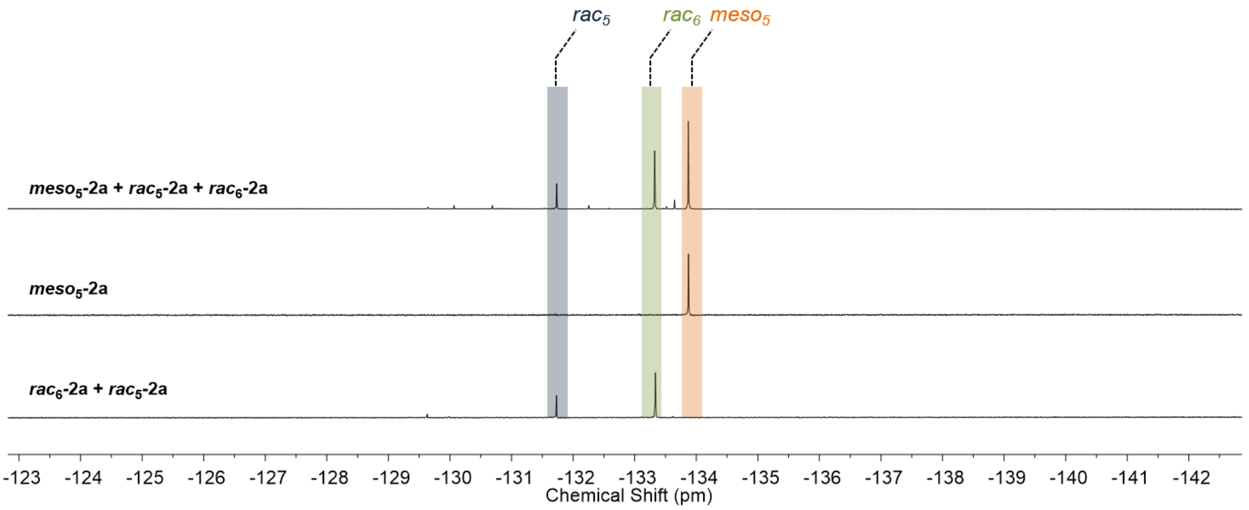

Figure S17. Stacked ^{19}F NMR spectra (376 MHz, DMSO-d_6) of: the **2a** mixture formed in DMSO-d_6 (top); ***meso*₅-2a** (middle); ***rac*₆-2a** and ***rac*₅-2a** after partial interconversion (bottom).

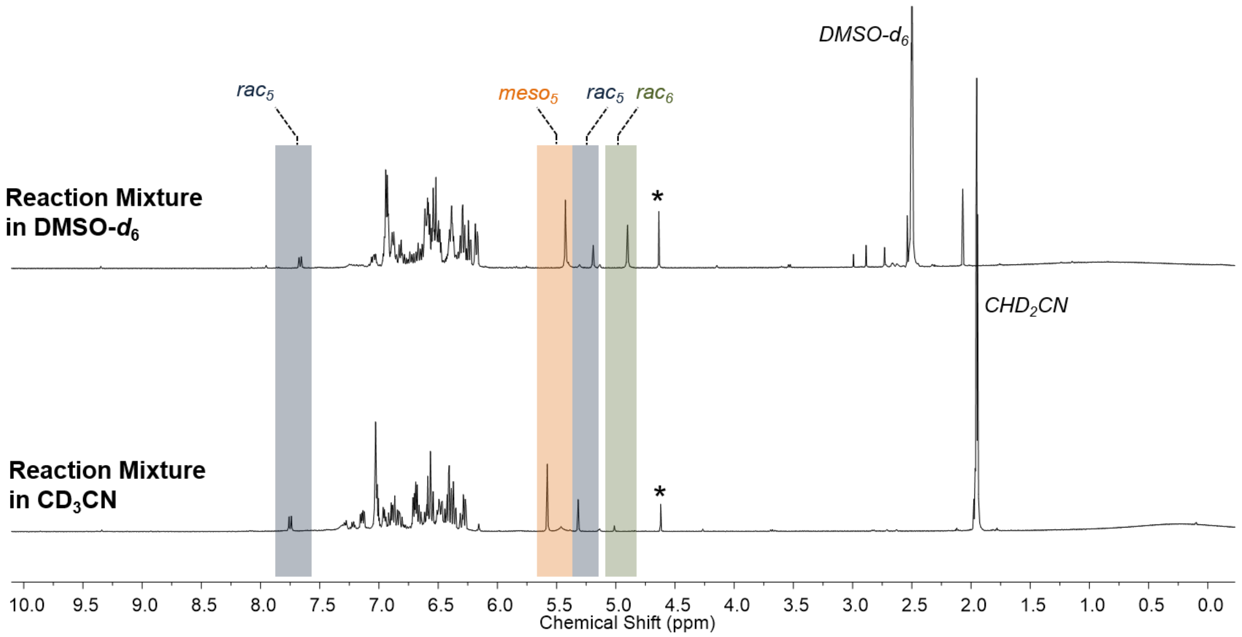

Figure S18. Stacked ^{1}H NMR spectra (400 MHz) of the **2a** mixture formed in DMSO-d_6 (top) and CD_3CN (bottom) showing the similarity of the chemical shift patterns of the ***rac*$_5$-2a** and ***meso*$_5$-2a** products in both solvents. This allowed the identification of these isomers as the products from the reaction in CD_3CN. * is attributed to a transient species of Cp_2Co^+.

3.3 ***meso*$_5$-2a**

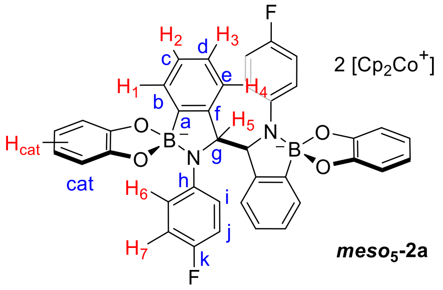

In a nitrogen atmosphere glove box, Cp_2Co (6.00 mg, 0.032 mmol) was dissolved in 0.5 mL of CH_3CN. This solution was then agitated with **1a** (5.00 mg, 0.017 mmol) until the solid had fully dissolved and left to crystallise for several days at room temperature. The solution was decanted and the remaining crystals were washed twice with CH_3CN (ca. 1 mL) and redissolved in 0.5 mL DMSO-d_6.

^{1}H NMR (400 MHz, 298.0 K, DMSO-d_6): δ 6.97-6.85 (8H, m, $H_{1\text{-}4}$), 6.58 (4H, dd, $^3J_{6,7}$ = 9.0 Hz, $^4J_{HF}$ = 4.9 Hz, H_6), 6.51 (4H, t, $^3J_{6,7}$ = $^3J_{HF}$ = 9.0 Hz, H_7), 6.33-6.21 (6H, m, H_{cat}), 6.16 (2H, d, ^3J = 6.7 Hz, H_{cat}), 5.41 (2H, s, H_5)

^{13}C NMR (126 MHz, 298.0 K, DMSO-d_6): δ 155.3 (C_{cat}), 154.4 (C_{cat}), 152.8 (d, $^1J_{CF}$ = 227 Hz, C_k), 150.1 (C_a), 147.6 (C_h), 147.1 (C_f), 127.8 ($C_{b/d}$), 125.0 ($C_{b/d}$), 124.7 (C_c), 122.7 (C_e), 116.7 (d, $^3J_{CF}$ = 6 Hz, C_i), 115.9 (C_{cat}), 115.7 (C_{cat}), 113.3 (d, $^2J_{CF}$ = 21 Hz, C_j), 107.1 (C_{cat}), 106.2 (C_{cat}), 84.7 (Cp_2Co^+), 64.5 (C_g)

^{19}F NMR (376 MHz, 298.0 K, DMSO-d_6): δ -133.873 (s)

It was not possible to obtain the boron chemical shift due to the small quantity of the isolated crystals and the broadness of boron peaks.

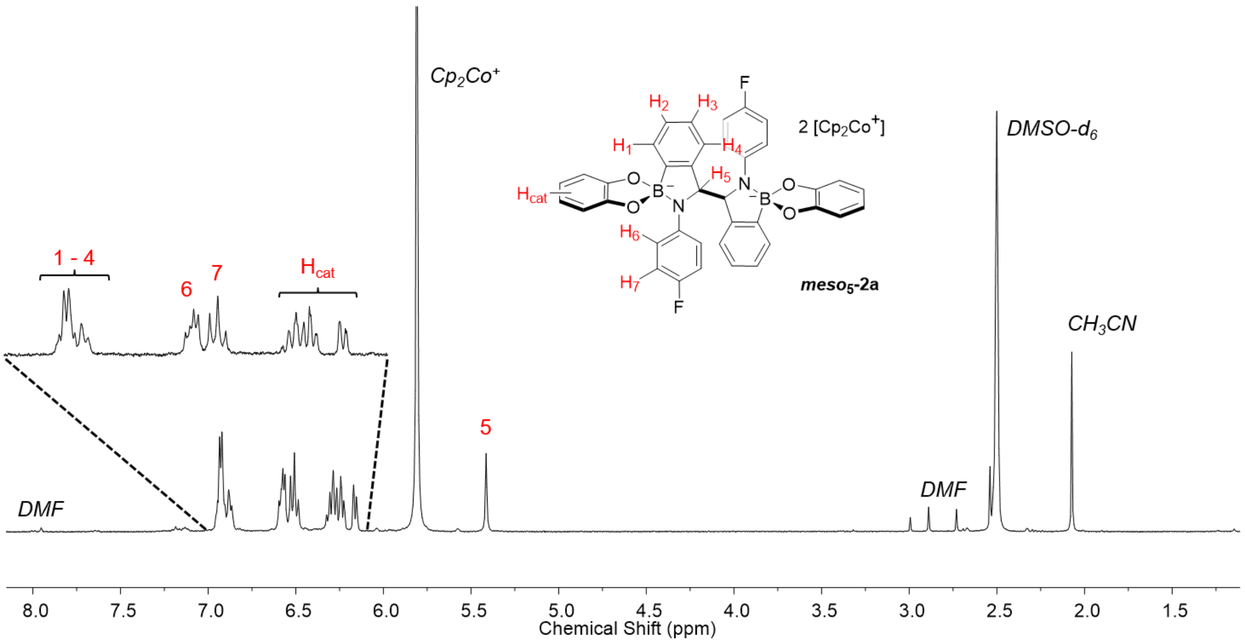

Figure S19. ^{1}H NMR spectrum (400 MHz, DMSO-d_6) of ***meso*₅-2a** crystals.

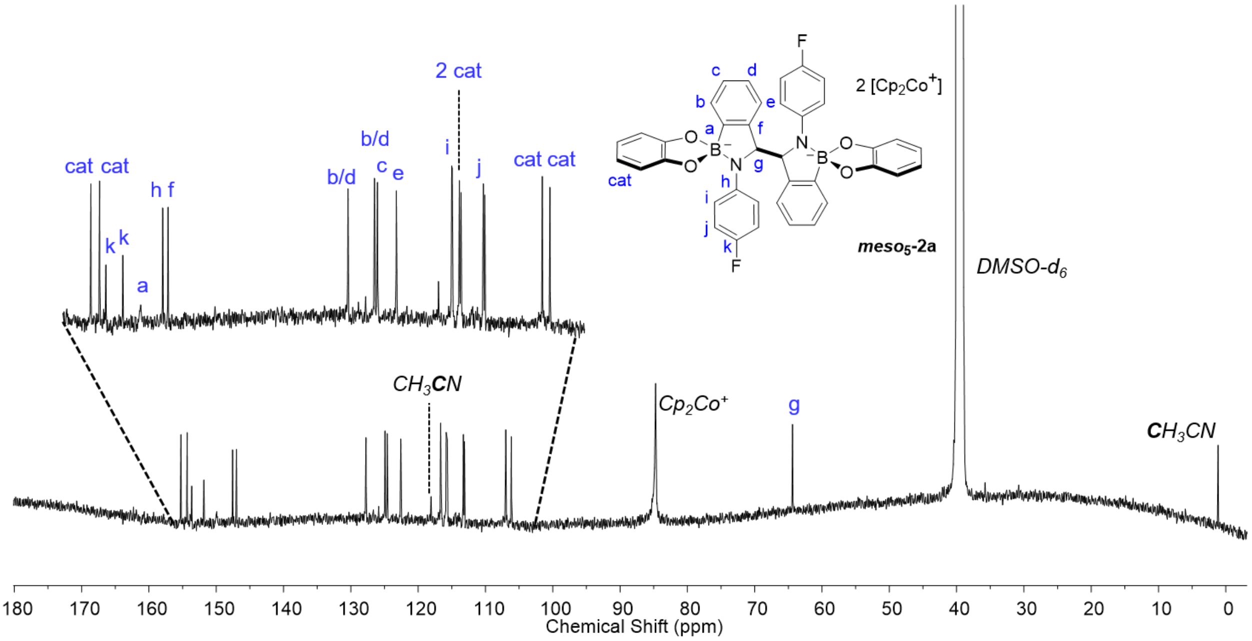

Figure S20. ^{13}C NMR spectrum (126 MHz, DMSO-d_6) of ***meso*₅-2a** crystals.

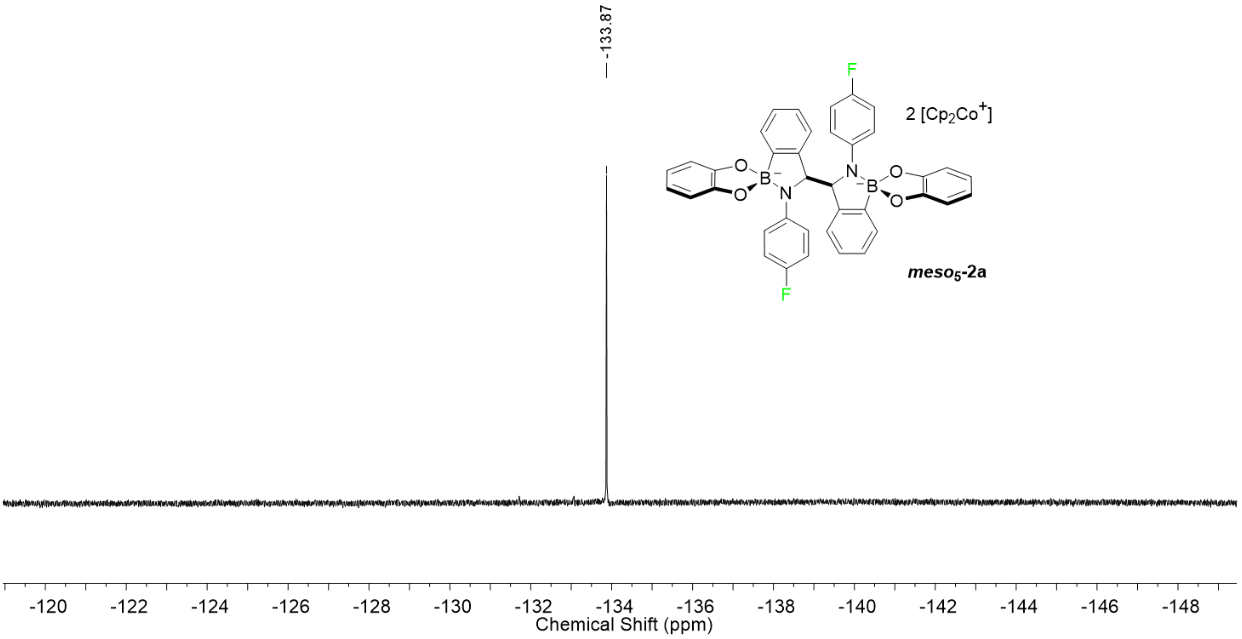

Figure S21. ^{19}F NMR spectrum (376 MHz, DMSO-d_6) of ***meso*₅-2a** crystals.

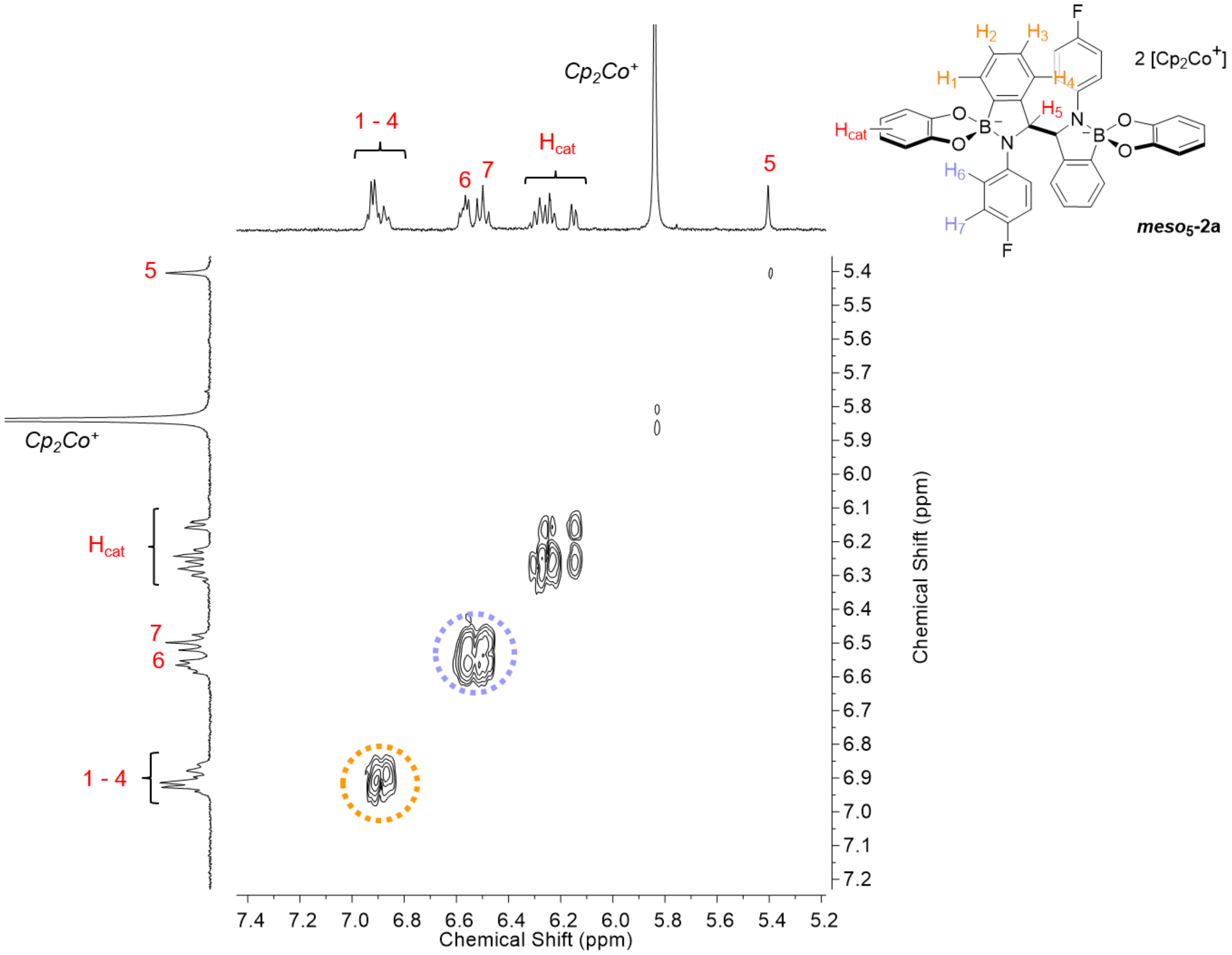

Figure S22. COSY NMR spectrum (400 MHz, DMSO-d_6) of ***meso*$_5$-2a** crystals.

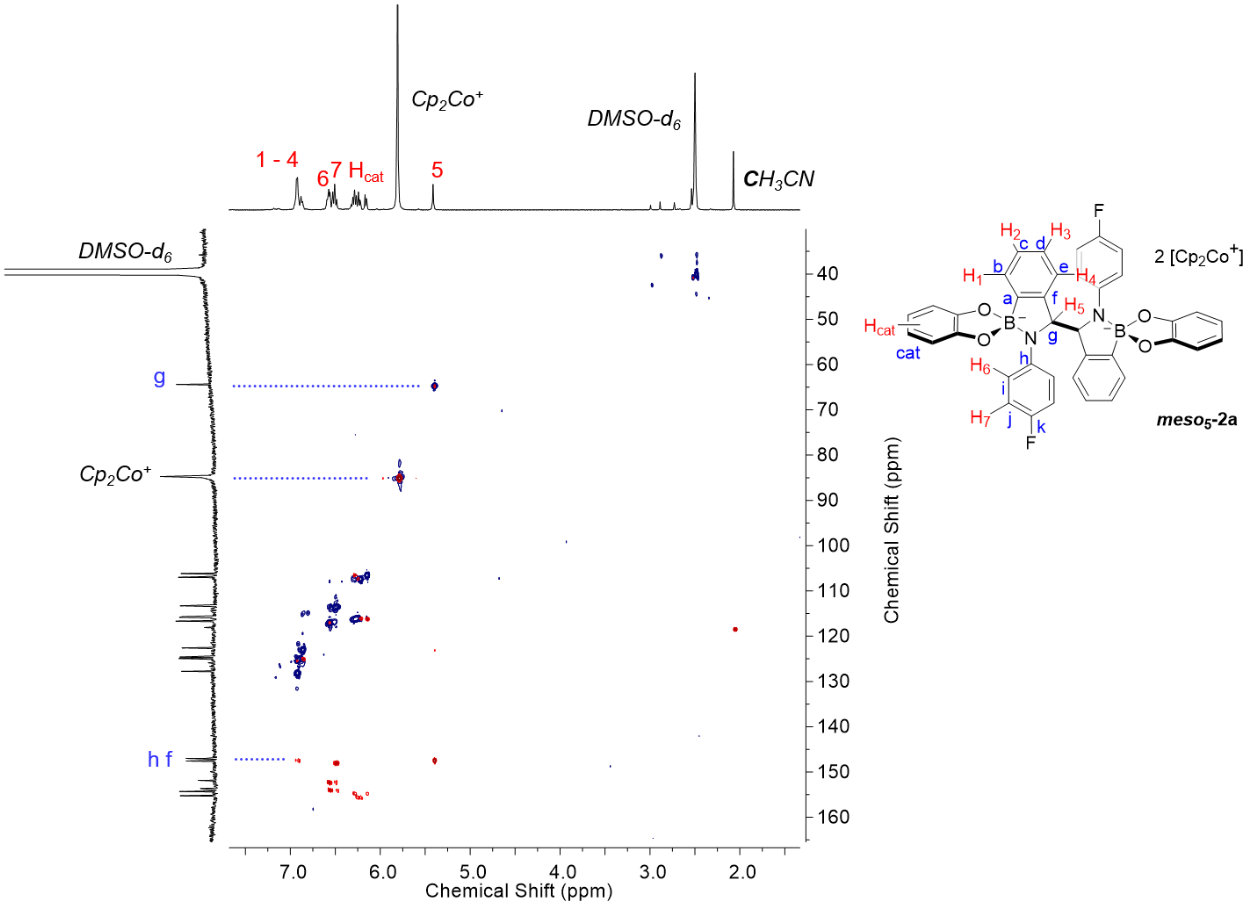

Figure S23. Overlay of the HSQC (blue) and HMBC (red) spectra (DMSO-d_6) of ***meso*$_5$-2a** crystals showing key assignments.

3.4 *rac*$_6$-2a

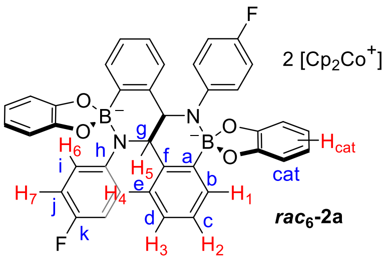

1a (11.62 mg, 0.37 mmol) was treated with Cp_2Co (6.93 mg, 0.37 mmol) in CD_3CN (1 mL) in a nitrogen filled glove box at room temperature. Crystals grown from the unperturbed reaction mixture were isolated by decanting the solution. They were washed twice with CH_3CN (ca. 1 mL) and redissolved in 0.5 mL DMSO-*d*$_6$.

While the ^{1}H NMR spectrum of the redissolved ***rac*$_6$-2a** crystals showed one species initially (Figure S24), a new set of signals grew over time corresponding to the ***rac*$_5$-2a** isomer (see Section 3.5). Due to this interconversion to the ***rac*$_5$-2a** isomer, it was not possible to record ^{13}C, ^{19}F and 2D NMR spectra of the ***rac*$_6$-2a** isomer alone. The ^{13}C chemical shifts reported below were assigned from this mixture.

^{1}H NMR (400 MHz, 298.0 K, DMSO-*d*$_6$): δ 6.92 (2H, d, ^3J = 7.3 Hz, H_1), 6.81 (2H, d, ^3J = 7.4 Hz, H_4), 6.66 (2H, td, ^3J = 7.4 Hz, ^4J = 1.2 Hz, H_3), 6.63-6.57 (6H, m, H_{7+2}), 6.52 (4H, dd, $^3J_{6,7}$ = 9.4 Hz, $^4J_{HF}$ = 4.8 Hz, H_6), 6.51-6.47 (2H, m, H_{cat}), 6.43-6.33 (6H, m, H_{cat}), 4.90 (2H, s, H_5)

^{13}C NMR (126 MHz, 298.0 K, DMSO-*d*$_6$): δ 156.2 (C_{cat}), 153.9 (C_{cat}), 152.8 (d, $^2J_{CF}$ = 228 Hz, C_k), 149.7 (C_h), 142.7 (C_f), 128.8 (C_b), 124.2 ($C_{d,e}$), 123.6 (C_c), 116.4 (C_{cat}), 116.1 (C_{cat}), 115.6 (d, $^4J_{CF}$ = 6 Hz), 113.5 (d, $^3J_{CF}$ = 21 Hz), 108.0 (C_{cat}), 106.6 (C_{cat}), 64.4 (C_g)

^{19}F NMR (376 MHz, 298.0 K, DMSO-*d*$_6$): δ -133.33 (s)

It was not possible to obtain the boron chemical shift due to the small quantity of the isolated crystals and the broadness of boron peaks.

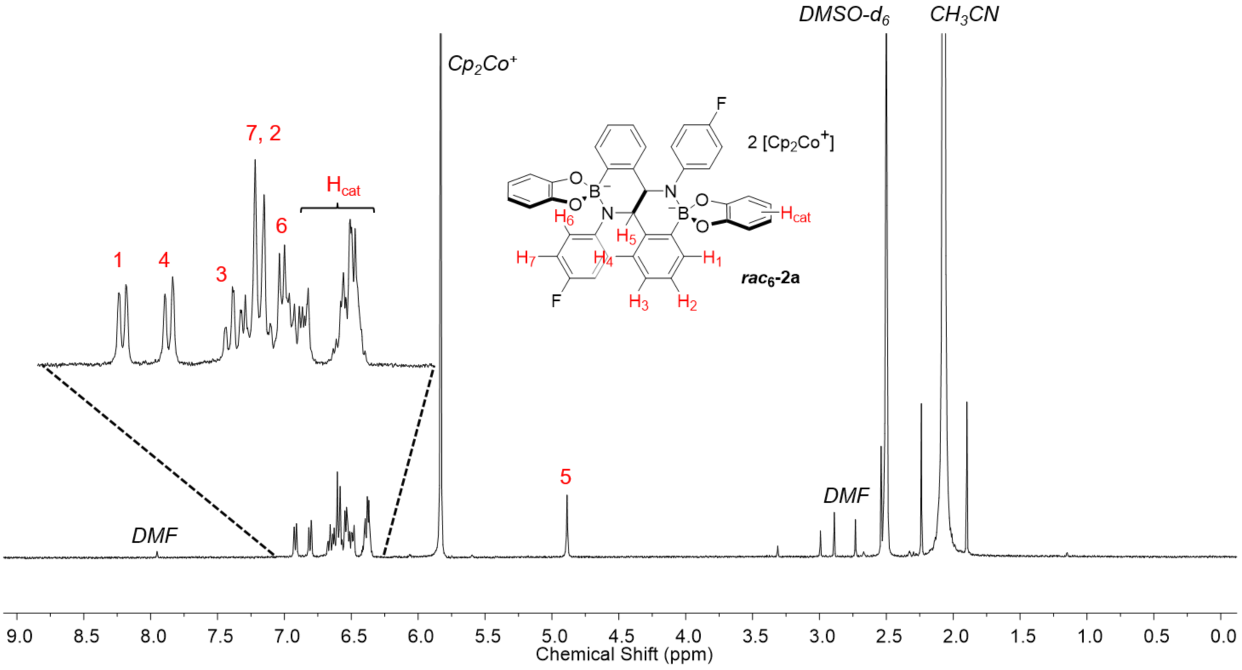

Figure S24. ^{1}H NMR spectrum (400 MHz, DMSO-*d*$_6$) of ***rac*$_6$-2a** crystals.

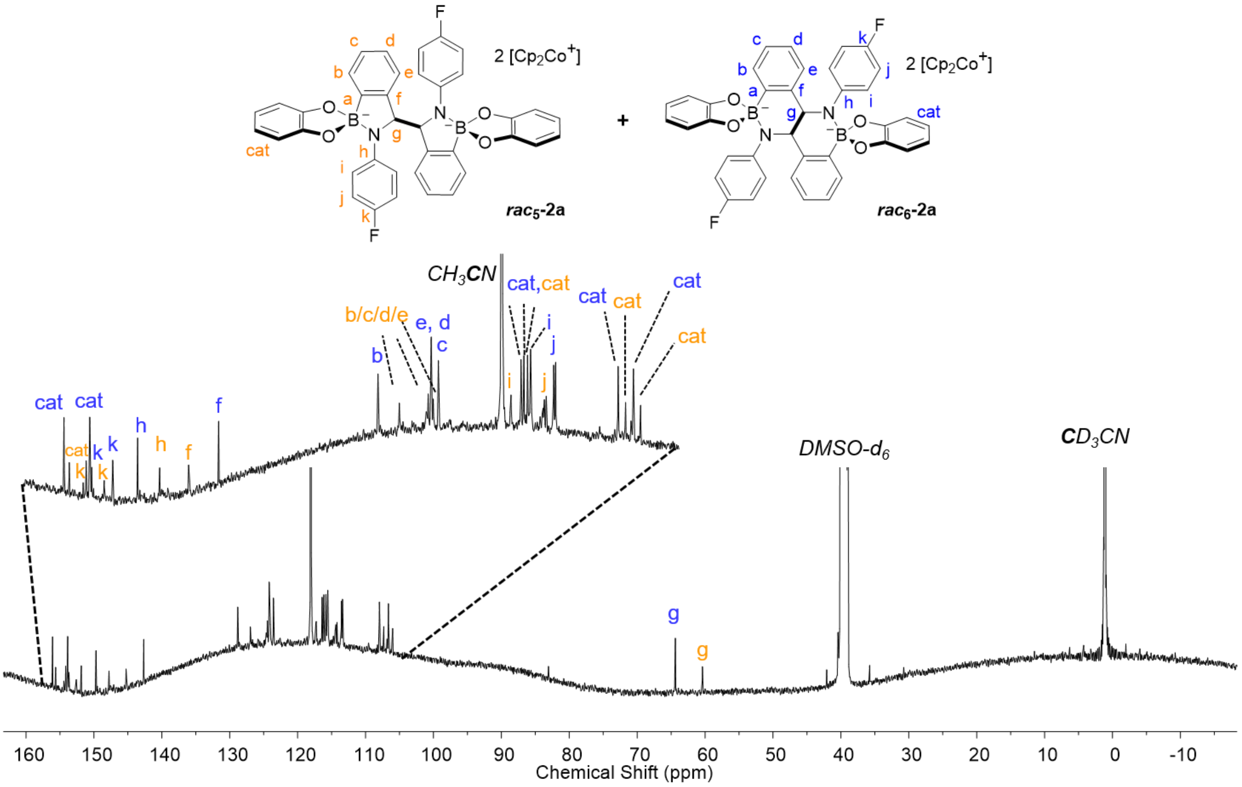

Figure S25. ^{13}C NMR spectrum (126 MHz, DMSO-d_6) of ***rac*$_6$-2a** and ***rac*$_5$-2a** after partial interconversion.

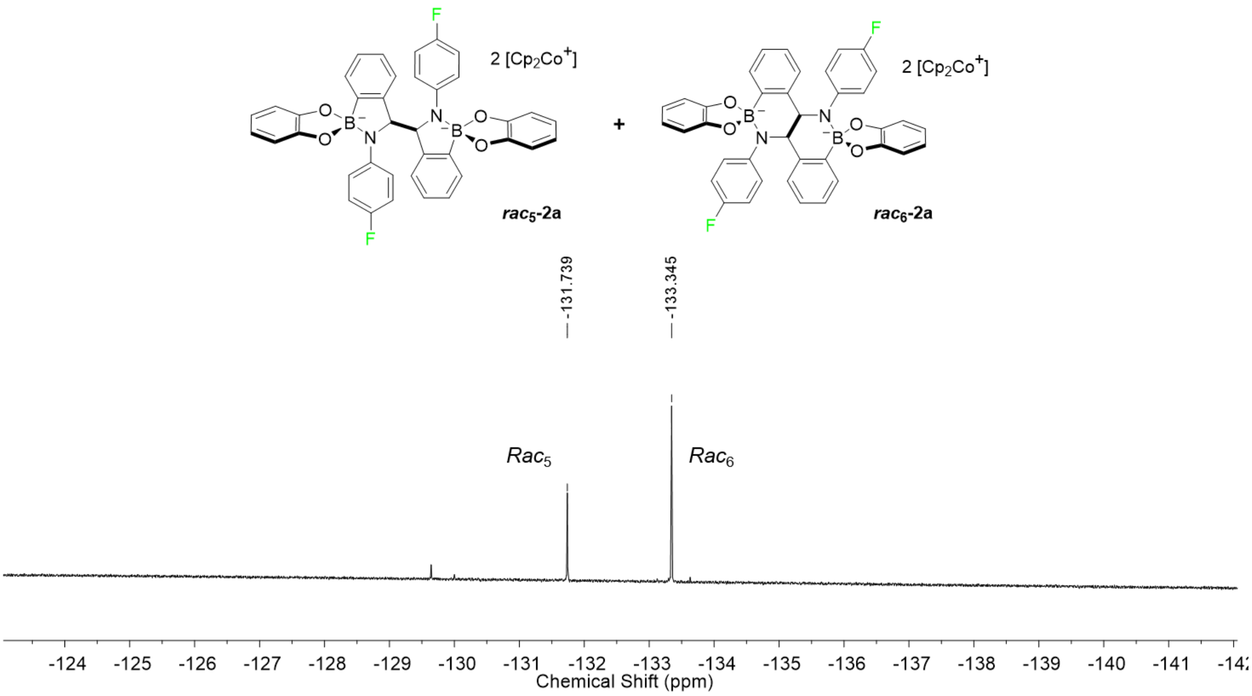

Figure S26. ^{19}F NMR spectrum (376 MHz, DMSO-d_6) of ***rac*$_6$-2a** and ***rac*$_5$-2a** mixture.

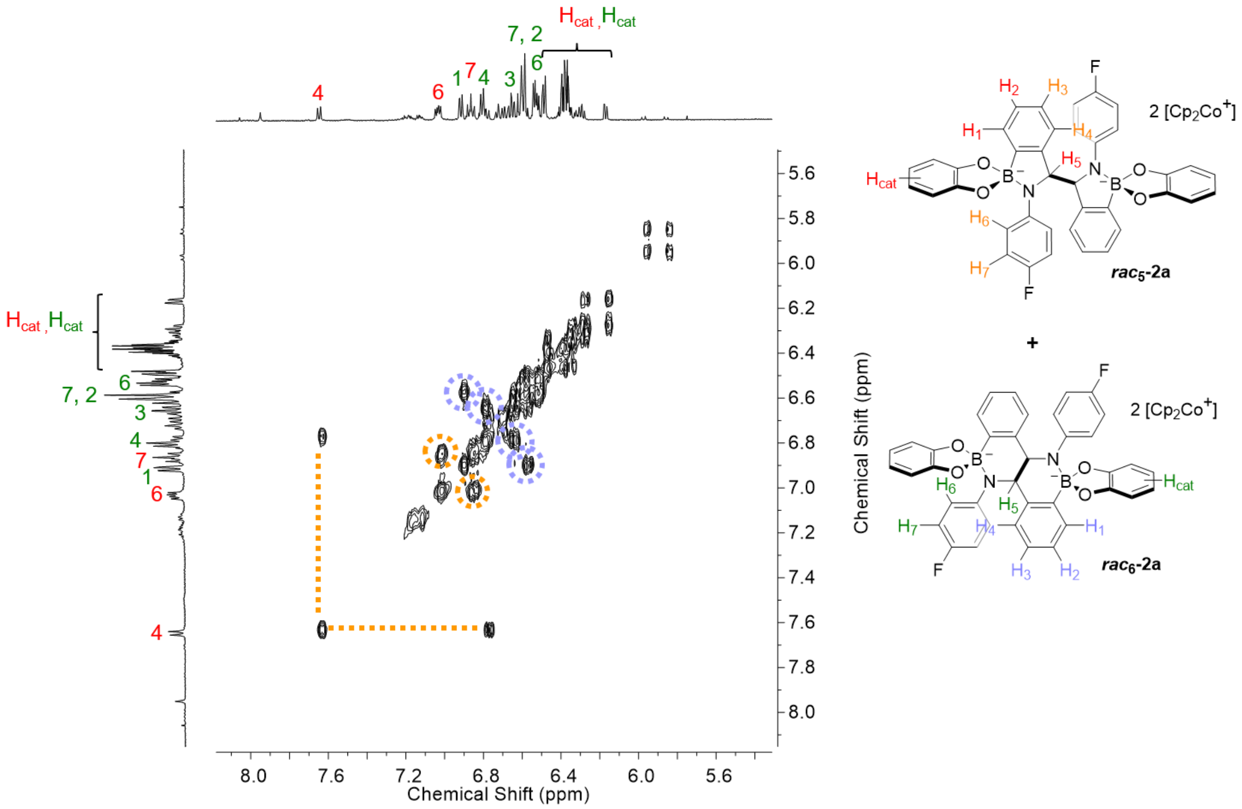

Figure S27. COSY NMR spectrum (500 MHz, DMSO-d_6) of ***rac*$_6$-2a** and ***rac*$_5$-2a** mixture.

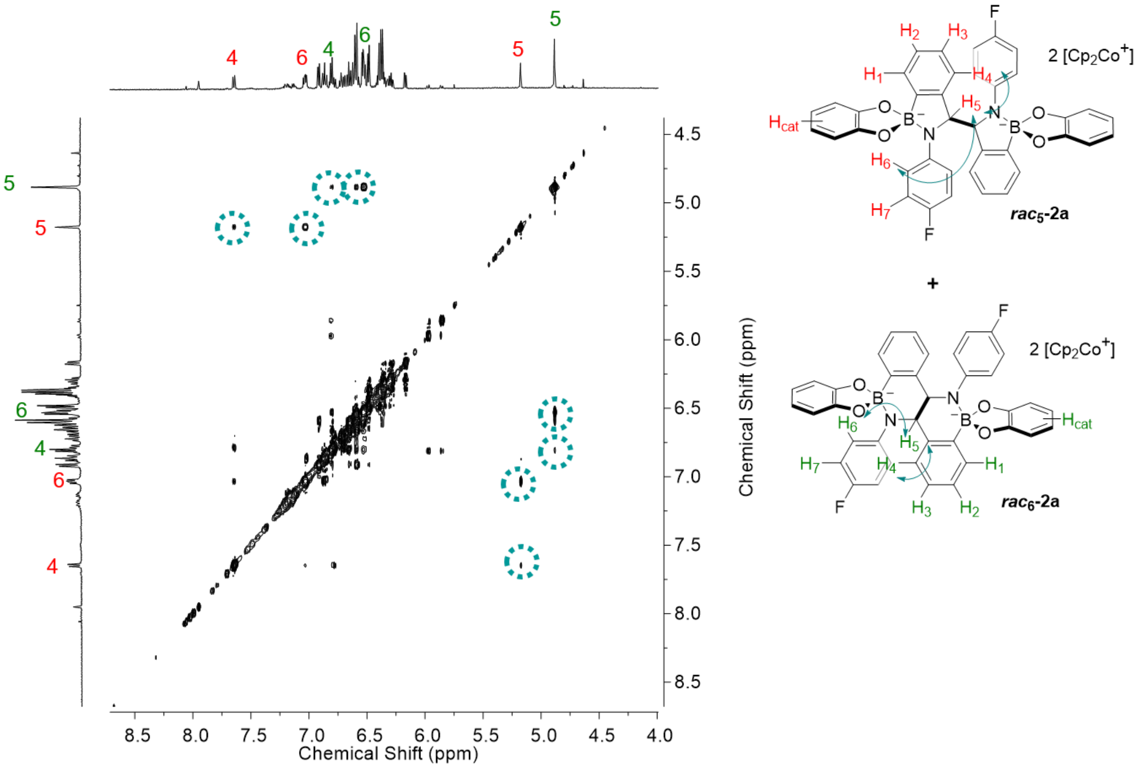

Figure S28. NOESY NMR spectrum (500 MHz, DMSO-d_6) of ***rac*$_6$-2a and *rac*$_5$-2a** after interconversion.

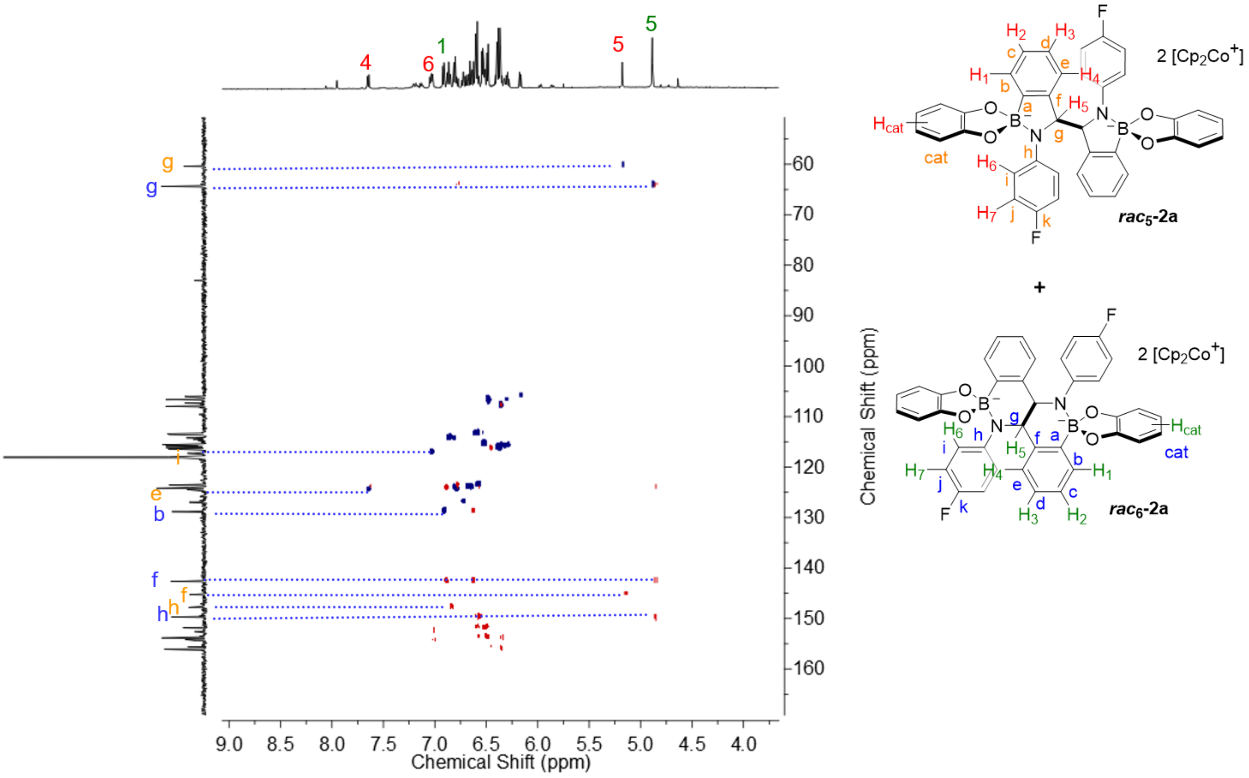

Figure S29. Overlay of HSQC and HMBC NMR spectra (500 MHz/126 MHz, DMSO-*d*$_6$) of ***rac*$_6$-2a and *rac*$_5$-2a** after interconversion.

3.5 *rac*$_5$-2a

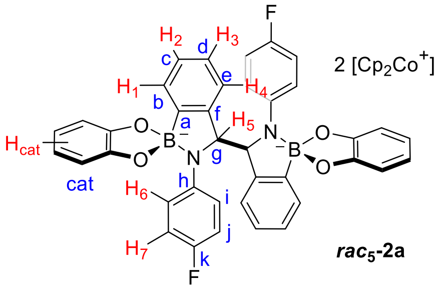

Characterisation data for the ***rac*$_5$-2a** isomer could be inferred from the NMR data of the equilibrated ***rac*$_5$-2a** and ***rac*$_6$-2a** mixture from Section 3.4. Due to the number of overlapping signals in the ^{1}H NMR spectrum of the mixture, the ^{1}H NMR chemical shifts are not reported below but Figure S30 shows the assignments. The ^{13}C NMR chemical shifts reported below were assigned from the ^{13}C NMR and HSQC/HMBC spectra (Figures S25, S29) and the ^{19}F NMR chemical shift was assigned from the spectrum in Figure S26.

^{13}C NMR (126 MHz, 298.0 K, DMSO-*d*$_6$): δ 155.6 (C_{cat}), 154.2 (C_{cat}), 153.4 (d, $^2J_{CF}$ = 232 Hz, C_k), 147.8 (C_h), 145.3 (C_f), 127.0 ($C_{b/c/d/e}$), 124.4 ($C_{b/c/d/e}$), 124.0 ($C_{b/c/d/e}$), 117.3 (d, $^4J_{CF}$ = 7 Hz, C_i), 115.8 (C_{cat}), 114.3 (d, $^3J_{CF}$ = 22 Hz, C_j), 107.3 (C_{cat}), 106.0 (C_{cat}), 60.4 (C_g)

^{19}F NMR (376 MHz, 298.0 K, DMSO-*d*$_6$): δ -131.74 (s)

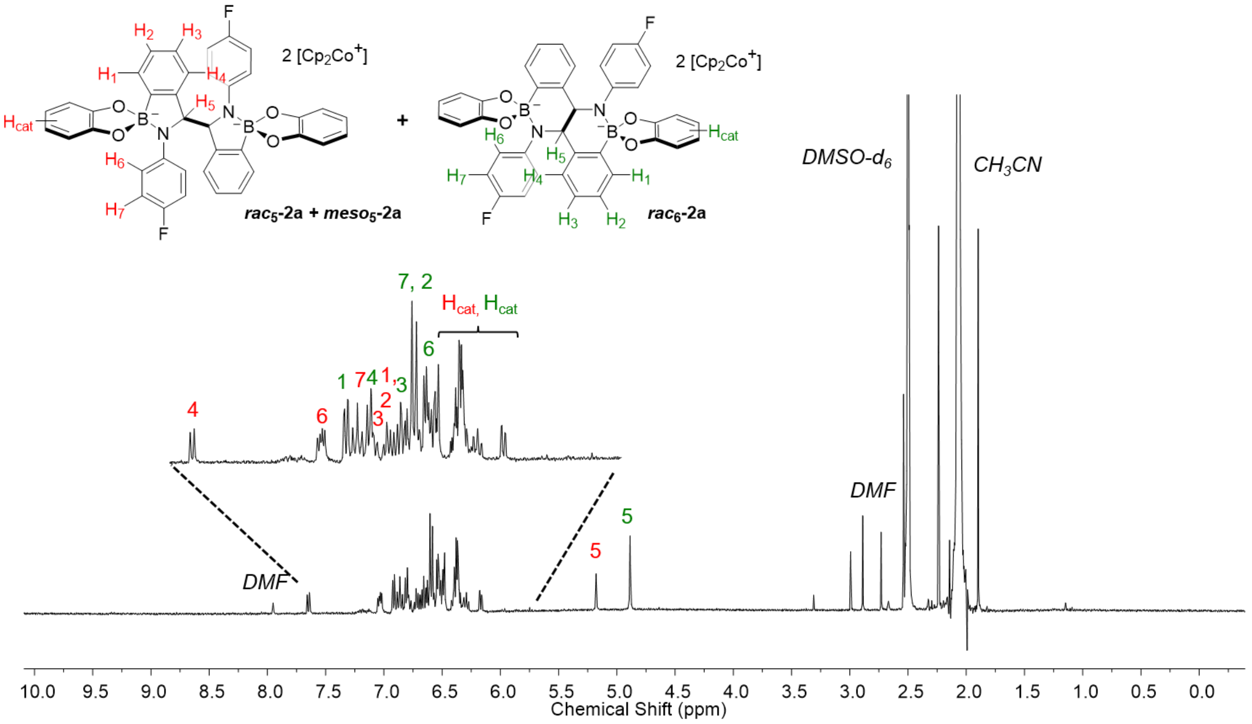

Figure S30. ^{1}H NMR spectrum (400 MHz, DMSO-d_6) of ***rac*$_6$-2a and *rac*$_5$-2a** after partial interconversion.

4 Solution Characterisation of the Toluidine-Pyrocatechol Reductively Coupled Dimer (2b)

4.1 *rac*$_5$-2b and *meso*$_5$-2b Mixture in CD_3CN

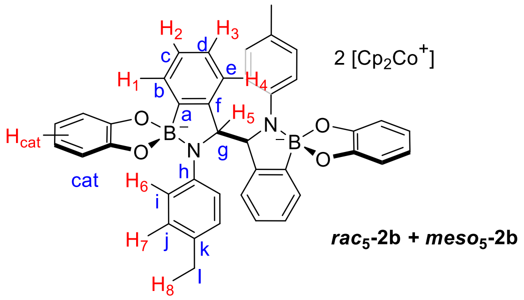

In a nitrogen atmosphere glove box, Cp_2Co (3.02 mg, 0.016 mmol) was dissolved in 0.5 mL of CD_3CN. This solution was then agitated with **1b** (5.00 mg, 0.016 mmol) until the solid was fully dissolved and transferred to a J. Young NMR tube.

As ***meso*$_5$-2b** was not observed to crystallise from the reaction mixture, it was characterised in solution as a mixture with ***rac*$_5$-2b**. The signals in the ^{1}H NMR spectrum could be assigned to the two isomers due to good dispersion of the signals. Signals marked with ' are attributed to ***meso*$_5$-2b**.

^{1}H NMR (400 MHz, 298.0 K, CD_3CN): δ 7.77 (2H, d, ^{3}J = 7.6 Hz, H_4), 7.13 (4H, d, ^{3}J = 8.4 Hz, H_6), 7.06-6.96 (8H, m, $H_{1'-4'}$), 6.91 (4H, d, ^{3}J = 8.4 Hz, H_7), 6.89 (2H, td, ^{3}J = 7.6 Hz, ^{4}J = 1.7 Hz, H_3), 6.85-6.77 (4H, m, $H_{1,2}$), 6.74 (4H, d, ^{3}J = 8.5 Hz, $H_{6'}$), 6.62 (4H, d, ^{3}J = 8.5 Hz, $H_{7'}$), 6.58 (2H, dd, ^{3}J = 7.3 Hz, ^{4}J = 1.3 Hz, H_{cat}), 6.47 (2H, td, ^{3}J = 7.5 Hz, ^{4}J =1.4 Hz, H_{cat}), 6.43-6.34 (8H, m, $H_{cat'}$), 6.29-6.23 (4H, m, $H_{cat,\ cat'}$), 5.60 (2H, s, $H_{5'}$), 5.38 (2H, s, H_5), 2.19 (3H, s, H_8), 2.13 (3H, s, $H_{8'}$)

^{13}C NMR (101 MHz, 298.0 K, CD_3CN): 157.0 (C_{cat}), 156.7 ($C_{cat'}$), 155.6 ($C_{cat,\ cat'}$), 150.4 ($C_{h'}$), 150.2 (C_h), 148.9 ($C_{f'}$), 146.9 (C_f), 129.9 (C_j), 129.1($C_{j'}$), 128.7 ($C_{b'/c'/d'/e'}$), 128.0 (C_b), 126.2 (C_e,

$_{b'/c'/d'/e'}$), 126.0 ($C_{b'/c'/d'/e'}$), 125.6 (C_d), 125.5 (C_c), 124.0 ($C_{b'/c'/d'/e'}$), 123.9 ($C_{k'}$), 122.2 (C_k), 118.4 (C_i), 118.0 ($C_{i'}$), 117.3 ($C_{cat\ cat'}$), 117.1 ($C_{cat\ cat'}$), 108.5 (C_{cat}), 108.3 ($C_{cat'}$), 107.4 ($C_{cat'}$), 107.2 (C_{cat}), 65.6 ($C_{g'}$), 61.6 (C_g), 20.6 ($C_{l,l'}$)

^{11}B NMR (128 MHz, 298.0 K, CD_3CN): δ 13.65 (bs)

The high-resolution mass spectral data reported below was obtained from an analogous reaction using the reductant potassium graphite instead of cobaltocene, as it was not possible to observe **2b** in the mass spectrum from the reduction with cobaltocene.

HRMS-NSI: *m/z* calcd for $C_{40}H_{33}{}^{10}B_2N_2O_4$ $[M-2K+H]^-$ 625.2705, found $[M-2K+H]^-$ 625.2708 (0.6 ppm)

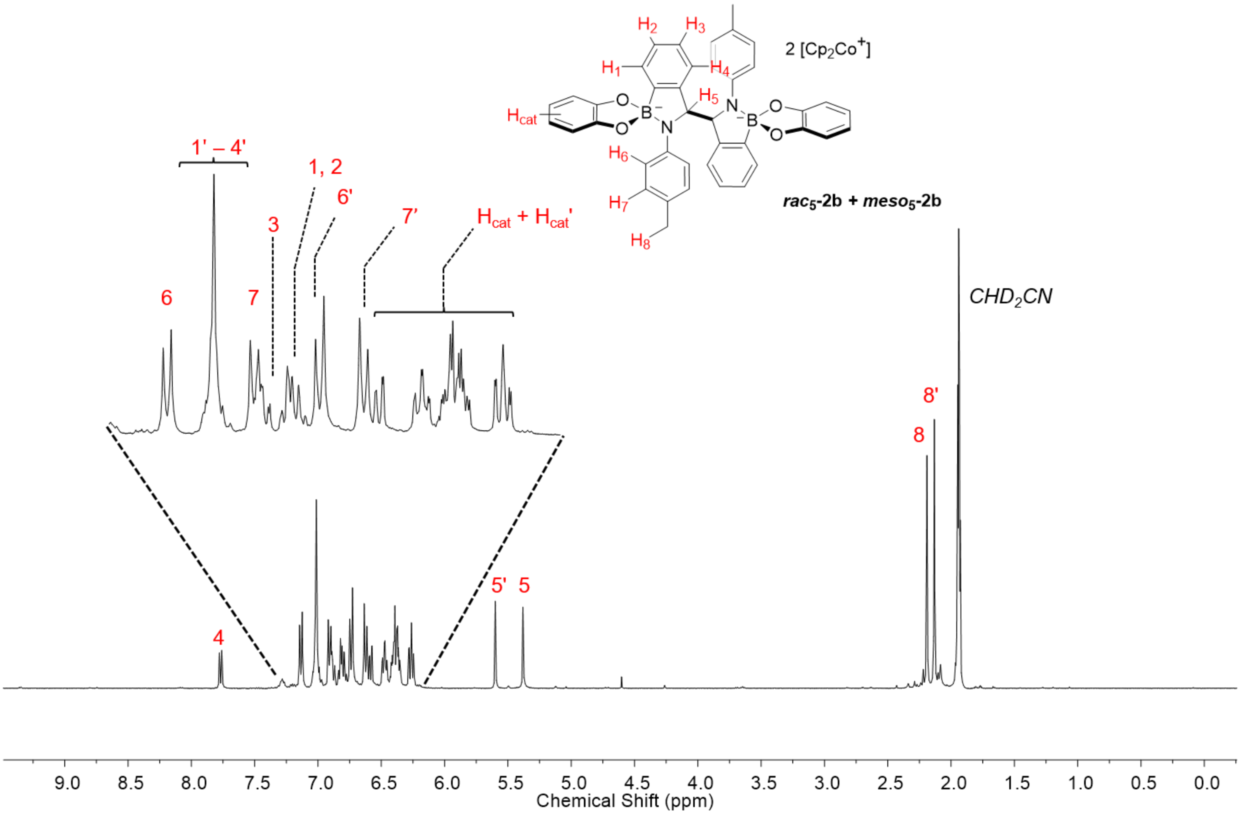

Figure S31. ^{1}H NMR spectrum (400 MHz, CD_3CN) of the reaction mixture from the reductive coupling of **1b**. Protons 1-8 correspond to ***rac*$_5$-2b** and protons 1'-8' to ***meso*$_5$-2b**.

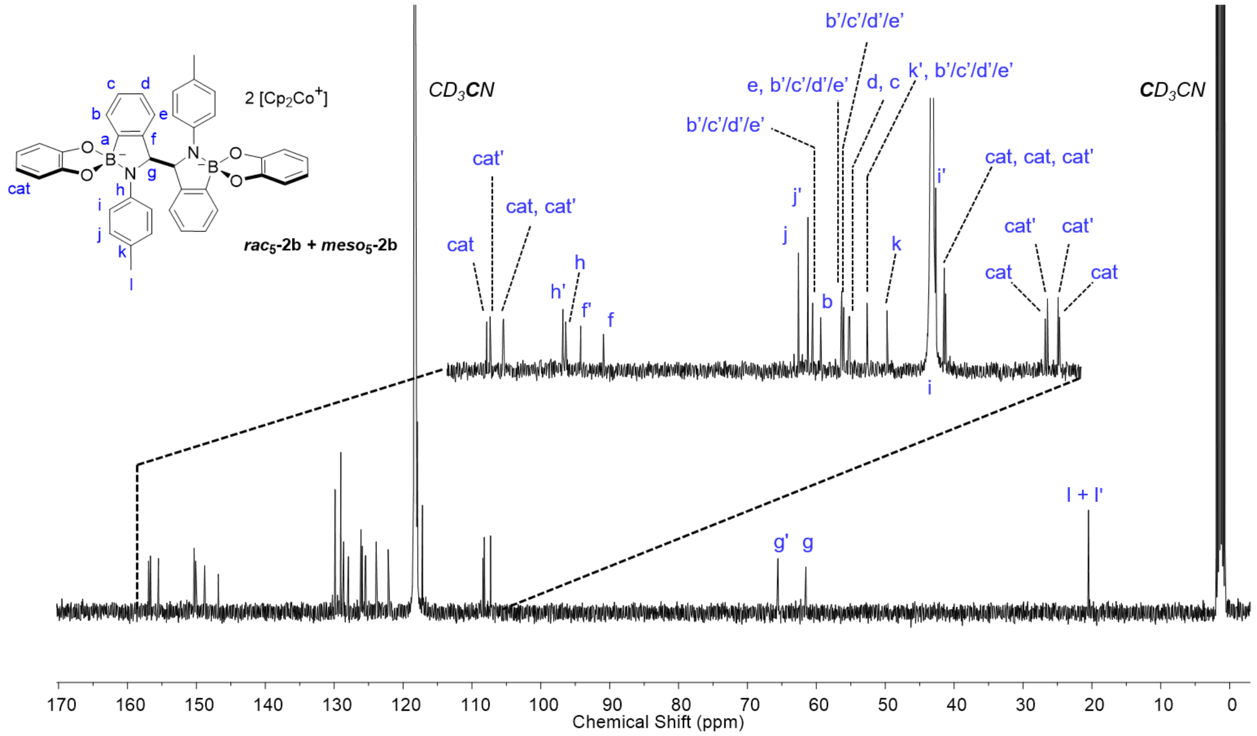

Figure S32. ^{13}C NMR spectrum (101 MHz, CD_3CN) of ***meso*₅-2b** and ***rac*₅-2b** after partial interconversion. Carbons without prime labels correspond to ***rac*₅-2b** and carbons with prime labels correspond to ***meso*₅-2b**.

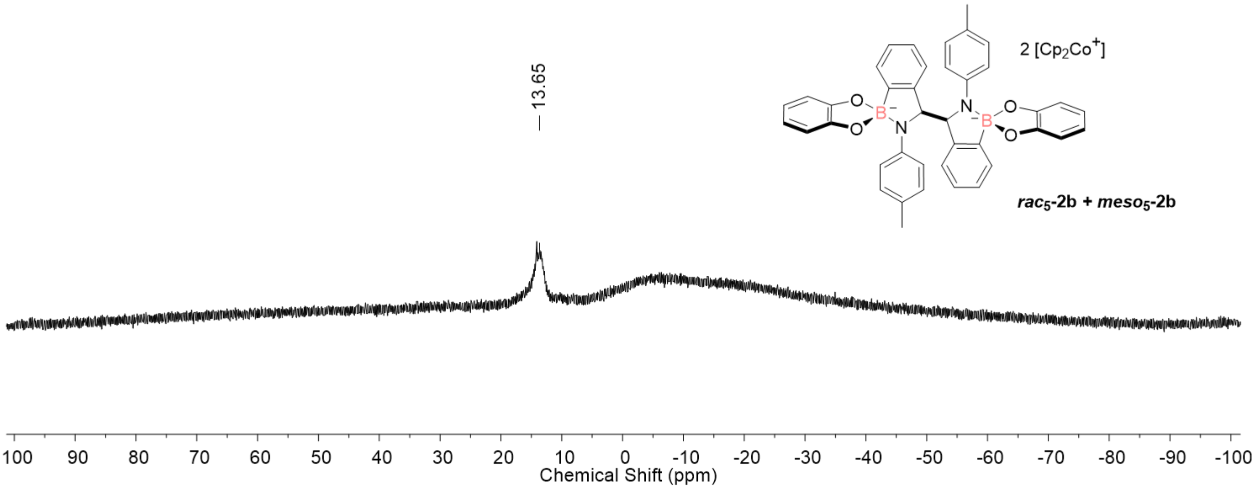

Figure S33. ^{11}B NMR spectrum (128 MHz, CD_3CN) of the reaction mixture from the reductive coupling of **1b**.

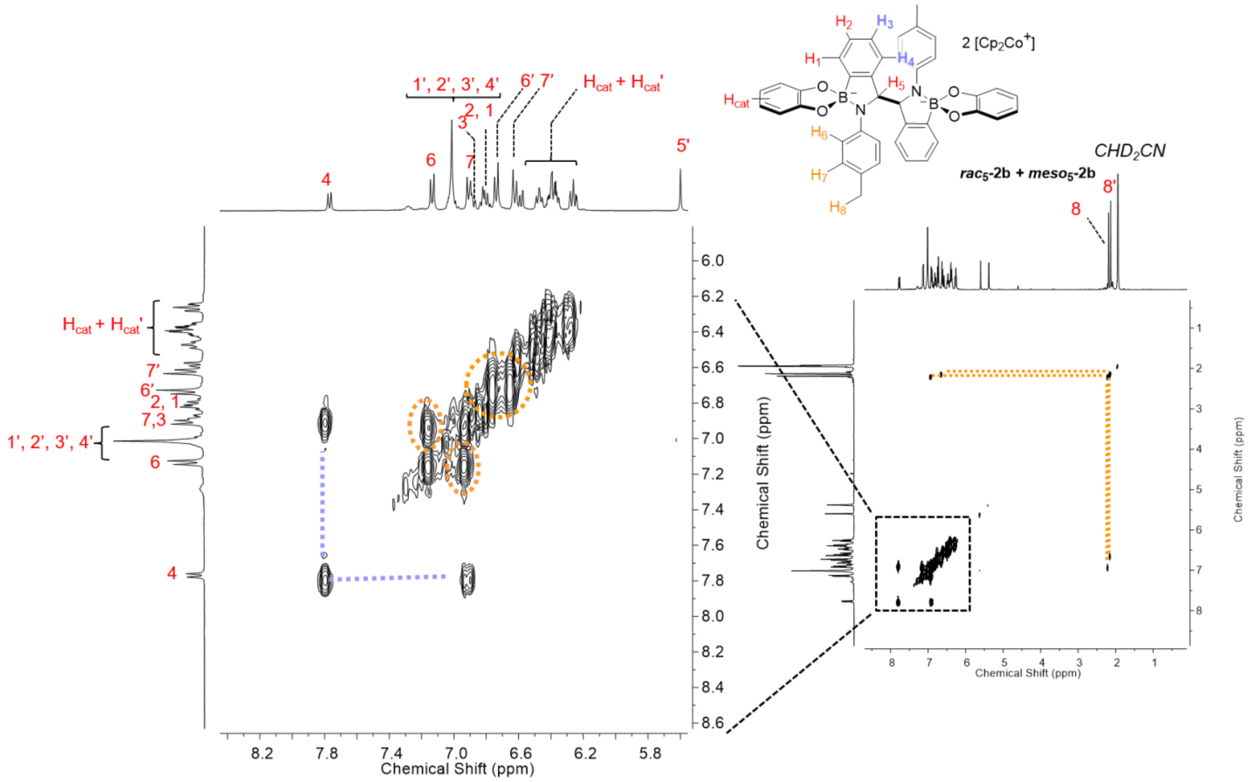

Figure S34. COSY NMR spectrum (400 MHz, CD_3CN) of the reaction mixture from the reductive coupling of **1b**. Protons 1-8 correspond to ***rac*$_5$-2b** and protons 1'-8' to ***meso*$_5$-2b**.

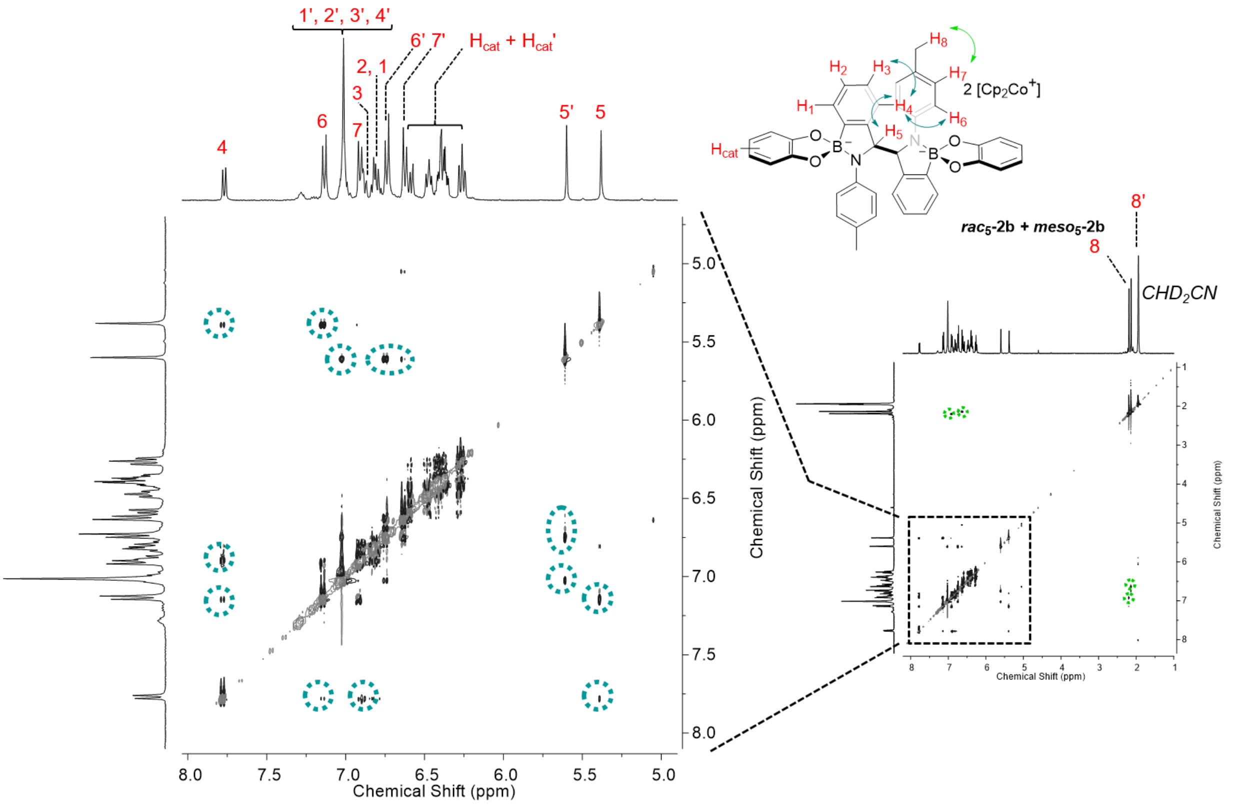

Figure S35. NOESY NMR spectrum (400 MHz, CD_3CN) of the reaction mixture from the reductive coupling of **1b**. Protons 1-8 correspond to ***rac*$_5$-2b** and protons 1'-8' to ***meso*$_5$-2b**.

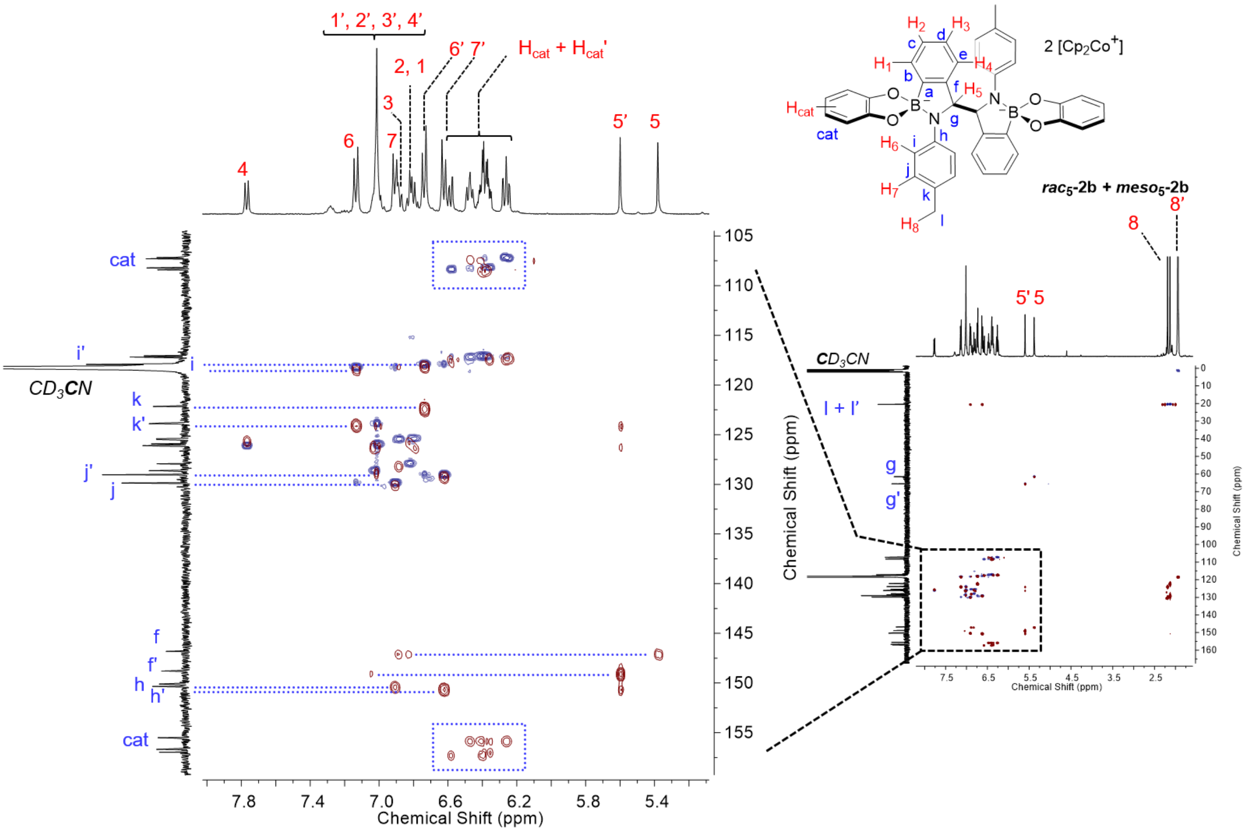

Figure S36. Overlay of HSQC (blue) and HMBC (red) NMR spectra of the reaction mixture from the reductive coupling of **1b**. Protons/carbons without prime labels correspond to ***rac*₅-2b** and protons/carbons with prime labels correspond to ***meso*₅-2b**.

4.2 *rac*$_5$-2b, *rac*$_6$-2b and *meso*$_5$-2b Mixture in DMSO-d_6

In a nitrogen atmosphere glove box, Cp_2Co (3.02 mg, 0.016 mmol) was dissolved in 0.5 mL of DMSO-d_6. This solution was then agitated with **1b** (4.97 mg, 0.016 mmol) until the solid was fully dissolved and transferred to a J. Young NMR tube. Reaction progress was monitored by NMR spectroscopy.

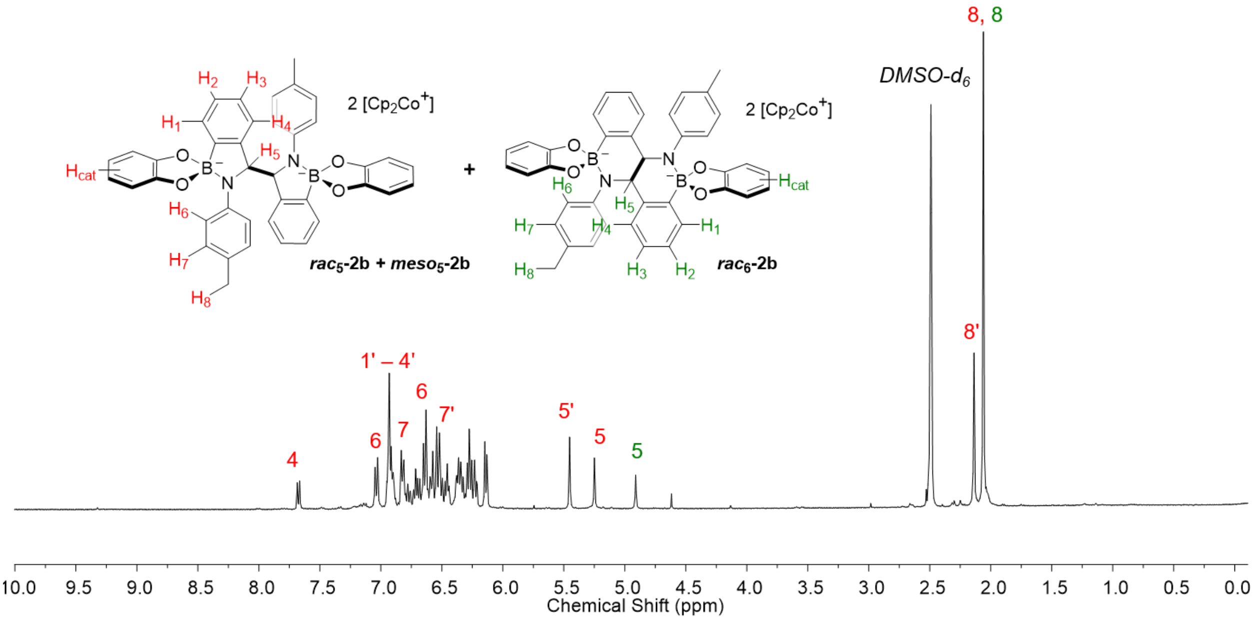

Figure S37. ^{1}H NMR spectrum of the reductive coupling of **1b** in DMSO-d_6 showing the formation of the ***rac*$_5$-2b**, ***rac*$_6$-2b** and ***meso*$_5$-2b** products. As a complex mixture of three products, full assignment was not carried out but key protons for ***rac*$_5$-2b** (red), ***meso*$_5$-2b** (red') and ***rac*$_6$-2b** (green) were assigned.

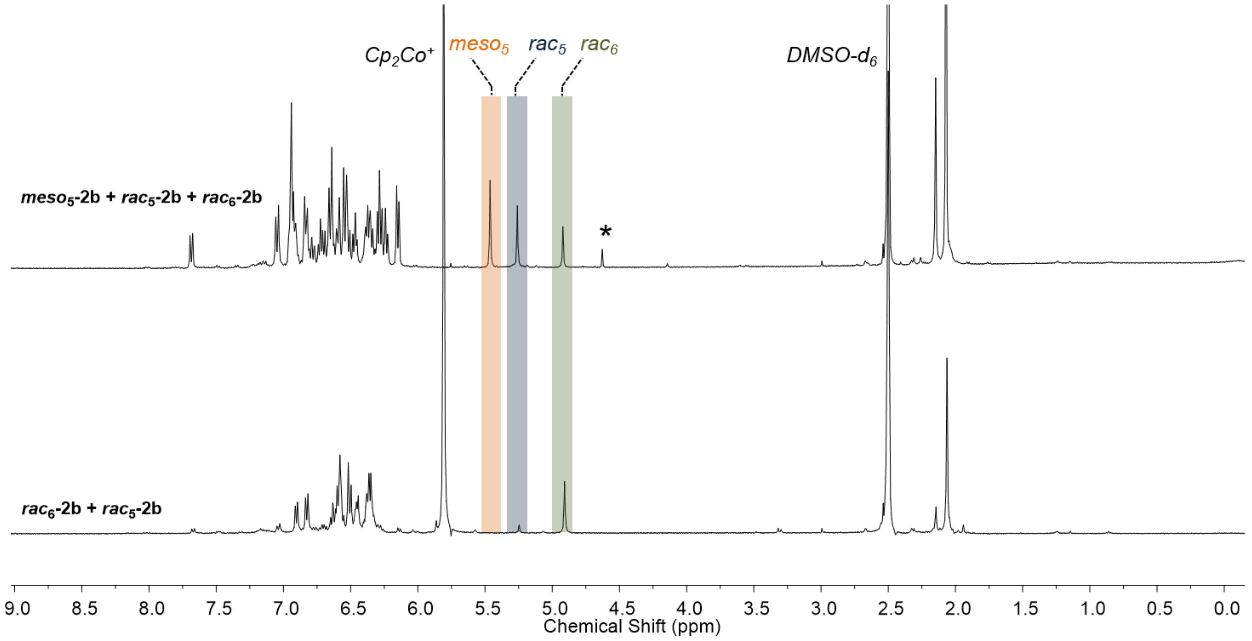

Figure S38. Stacked ^{1}H NMR spectra (400 MHz, DMSO-d_6) of: the **2b** mixture formed in DMSO-d_6 (top) and the mixture of ***rac*$_6$-2b** and ***rac*$_5$-2b** after partial interconversion (bottom). * is attributed to a transient Cp_2Co^+ species.

4.3 ***rac*$_6$-2b**

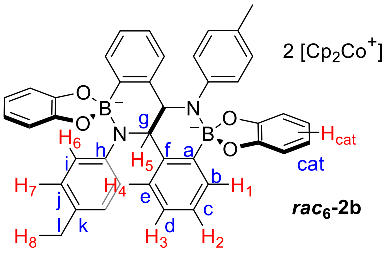

In a nitrogen atmosphere glove box, Cp_2Co (3.01 mg, 0.016 mmol) was dissolved in 0.5 mL of CD_3CN. This solution was then agitated with **1b** (4.98 mg, 0.016 mmol) until the solid was fully dissolved and transferred to a J. Young NMR tube. Crystals grown from the unperturbed reaction mixture were isolated by decanting the solution. They were washed twice with CH_3CN (ca. 1 mL) and redissolved in 0.5 mL DMSO-d_6.

While the ^1H NMR spectrum of the redissolved ***rac*$_6$-2b** crystals showed one species initially (Figure S39), a new set of signals grew over time corresponding to the ***rac*$_5$-2b** isomer (see Section 4.4, Figure S44). Due to this interconversion to the ***rac*$_5$-2b** isomer, it was not possible to record ^{13}C, ^{19}F and 2D NMR spectra of the ***rac*$_6$-2b** isomer alone. The ^{13}C chemical shifts reported below were assigned from this mixture.

^1H NMR (400 MHz, 298.0 K, DMSO-d_6): δ 6.90 (2H, d, ^3J = 6.6 Hz, H_1), 6.83 (2H, d, ^3J = 7.3 Hz, H_4), 6.63 (2H, td, ^3J = 7.3 Hz, ^4J = 1.2 Hz, H_3), 6.62-6.54 (6H, m, $H_{7,2}$), 6.51 (4H, d, ^3J = 8.6 Hz, H_6), 6.48-6.42 (2H, m, H_{cat}), 6.42-6.31 (6H, m, H_{cat}), 4.90 (2H, s, H_5), 2.07 (3H, s, H_8)

^{13}C NMR (126 MHz, 298.0 K, DMSO-d_6): δ 156.3 (C_{cat}), 154.0 (C_{cat}), 150.8 (C_h), 147.7 (b, C_a), 143.2 (C_f), 128.8 (C_b), 128.0 (C_j), 124.5 (C_e), 124.0 (C_d), 123.4 (C_c), 120.5 (C_k), 116.2 (C_{cat}), 116.0 (C_{cat}), 115.3 (C_i), 107.8 (C_{cat}), 106.4 (C_{cat}), 63.8 (C_g), 20.0 (C_l)

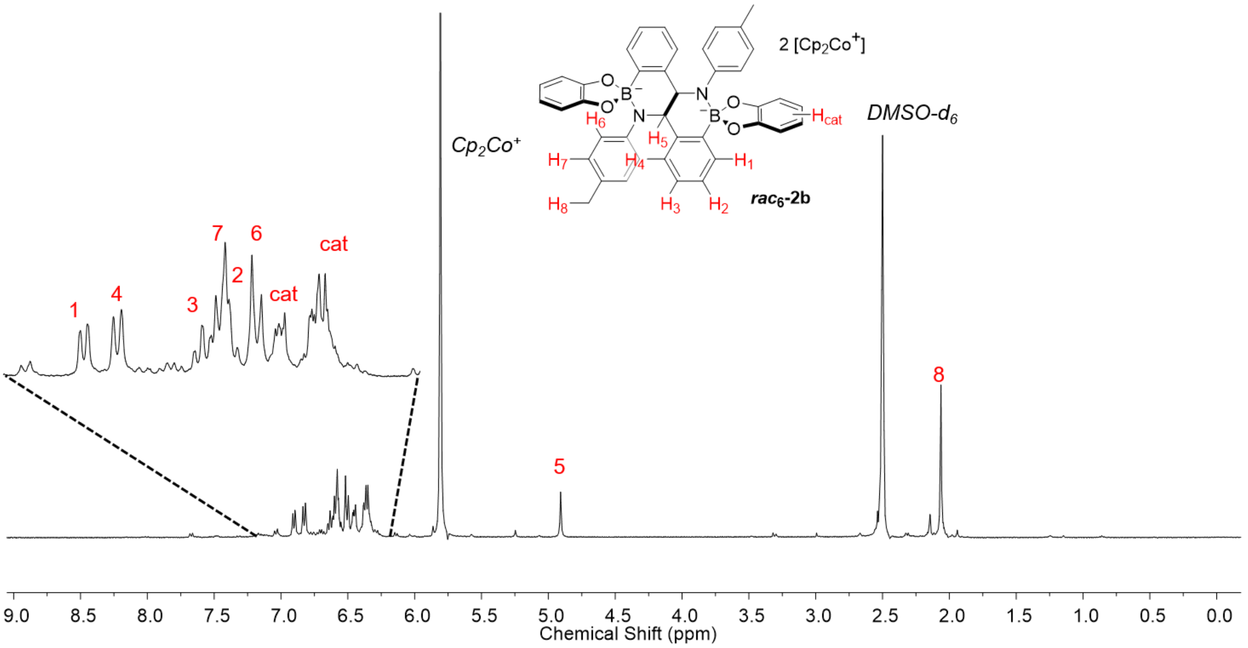

Figure S39. ^1H NMR spectrum (400 MHz, DMSO-d_6) of ***rac*$_6$-2b** crystals.

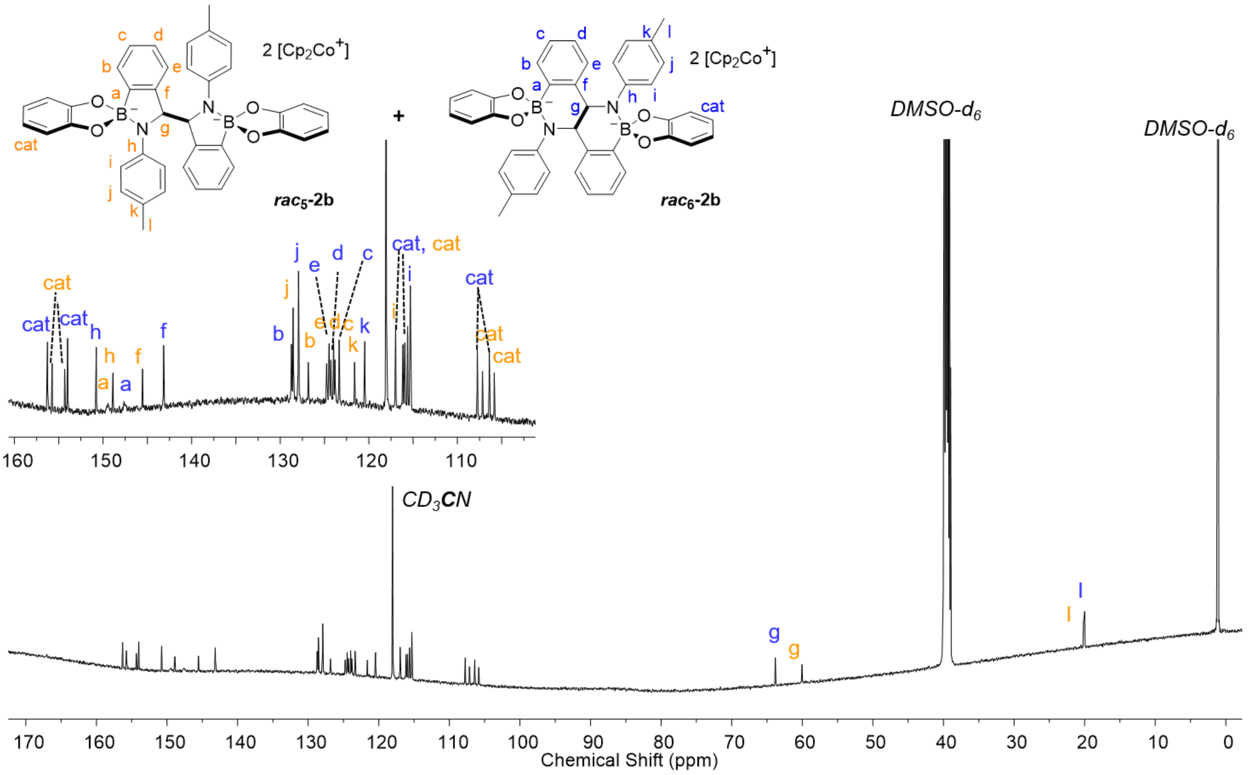

Figure S40. ^{13}C NMR spectrum (126 MHz, DMSO-d_6) of ***rac*$_6$-2b** and ***rac*$_5$-2b** after partial interconversion.

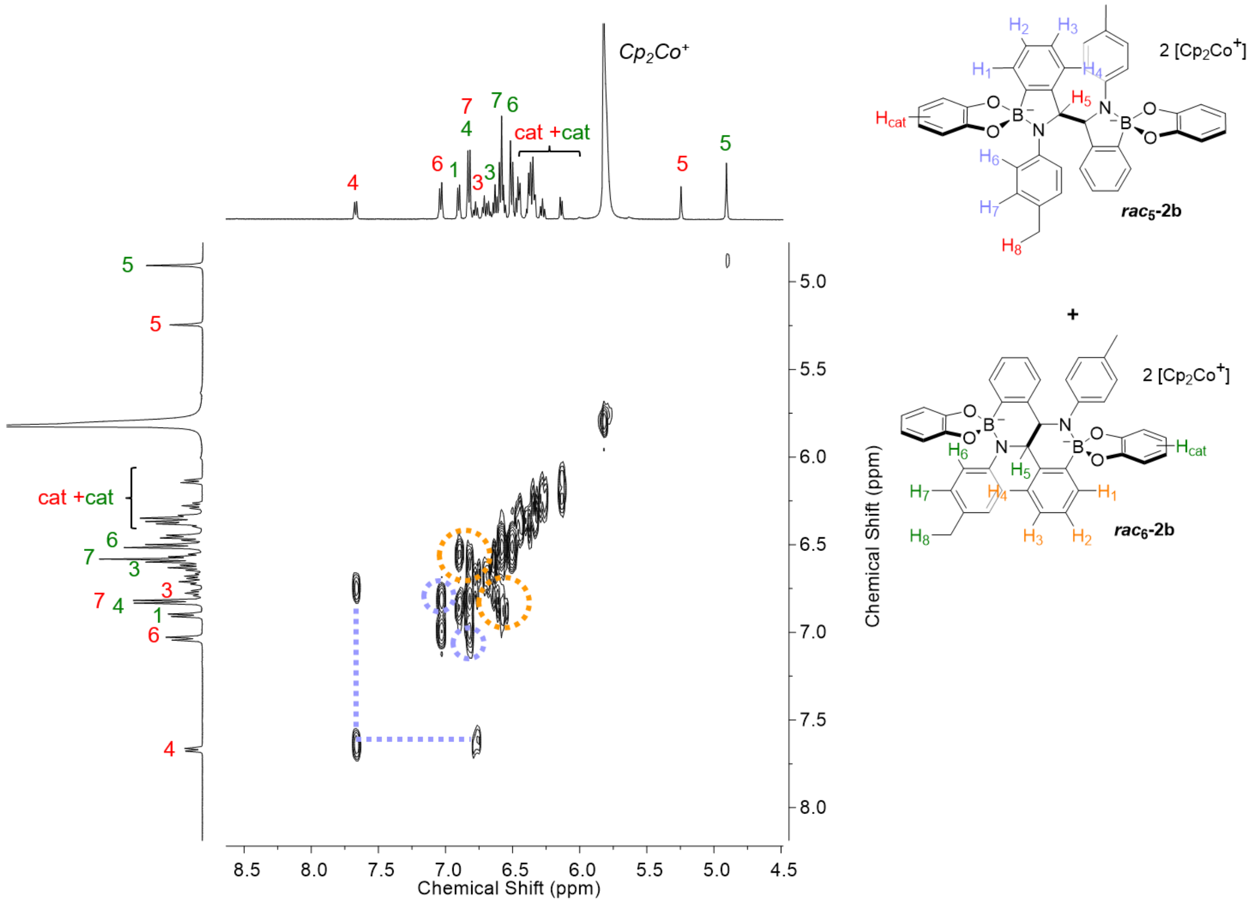

Figure S41. COSY NMR spectrum (500 MHz, DMSO-d_6) of ***rac*$_6$-2b** and ***rac*$_5$-2b** after partial interconversion.

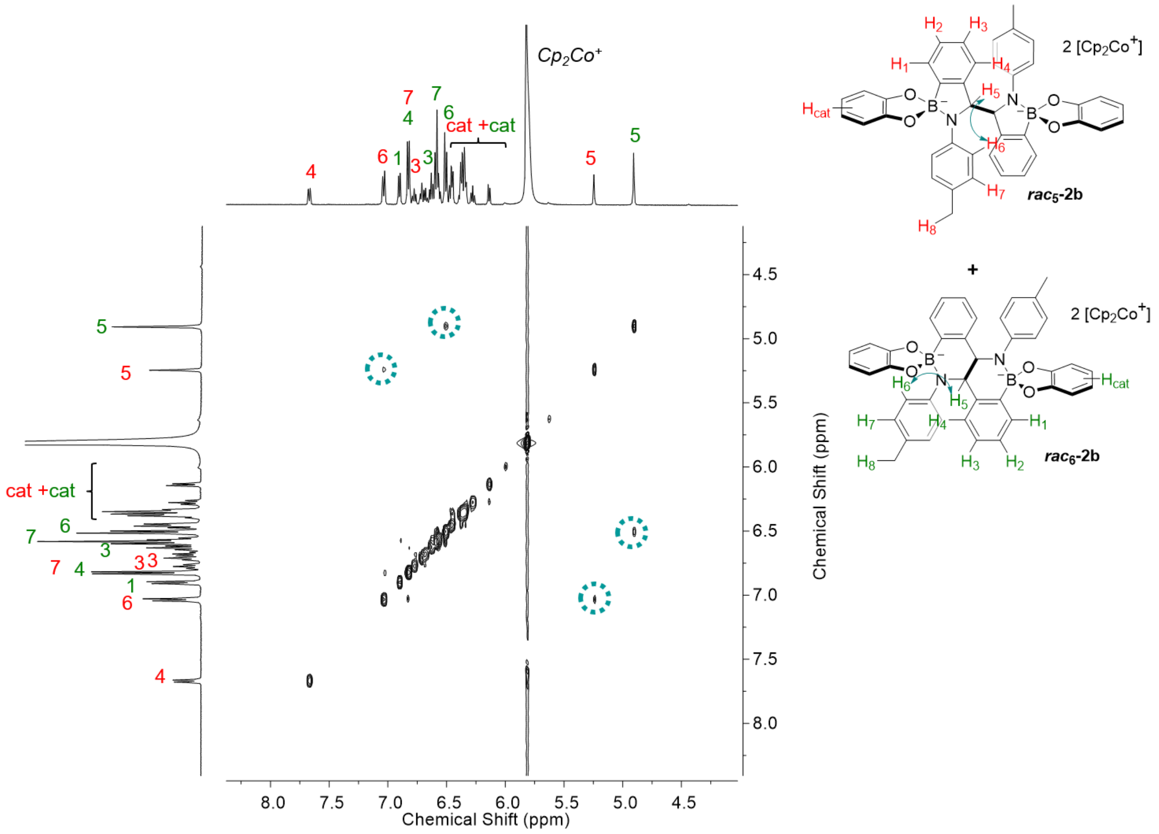

Figure S42. NOESY NMR spectrum (500 MHz, DMSO-d_6) of ***rac*$_6$-2b** and ***rac*$_5$-2b** after partial interconversion.

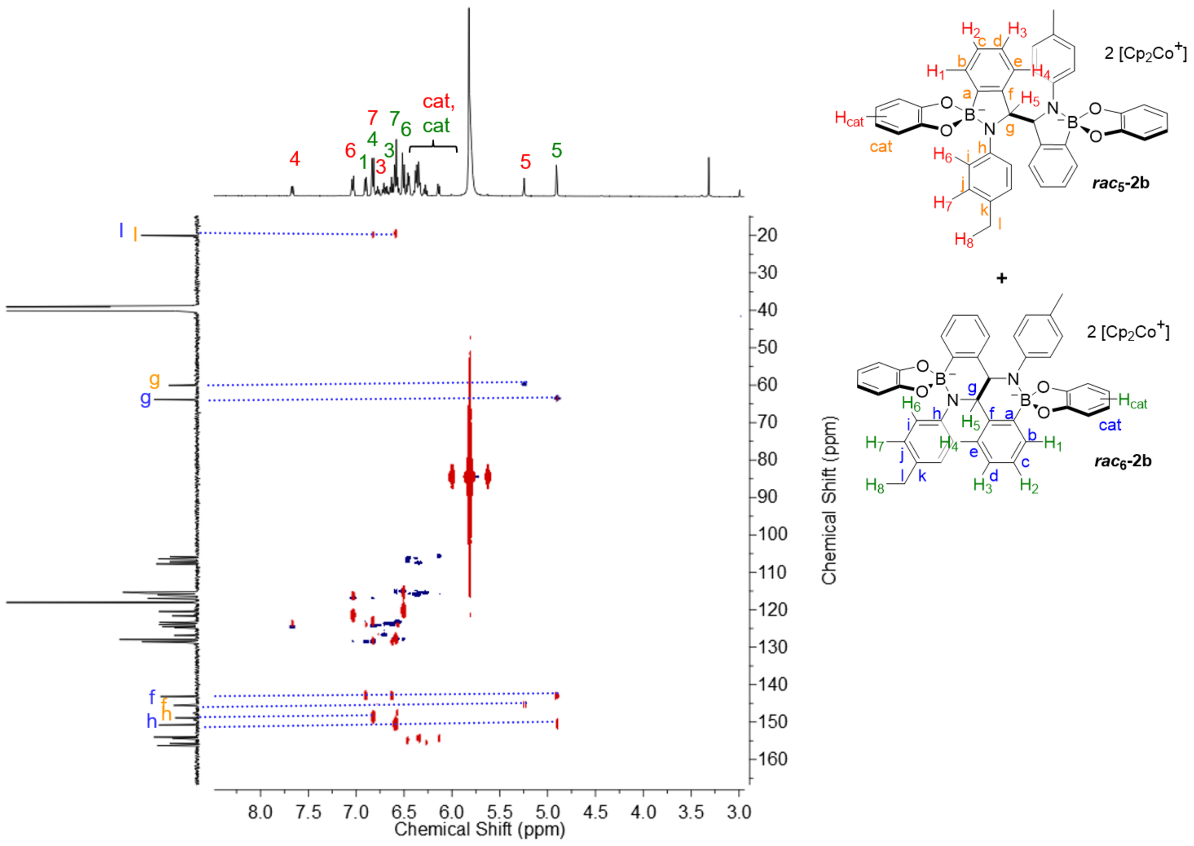

Figure S43. Overlay of HSQC (blue) and HMBC (red) NMR spectra (DMSO-d_6) of ***rac*$_6$-2b** and ***rac*$_5$-2b** after partial interconversion.

4.4 *rac*₅-2b

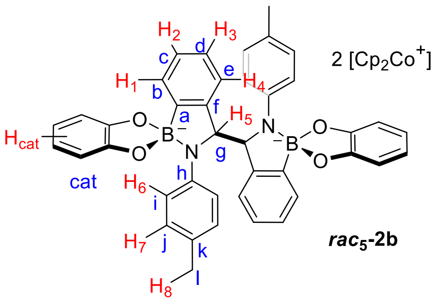

Characterisation data for the ***rac*$_5$-2b** isomer could be inferred from the NMR data of the equilibrated ***rac*$_5$-2b** and ***rac*$_6$-2b** mixture from Section 4.3. Due to the number of overlapping signals in the ^{1}H NMR spectrum of the mixture, the ^{1}H NMR chemical shifts are not reported below but Figure S44 shows the assignments. The ^{13}C NMR chemical shifts reported below were assigned from the ^{13}C NMR and HSQC/HMBC spectra (Figures S40, S43)

^{13}C NMR (126 MHz, 298.0 K, DMSO-*d*$_6$): 155.8 (C_{cat}), 154.4 (C_{cat}), 149.5 (b, C_a), 148.9 (C_h), 145.6 (C_f), 128.6 (C_j), 126.9 (C_b), 124.8 (C_e), 124.3 (C_d), 123.8 (C_c), 121.6 (C_k), 117.0 (C_i), 115.6 (C_{cat}), 107.2 (C_{cat}), 105.9 (C_{cat}), 60.1 (C_g), 20.2 (C_l)

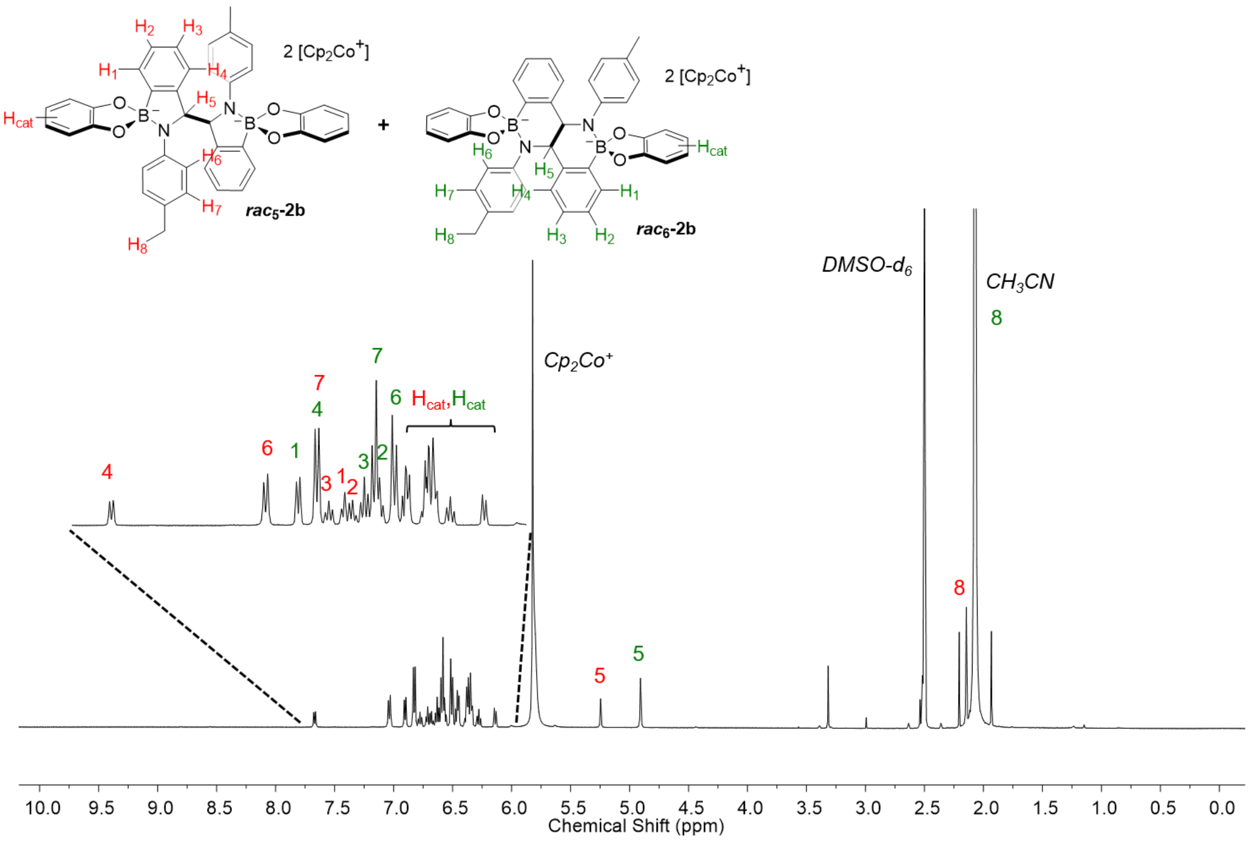

Figure S44. ^{1}H NMR spectrum (500 MHz, DMSO-*d*$_6$) of ***rac*$_6$-2b and *rac*$_5$-2b** after partial interconversion.

5 Solution Characterisation of the *tert*-Butylaniline-Pyrocatechol Reductively Coupled Dimer (2c)

5.1 *rac*₅-2c and *meso*₅-2c Mixture in CD_3CN

In a nitrogen atmosphere glove box, Cp_2Co (3.00 mg, 0.016 mmol) was dissolved in 0.5 mL of CD_3CN. This solution was then agitated with **1c** (6.5 mg, 0.016 mmol) until the solid was fully dissolved and transferred to a J. Young NMR tube.

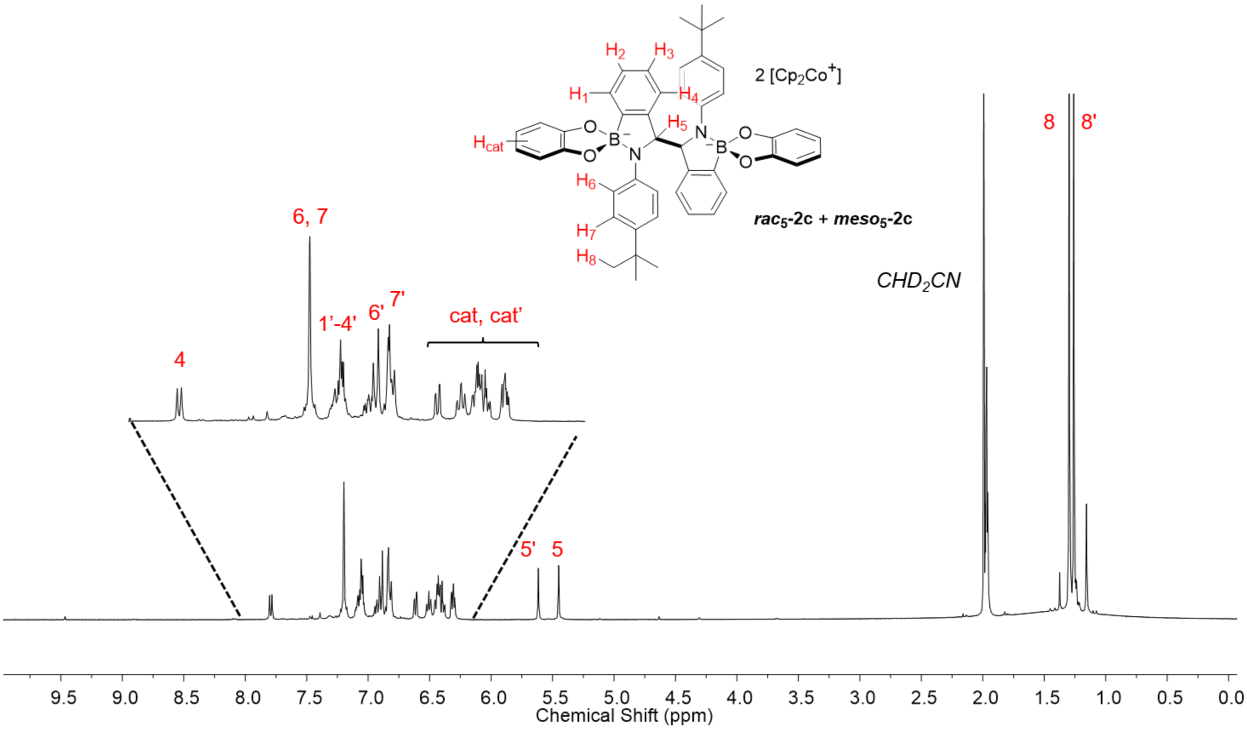

Figure S45. ^{1}H NMR spectrum (400 MHz, CD_3CN) of the reaction mixture from the reductive coupling of **1c**. Protons 1-8 correspond to ***rac*$_5$-2c** and protons 1'-8' to ***meso*$_5$-2c**.

5.2 *meso*$_5$-2c

After standing unperturbed at room temperature for 7 days, the **2c** mixture (Section 5.1) contained only the *meso*$_5$ isomer as crystallization of ***rac*$_6$-2c** removed nearly all of the *rac* species (***rac*$_5$-2c** and ***rac*$_6$-2c**) from solution. Due to the small quantity of ***meso*$_5$-2c** in solution and overlapping signals for protons 1-4, unambiguous assignment of carbons *b*-*f* and *h* by 2D techniques (HSQC and HMBC, Figure S49) was not possible (Figure S47).

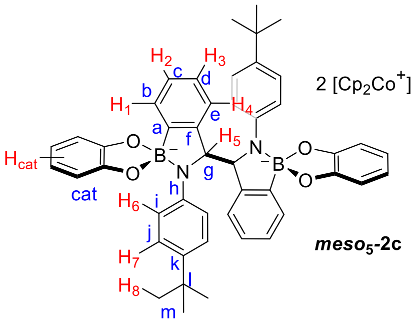

^{1}H NMR (400 MHz, 298.0 K, CD_3CN): δ 7.09-7.00 (8H, m, $H_{1\text{-}4}$), 6.86 (4H, d, ^{3}J = 8.9 Hz, H_7), 6.79 (4H, d, ^{3}J = 8.9 Hz, H_6), 6.50-6.34 (6H, m, H_{cat}), 6.29-6.23 (2H, m, H_{cat}), 5.58 (2H, s, H_5), 1.23 (18H, s, H_8)

^{13}C NMR (126 MHz, 298.0 K, CD_3CN): δ 156.8 (C_{cat}), 155.7 (C_{cat}), 150.2 ($C_{h/f}$), 148.9 ($C_{h/f}$), 135.9 (C_k), 128.7 ($C_{b/c/d/e}$), 126.2 ($C_{b/c/d/e}$), 126.0 ($C_{b/c/d/e}$), 125.3 (C_j), 124.0 ($C_{b/c/d/e}$), 117.6 (C_i), 117.2 (C_{cat}), 117.2 (C_{cat}), 108.3 (C_{cat}), 107.4 (C_{cat}), 66.0 (C_g), 34.2 (C_l), 32.1 (C_m)

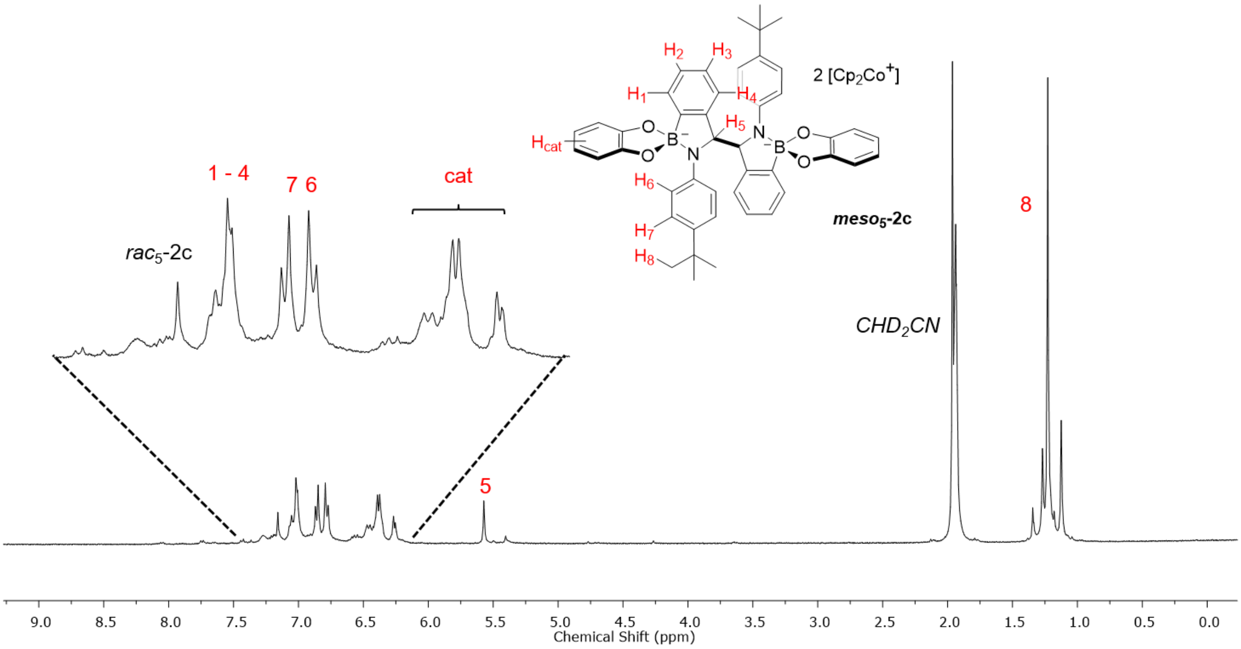

Figure S46. ^{1}H NMR spectrum (400 MHz, CD_3CN) of ***meso*₅-2c** left in solution following crystallisation of ***rac*₆-2c**.

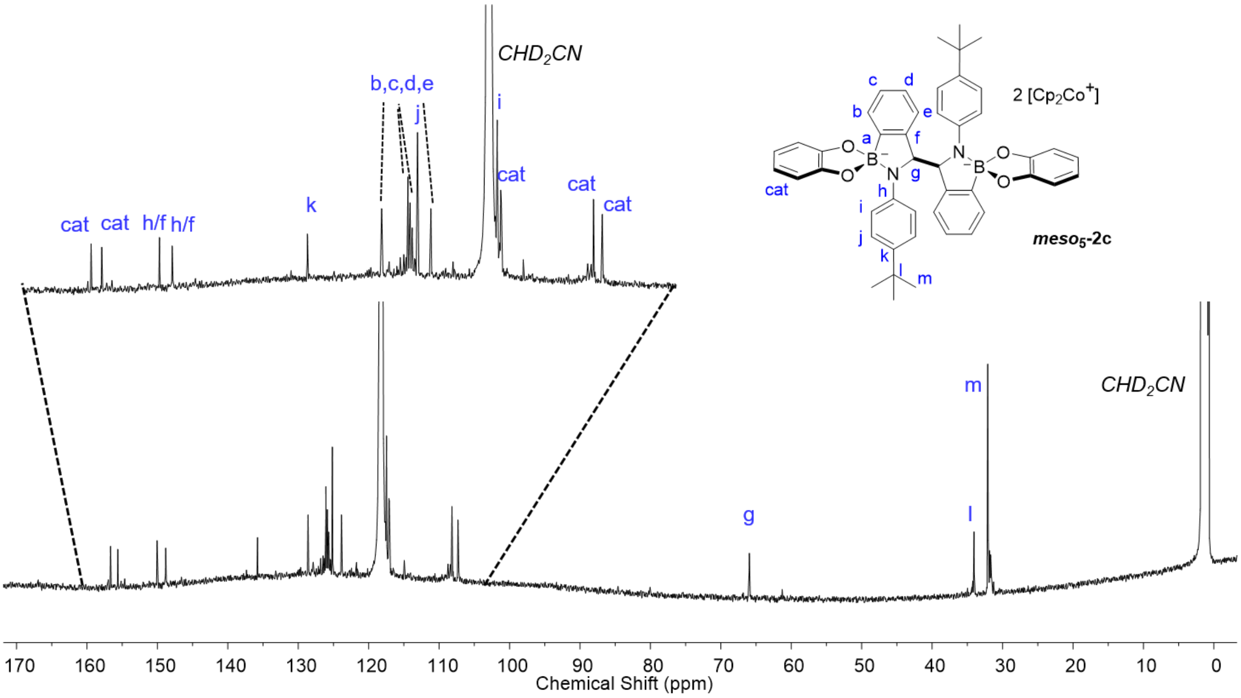

Figure S47. ^{13}C NMR spectrum (126 MHz, CD_3CN) of ***meso*₅-2c** left in solution following crystallisation of the ***rac*₆** isomeromer. Unambiguous assignment of carbons *b*-*f* and *h* by 2D techniques (HSQC and HMBC) was not possible due to the quantity of sample in solution and the overlapping proton signals.

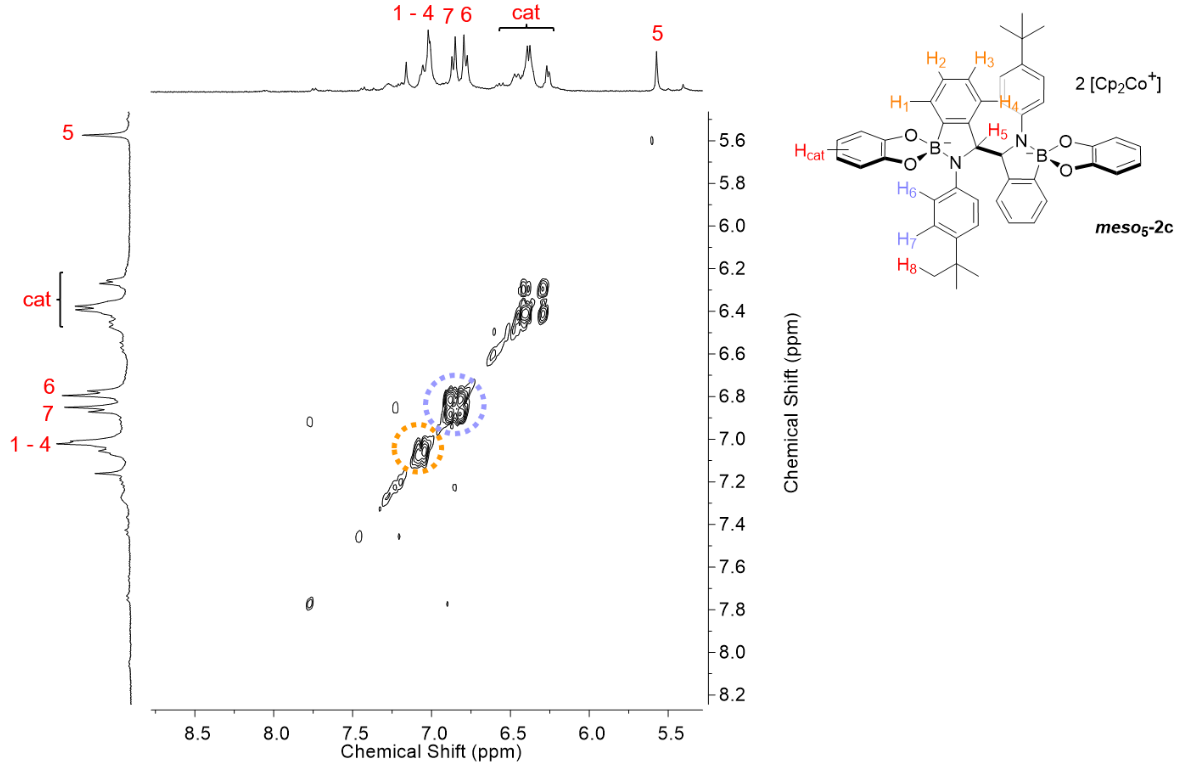

Figure S48. COSY NMR spectrum (400 MHz, CD_3CN) of ***meso*₅-2c** left in solution following crystallisation of the ***rac*₆** diastereomer.

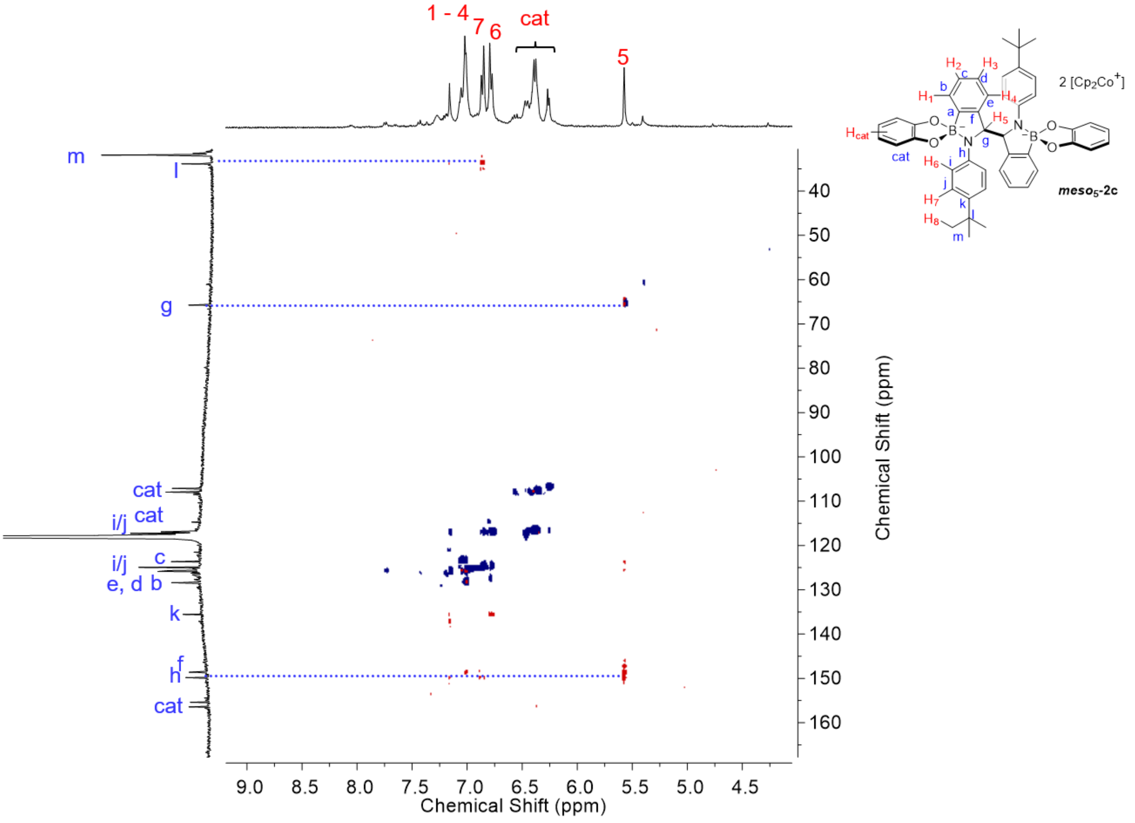

Figure S49. Overlay of the HSQC (blue) and HMBC (red) NMR spectra of ***meso*₅-2c** left in solution following crystallisation of the ***rac*₆** isomer.

5.3 *rac*$_6$-2c

Crystals grown from the unperturbed reaction mixture in Section 5.1 were isolated by decanting the solution. They were washed twice with CH_3CN (ca. 1 mL) and redissolved in 0.5 mL DMSO-d_6.

The ^{1}H and COSY NMR spectra of the redissolved crystals were recorded before interconversion to ***rac*$_5$-2c** (Figures S51, S52), but partial interconversion had occurred during the recording of the ^{13}C, NOESY, HSQC and HMBC spectra (Figures S53-S54). The ^{13}C chemical shifts reported below were assigned from this mixture.

^{1}H NMR (400 MHz, 298.0 K, DMSO-d_6): δ 6.92 (2H, d, ^3J = 7.3 Hz, H_1), 6.84 (2H, d, ^3J = 7.3 Hz, H_4), 6.79 (4H, d, ^3J = 8.9 Hz, H_7), 6.64 (2H, t, ^3J = 7.3 Hz, H_3), 6.58 (2H, t, ^3J = 7.3 Hz, H_2), 6.53 (4H, d, ^3J = 8.9 Hz, H_6), 6.49-6.43 (2H, m, H_{cat}), 6.43-6.32 (6H, m, H_{cat}), 4.93 (2H, s, H_5), 1.16 (18H, s, H_8)

^{13}C NMR (126 MHz, 298.0 K, DMSO-d_6): δ 156.2 (C_{cat}), 154.1 (C_{cat}), 150.6 (C_h), 147.6 (b, C_a), 143.2 (C_f), 134.4 (C_k), 128.8 (C_b), 124.3 (C_e), 124.0 ($C_{d,j}$), 123.4 (C_c), 116.2 (C_{cat}), 115.9 (C_{cat}), 114.8 (C_i), 107.8 (C_{cat}), 106.5 (C_{cat}), 84.7 (Cp_2Co^+), 63.7 (C_g), 33.1 (C_l), 31.6 (C_m)

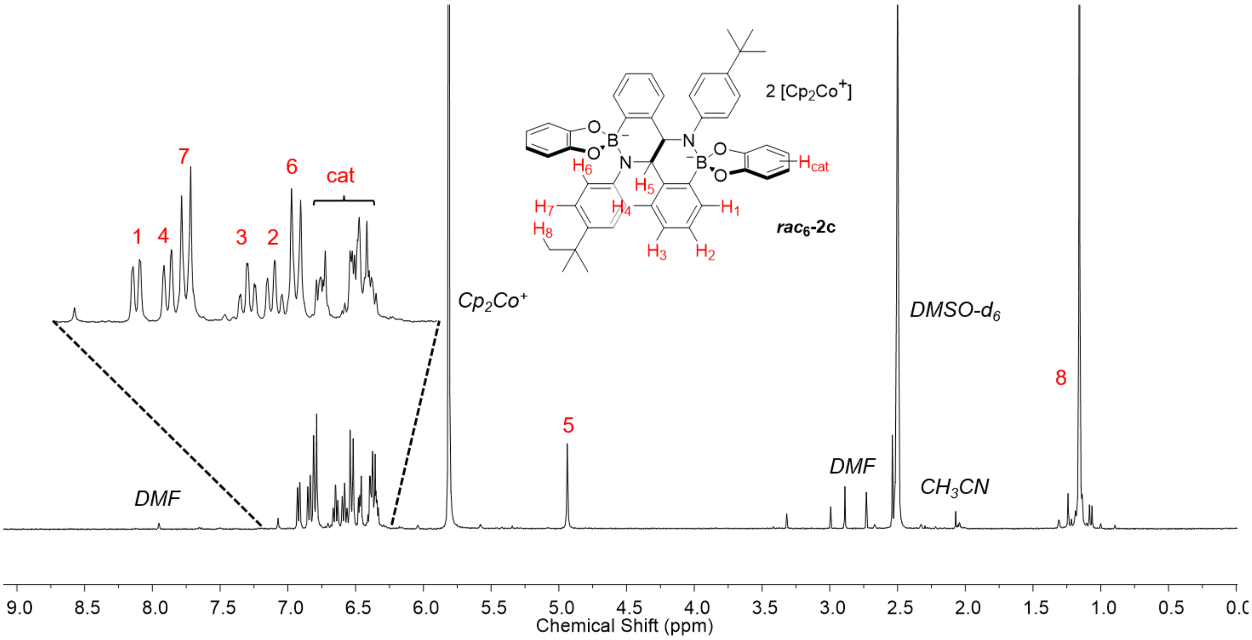

Figure 50. ^{1}H NMR spectrum (400 MHz, DMSO-d_6) of ***rac*$_6$-2c** crystals.

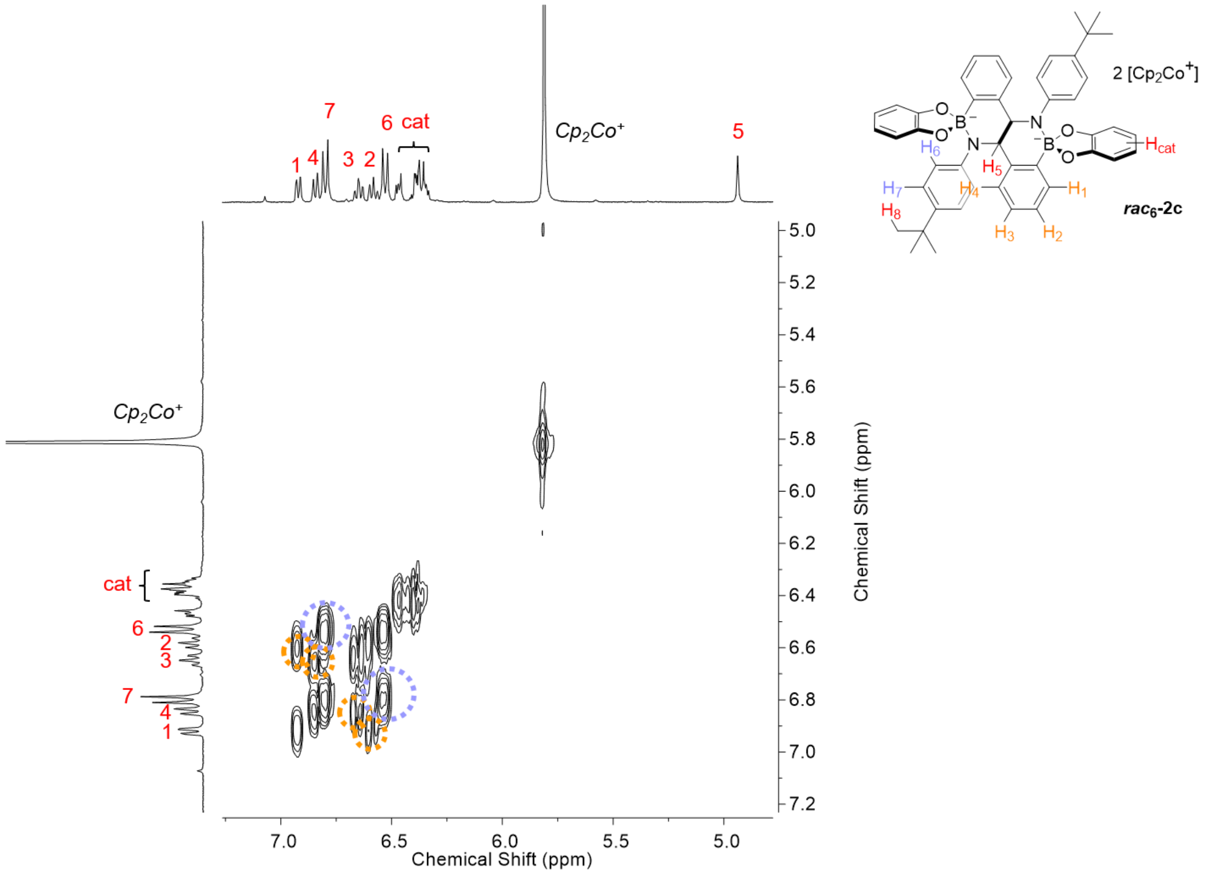

Figure 51. COSY NMR spectrum (400 MHz, DMSO-d_6) of ***rac*$_6$-2c** crystals.

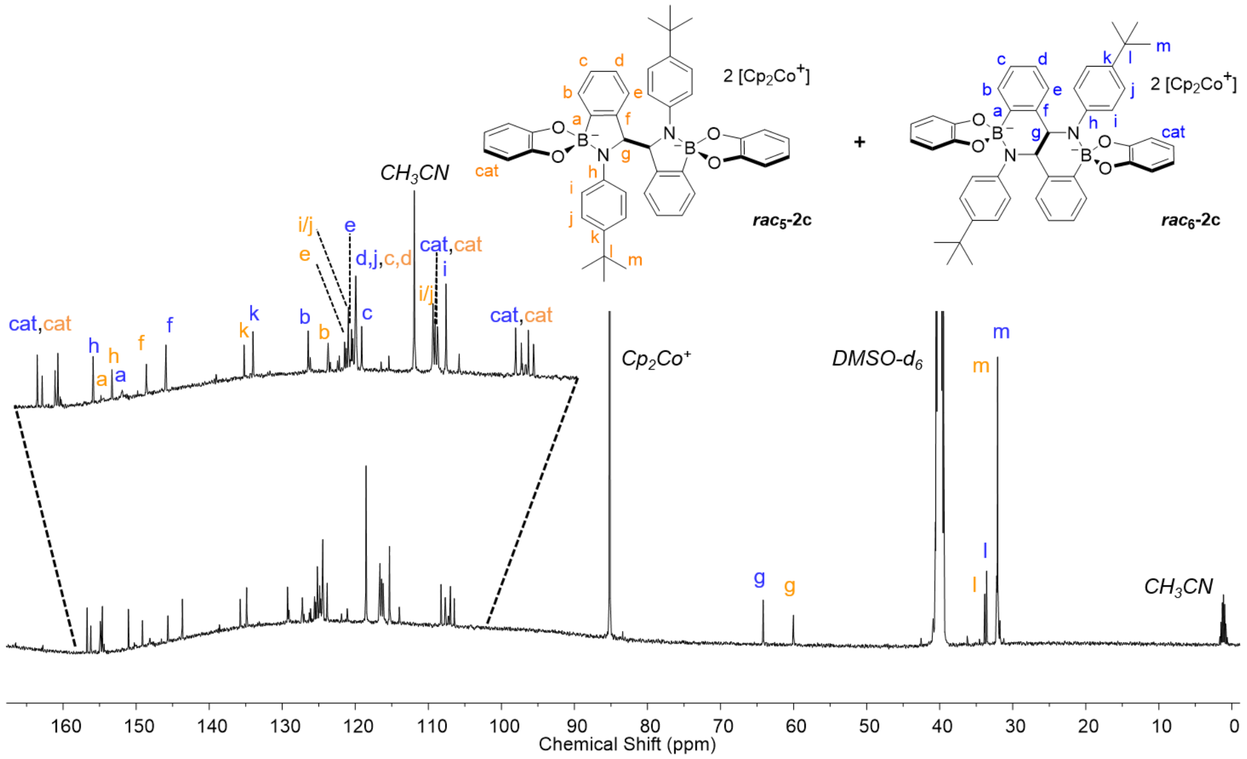

Figure 52. ^{13}C NMR spectrum (126 MHz, DMSO-d_6) of ***rac*$_6$-2c** and ***rac*$_5$-2c** after partial interconversion.

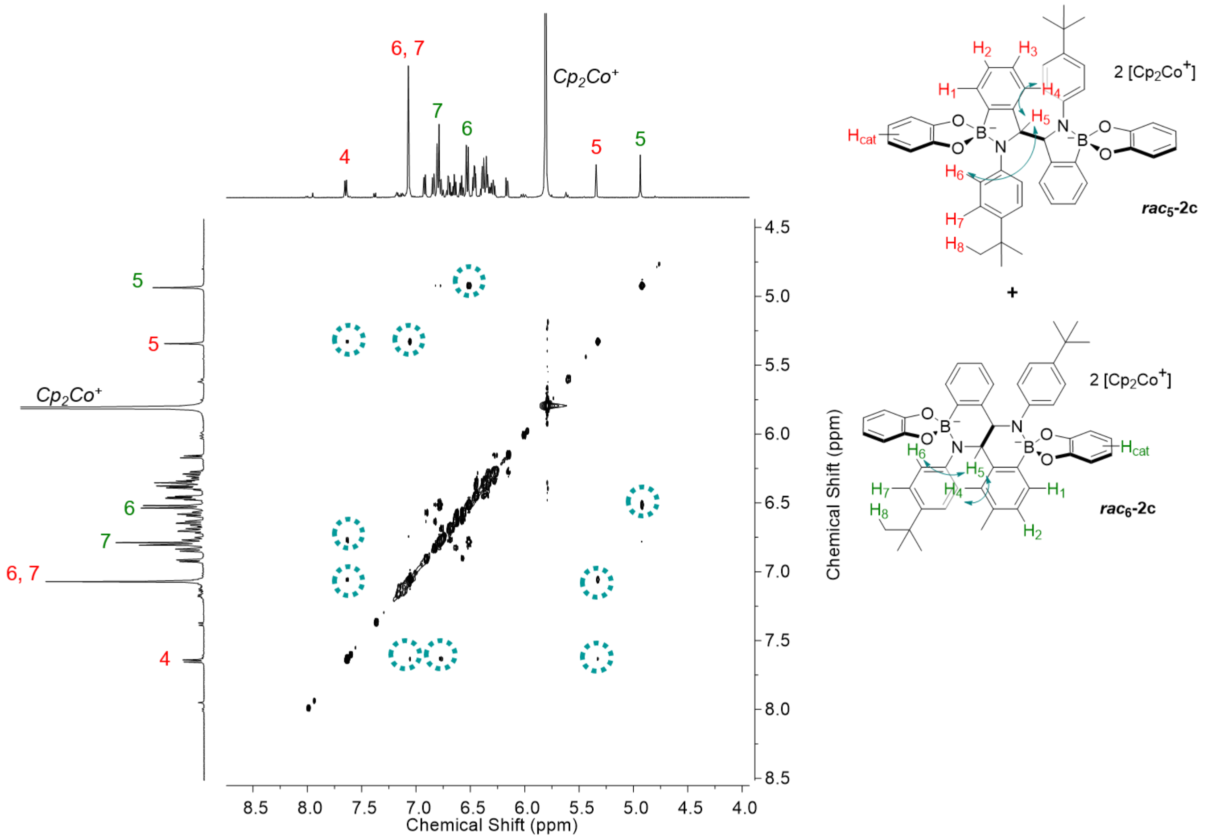

Figure 53. NOESY NMR spectrum (500 MHz, DMSO-d_6) of ***rac*₆-2c** and ***rac*₅-2c** after partial interconversion.

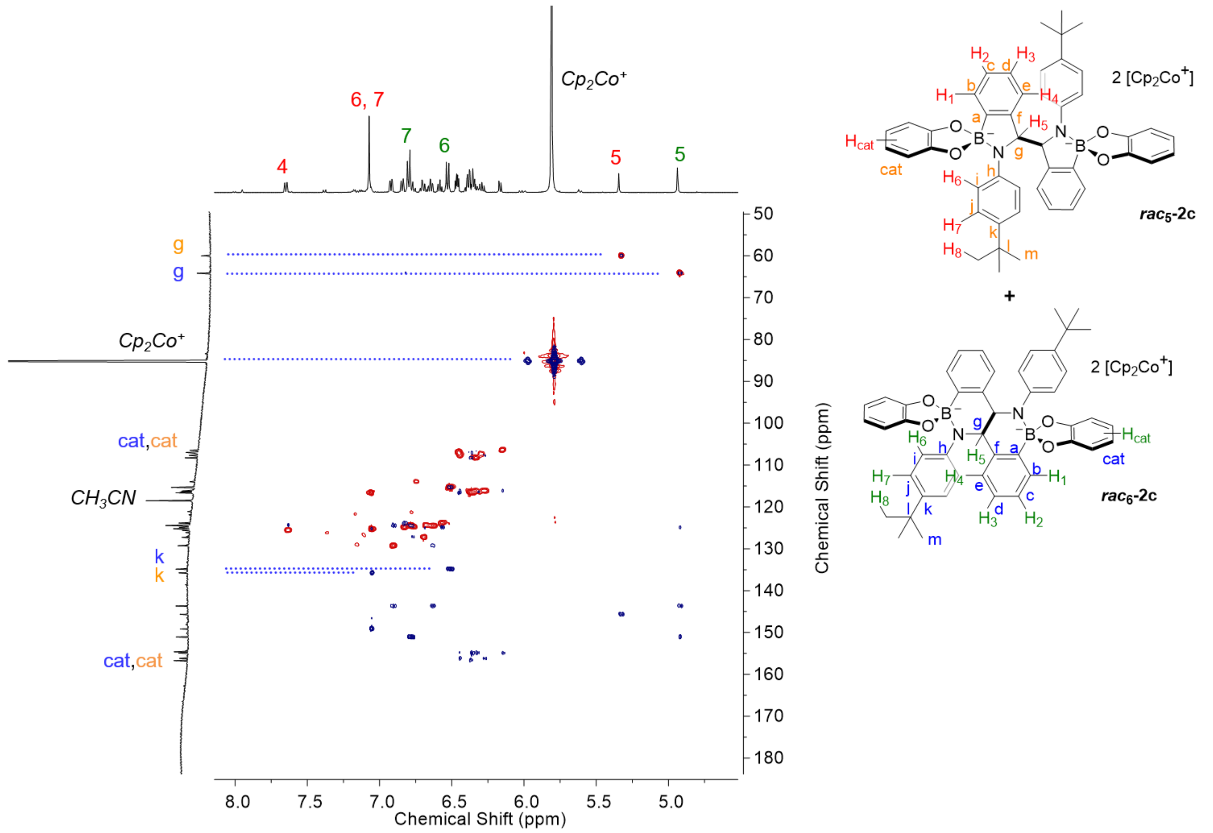

Figure 54. Overlay of HSQC (red) and HMBC (blue) spectra of ***rac*₆-2c** and ***rac*₅-2c** after partial interconversion. Key assignments are shown.

5.4 *rac*$_5$-2c

Characterisation data for the ***rac*$_5$-2c** isomer could be inferred from the NMR data of the equilibrated ***rac*$_5$-2c** and ***rac*$_6$-2c** mixture in Section 5.3. However, only the ^{13}C NMR shifts are reported below due to overlapping signals with ***rac*$_6$-2c** in the ^{1}H NMR spectrum. Figure S55 shows the assignment of both isomers in the ^{1}H NMR spectrum

^{13}C NMR (126 MHz, 298.0 K, DMSO-*d*$_6$): δ 155.7 (C_{cat}), 154.4 (C_{cat}), 149.6 (b, C_a), 148.7 (C_h), 145.2 (C_f), 135.3 (C_k), 126.8 (C_b), 125.1 (C_e), 124.7 ($C_{i/j}$), 124.0 ($C_{c,d}$), 116.1 ($C_{i/j}$), 115.7 (C_{cat}), 115.6 (C_{cat}), 107.2 (C_{cat}), 106.0 (C_{cat}), 84.7 (Cp_2Co^+), 59.4 (C_g), 33.4 (C_l), 31.7 (C_m)

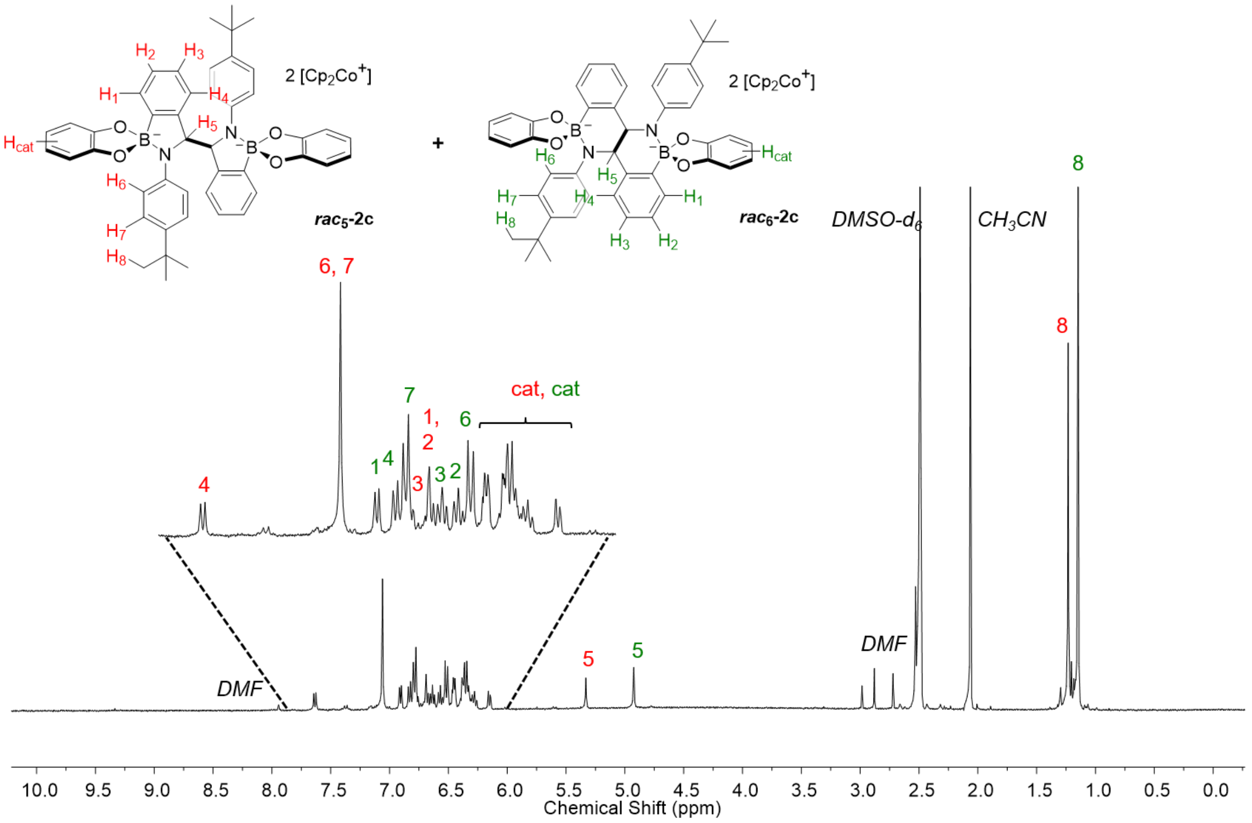

Figure S55. ^{1}H NMR spectrum (400 MHz, DMSO-*d*$_6$) of ***rac*$_6$-2c and *rac*$_5$-2c** after partial interconversion.

6 Solution Characterisation of the Methoxyaniline-Pyrocatechol Reductively Coupled Dimer (2d)

6.1 *rac*$_5$-2d and *meso*$_5$-2d Mixture in CD_3CN

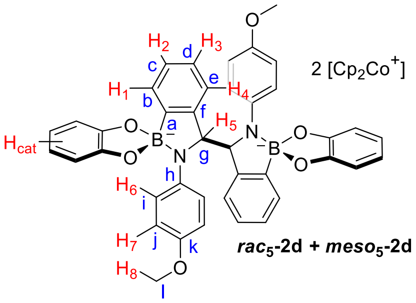

***rac*$_5$-2d + *meso*$_5$-2d**

In a nitrogen atmosphere glove box, Cp_2Co (3.00 mg, 0.016 mmol) was dissolved in 0.5 mL of CD_3CN. This solution was then agitated with **1d** (5.21 mg, 0.016 mmol) until the solid was fully dissolved and transferred to a J. Young NMR tube.

As ***meso*$_5$-2d** was not observed to crystallise from the reaction mixture, it was characterised in solution as a mixture with ***rac*$_5$-2d**. Signals marked with ' are attributed to the *meso*-diastereomer. Unlike the reductive couplings of **1a-1c**, small quantities of ***rac*$_6$-2d** were also observed in the reaction mixture in CD_3CN before it crystallised from the reaction mixture (Figure S56). The spectrum of the reaction mixture was not observed to change significantly over 6 h (Figure S57). As a complex mixture of three products, complete assignment of the ^{13}C NMR data was not possible (Figure S58). The signals corresponding to ***rac*$_5$-2d** were fully assigned (with the exception of carbon *a* since this signal is too broad) and assignments of ***meso*$_5$-2d** are reported below as signals marked with '.

^{1}H NMR (500 MHz, 298.0 K, CD_3CN): δ 7.79 (2H, d, ^3J = 7.5 Hz, H_4), 7.17 (4H, d, ^3J = 9.0 Hz, H_6), 7.01-6.98 (8H, m, $H_{1'-4'}$) 6.89 (2H, t, ^3J = 7.5 Hz, H_3), 6.84 (2H, d, ^3J = 6.0 Hz, H_1), 6.80 (2H, t, ^3J = 7.5 Hz, H_2), 6.78-6.74 (8H, m, $H_{7,6'}$), 6.59 (4H, d, ^3J = 7.5 Hz, H_{cat}), 6.57-6.43 (8H, m, $H_{7',cat}$), 6.43-6.34 (4H, m, H_{cat}), 6.28 (4H, t, ^3J = 7.5 Hz, H_{cat}), 5.57 (2H, s, $H_{5'}$), 5.32 (2H, s, H_5), 3.70 (6H, s, H_8), 3.59 (6H, s, $H_{8'}$).

^{13}C NMR (126 MHz, 298.0 K, CD_3CN): δ 156.9 (C_{cat}), 156.6 ($C_{cat'}$), 155.6 (C_{cat}), 155.5 ($C_{cat'}$), 151.1 (C_k), 150.0 ($C_{k'}$), 148.8 ($C_{f'}$), 147.0 (C_f), 146.9 ($C_{h'}$), 146.6 (C_h), 128.6 ($C_{b'/c'/d'/e'}$), 127.9 (C_b), 126.0 (C_e), 125.9 ($C_{b'/c'/d'/e'}$), 125.4 (C_d), 125.3 (C_c), 123.8 ($C_{b'/c'/d'/e'}$), 119.0 (C_i), 118.4 ($C_{i'}$), 117.2 ($C_{cat/cat'}$), 117.1 ($C_{cat/cat'}$), 115.1 (C_j), 114.3 ($C_{j'}$), 108.3 (C_{cat}), 108.2 ($C_{cat'}$), 107.3 ($C_{cat'}$), 107.2 (C_{cat}), 65.7 ($C_{g'}$), 61.9 (C_g), 56.2 (C_l), 56.1 ($C_{l'}$)

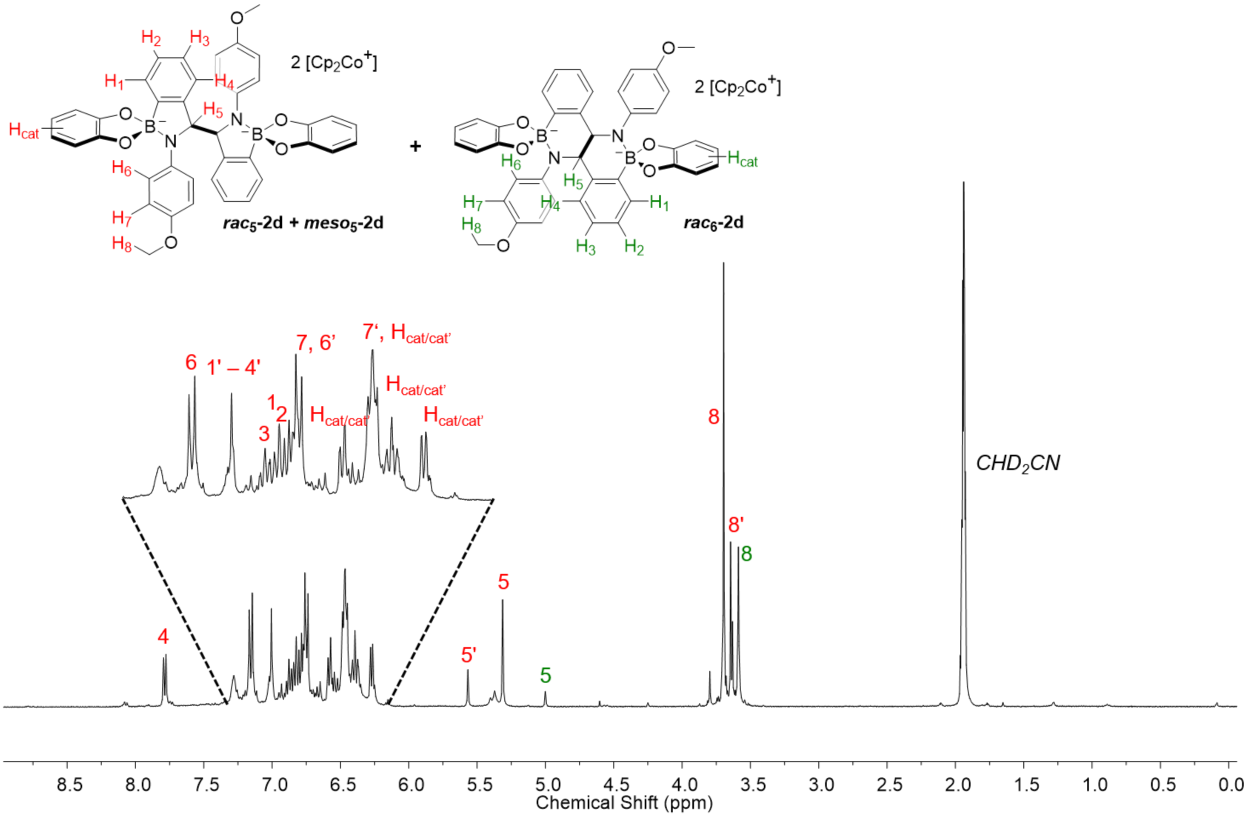

Figure S56. ^{1}H NMR spectrum (500 MHz, CD_3CN) of the reaction mixture from the reductive coupling of **1d**. Protons 1-8 in red correspond to ***rac*₅-2d** and protons 1'-8' in red to ***meso*₅-2d**. Small quantities of ***rac*₆-2d** (green signals) was also observed.

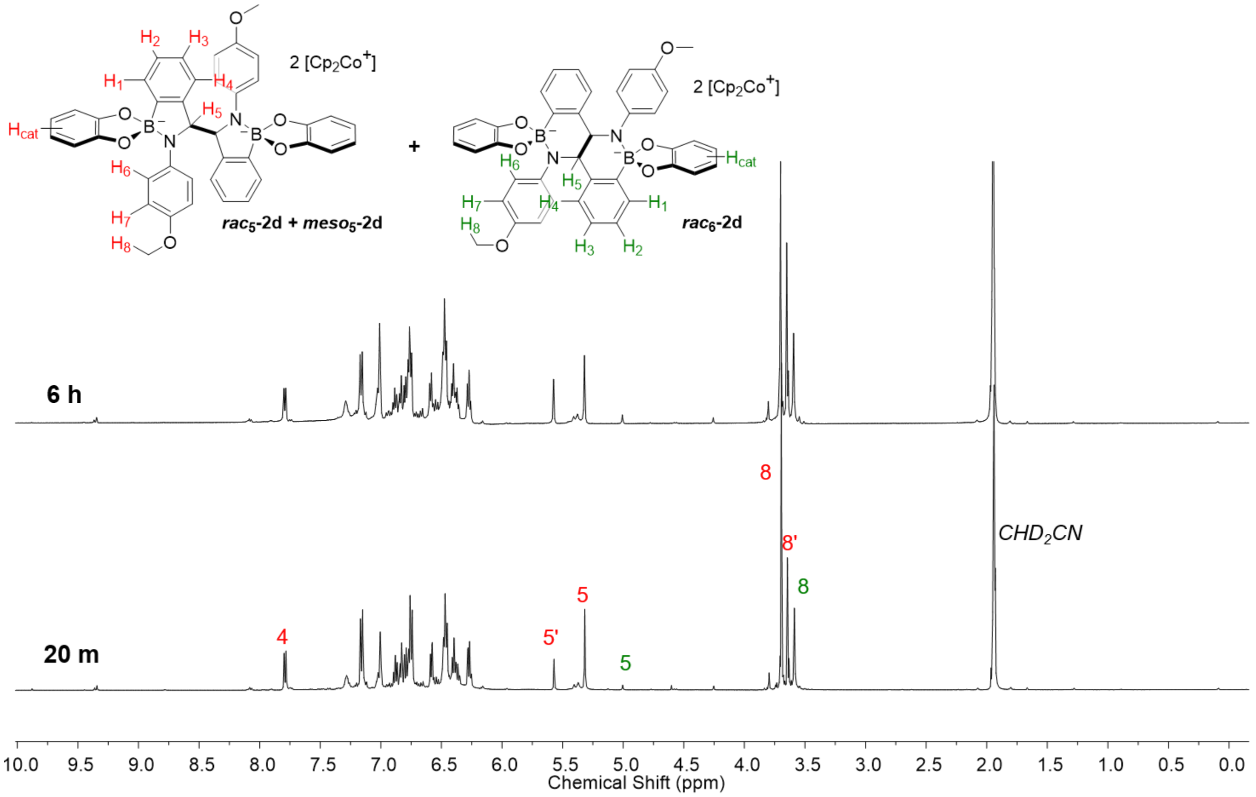

Figure S57. ^{1}H NMR spectra (500 MHz, CD_3CN) of the reaction mixture from the reductive coupling of **1d** over 6 h.

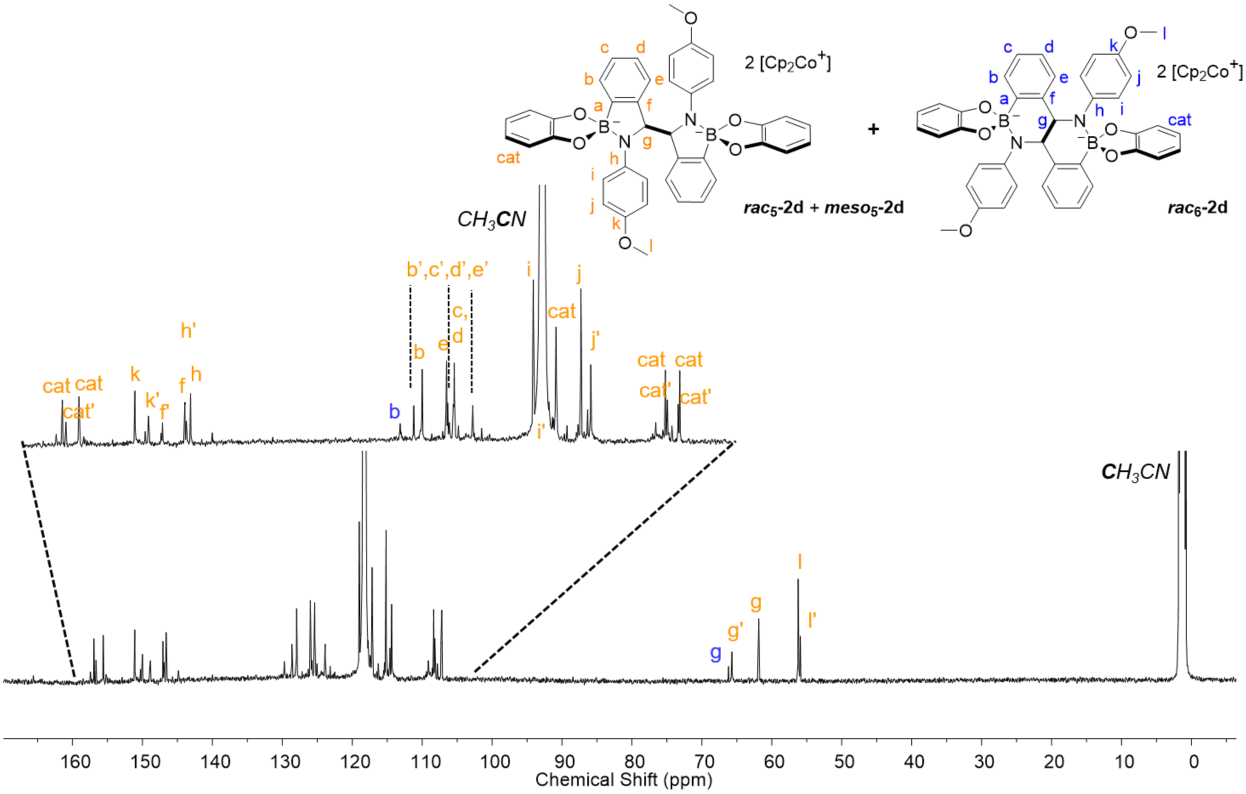

Figure S58. ^{13}C NMR spectrum (126 MHz, CD_3CN) of the reaction mixture from the reductive coupling of **1d**. Carbons in orange without prime labels correspond to ***rac*₅-2d**, carbons in orange with prime labels correspond to ***meso*₅-2d** and carbons in blue correspond to small quantities of ***rac*₅-2d**.

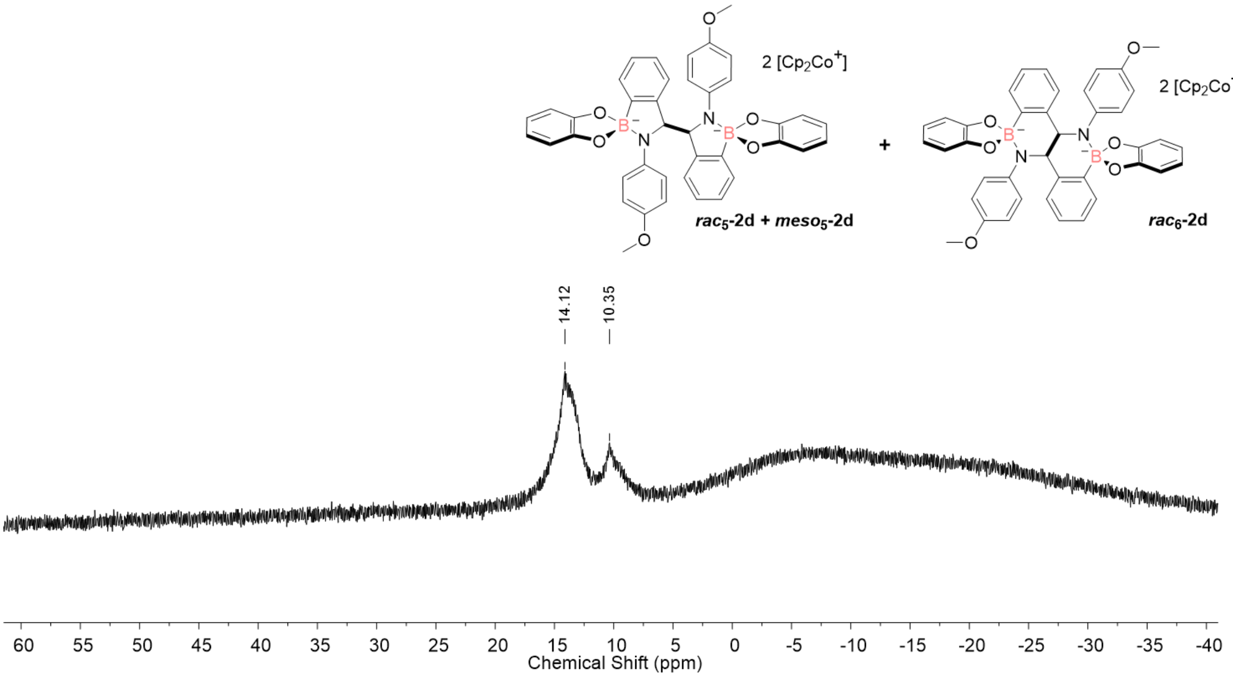

Figure S59. ^{11}B NMR spectrum (128 MHz, CD_3CN) of the reaction mixture from the reductive coupling of **1d**.

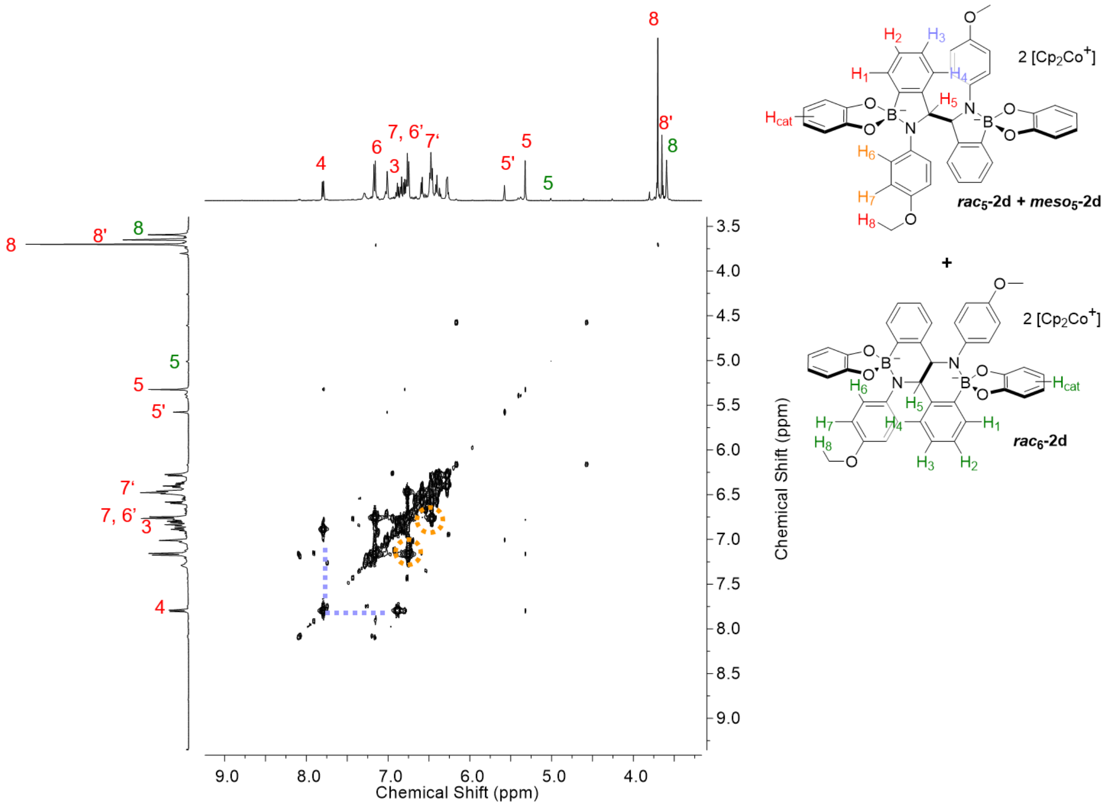

Figure S60. COSY NMR spectrum (500 MHz, CD_3CN) of the reaction mixture from the reductive coupling of **1d**. Protons 1-8 in red correspond to ***rac*₅-2d** and protons 1'-8' in red to ***meso*₅-2d**. Small quantities of ***rac*₆-2d** (green signals) was also observed.

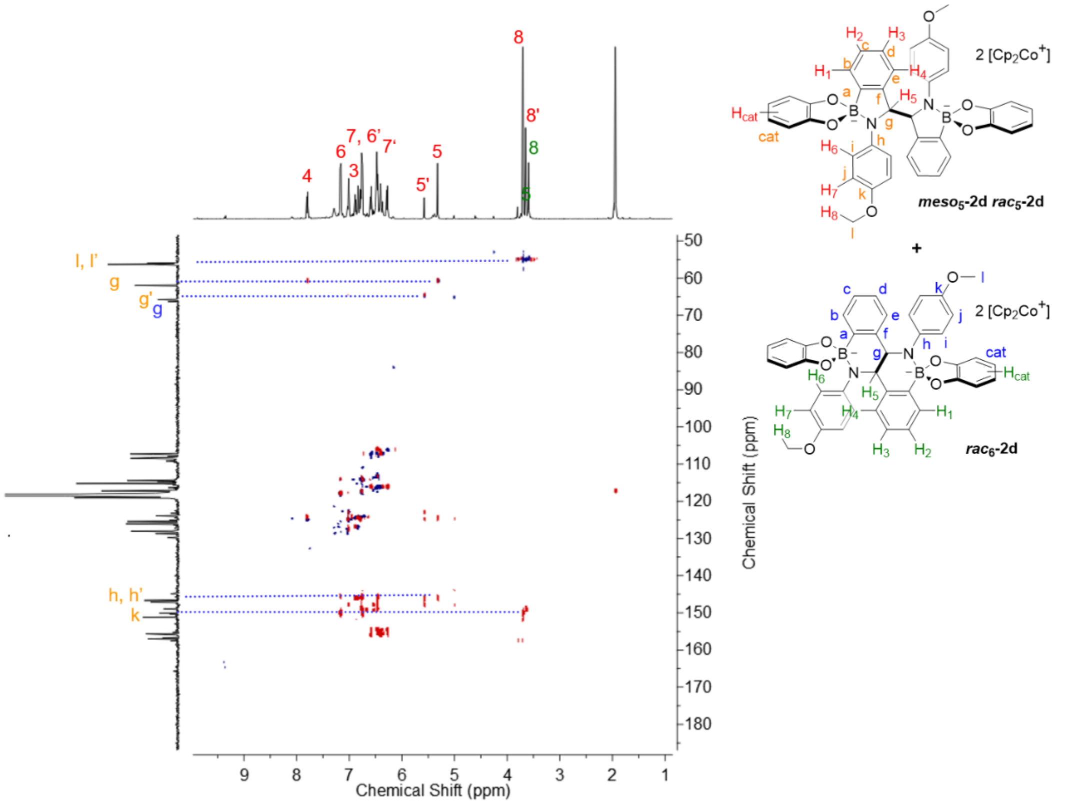

Figure S61. Overlay of HSQC (blue) and HMBC (red) NMR spectra of the reaction mixture from the reductive coupling of **1d**. Labels without primes correspond to ***rac*₅-2d** and labels with primes correspond to ***meso*₅-2d**.

6.2 *rac*$_5$-2d, *rac*$_6$-2d and *meso*$_5$-2d Mixture in DMSO-d_6

In a nitrogen atmosphere glove box, Cp_2Co (3.00 mg, 0.016 mmol) was dissolved in 0.5 mL of CD_3CN. This solution was then agitated with **1d** (5.22 mg, 0.016 mmol) until the solid was fully dissolved and transferred to a J. Young NMR tube.

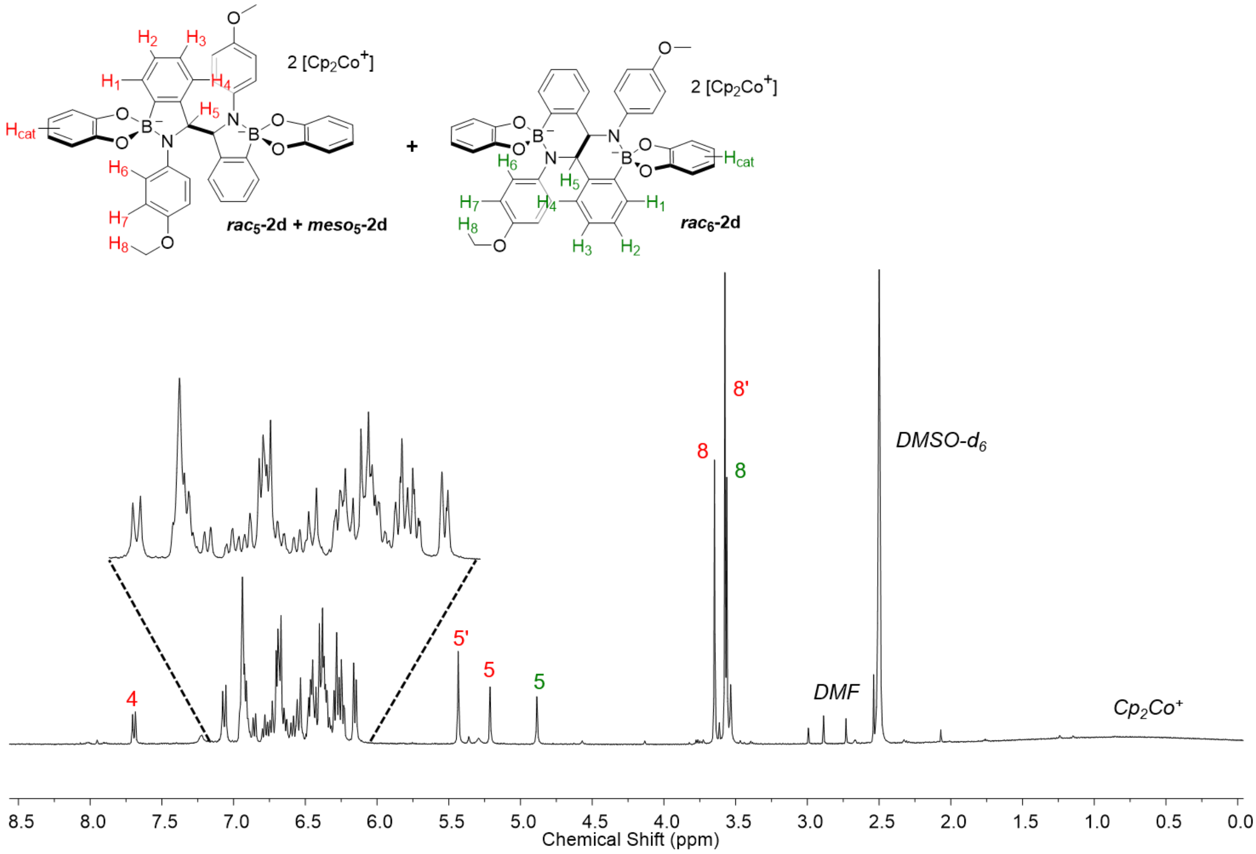

Figure S62. ^{1}H NMR spectrum (400 MHz, DMSO-d_6) of the reaction mixture from the reductive coupling of **1d**. As a complex mixture of three products, full assignment was not carried out but key protons for ***rac*$_5$-2d** (red), ***meso*$_5$-2d** (red') and ***rac*$_6$-2d** (green) were assigned.

6.3 *rac*$_6$-2d and *rac*$_5$-2d

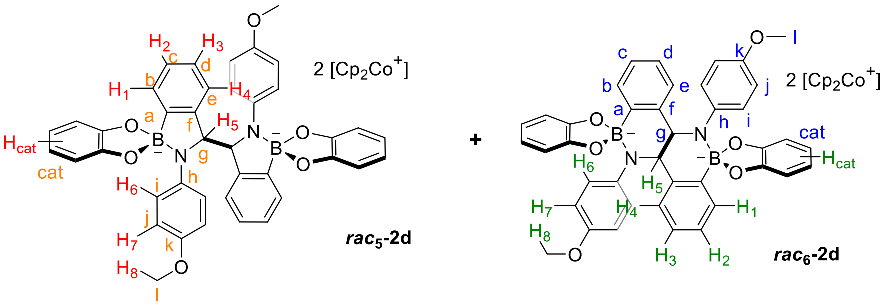

Crystals grown from the unperturbed reaction mixture in Section 6.1 were isolated by decanting the solution. They were washed twice with CH_3CN (ca. 1 mL) and redissolved in 0.5 mL DMSO-d_6.

X-ray analysis of crystals from an identical reaction were found to be ***rac*$_6$-2d** (Section 8.5), however, the NMR spectrum of the crystals immediately after they were dissolved in DMSO-d_6 showed the presence of both ***rac*$_6$-2d** and ***rac*$_5$-2d** (Figure S63). This is attributed to the fast interconversion between the two isomers. Therefore, it was not possible to characterise ***rac*$_6$-2d** before interconversion to ***rac*$_5$-2d** and signals marked with ' are attributed to ***rac*$_5$-2d** signals.

^{1}H NMR (400 MHz, 298.0 K, DMSO-d_6): δ 7.69 (2H, d, ^3J = 7.6 Hz, $H_{4'}$), 7.07 (4H, d, ^3J = 9.0 Hz, $H_{6'}$), 6.92 (2H, d, ^3J = 6.9 Hz, H_1), 6.85 (2H, d, ^3J = 7.3 Hz, H_4), 6.79 (2H, 7, ^3J = 7.6 Hz, $H_{3'}$), 6.76-6.62 (10H, m, $H_{1',2',7',3}$), 6.62-6.50 (6H, m, $H_{2,6}$), 6.50-6.41 (m, $H_{6,cat,cat'}$), 6.41-6.33 (m, $H_{6,cat,cat'}$), 6.29 (2H, t, ^3J = 7.6 Hz, $H_{cat'}$), 6.16 (2H, d, ^3J = 7.3 Hz, $H_{cat'}$), 5.21 (2H, s, $H_{5'}$), 4.89 (2H, s, H_5), 3.65 (6H, s, $H_{8'}$), 3.56 (6H, s, H_8)

The multiplets at 6.33 ppm and 6.41 ppm could not be accurately integrated since the sample was a mixture of two species and the catechol signals were overlapping in both the ^{1}H and ^{13}C NMR spectra.

^{13}C NMR (126 MHz, 298.0 K, DMSO-d_6): δ 156.4 (C_{cat}), 155.9 ($C_{cat'}$), 154.5 ($C_{cat'}$), 154.2 (C_{cat}), 149.5 ($C_{k'}$), 148.5 (C_k), 147.6 (C_h), 145.9 ($C_{h'}$), 145.4 ($C_{f'}$), 143.5 (C_f), 128.8 (C_b), 126.9 ($C_{b'}$), 124.7 ($C_{e'}$), 124.6 (C_e), 124.3 ($C_{d'}$), 124.1 (C_d), 123.9 ($C_{c'}$), 123.5 (C_d), 117.7 ($C_{i'}$), 116.3 ($C_{cat/cat'}$), 116.0 ($C_{cat/cat'}$), 115.8 (C_i), 115.7 ($C_{cat/cat'}$), 114.0 ($C_{j'}$), 113.4 (C_j), 107.9 (C_{cat}), 107.2 ($C_{cat'}$), 106.5 (C_{cat}), 106.0 ($C_{cat'}$), 85.6 (b, Cp_2Co^+), 64.4 (C_g), 60.3 ($C_{g'}$), 55.3 ($C_{l,l'}$)

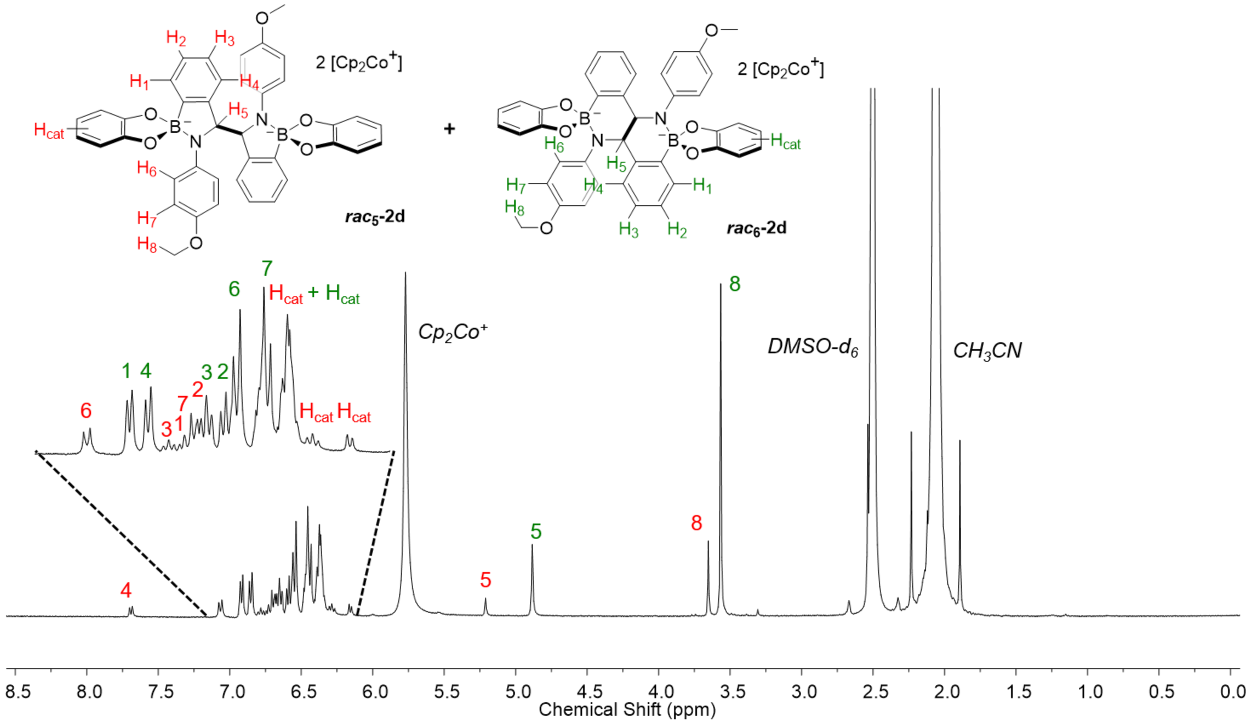

Figure 63. ^{1}H NMR spectrum (400 MHz, DMSO-d_6) of ***rac*₆-2d** and ***rac*₅-2d** after partial interconversion.

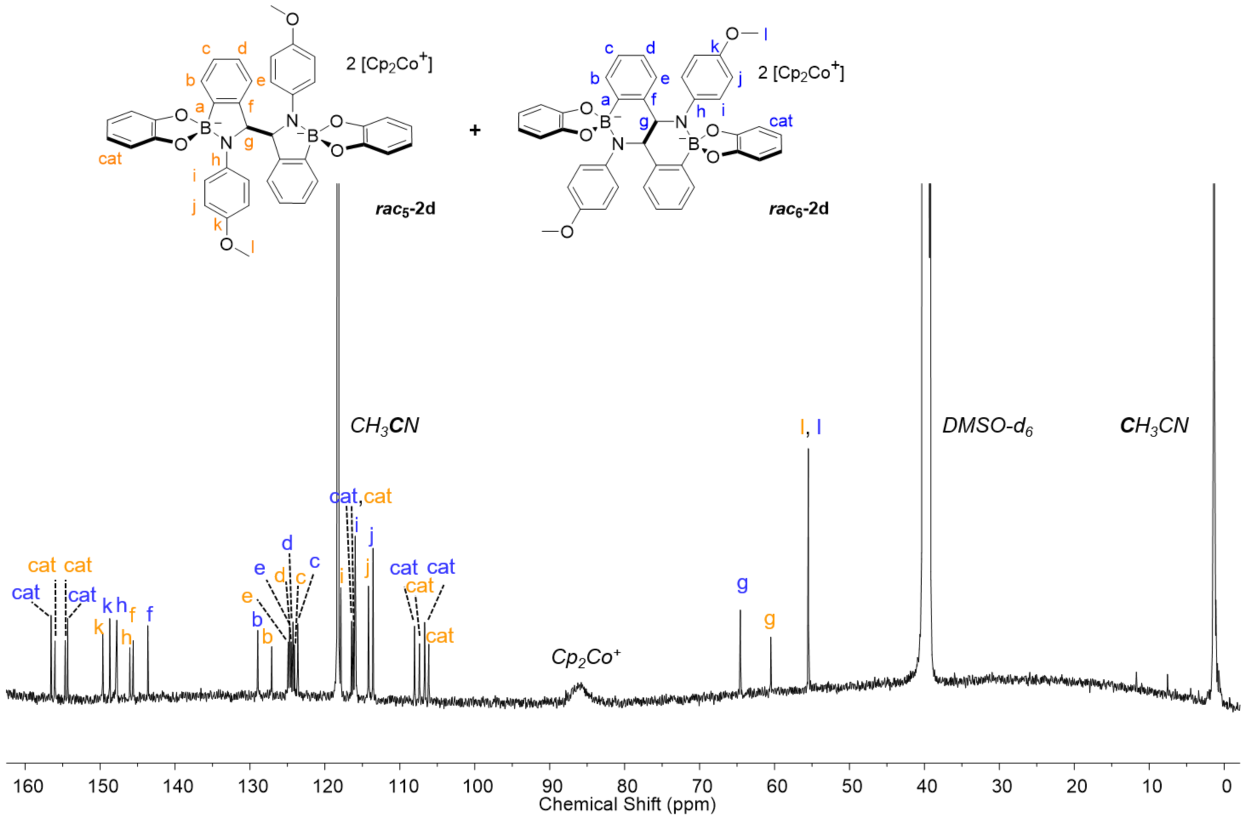

Figure 64. ^{13}C NMR spectrum (126 MHz, DMSO-d_6) of ***rac*₆-2d** and ***rac*₅-2d** after partial interconversion.

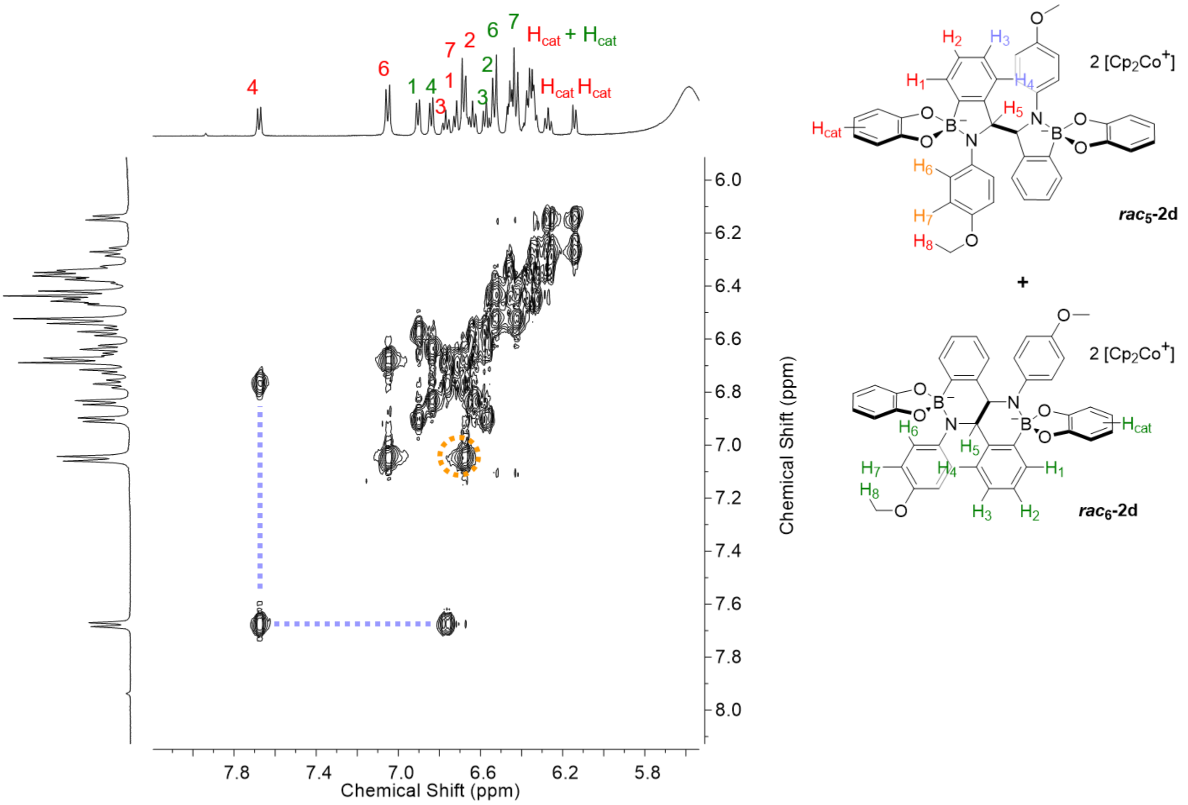

Figure 65. COSY NMR spectrum (500 MHz, DMSO-d_6) of ***rac*₆-2d** and ***rac*₅-2d** after partial interconversion.

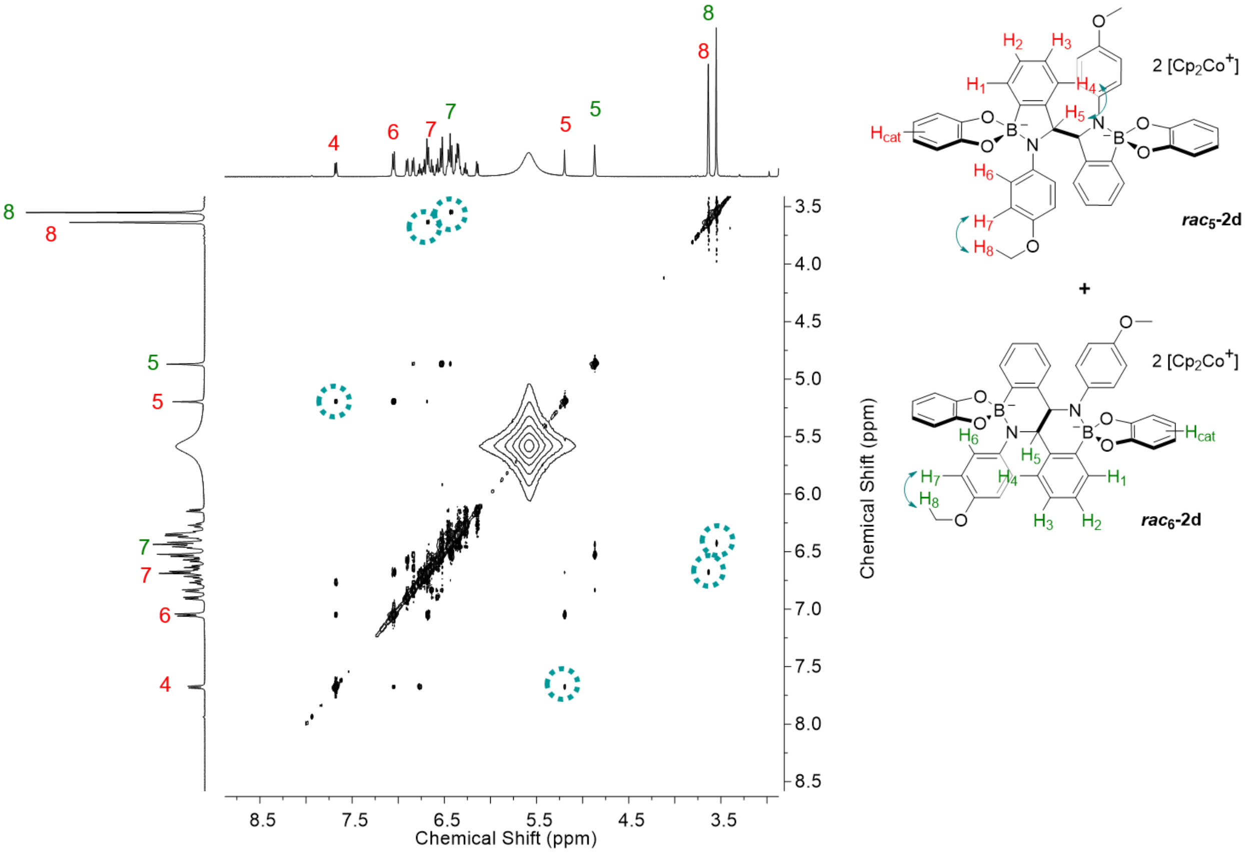

Figure 66. NOESY NMR spectrum (500 MHz, DMSO-d_6) of ***rac*₆-2d** and ***rac*₅-2d** after partial interconversion.

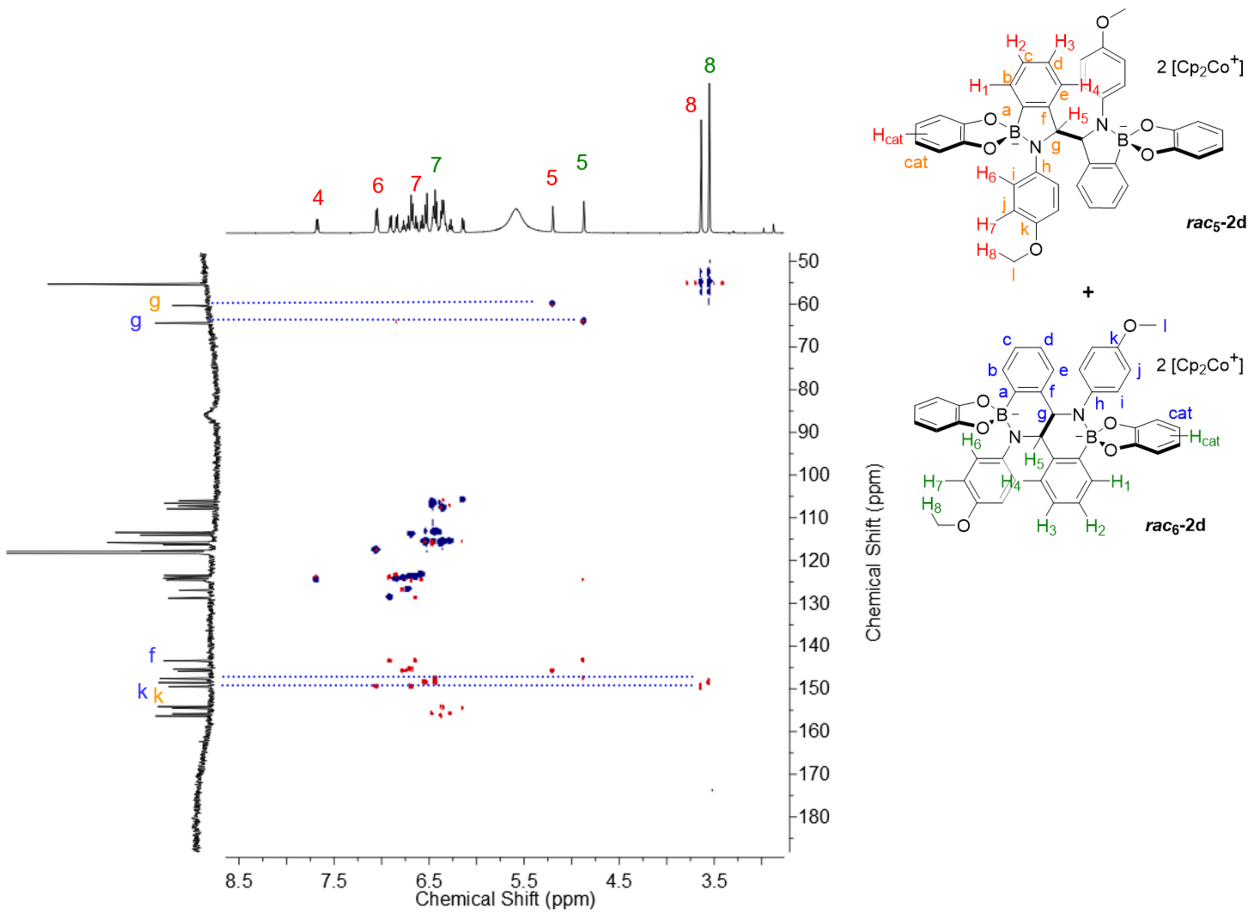

Figure 67. Overlay of HSQC (blue) and HMBC (red) NMR spectra (DMSO-d_6) of ***rac*₆-2d** and ***rac*₅-2d** after partial interconversion.

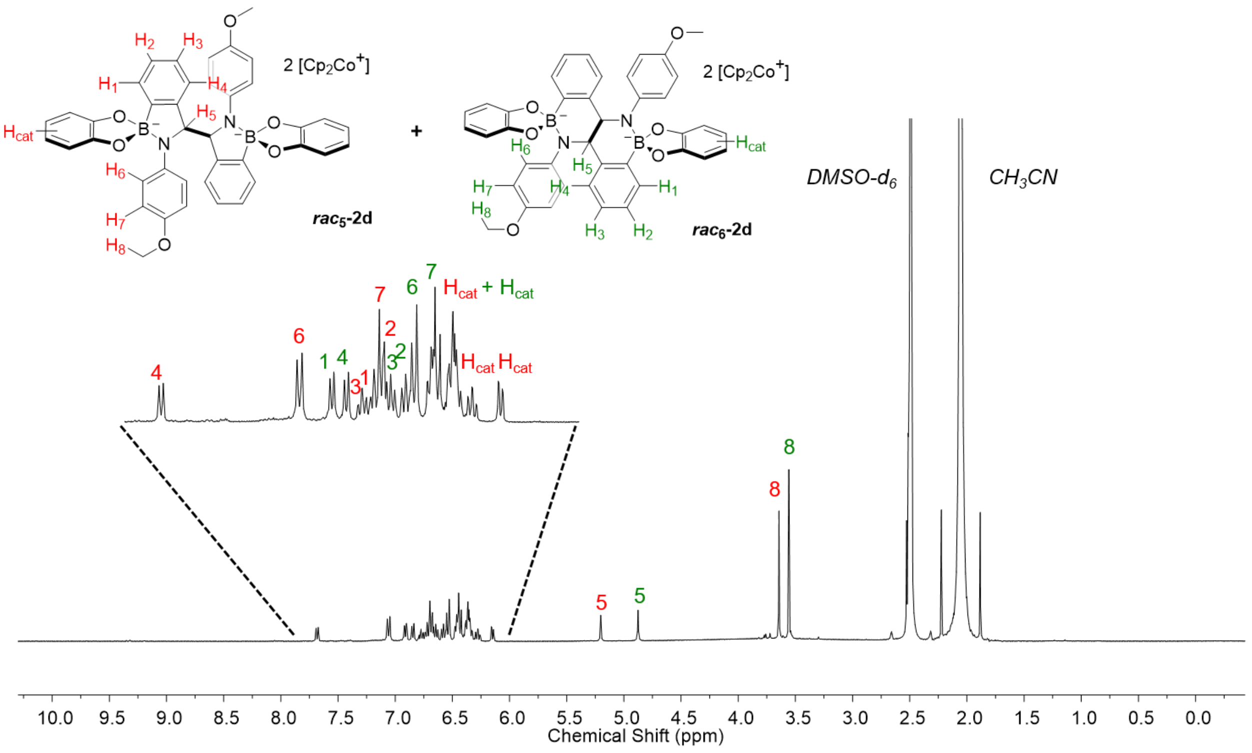

Figure S68. ^{1}H NMR spectrum (400 MHz, DMSO-d_6) of ***rac*₆-2d and *rac*₅-2d** after equilibration.

7 Solution Characterisation of the Toluidine-Tetrachlorocatechol Reductively Coupled Dimer (2e)

7.1 ***rac*₅-2e** and ***meso*₅-2e** Mixture in CD_3CN

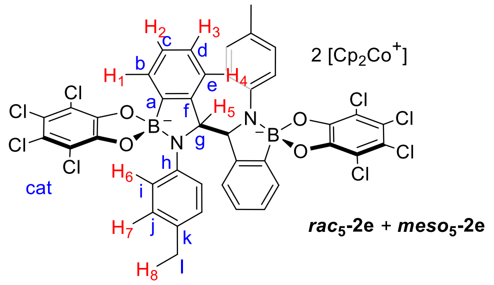

In a nitrogen atmosphere glove box, Cp_2Co (2.52 mg, 0.013 mmol) was dissolved in 0.5 mL of CD_3CN. This solution was then agitated with **1e** (6.00 mg, 0.013 mmol) until the solid was fully dissolved and transferred to a J. Young NMR tube.

As ***meso*$_5$-2e** was not observed to crystallise from the reaction mixture, it was characterised in solution as a mixture with ***rac*$_5$-2e**. Signals marked with ' are attributed to the *meso*-diastereomer.

^{1}H NMR (400 MHz, 298.0 K, CD_3CN): δ 7.73 (2H, d, ^3J = 7.7 Hz, H_4), 7.13-6.94 (18H, m, $H_{3,6,7,1'-4'}$), 6.88-6.80 (4H, m, $H_{1,2}$), 6.72 (4H, d, ^3J = 8.5 Hz, $H_{7'}$), 6.62 (4H, d, ^3J = 8.5 Hz, $H_{6'}$), 5.65 (2H, s, $H_{5'}$), 5.43 (2H, s, H_5), 2.23 (3H, s, H_8), 2.15 (3H, s, $H_{8'}$)

^{13}C NMR (126 MHz, 298.0 K, CD_3CN): δ 153.4 (C_{cat}), 153.2 ($C_{cat'}$), 152.3 ($C_{cat'}$), 152.2 (C_{cat}), 148.8 ($C_{h,h'}$), 148.6 ($C_{f'}$), 146.5 (C_f), 130.3 ($C_{a'/b'/c'/d'}$), 129.5 ($C_{j'}$), 128.6 ($C_{a'/b'/c'/d'}$), 127.8 (C_b), 126.8 ($C_{a'/b'/c'/d'}$), 126.3 ($C_{e,d}$), 125.8 (C_c), 125.3 ($C_{a'/b'/c'/d'}$), 123.9 (C_k), 123.6 ($C_{k'}$), 118.2 (C_i), 117.8 ($C_{i'}$), 111.7 (C_{cat}), 111.3 ($C_{cat'}$), 110.3 ($C_{cat'}$), 110.2 (C_{cat}), 65.7 ($C_{g'}$), 61.8 (C_g), 20.6 ($C_{l'}$), 20.5 (C_l)

^{11}B NMR (128 MHz, 298.0 K, CD_3CN): δ 14.83 (bs)

HRMS-NSI: *m/z* calcd for $C_{40}H_{24}{}^{10}B_2Cl_8N_2O_4$ $[M-2Cp_2Co]^{2-}$ 447.9757, found $[M-2Cp_2Co]^{2-}$ 447.9766 (2.0 ppm)

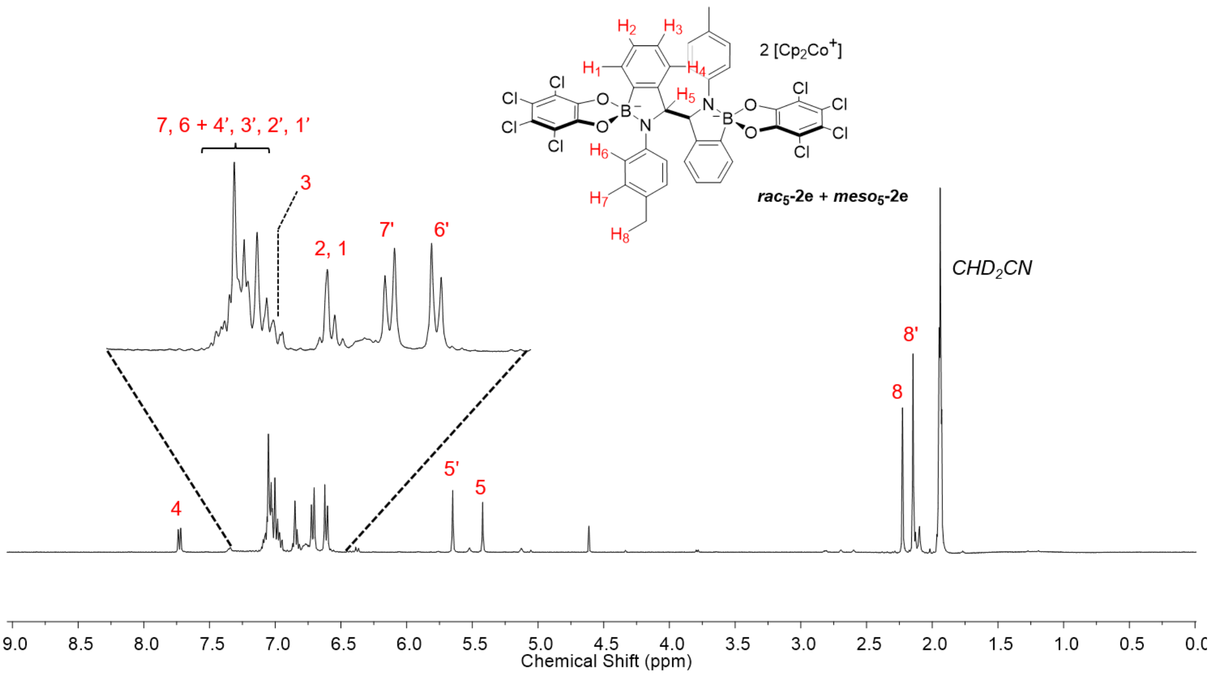

Figure S69. ^{1}H NMR spectrum (400 MHz, CD_3CN) of the reaction mixture from the reductive coupling of **1e**. Protons 1-8 correspond to ***rac*$_5$-2e** and protons 1'-8' to ***meso*$_5$-2e**.

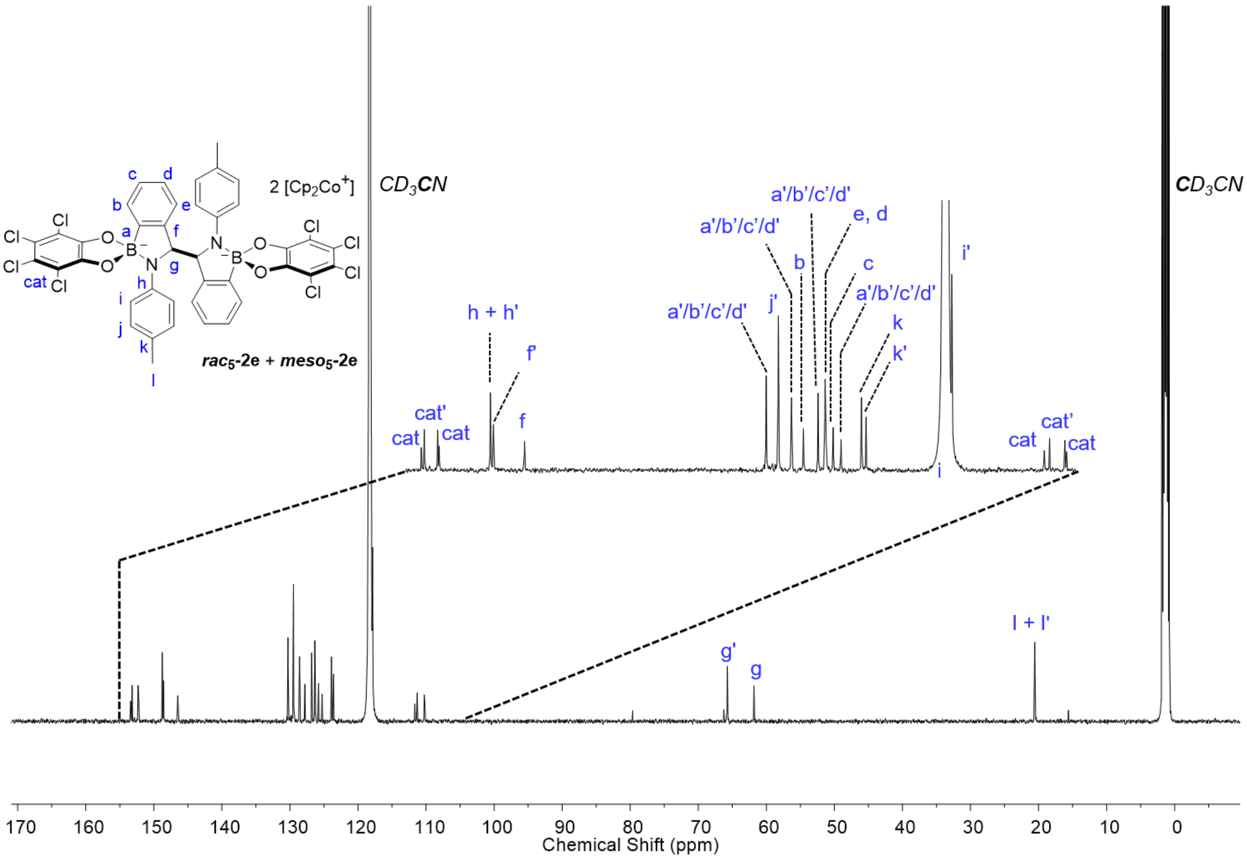

Figure S70. ^{13}C NMR spectrum (126 MHz, CD_3CN) of the reaction mixture from the reductive coupling of **1e**. Carbons without prime labels correspond to ***rac*₅-2e** and carbons with prime labels correspond to ***meso*₅-2e**.

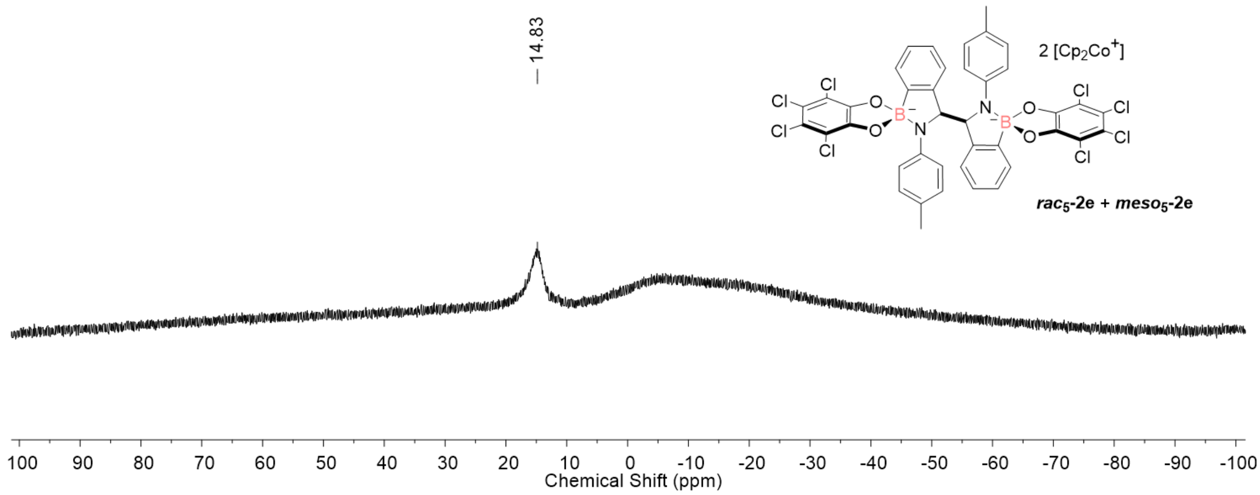

Figure S71. ^{11}B NMR spectrum (128 MHz, CD_3CN) of the reaction mixture from the reductive coupling of **1e**.

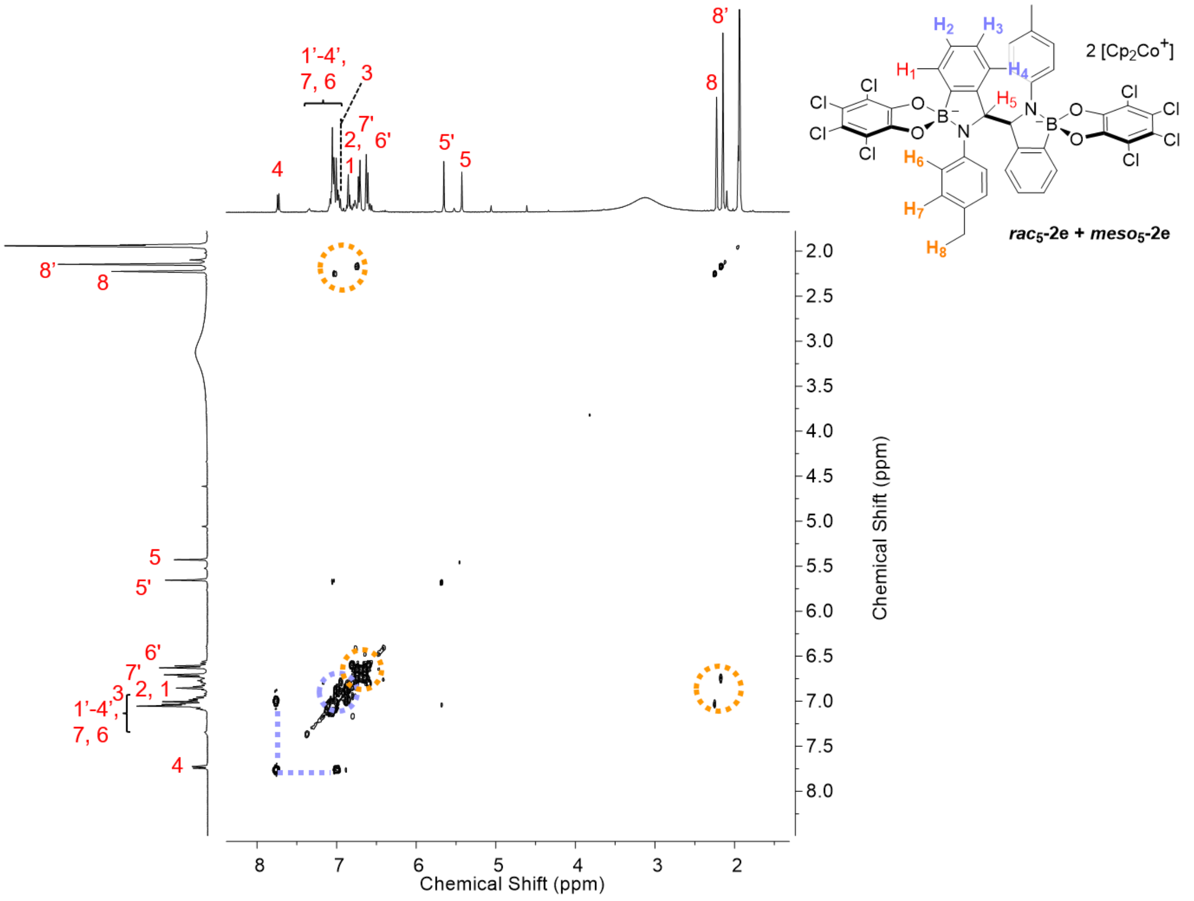

Figure S72. COSY NMR spectrum (400 MHz, CD_3CN) of the reaction mixture from the reductive coupling of **1e**. Protons 1-8 correspond to ***rac*$_5$-2e** and protons 1’-8’ to ***meso*$_5$-2e**.

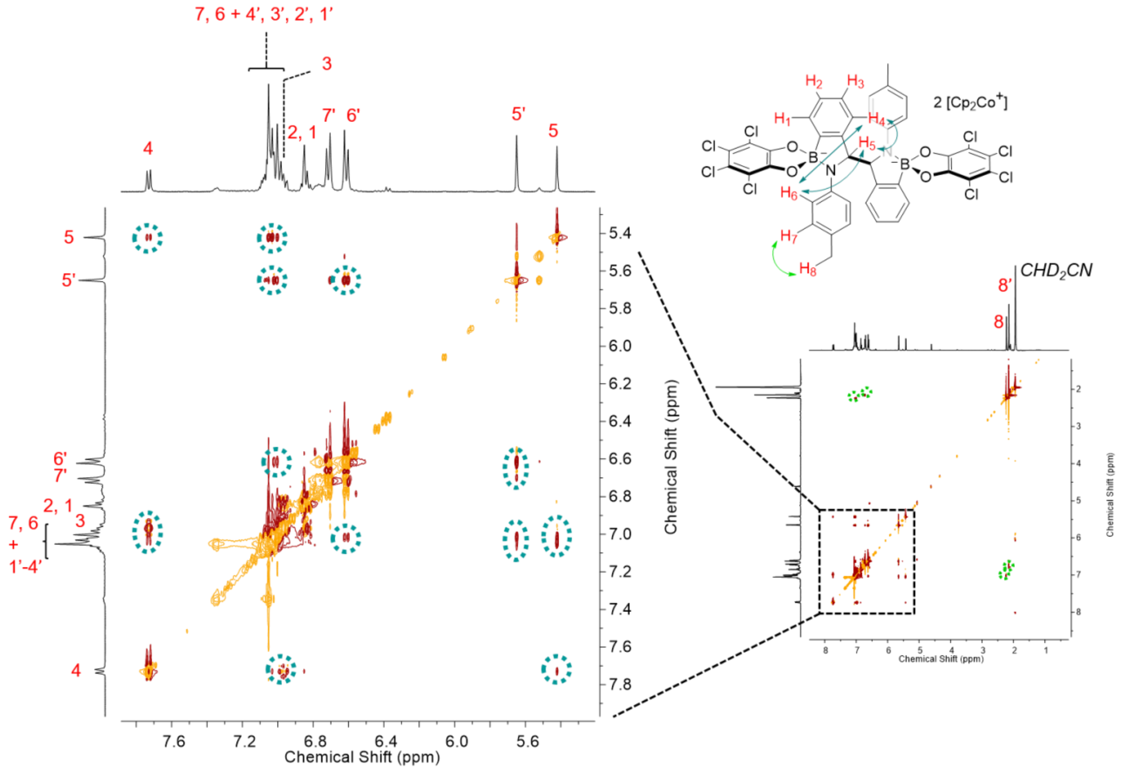

Figure S73. NOESY NMR spectrum (400 MHz, CD_3CN) of the reaction mixture from the reductive coupling of **1e**. Protons 1-8 correspond to ***rac*$_5$-2e** and protons 1’-8’ to ***meso*$_5$-2e**.

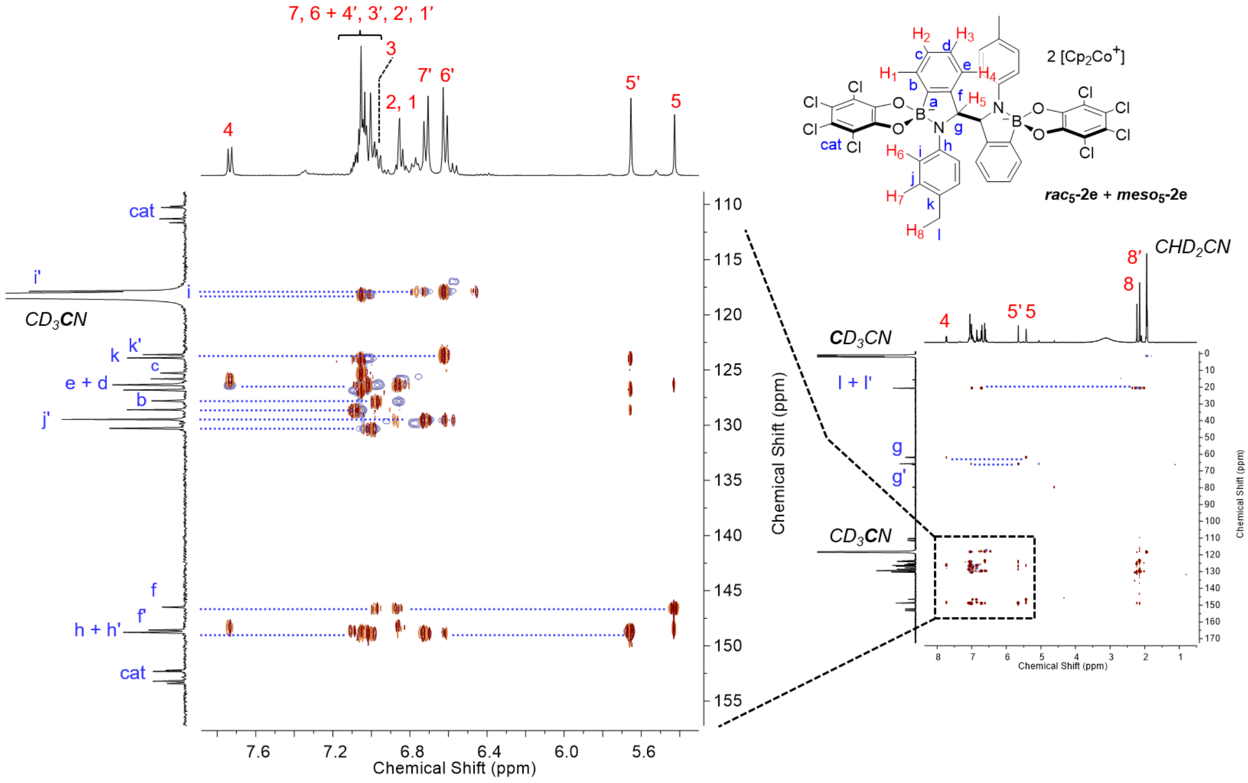

Figure S74. Overlay of HSQC (blue) and HMBC (red) NMR spectra of the reaction mixture from the reductive coupling of **1e**. Labels without primes correspond to ***rac*₅-2e** and labels with primes correspond to ***meso*₅-2e**.

7.2 *rac*₅-2e, *rac*₆-2e and *meso*₅-2e Mixture in DMSO-*d*₆

In a nitrogen atmosphere glove box, **1e** (7.15 mg, 0.016 mmol) was dissolved in 0.5 mL of DMSO-d_6 in a J Young NMR tube and Cp_2Co (3.00 mg, 0.016 mmol) was added to the solution.

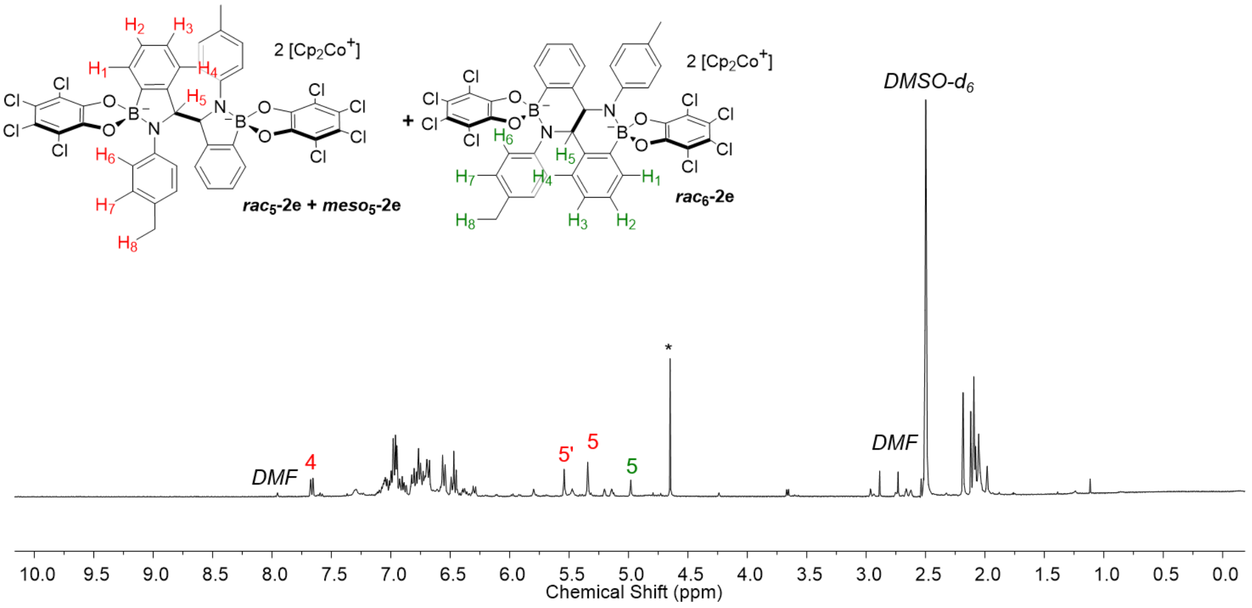

Figure S75. ^{1}H NMR spectrum of the reductive coupling of **1e** in DMSO-d_6 showing the formation of the ***rac*₅-2e**, ***rac*₆-2e** and ***meso*₅-2e** products. As a mixture of three products, full assignment was not carried out but key protons for ***rac*₅-2e** (red), ***meso*₅-2e** (red') and ***rac*₆-2e** (green) were assigned. * is attributed to a transient Cp_2Co^+ species.

7.3 *rac*$_5$-2e

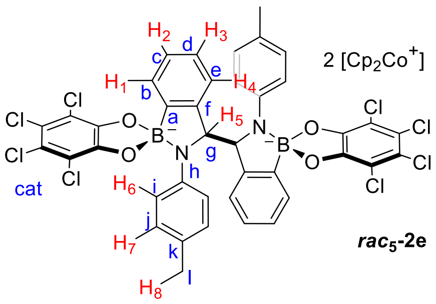

In a nitrogen atmosphere glove box, Cp_2Co (3.00 mg, 0.016 mmol) was dissolved in 0.5 mL of CH_3CN. This solution was then agitated with **1e** (7.15 mg, 0.016 mmol) until the solid was fully dissolved. Crystals grown from the unperturbed reaction mixture were isolated by decanting the solution. They were washed twice with CH_3CN (ca. 1 mL) and redissolved in 0.5 mL DMSO-d_6.

^{1}H NMR (400 MHz, 298.0 K, DMSO-d_6): δ 7.64 (2H, d, ^3J = 7.8 Hz, H_4), 6.96 (4H, d, ^3J = 8.5 Hz, H_7), 6.92 (4H, d, ^3J = 8.5 Hz, H_6), 6.87 (2H, td, ^3J = 7.8 Hz, ^4J = 1.7 Hz, H_3), 6.79-6.72 (4H, m, $H_{1,2}$), 5.32 (2H, s, H_5), 2.17 (6H, s, H_8)

The ^{13}C NMR spectrum was recorded after partial interconversion to ***rac*$_6$-2e**. Due to the overlapping signals and lack of protons on the tetrachlorocatechol unit, full assignment of the mixture was not possible. However, the signals of ***rac*$_5$-2e** (with the exception of two catechol carbons) were assigned using HSQC and HMBC NMR data acquired before the interconversion and are reported below (Figure S78).

^{13}C NMR (126 MHz, 298.0 K, DMSO-d_6): δ 152.2 (C_{cat}), 151.1 (C_{cat}), 147.3 (C_h), 146.7 (C_a), 145.0 (C_f), 129.1 (C_j), 126.6 (C_b), 125.2 (C_e), 125.0 (C_d), 124.5 (C_c), 123.3 (C_k), 116.4 (C_i), 109.8 (C_{cat}), 108.5 (C_{cat}), 84.7 (Cp_2Co^+), 60.4 (C_g), 20.1 (C_l)

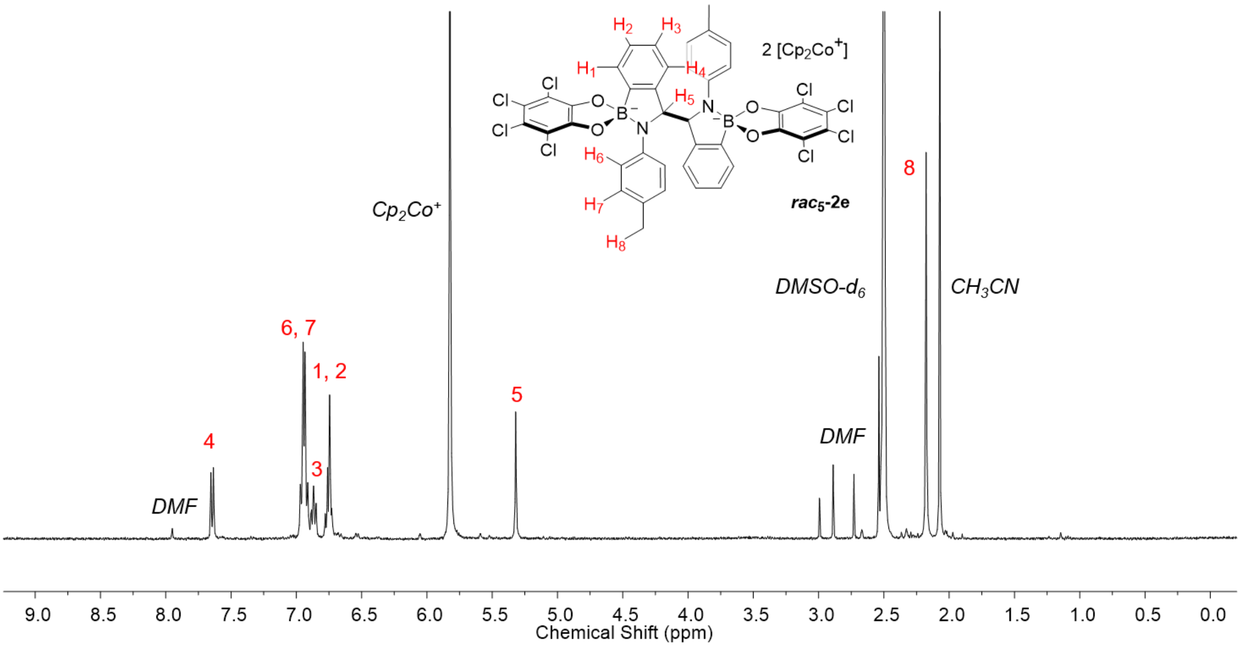

Figure S76. ^{1}H NMR spectrum (400 MHz, DMSO-d_6) of ***rac*$_5$-2e** crystals.

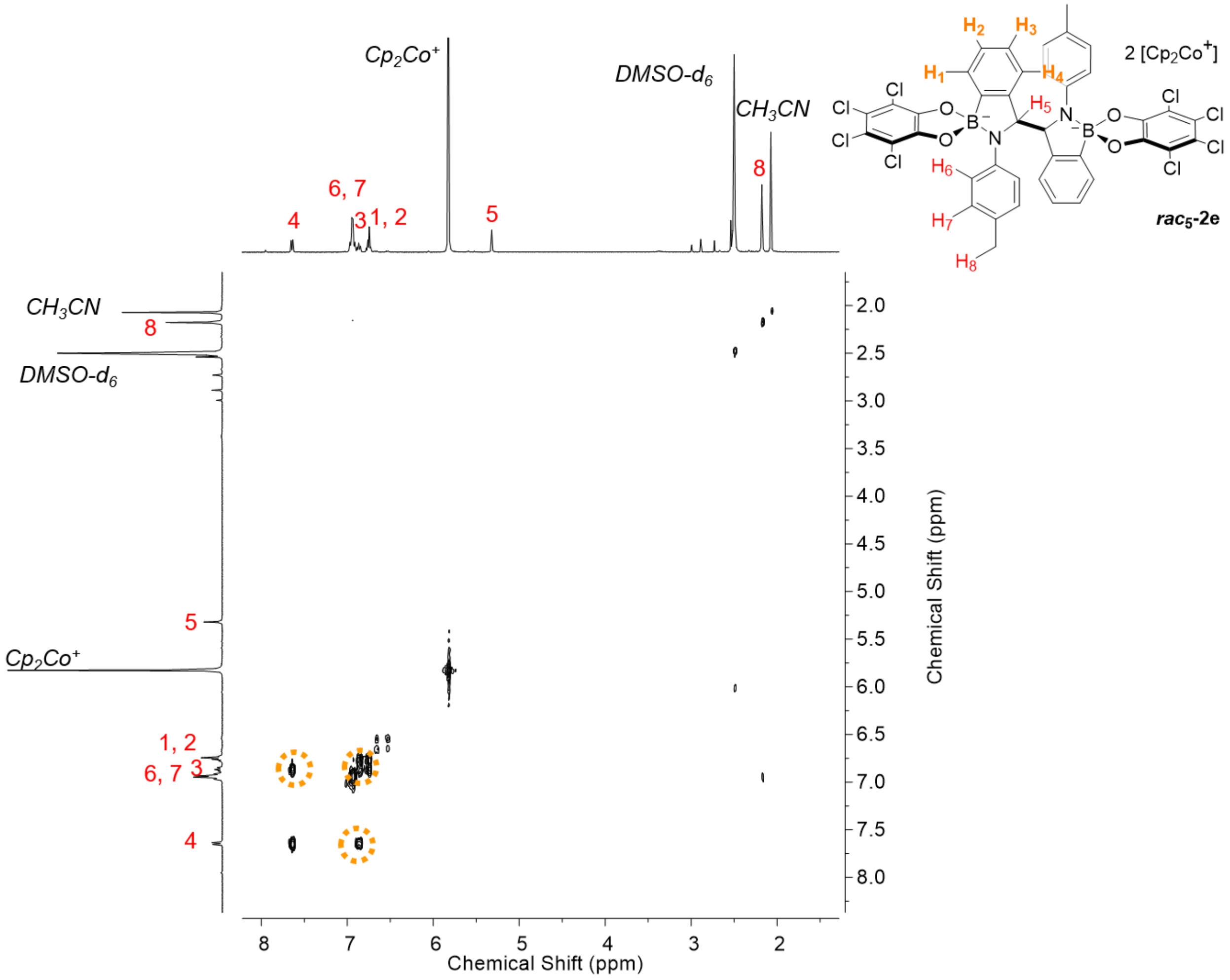

Figure S77. COSY NMR spectrum (400 MHz, DMSO-d_6) of ***rac*$_5$-2e** crystals.

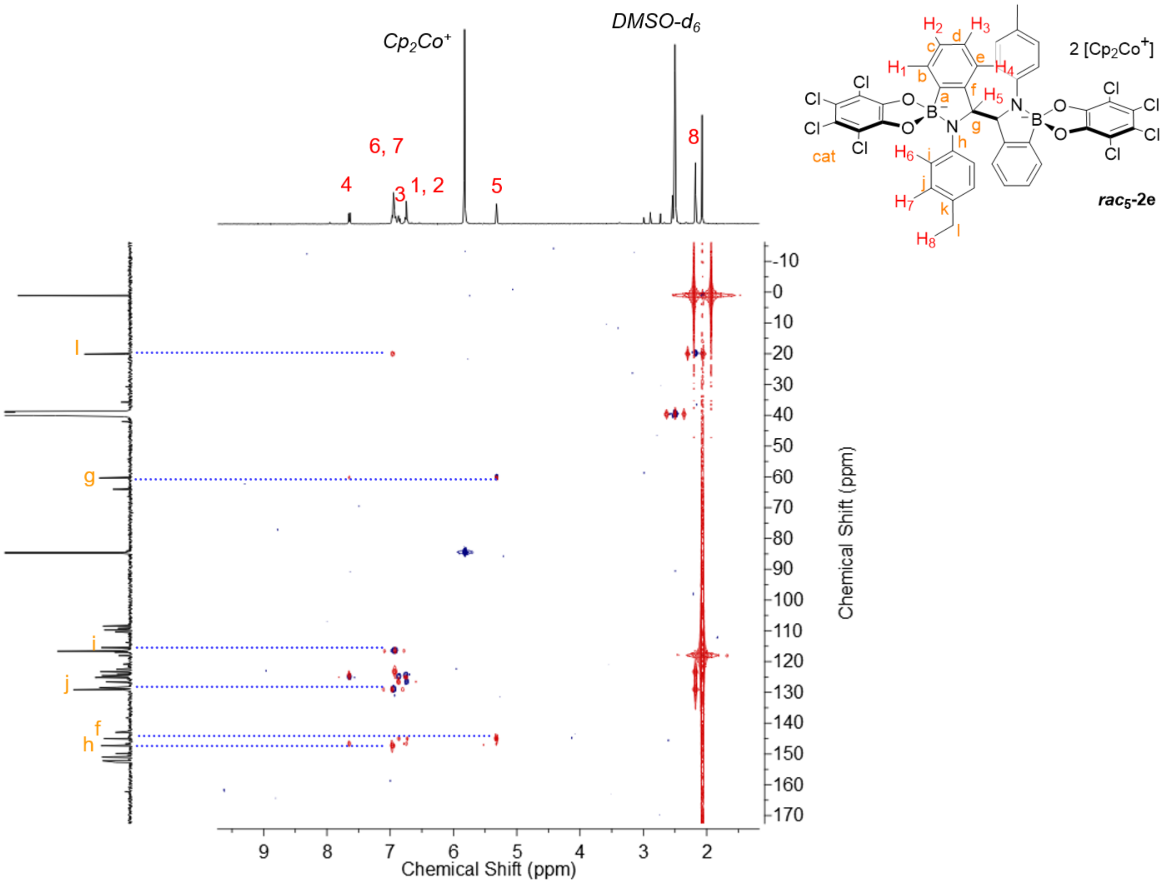

Figure S78. Overlay of HSQC (blue) and HMBC (red) NMR spectra (DMSO-d_6) of ***rac*₅-2e** crystals. Partial interconversion to ***rac*₆-2e** had occurred when the ^{13}C NMR spectrum (used here as a projection) was recorded.

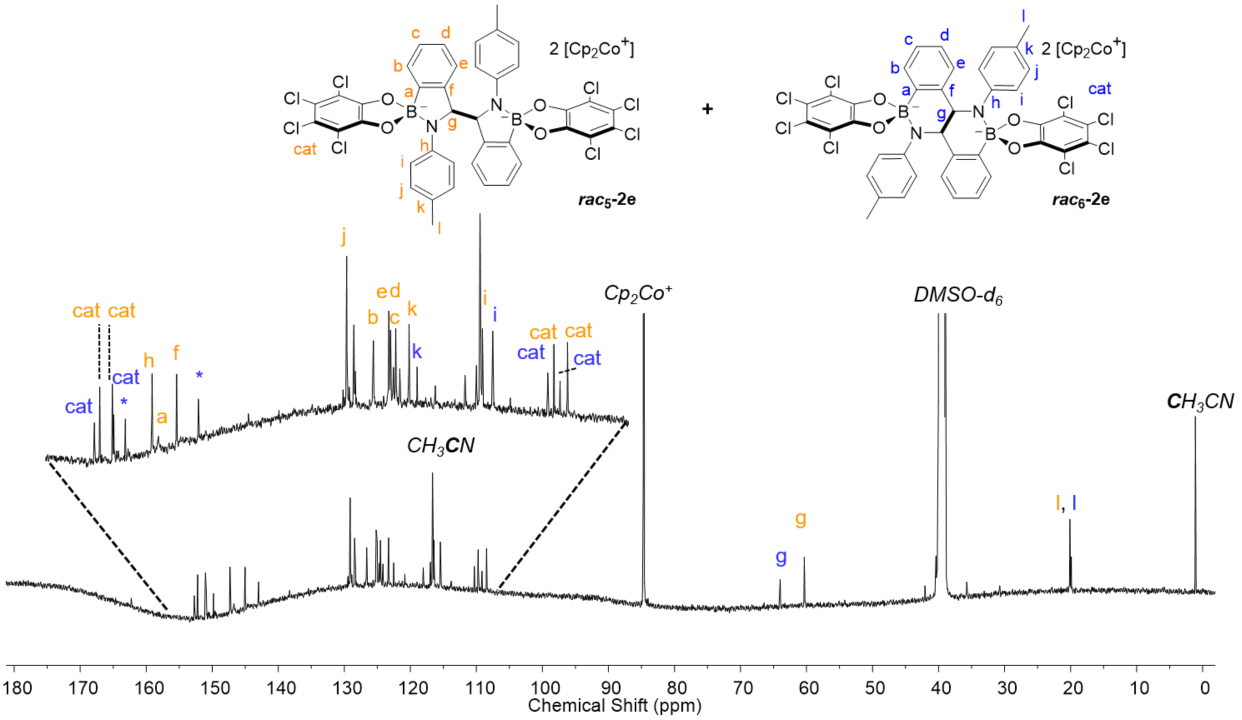

Figure S79. ^{13}C NMR spectrum (126 MHz, DMSO-d_6) of ***rac*₆-2e** and ***rac*₅-2e** after partial interconversion (unmarked and peaks marked with * could not be unambiguously assigned to specific carbon atoms on each isomer).

7.4 *rac*$_6$-2e

It was not possible to fully characterise the ***rac*$_6$-2e** isomer from the mixture with ***rac*$_5$-2e** due to the number of overlapping signals and the observance of a small amount of decomposition after 1 day at room temperature (Figure S80). Several signals could be assigned in the ^{1}H (Figure S80) and ^{13}C NMR (Figure S79) spectra based on the HSQC/HMBC NMR data in Figure S81.

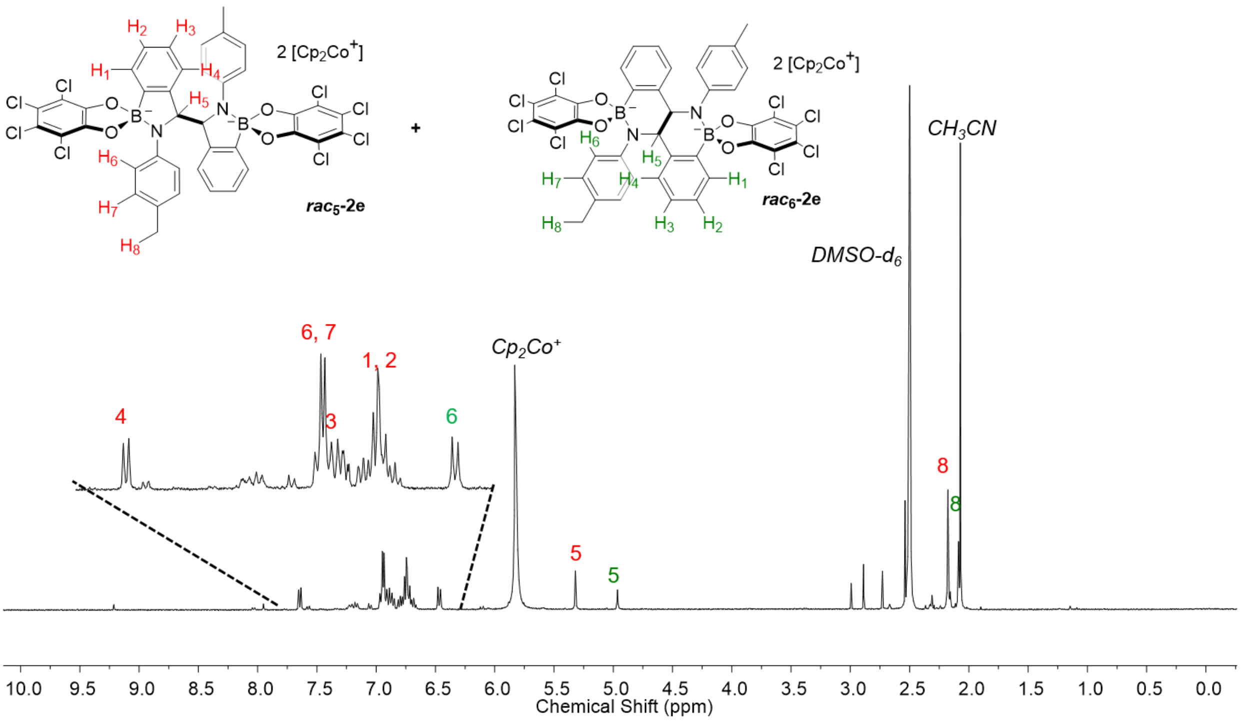

Figure S80. ^{1}H NMR spectrum (400 MHz, DMSO-d_6) of the equilibrated ***rac*$_5$-2e** and ***rac*$_6$-2e** mixture after 1 day at room temperature.

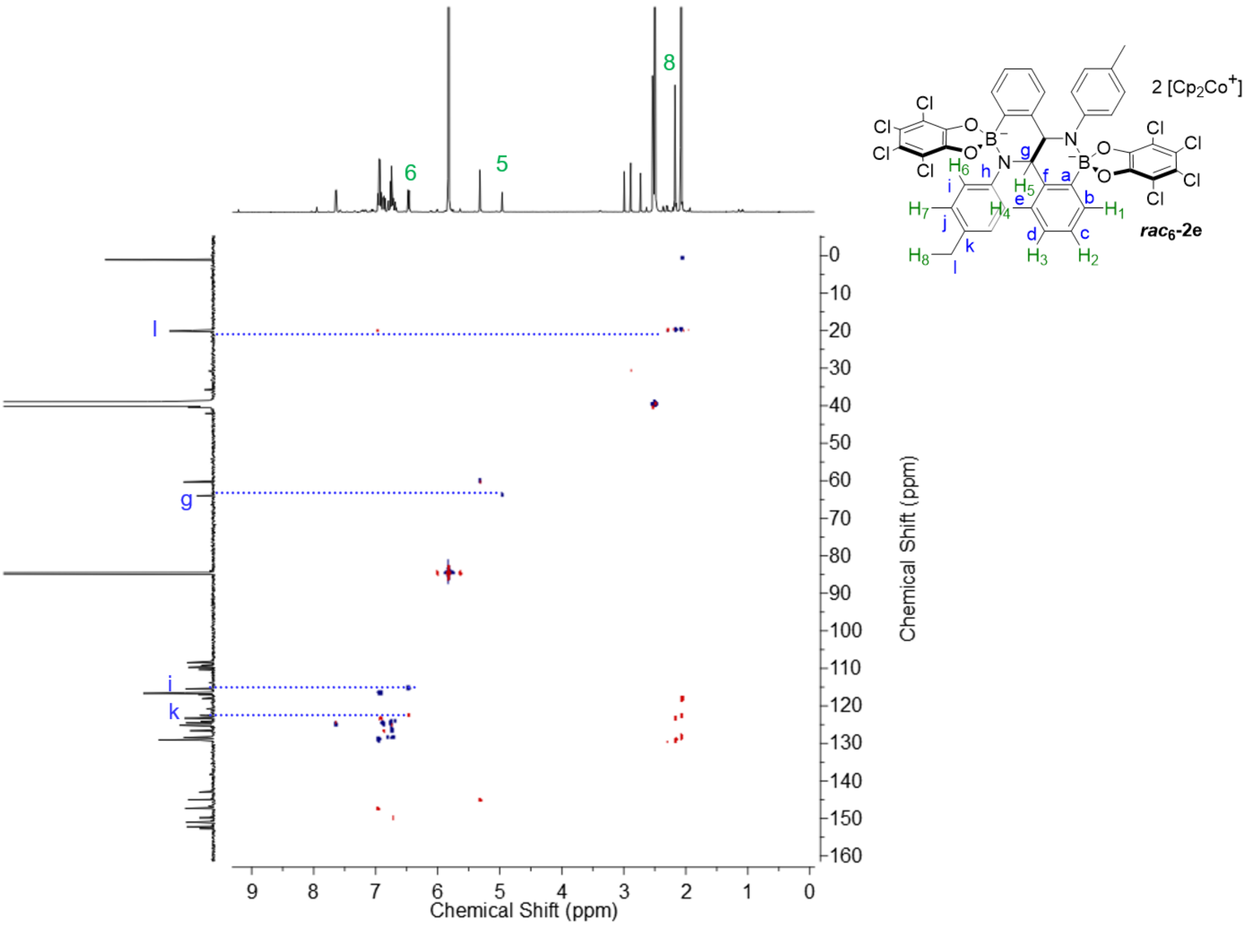

Figure S81. Overlay of HSQC (blue) and HMBC (red) NMR spectra (DMSO-d_6) of the equilibrated ***rac*$_5$-2e** and ***rac*$_6$-2e** mixture.

8 X-Ray Crystal Structures of Reductively Coupled Products

8.1 *meso*$_5$-2a

***meso*$_5$-2a**·2MeCN**:** Formula $C_{62}H_{52}B_2Co_2F_2N_4O_4$, *M* 1094.55, Orthorhombic, space group P b c a (#61), *a* 13.4339(5), *b* 23.2118(9), *c* 33.5763(14) Å, *V* 10469.9(7) Å^3, D_c 1.389 g cm^{-3}, *Z* 8, crystal size 0.180 by 0.140 by 0.080 mm, colour orange, habit block, temperature 180(2) Kelvin, λ(CuKα) 1.54178 Å, μ(CuKα) 5.455 mm^{-1}, T(SADABS)$_{min,max}$ 0.5354, 0.7516, $2\theta_{max}$ 117.84, *hkl* range -10 14, -22 25, -36 37, *N* 47478, N_{ind} 7435(R_{merge} 0.0425), N_{obs} 5934(I > 2σ(I)), N_{var} 653, residuals* $R1(F)$ 0.0612, $wR2(F^2)$ 0.1350, GoF(all) 1.093, $\Delta\rho_{min,max}$ -0.345, 0.461 e$^-$ Å^{-3}.

*$R1 = \Sigma||F_o| - |F_c||/\Sigma|F_o|$ for $F_o > 2\sigma(F_o)$; $wR2 = (\Sigma w(F_o^2 - F_c^2)^2/\Sigma(wF_c^2)^2)^{1/2}$ all reflections

$w=1/[\sigma^2(F_o^2)+(0.0311P)^2+25.5825P]$ where $P=(F_o^2+2F_c^2)/3$

In a nitrogen atmosphere glove box, a number of crystallisations were setup up from the reaction of **1a** (20.10 mg, 0.063 mmol) and Cp_2Co (12.00 mg, 0.063 mmol) in 2-3 mL of CH_3CN. Crystals were obtained as a result of slow solvent evaporation from a vapour diffusion crystallisation with toluene.

The crystals immediately lost solvent after removal from the mother liquor and rapid handling prior to flash cooling in the cryostream was required to collect data. Despite these measures and the use of a high intensity laboratory source few reflections at greater than 0.9 Å resolution were observed. Nevertheless, the quality of the data is far more than sufficient to establish the connectivity of the structure. Reflecting the less than ideal diffraction, all of the solvent molecules within the crystal lattice were disordered and modelled over two or three locations. As a consequence of this disorder there are a few short contacts involving hydrogen atoms of low occupancy acetonitrile molecules.

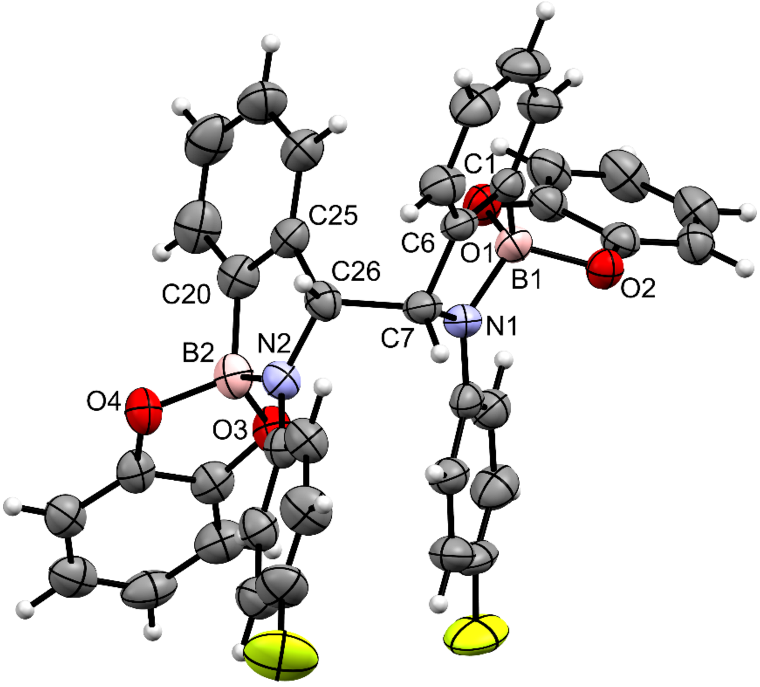

Figure S82. ORTEP diagram of the anionic portion of the structure of ***meso*₅-2a**. Thermal ellipsoids are drawn at the 50% probability level. Selected bond lengths (Å) and angles (°): C(7)-C(26) 1.575(6), N(1)-C(7) 1.467(5), N(2)-C(26) 1.460(5), C(6)-C(7) 1.512(6), C(25)-C(26) 1.513(6), B(1)-O(1) 1.529(6), B(1)-N(1) 1.529(6), B(1)-O(2) 1.532(6), B(1)-C(1) 1.591(7), B(2)-O(3) 1.515(6), B(2)-N(2) 1.530(6), B(2)-O(4) 1.531(6), B(2)-C(20) 1.596(7), N(1)-C(7)-C(6), 104.0(3), N(1)-C(7)-C(26) 115.4(3), C(6)-C(7)-C(26) 109.4(3), N(2)-C(26)-C(25) 104.4(3), N(2)-C(26)-C(7) 113.5(3), C(25)-C(26)-C(7) 113.4(3).

8.2 *rac*₆-2a

***rac*₆-2a**·3MeCN**:** Formula $C_{64}H_{55}B_2Co_2F_2N_5O_4$, *M* 1135.61, Triclinic, space group P -1 (#2), *a* 11.4517(5), *b* 13.8129(6), *c* 18.6700(7) Å, α 79.971(2), β 88.128(2), γ 70.296(2)°, *V* 2736.8(2) Å^3, D_c 1.378 g cm^{-3}, *Z* 2, crystal size 0.300 by 0.170 by 0.150 mm, colour orange, habit block, temperature 180(2) Kelvin, λ(CuKα) 1.54178 Å, μ(CuKα) 5.243 mm^{-1}, T(SADABS)$_{min,max}$ 0.5529, 0.7528, $2\theta_{max}$ 133.50, *hkl* range -13 13, -16 16, -22 21, *N* 31031, N_{ind} 9631(R_{merge} 0.0331), N_{obs} 8514(I > 2σ(I)), N_{var} 715, residuals* $R1(F)$ 0.0361, $wR2(F^2)$ 0.0991, GoF(all) 1.066, $\Delta\rho_{min,max}$ -0.236, 0.558 e$^-$ Å^{-3}.

*$R1 = \Sigma||F_o| - |F_c||/\Sigma|F_o|$ for $F_o > 2\sigma(F_o)$; $wR2 = (\Sigma w(F_o^2 - F_c^2)^2/\Sigma(wF_c^2)^2)^{1/2}$ all reflections

$w=1/[\sigma^2(F_o^2)+(0.0569P)^2+0.5153P]$ where $P=(F_o^2+2F_c^2)/3$

Crystals were obtained from the unperturbed reaction of **1a** (11.62 mg, 0.036 mmol) and Cp_2Co (6.93 mg, 0.037 mmol) in 1 mL of CD_3CN in a J Young NMR tube.

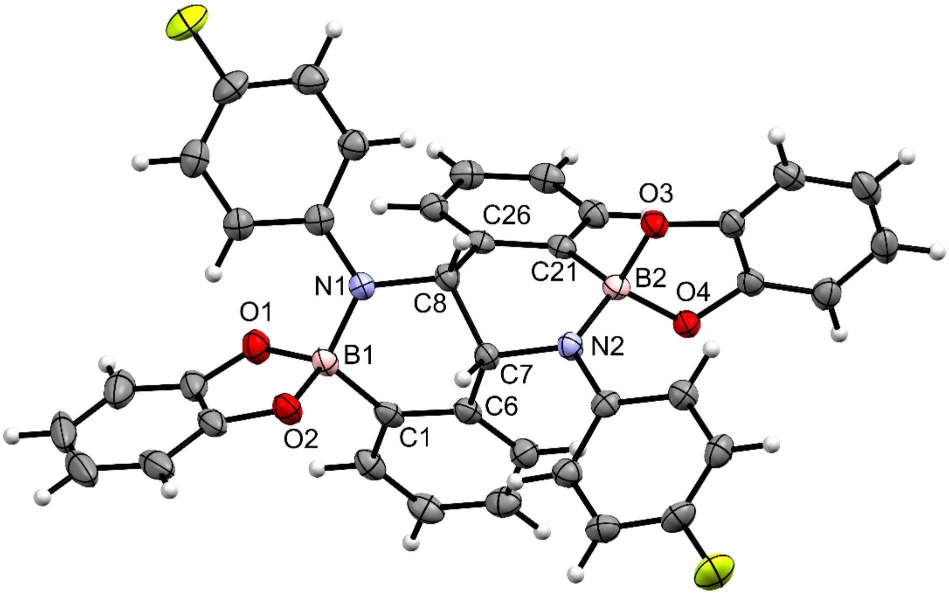

Figure S83. ORTEP diagram of the anionic portion of the structure of ***rac*₆-2a**. Thermal ellipsoids are drawn at the 50% probability level. Selected bond lengths (Å) and angles (°): C(7)-C(8) 1.540(2), N(1)-C(8) 1.473(2), C(8)-C(26) 1.517(2), N(2)-C(7) 1.467(2), C(6)-C(7) 1.518(2), O(1)-B(1) 1.519(2), O(2)-B(1) 1.543(2), B(1)-N(1) 1.532(2), B(1)-C(1) 1.611(3), O(3)-B(2) 1.541(2), O(4)-B(2) 1.523(2), B(2)-N(2) 1.530(2), B(2)-C(21) 1.609(3), N(2)-C(7)-C(6) 114.80(14), N(2)-C(7)-C(8) 110.70(13), C(6)-C(7)-C(8) 108.74(14), N(1)-C(8)-C(26) 115.08(14), N(1)-C(8)-C(7) 110.15(13), C(26)-C(8)-C(7) 109.17(14).

8.3 *rac*$_6$-2b

***rac*$_6$-2b**·2MeCN: Formula $C_{64}H_{58}B_2Co_2N_4O_4$, *M* 1086.62, Monoclinic, space group C 2/c (#15), *a* 25.3705(9), *b* 11.5408(4), *c* 18.8690(7) Å, β 106.319(2), *V* 5302.2(3) Å^3, D_c 1.361 g cm^{-3}, *Z* 4, crystal size 0.280 by 0.180 by 0.050 mm, colour orange, habit plate, temperature 180(2) Kelvin, λ(CuKα) 1.54178 Å, μ(CuKα) 5.325 mm^{-1}, T(SADABS)$_{min,max}$ 0.3965, 0.7531, $2\theta_{max}$ 136.48, *hkl* range -30 30, -13 12, -22 22, *N* 24235, N_{ind} 4784(R_{merge} 0.0549), N_{obs} 3888(I > 2σ(I)), N_{var} 464, residuals* $R1(F)$ 0.0779, $wR2(F^2)$ 0.2034, GoF(all) 1.059, $\Delta\rho_{min,max}$ -0.306, 1.006 e$^-$ Å^{-3}.

*$R1 = \Sigma||F_o| - |F_c||/\Sigma|F_o|$ for $F_o > 2\sigma(F_o)$; $wR2 = (\Sigma w(F_o^2 - F_c^2)^2/\Sigma(wF_c^2)^2)^{1/2}$ all reflections

$w=1/[\sigma^2(F_o^2)+(0.0892P)^2+25.6124P]$ where $P=(F_o^2+2F_c^2)/3$

Crystals were obtained from the unperturbed reaction of **1b** (5.03 mg, 0.016 mmol) and Cp_2Co (3.24 mg, 0.017 mmol) in 0.5 mL of CD_3CN in a J Young NMR tube.

The catechol unit was modelled as disordered over two locations with similarity restraints (SAME) employed to the ensure similar bond lengths and angles between the two parts. One cyclopentadienyl ring of the Cp_2Co^+ counterion was also modelled as disordered over two locations.

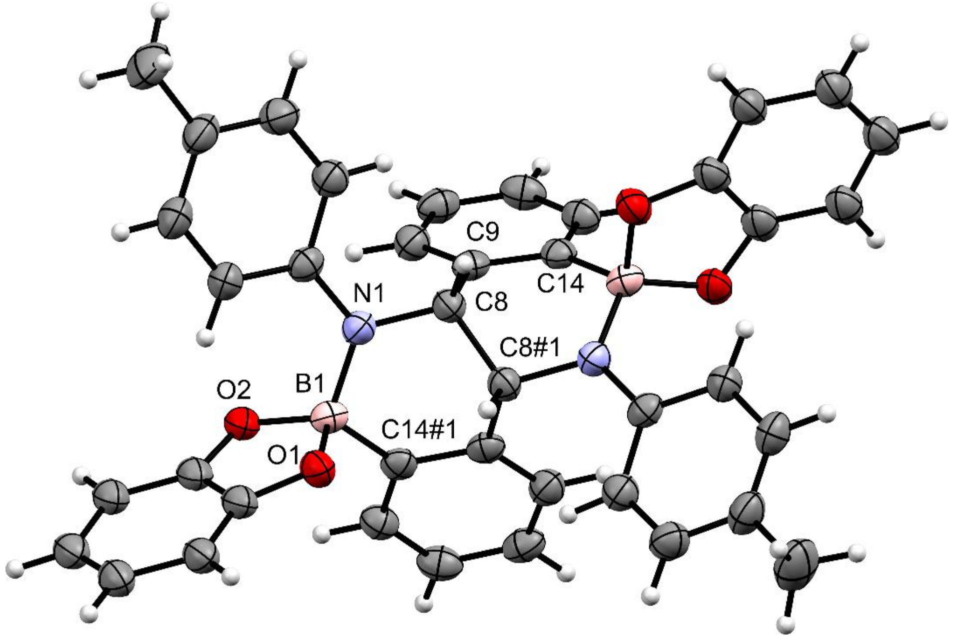

Figure S84. ORTEP diagram of the anionic portion of the structure of ***rac*₆-2b**. Only the major occupancy location of the disordered catechol unit is shown. Thermal ellipsoids are drawn at the 50% probability level. Selected bond lengths (Å) and angles (º): C(8)-C(9) 1.507(6), C(8)-C(8)#1 1.533(8), N(1)-C(8) 1.483(5), B(1)-N(1) 1.530(6), B(1)-O(2) 1.533(12), B(1)-O(1) 1.537(13), B(1)-C(14)#1 1.613(6), N(1)-C(8)-C(9) 115.3(3), N(1)-C(8)-C(8)#1 110.0(4), C(9)-C(8)-C(8)#1 109.4(3). Symmetry transformations used to generate equivalent atoms: #1 -x+1,y,-z+1/2.

8.4 *rac*$_6$-2c

***rac*$_6$-2c**·6MeCN**:** Formula $C_{78}H_{82}B_2Co_2N_8O_4$, *M* 1334.99, Monoclinic, space group C 2/c (#15), *a* 36.1527(15), *b* 11.2705(4), *c* 19.1133(8) Å, β 116.046(2), *V* 6997.0(5) Å^3, D_c 1.267 g cm^{-3}, *Z* 4, crystal size 0.340 by 0.270 by 0.070 mm, colour orange, habit plate, temperature 180(2) Kelvin, λ(CuKα) 1.54178 Å, μ(CuKα) 4.147 mm^{-1}, T(SADABS)$_{min,max}$ 0.5043, 0.7531, $2\theta_{max}$ 136.66, *hkl* range -42 42, -13 13, -20 22, *N* 23586, N_{ind} 6155(R_{merge} 0.0337), N_{obs} 5245(I > 2σ(I)), N_{var} 580, residuals* $R1(F)$ 0.0511, $wR2(F^2)$ 0.1558, GoF(all) 1.033, $\Delta\rho_{min,max}$ -0.227, 0.463 e$^-$ Å^{-3}.

*$R1 = \Sigma||F_o| - |F_c||/\Sigma|F_o|$ for $F_o > 2\sigma(F_o)$; $wR2 = (\Sigma w(F_o^2 - F_c^2)^2/\Sigma(wF_c^2)^2)^{1/2}$ all reflections

$w=1/[\sigma^2(F_o^2)+(0.0930P)^2+8.6600P]$ where $P=(F_o^2+2F_c^2)/3$

Crystals were obtained from the unperturbed reaction of **1c** (6.46 mg, 0.016 mmol) and Cp_2Co (3.09 mg, 0.016 mmol) in 0.5 mL of CD_3CN in a J Young NMR tube.

The catechol unit was modelled as disordered over two locations with similarity restraints (SAME) employed to the ensure similar bond lengths and angles between the two parts. The methyl groups of the *tert*-butyl substituent, one cyclopentadienyl ring of the Cp_2Co^+ counterion and two acetonitrile solvent molecules were also modelled as disordered over two locations.

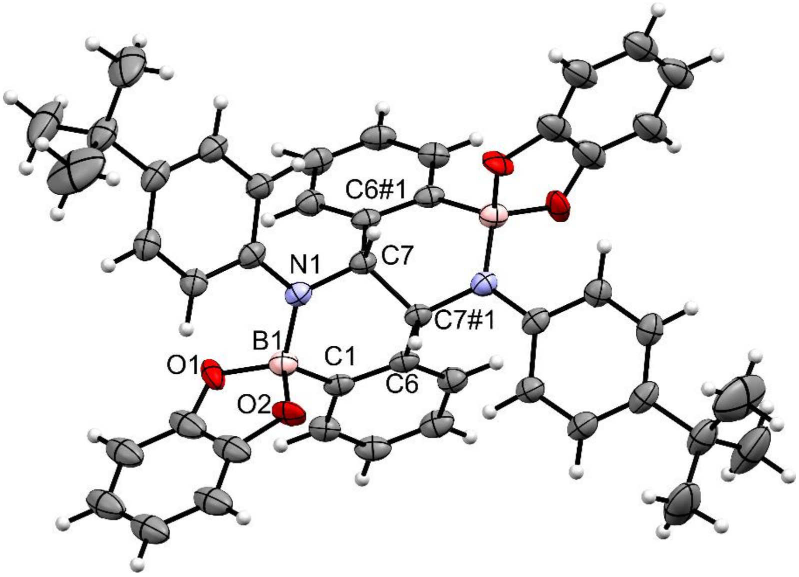

Figure S85. ORTEP diagram of the anionic portion of the structure of ***rac*₆-2c**. Only the major occupancy locations of the disordered catechol and *tert*-butyl units are shown. Thermal ellipsoids are drawn at the 50% probability level. Selected bond lengths (Å) and angles (°): N(1)-C(7) 1.468(3), C(7)-C(6)#1 1.522(3), C(7)-C(7)#1 1.542(4), B(1)-O(2) 1.512(9), B(1)-N(1) 1.535(4), B(1)-O(1) 1.538(10), B(1)-C(1) 1.612(4), N(1)-C(7)-C(6)#1 115.74(19), N(1)-C(7)-C(7)#1 110.6(2), C(6)#1-C(7)-C(7)#1 109.06(16). Symmetry transformations used to generate equivalent atoms: #1 -x+1,y,-z+3/2.

8.5 *rac*$_6$-2d

***rac*$_6$-2d**·2MeCN**:** Formula $C_{64}H_{58}B_2Co_2N_4O_6$, *M* 1118.62, Monoclinic, space group C 2/c (#15), *a* 25.4168(8), *b* 11.4331(4), *c* 19.1422(6) Å, β 107.611(2), *V* 5301.9(3) Å^3, D_c 1.401 g cm^{-3}, *Z* 4, crystal size 0.275 by 0.249 by 0.032 mm, colour orange, habit plate, temperature 180(2) Kelvin, λ(CuKα) 1.54178 Å, μ(CuKα) 5.371 mm^{-1}, *T*(SADABS)$_{min,max}$ 0.4452, 0.7528, 2θ_{max} 133.60, *hkl* range -30 30, -13 13, -22 22, *N* 21689, N_{ind} 4656(R_{merge} 0.0561), N_{obs} 3654(I > 2σ(I)), N_{var} 354, residuals* *R*1(*F*) 0.0739, *wR*2(F^2) 0.2052, GoF(all) 1.041, $\Delta\rho_{min,max}$ -0.259, 1.200 e$^-$ Å^{-3}.

*$R1 = \Sigma||F_o| - |F_c||/\Sigma|F_o|$ for $F_o > 2\sigma(F_o)$; $wR2 = (\Sigma w(F_o^2 - F_c^2)^2/\Sigma(wF_c^2)^2)^{1/2}$ all reflections

$w=1/[\sigma^2(F_o^2)+(0.1165P)^2+13.9544P]$ where $P=(F_o^2+2F_c^2)/3$

Crystals were obtained from the unperturbed reaction of **1d** (5.22 mg, 0.016 mmol) and Cp_2Co (3.00 mg, 0.016 mmol) in 0.5 mL of CD_3CN in a J Young NMR tube.

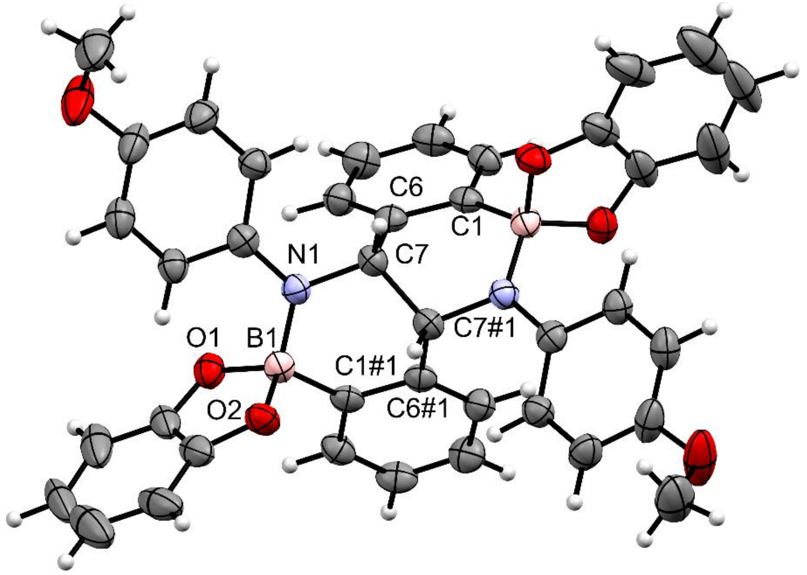

Figure S86. ORTEP diagram of the anionic portion of the structure of ***rac*₆-2d**. Thermal ellipsoids are drawn at the 50% probability level. Selected bond lengths (Å) and angles (°): N(1)-C(7) 1.485(5), C(6)-C(7) 1.517(5), C(7)-C(7)#1 1.545(7), B(1)-O(1) 1.512(5), B(1)-N(1) 1.518(5), B(1)-O(2) 1.544(5), B(1)-C(1)#1 1.614(6), N(1)-C(7)-C(6) 115.0(3), N(1)-C(7)-C(7)#1 110.3(4), C(6)-C(7)-C(7)#1 109.2(2). Symmetry transformations used to generate equivalent atoms: #1 -x+1,y,-z+1/2.

8.6 ***rac*$_5$-2e**

Three different polymorphs of ***rac*$_5$-2e** as the Cp_2Co^+ salt were obtained. The structure of the $Cp*_2Co^+$ analogue was also determined. Data for all four structures is given below.

***rac*$_5$-2e**·2MeCN**:** Formula $C_{64}H_{50}B_2Cl_8Co_2N_4O_4$, *M* 1362.16, Triclinic, space group P -1 (#2), *a* 10.7676(3), *b* 16.3128(5), *c* 17.9339(5) Å, α 81.3170(10), β 75.2360(10), γ 84.6720(10)°, *V* 3006.29(15) Å^3, D_c 1.505 g cm^{-3}, *Z* 2, crystal size 0.190 by 0.190 by 0.110 mm, colour orange, habit block, temperature 180(2) Kelvin, λ(CuKα) 1.54178 Å, μ(CuKα) 8.016 mm^{-1}, T(SADABS)$_{min,max}$ 0.4857, 0.7531, 2θ_{max} 136.79, *hkl* range -12 12, -19 19, -21 21, *N* 46331, N_{ind} 10858(R_{merge} 0.0365), N_{obs} 9920(I > 2σ(I)), N_{var} 761, residuals* *R*1(*F*) 0.0344, *wR*2(F^2) 0.0883, GoF(all) 1.054, $\Delta\rho_{min,max}$ -0.385, 0.592 e$^-$ Å^{-3}.

$^*R1 = \Sigma||F_o| - |F_c||/\Sigma|F_o|$ for $F_o > 2\sigma(F_o)$; $wR2 = (\Sigma w(F_o^2 - F_c^2)^2/\Sigma(wF_c^2)^2)^{1/2}$ all reflections

$w=1/[\sigma^2(F_o^2)+(0.0357P)^2+2.5453P]$ where $P=(F_o^2+2F_c^2)/3$

Crystals were obtained from the unperturbed reaction of **1e** (6.10 mg, 0.013 mmol) and Cp_2Co (2.52 mg, 0.013 mmol) in 0.5 mL of CD_3CN in a J Young NMR tube.

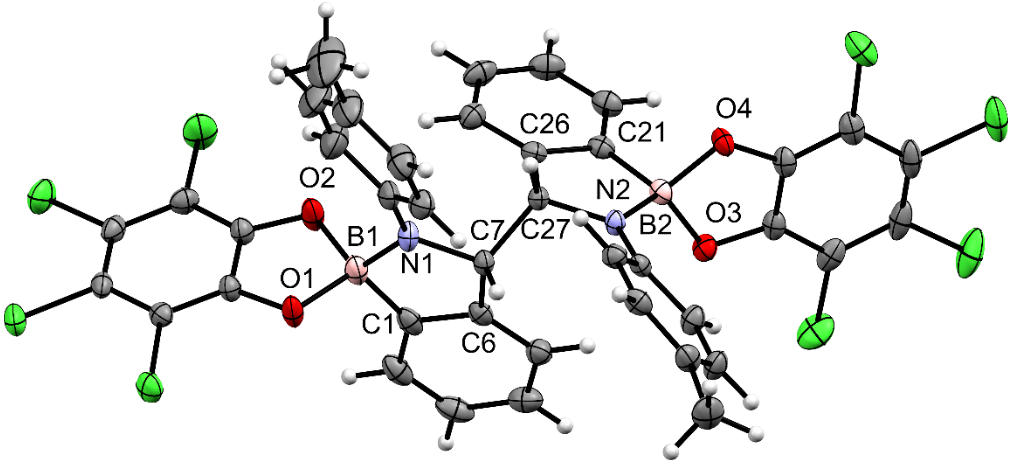

Figure S87. ORTEP diagram of the anionic portion of the structure of ***rac*₅-2e** from the triclinic polymorph. Thermal ellipsoids are drawn at the 50% probability level. Selected bond lengths (Å) and angles (º): C(7)-C(27) 1.543(3), N(1)-C(7) 1.469(2), C(6)-C(7) 1.515(3), N(2)-C(27) 1.468(2), C(26)-C(27) 1.518(3), B(1)-N(1) 1.497(3), B(1)-O(2) 1.548(3), B(1)-O(1) 1.549(3), B(1)-C(1) 1.593(3), B(2)-N(2) 1.505(3), B(2)-O(3) 1.531(3), B(2)-O(4) 1.545(3), B(2)-C(21) 1.591(3), N(1)-C(7)-C(6) 104.06(16), N(1)-C(7)-C(27) 111.51(16), C(6)-C(7)-C(27) 116.04(16), N(2)-C(27)-C(26) 103.79(15), N(2)-C(27)-C(7) 112.56(16), C(26)-C(27)-C(7) 115.94(17).

***rac*$_5$-2e·**2MeCN**:** Formula $C_{64}H_{50}B_2Cl_8Co_2N_4O_4$, *M* 1362.16, Monoclinic, space group C 2/c (#15), *a* 22.5288(8), *b* 14.5012(5), *c* 19.0824(7) Å, β 101.451(2), *V* 6110.0(4) Å^3, D_c 1.481 g cm^{-3}, *Z* 4, crystal size 0.160 by 0.130 by 0.080 mm, colour orange, habit block, temperature 180(2) Kelvin, λ(CuKα) 1.54178 Å, μ(CuKα) 7.888 mm^{-1}, $T(\text{SADABS})_{\text{min,max}}$ 0.5672, 0.7528, $2\theta_{max}$ 133.29, *hkl* range -26 26, -13 17, -20 21, *N* 21158, N_{ind} 5144(R_{merge} 0.0363), N_{obs} 4511(I > 2σ(I)), N_{var} 381, residuals* $R1(F)$ 0.0422, $wR2(F^2)$ 0.1075, GoF(all) 1.031, $\Delta\rho_{min,max}$ -0.815, 0.680 e$^-$ Å^{-3}.

*$R1 = \Sigma||F_o| - |F_c||/\Sigma|F_o|$ for $F_o > 2\sigma(F_o)$; $wR2 = (\Sigma w(F_o^2 - F_c^2)^2/\Sigma(wF_c^2)^2)^{1/2}$ all reflections

$w=1/[\sigma^2(F_o^2)+(0.0432P)^2+14.5907P]$ where $P=(F_o^2+2F_c^2)/3$

Crystals were obtained from the unperturbed reaction of **1e** (12.6 mg, 0.027 mmol) and Cp_2Co (5.1 mg, 0.026 mmol) in 0.5 mL of CD_3CN in a J Young NMR tube.

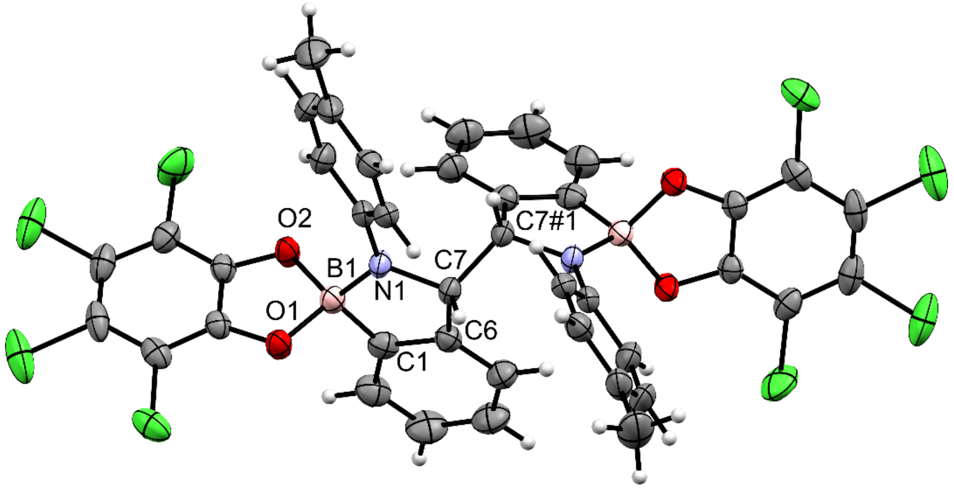

Figure S88. ORTEP diagram of the anionic portion of the structure of ***rac*₅-2e** from the monoclinic C 2/c polymorph. Thermal ellipsoids are drawn at the 50% probability level. Selected bond lengths (Å) and angles (°): N(1)-C(7) 1.472(3), C(6)-C(7) 1.518(4), C(7)-C(7)#1 1.540(5), B(1)-N(1) 1.500(4), B(1)-O(2) 1.524(3), B(1)-O(1) 1.561(4), B(1)-C(1) 1.586(4), N(1)-C(8) 1.410(3), N(1)-C(7)-C(6) 103.3(2), N(1)-C(7)-C(7)#1 112.4(3), C(6)-C(7)-C(7)#1 114.84(17). Symmetry transformations used to generate equivalent atoms: #1 -x+1,y,-z+1/2.

***rac*$_5$-2e·**2MeCN**:** Formula $C_{64}H_{50}B_2Cl_8Co_2N_4O_4$, *M* 1362.16, Monoclinic, space group C c (#9), *a* 32.6064(17), *b* 11.4712(6), *c* 18.9531(10) Å, β 120.824(2), *V* 6087.7(6) Å^3, D_c 1.486 g cm^{-3}, *Z* 4, crystal size 0.350 by 0.150 by 0.100 mm, colour orange, habit block, temperature 180(2) Kelvin, λ(CuKα) 1.54178 Å, μ(CuKα) 7.917 mm^{-1}, *T*(SADABS)$_{min,max}$ 0.4340, 0.7529, $2\theta_{max}$ 133.19, *hkl* range -38 38, -13 13, -22 22, *N* 27905, N_{ind} 10293(R_{merge} 0.0359), N_{obs} 9307(I > 2σ(I)), N_{var} 804, residuals* *R*1(*F*) 0.0538, *wR*2(F^2) 0.1336, GoF(all) 1.061, $\Delta\rho_{min,max}$ -0.408, 0.901 e$^-$ Å^{-3}.

*$R1 = \Sigma||F_o| - |F_c||/\Sigma|F_o|$ for $F_o > 2\sigma(F_o)$; $wR2 = (\Sigma w(F_o^2 - F_c^2)^2/\Sigma(wF_c^2)^2)^{1/2}$ all reflections

$w=1/[\sigma^2(F_o^2)+(0.0660P)^2+11.9709P]$ where $P=(F_o^2+2F_c^2)/3$

Crystals were obtained from the unperturbed reaction of **1e** (7.12 mg, 0.016 mmol) and Cp_2Co (12.02 mg, 0.063 mmol) in 0.5 mL of CD_3CN in a J Young NMR tube.

One complete Cp_2Co^+ counterion and one cyclopentadienyl ring of the other Cp_2Co^+ were modelled as disordered over two locations. The structure was refined as a racemic twin with the Flack parameter[7] refining to 0.402(6).

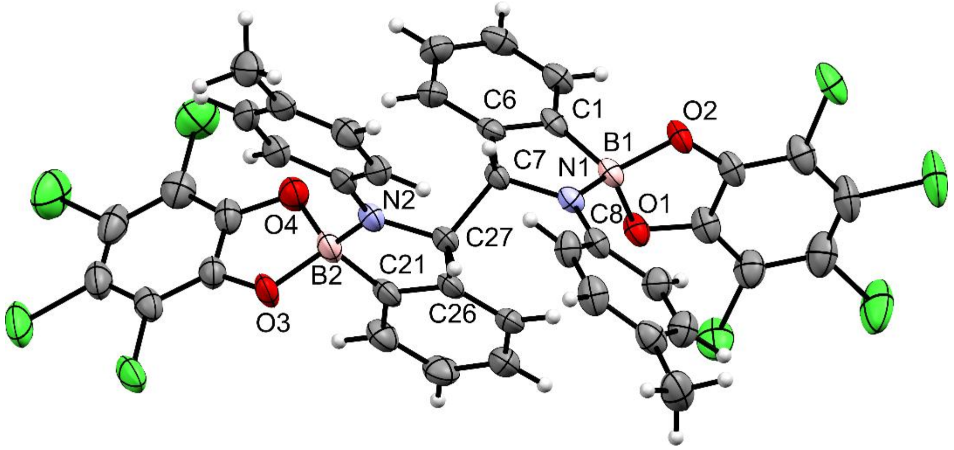

Figure S89. ORTEP diagram of the anionic portion of the structure of ***rac*₅-2e** from the monoclinic C c polymorph. Thermal ellipsoids are drawn at the 50% probability level. Selected bond lengths (Å) and angles (º): C(7)-C(27) 1.567(7), N(1)-C(7) 1.485(7), C(6)-C(7) 1.509(8), N(2)-C(27) 1.482(7), C(26)-C(27) 1.516(9), B(1)-N(1) 1.513(8), B(1)-O(1) 1.528(8), B(1)-O(2) 1.539(7), B(1)-C(1) 1.597(10), B(2)-N(2) 1.513(9), B(2)-O(4) 1.522(9), B(2)-O(3) 1.541(8), B(2)-C(21) 1.591(10), N(1)-C(7)-C(6) 103.8(4), N(1)-C(7)-C(27) 111.9(4), C(6)-C(7)-C(27) 114.2(5), N(2)-C(27)-C(26) 103.6(5), N(2)-C(27)-C(7) 112.6(4), C(26)-C(27)-C(7) 114.6(5).

***rac*$_5$-2e·**2MeCN ($Cp^*{}_2Co^+$ complex)**:** Formula $C_{84}H_{90}B_2Cl_8Co_2N_4O_4$, *M* 1642.67, Triclinic, space group P -1 (#2), *a* 10.4611(8), *b* 11.7854(9), *c* 33.412(3) Å, α 93.034(3), β 94.851(3), γ 105.297(3)º, *V* 3946.9(5) Å^3, D_c 1.382 g cm^{-3}, *Z* 2, crystal size 0.030 by 0.020 by 0.005 mm, colour pale yellow, habit plate, temperature 100(2) Kelvin, λ(synchrotron) 0.6889 Å, μ(synchrotron) 0.669 mm^{-1}, T(SADABS)$_{min,max}$ 0.6443, 0.7454, $2\theta_{max}$ 51.00, *hkl* range -13 13, -14 14, -41 41, *N* 59447, N_{ind} 15920(R_{merge} 0.1200), N_{obs} 9338(I > 2σ(I)), N_{var} 1079, residuals* $R1(F)$ 0.0727, $wR2(F^2)$ 0.1780, GoF(all) 1.027, $\Delta\rho_{min,max}$ -1.193, 1.241 e$^-$ Å^{-3}.

*$R1 = \Sigma||F_o| - |F_c||/\Sigma|F_o|$ for $F_o > 2\sigma(F_o)$; $wR2 = (\Sigma w(F_o^2 - F_c^2)^2/\Sigma(wF_c^2)^2)^{1/2}$ all reflections

$w=1/[\sigma^2(F_o^2)+(0.0364P)^2+10.4085P]$ where $P=(F_o^2+2F_c^2)/3$

Crystals were obtained from the unperturbed reaction of **1e** (5.14 mg, 0.011 mmol) and $Cp^*{}_2Co$ (3.65 mg, 0.011 mmol) in 0.5 mL of CD_3CN in a J Young NMR tube.

The catechol unit, one $Cp^*{}_2Co^+$ counterion and one acetonitrile solvent molecule were modelled as disordered over two locations. As a consequence of this disorder there is one short contact involving a hydrogen atom of the disordered $Cp^*{}_2Co^+$ counterion which was not accurately located.

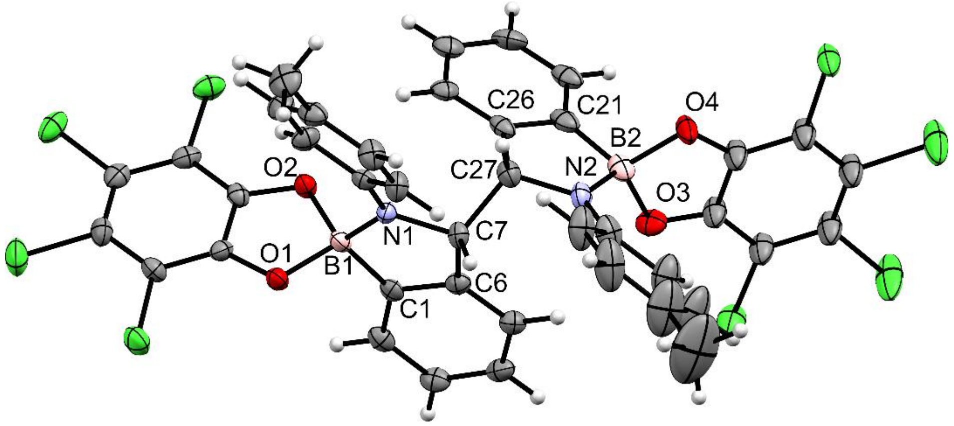

Figure S90. ORTEP diagram of the anionic portion of the structure of the $Cp^*{}_2Co^+$ salt of ***rac*$_5$-2e**. Only the major occupancy location of the disordered catechol unit is shown. Thermal ellipsoids are drawn at the 50% probability level. Selected bond lengths (Å) and angles (º): C(7)-C(27) 1.549(7), N(1)-C(7) 1.471(5), N(2)-C(27) 1.475(6), C(6)-C(7) 1.517(6), C(26)-C(27) 1.520(7), B(1)-N(1) 1.512(6), B(1)-O(1) 1.538(6), B(1)-O(2) 1.550(6), B(1)-C(1) 1.580(7), B(2)-N(2) 1.510(7), B(2)-O(4) 1.534(7), B(2)-O(3) 1.543(7), B(2)-C(21) 1.586(8), N(1)-C(7)-C(6) 104.1(4), N(1)-C(7)-C(27) 112.3(4), C(6)-C(7)-C(27) 117.2(4), N(2)-C(27)-C(26) 103.3(4), N(2)-C(27)-C(7) 112.3(4), C(26)-C(27)-C(7) 115.7(4).

9 Time-Course NMR Studies

9.1 Reductive Coupling of 1c in CD_3CN

In Section 5.2 ***meso*$_5$-2c** was characterised in solution following the crystallisation of ***rac*$_6$-2c** from the reaction mixture. The NMR spectra of the reaction mixture over a period of 7 days show that the peak for ***rac*$_5$-2c** decreases as it is converted to ***rac*$_6$-2c**, which crystallises from the reaction mixture (Figure S91).

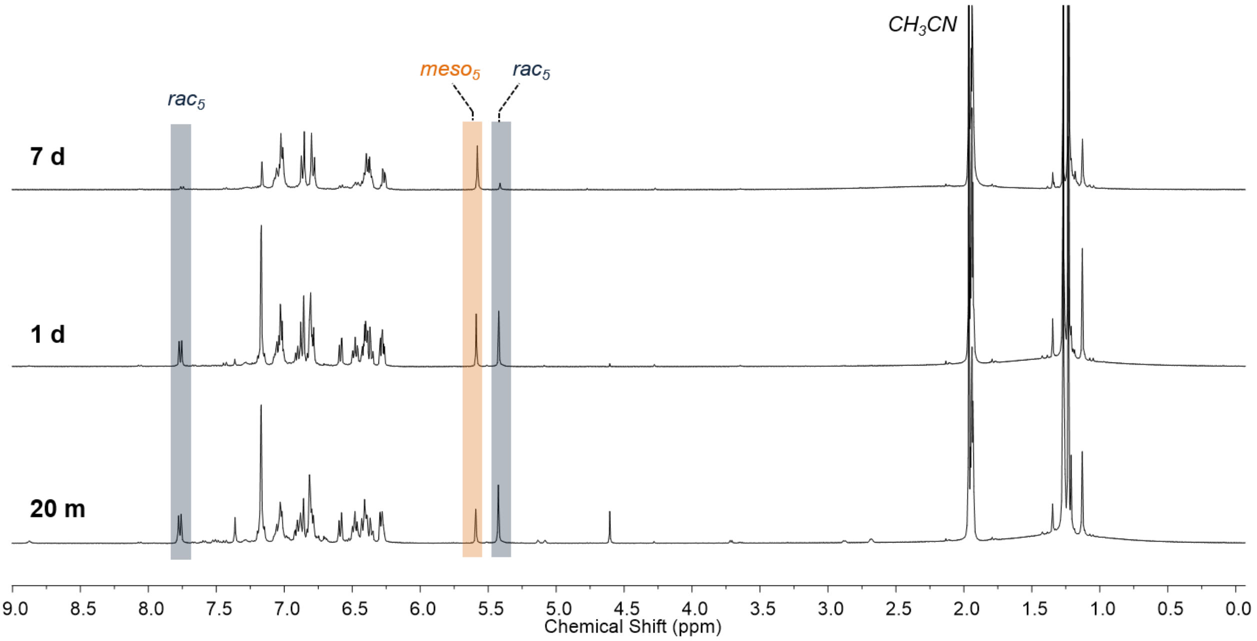

Figure S91. Time-course NMR spectra of the reductive coupling of **1c** in CD_3CN showing the disappearance of the ***rac*$_5$-2c** over time due to its interconversion to the ***rac*$_6$-2c** product, which subsequently crystallised from the reaction mixture.

9.2 Reductive Couplings in DMSO-d_6

In order to investigate the formation of the ***rac*₆-2** isomer in solution, the reductive coupling was monitored by NMR spectroscopy over time in DMSO-d_6. Iminoboronates **1a** and **1d** were chosen as the reactants since they contained the most electron-deficient and electron-rich anilines, respectively. In both cases, ***meso*$_5$-2** and ***rac*$_5$-2** formed initially as the kinetic products but over time ***rac*$_5$-2** converted to ***rac*$_6$-2**, the thermodynamic product (Figures S92, S93).

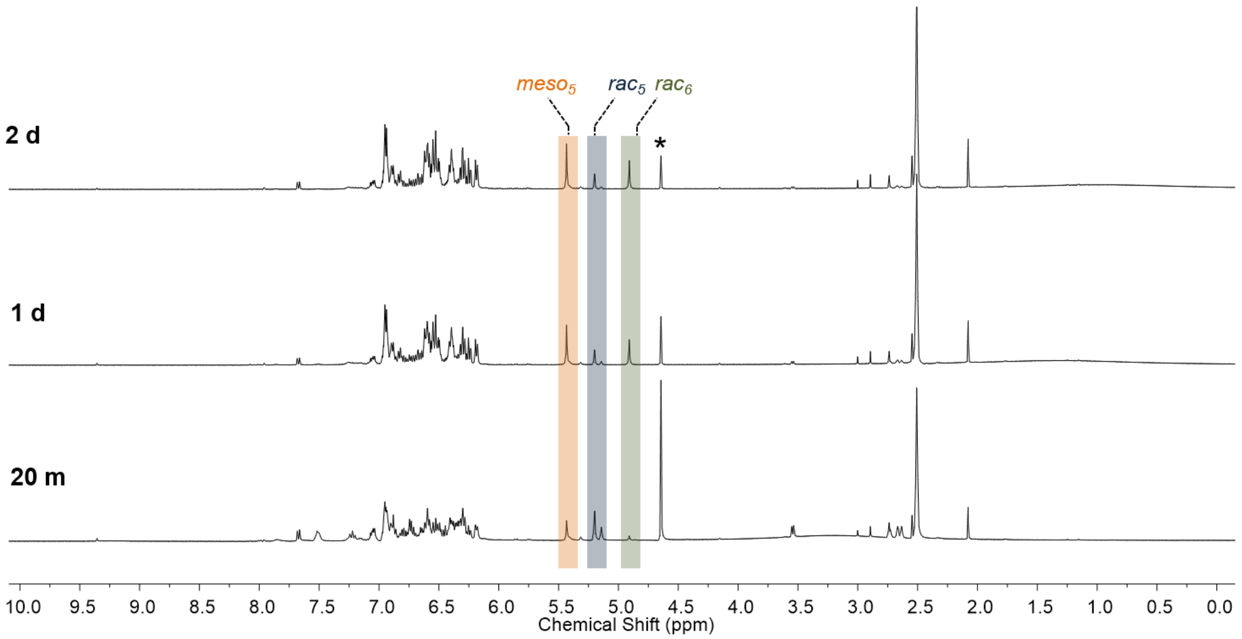

Figure S92. Time-course NMR spectra of the reductive coupling of **1a** in DMSO-d_6 showing the formation of the ***rac*$_5$-2a**, ***rac*$_6$-2a** and ***meso*$_5$-2a** products. * is attributed to a transient Cp_2Co^+ species.

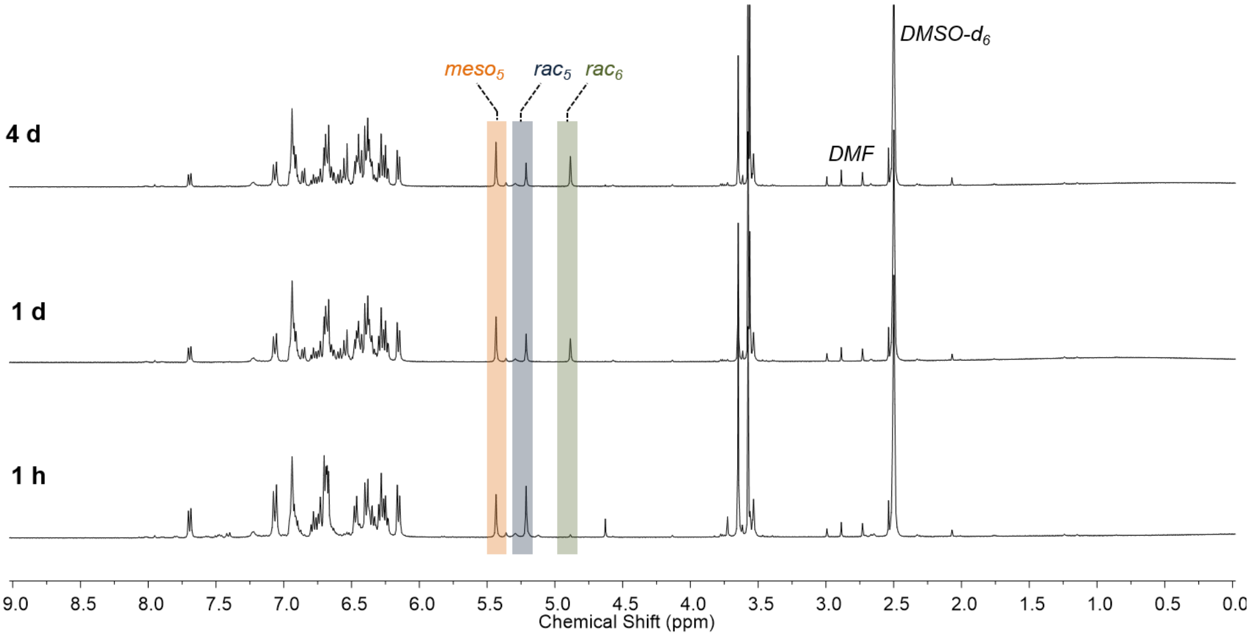

Figure S93. Time-course NMR spectra of the reductive coupling of **1d** in DMSO-d_6 showing the formation of the ***rac*$_5$-2d**, ***rac*$_6$-2d** and ***meso*$_5$-2d** products.

9.3 NMR Studies of Interconversion between the *meso*$_5$-2 and *rac*$_{5/6}$ Isomers

The redissolved ***meso*$_5$-2a** crystals in Section 3.3 were left to equilibrate at room temperature for 6 days and no interconversion between ***meso*$_5$-2a** and the ***rac*$_{5/6}$** isomers was observed during this time (Figure S94). In addition, there was no evidence for the formation of ***meso*$_6$-2a**. In order to probe if heat is required for these interconversions to take place, the sample was heated for 1 day at 90 °C but only decomposition was observed, particularly with prolonged heating for 4 days.

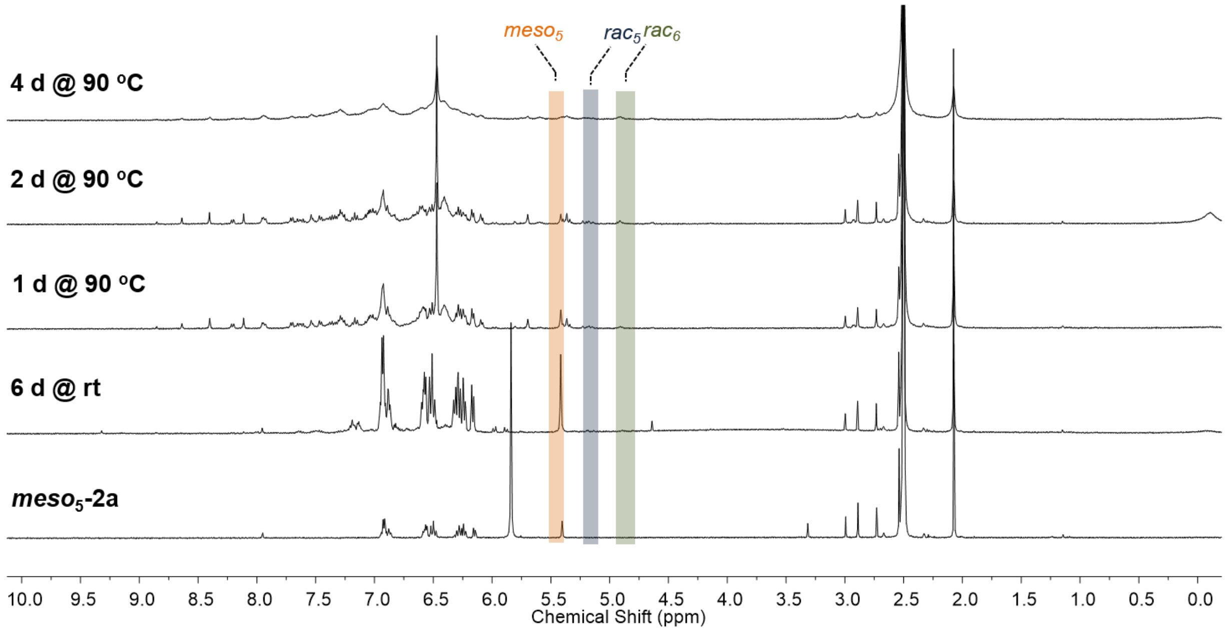

Figure S94. Time-course NMR spectra of redissolved ***meso*$_5$-2a** crystals in DMSO-d_6 upon equilibration at room temperature and heating.

The redissolved ***rac*$_6$-2c** crystals were heated for several hours at 90 °C for 2 h. During this time interconversion to ***rac*$_5$-2c** took place and the two species were stable to heating, however, no interconversion to ***meso*$_5$-2c** was observed (Figure S95). Increasing the temperature to 130 °C led to decomposition of the sample.

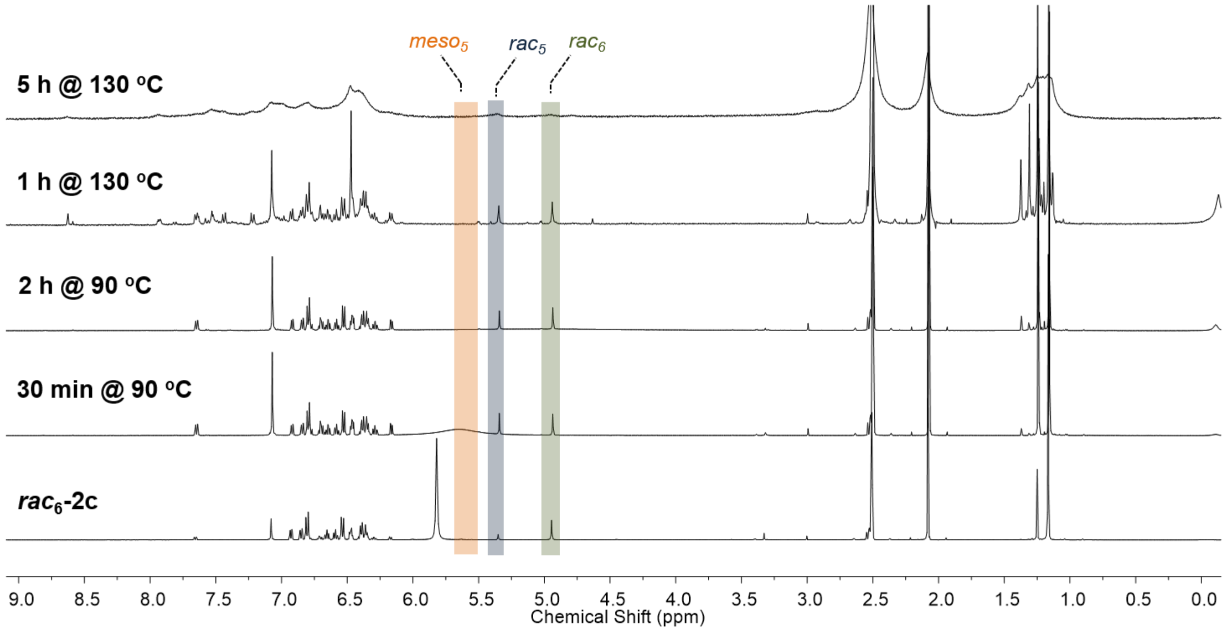

Figure S95. Time-course NMR spectra of redissolved ***rac*$_6$-2c** crystals in DMSO-d_6 upon heating.

10 Time-Course NMR Studies for *Rac*5/*Rac*6 Equilibration

Crystals of ***rac*6-2a-d** were obtained (Section 8) and redissolved in DMSO-d_6 in order to monitor the equilibration between the ***rac*6-2** and ***rac*5-2** isomers over time (Scheme S2, Figures S96-100). Due to difficulties redissolving the crystals and the relatively fast equilibration times, quantitative studies were not possible. However, the studies showed qualitatively that the substituent on the aniline influenced the rate of interconversion; the rate was fastest for ***rac*6-2d** with the most electron-rich substituent and consequently, the initial spectra were a mixture of the two isomers, whereas spectra were predominantly ***rac*6-2a-c** with less electron-rich substituents.

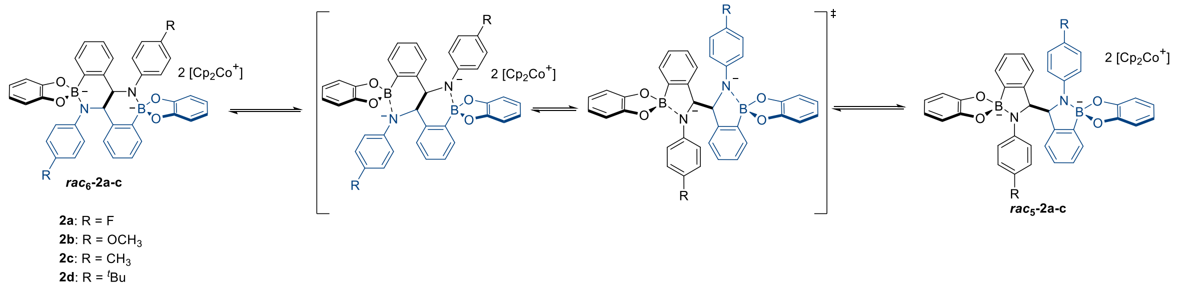

Scheme S2. Interconversion between ***rac*6-2** and ***rac*5-2** isomers and postulated mechanism.

10.1 2a

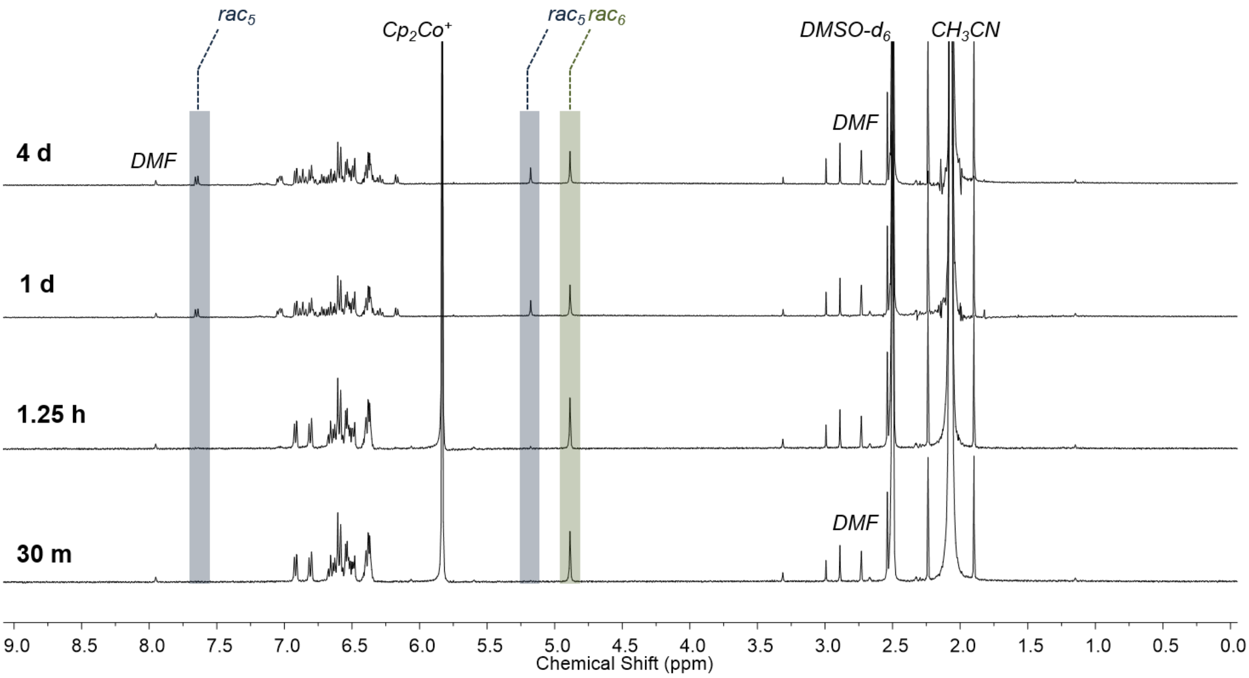

Figure S96. Time-course NMR spectra of redissolved ***rac*6-2a** crystals in DMSO-d_6 showing interconversion to ***rac*5-2a**.

10.2 2b

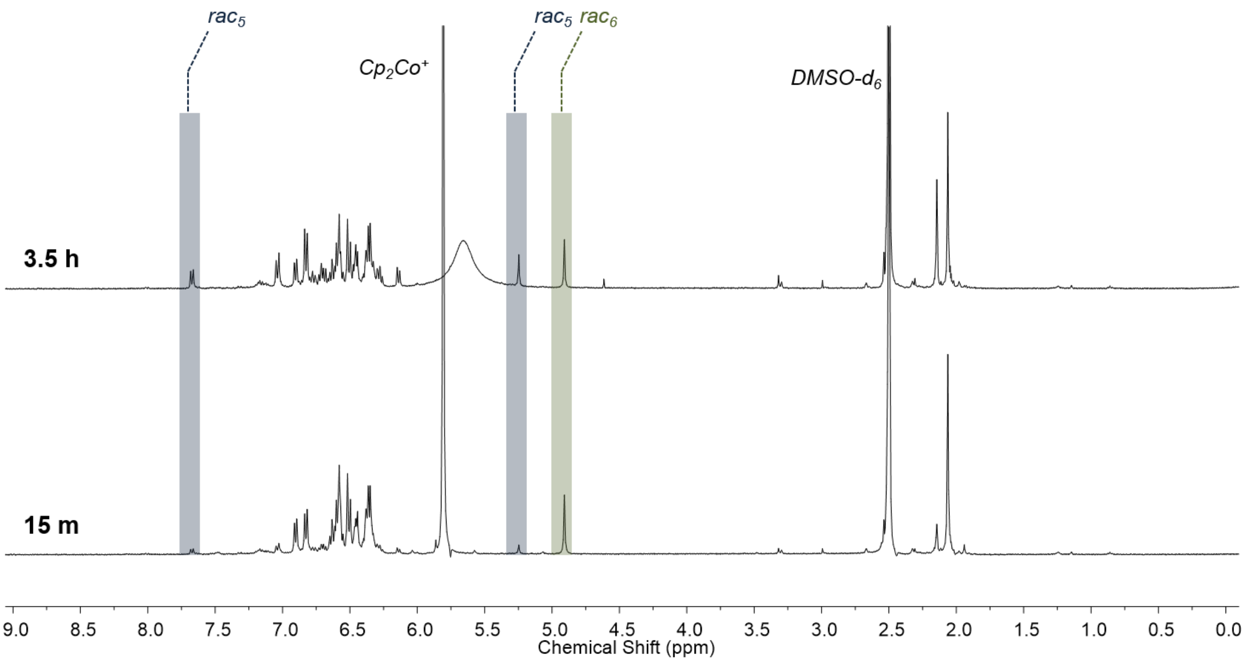

Figure S97. Time-course NMR spectra of redissolved ***rac*$_6$-2b** crystals in DMSO-d_6 showing interconversion to ***rac*$_5$-2b**.

10.3 2c

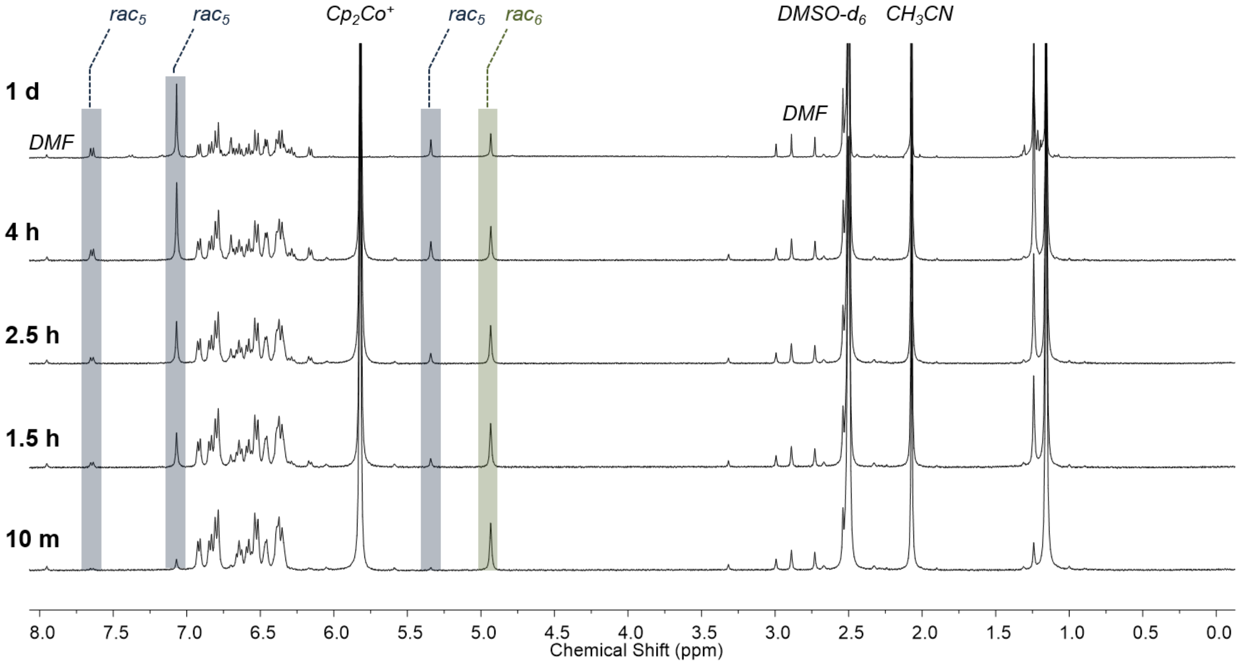

Figure S98. Time-course NMR spectra of redissolved ***rac*$_6$-2c** crystals in DMSO-d_6 showing interconversion to ***rac*$_5$-2c**.

10.4 2d

Crystals obtained from two different reactions were redissolved in DMSO-d_6 and the initial spectrum in both cases was found to contain both ***rac*$_6$-2d** and ***rac*$_5$-2d** (Figures S99 and S100). The appearance of both isomers following immediate dissolution and measurement of the NMR spectrum (15 min, Figure S99) is attributed to the fast interconversion between the two isomers.

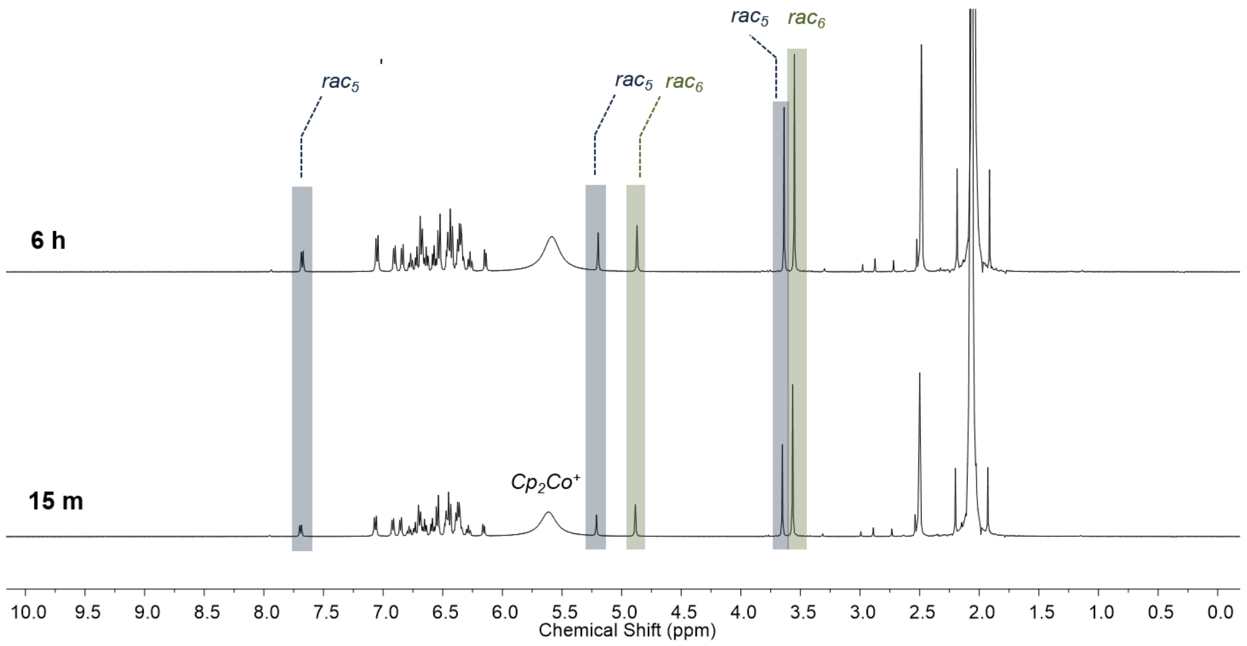

Figure S99. Time-course NMR spectra of redissolved ***rac*$_6$-2d** crystals in DMSO-d_6 showing fast equilibration to ***rac*$_5$-2d**.

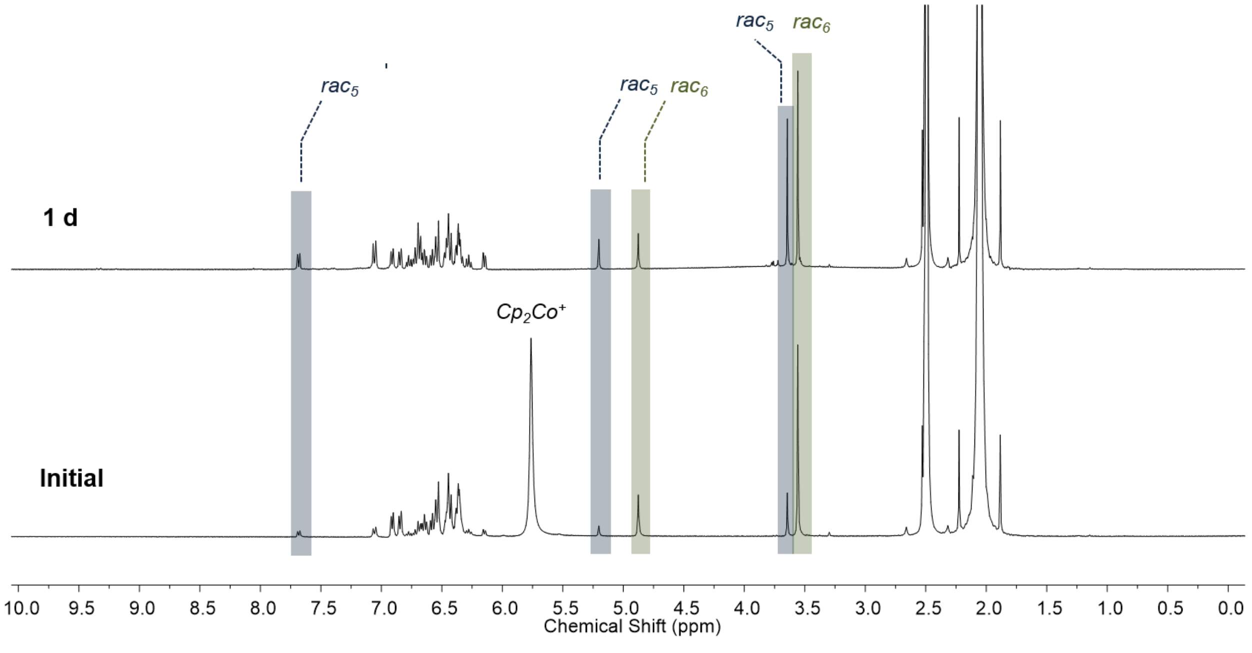

Figure S100. Time-course NMR spectra of redissolved ***rac*$_6$-2d** crystals in DMSO-d_6 showing fast equilibration to ***rac*$_5$-2d**.

10.5 2e

Crystals of ***rac*₅-2e** were also obtained and they were observed to interconvert to ***rac*₆-2e** (Scheme S3, Figure S101).

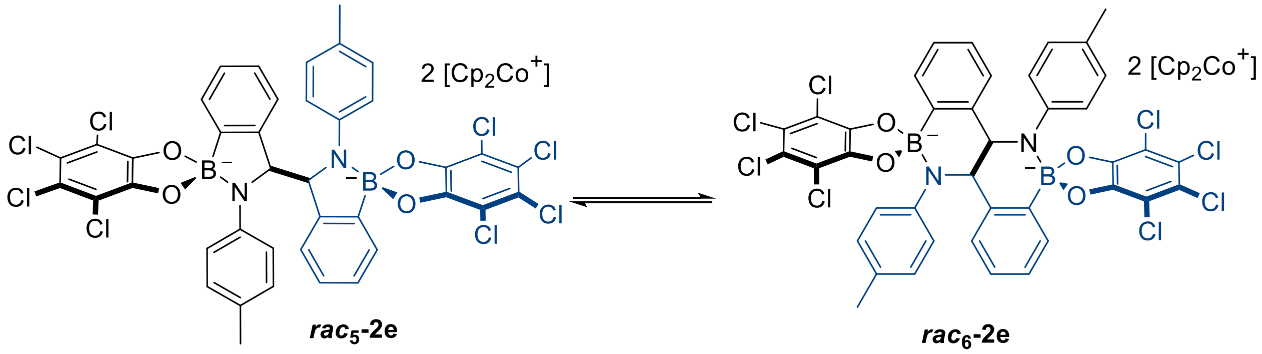

Scheme S3. Interconversion between ***rac*₅-2e** and ***rac*₆-2e** isomers.

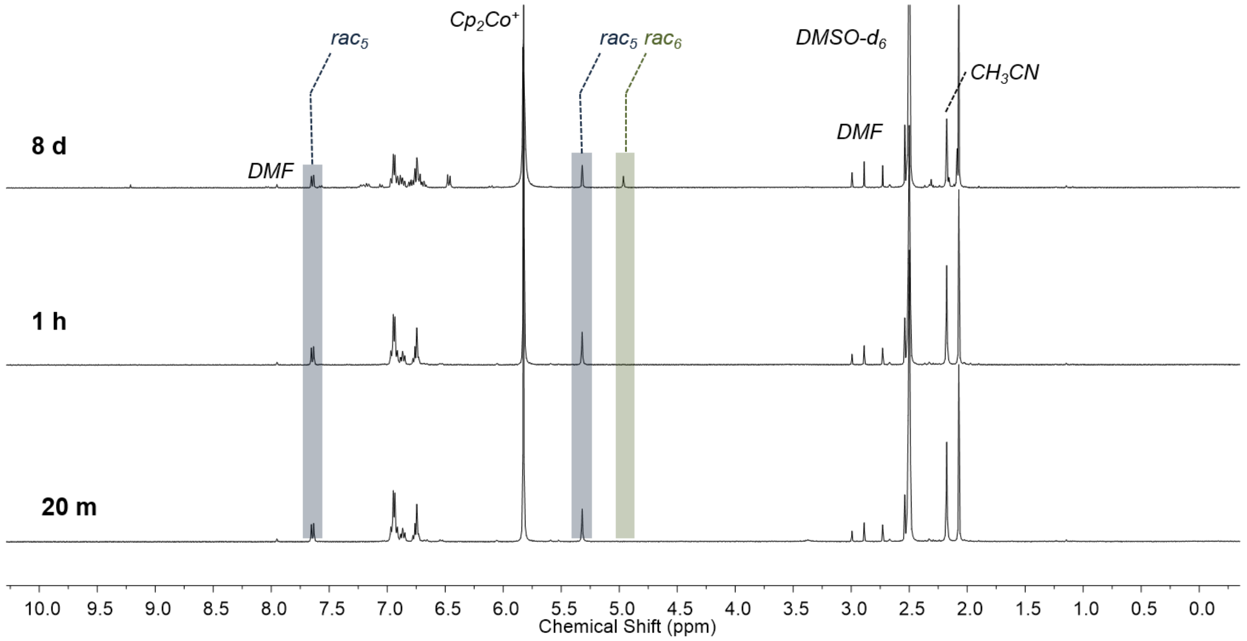

Figure S101. Time-course NMR spectra of redissolved ***rac*₅-2e** crystals in DMSO-d_6 showing interconversion to ***rac*₆-2e**.

10.6 Comparison of Chemical Shifts

In all cases, the redissolved ***rac*₆-2a-c** and ***rac*₅-2e** crystals equilibrated with a second species over time due to B-N bond rearrangements. It was not possible to crystallise this second species from DMSO-d_6 but the second species could be identified based on the methine chemical shift; a comparison of the equilibrated mixtures revealed that the methine chemical shift of the ***rac*₆-2** and ***rac*₅-2** isomers did not vary significantly with substitution of the catechol and amine subcomponents (Figure S102).

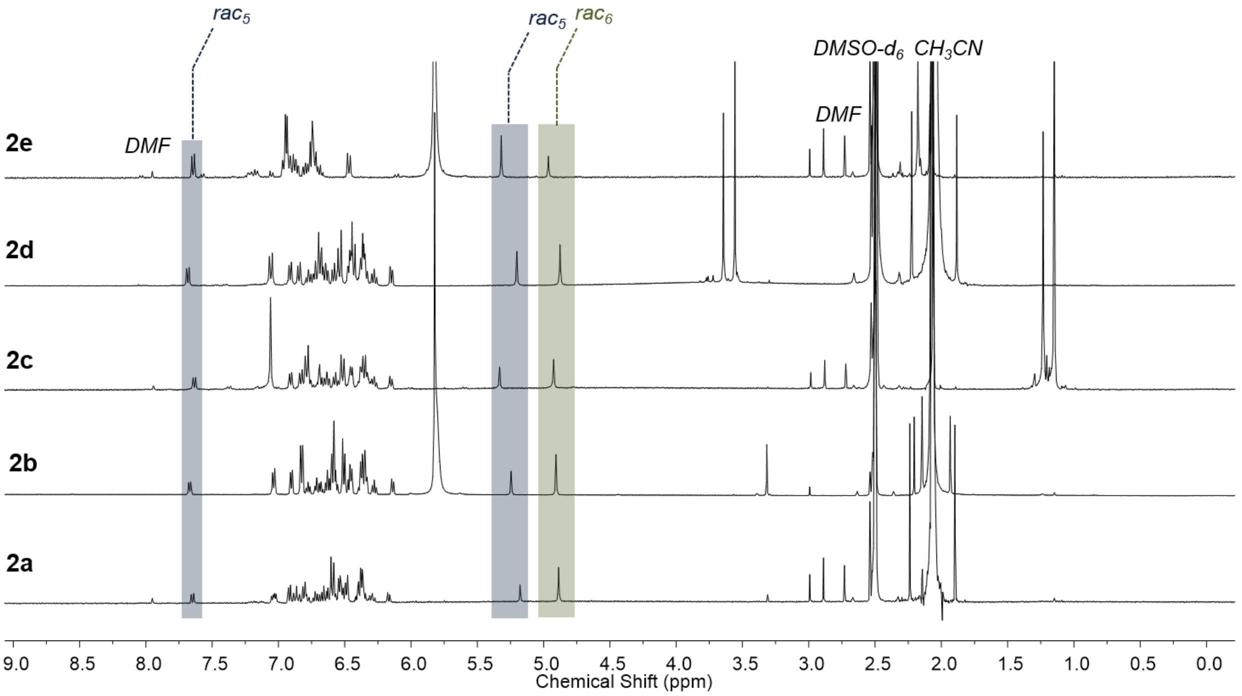

Figure S102. Stacked NMR spectra of the equilibrated mixtures of the ***rac*₅-2a-e** and ***rac*₆-2a-e** in DMSO-d_6 showing that the methine chemical shift of ***rac*₅-2** and ***rac*₆-2** does not vary significantly with substitution of the amine or catechol subcomponents.

11 Reversible Radical Coupling with Ph_3CBF_4

While interconversion between the ***meso*₅-2** and ***rac*₅/₆-2** was not observed under the tested conditions (Section 9.3), the reaction of the reductively coupled product mixture with the tritylium cation was investigated. The reaction mixtures of **2b** and **2e** were chosen to study as their ^{1}H NMR spectra in CD_3CN were well-defined with dispersed signals and competing crystallisation was slow compared to the timeframe of the experiment.

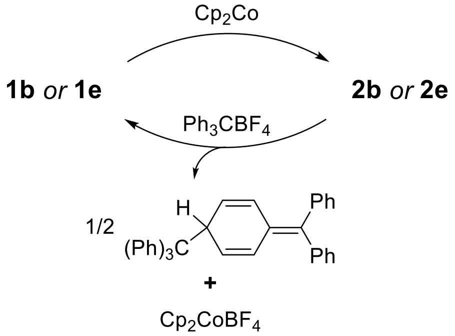

Scheme S4. Reductive coupling of iminoboronates **1b** and **1e** and subsequent tritylium-induced oxidative decoupling of dimers **2b** and **2e.**

11.1 2b

2b was obtained from the reaction of **1b** (5.03 mg, 0.016 mmol) and Cp_2Co (3.02 mg, 0.016 mmol) in 0.5 mL of CD_3CN in a J Young NMR tube. Ph_3CBF_4 (5.80 mg, 0.018 mmol) was added to this solution of **2b** in CD_3CN in a J Young NMR tube under an inert atmosphere at room temperature. After 10 minutes the reaction had gone to completion as indicated by the ^1H NMR spectrum (Figure S103).

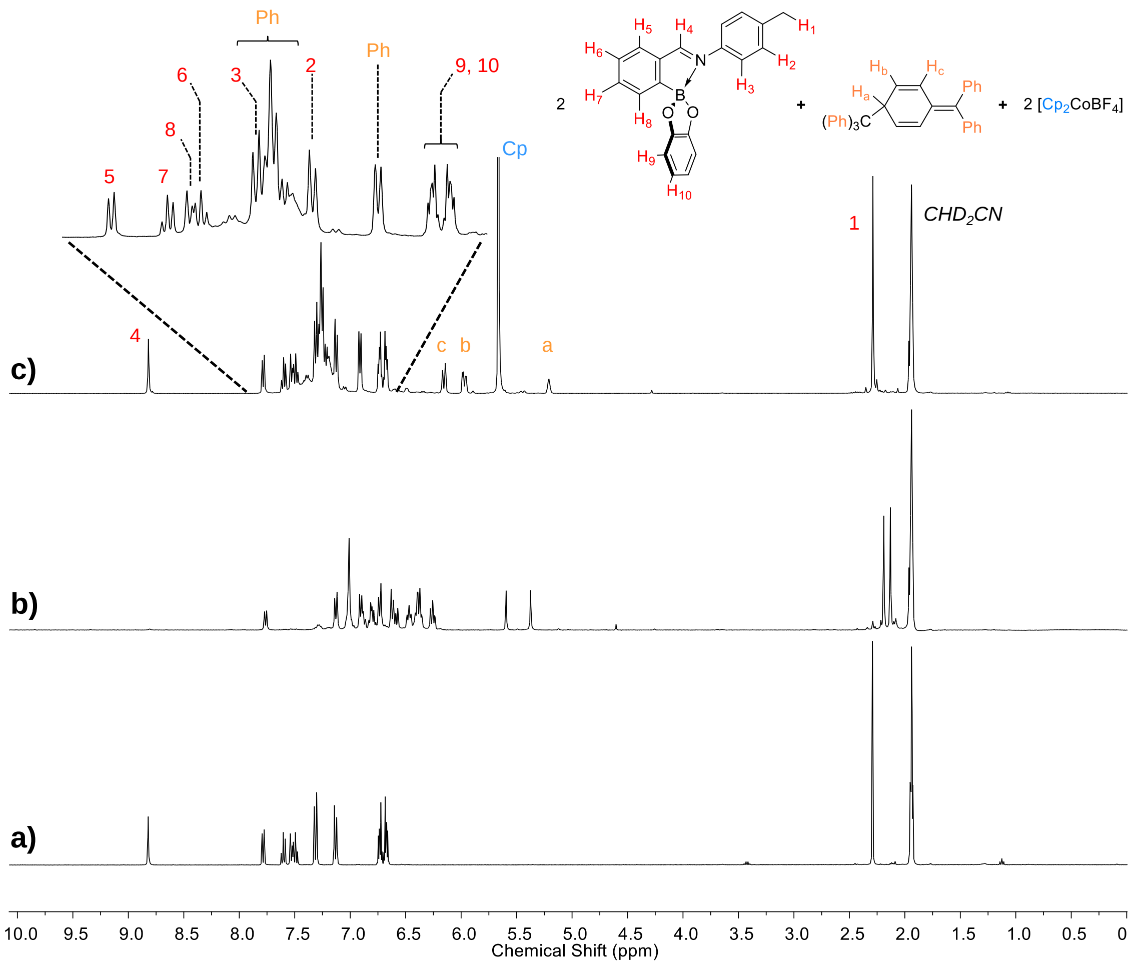

Figure S103. ^1H NMR spectra (400 MHz, CD_3CN) of the reaction of **2b** with Ph_3CBF_4. a) spectrum of **1b**, b) spectrum of **2b**, c) reaction mixture containing **2b** (red), trityl dimer (yellow), and Cp_2CoBF_4 (blue).

11.2 2e

2e was obtained from the reaction of **1e** (5.00 mg, 0.011 mmol) and Cp_2Co (2.10 mg, 0.011 mmol) in 0.5 mL of CD_3CN in a J Young NMR tube. Ph_3CBF_4 (4.03 mg, 0.012 mmol) was added to this solution of **2e** in CD_3CN in a J Young NMR tube under an inert atmosphere at room temperature. After 10 minutes the reaction had gone to completion as indicated by the ^1H NMR spectrum (Figure S104).

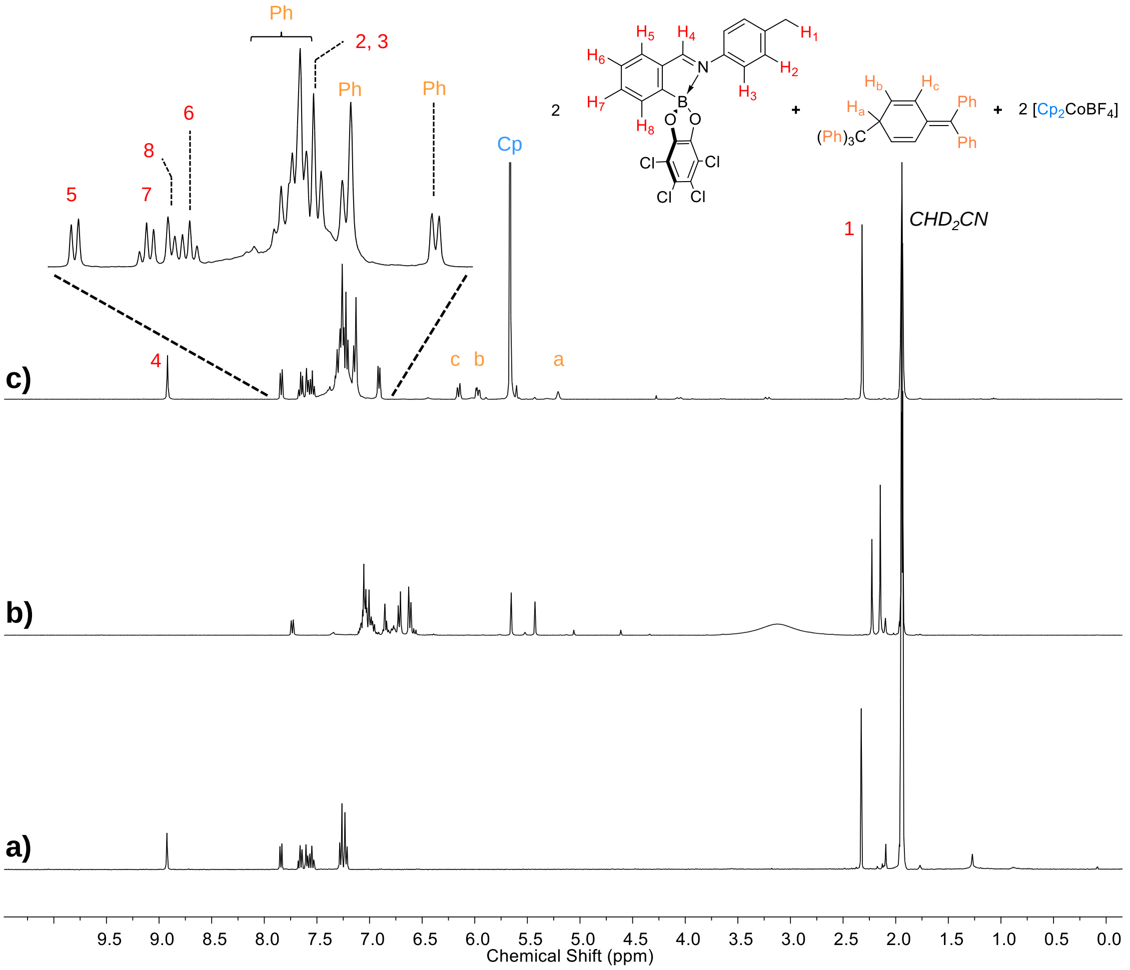

Figure S104. ^{1}H NMR spectra (400 MHz, CD_3CN) of the reaction of **2e** with Ph_3CBF_4. a) spectrum of **1e**, b) spectrum of **2e**, c) reaction mixture containing **2e** (red), trityl dimer (yellow), and Cp_2CoBF_4 (blue).

12 Reaction of 2e with TEMPO

2e was obtained from the reaction of **1e** (5.10 mg, 0.011 mmol) and Cp_2Co (2.10 mg, 0.011 mmol) in 0.5 mL of CD_3CN in a J Young NMR tube. TEMPO (1.74 mg, 0.011 mmol) was added to this solution of **2e** in CD_3CN in a J Young NMR tube under an inert atmosphere, and the reaction was heated at 70 °C for 3 days.

The reaction of **2e** with TEMPO was investigated to further probe the reaction of the reductively coupled products with radicals (Scheme S5). The ^{1}H NMR spectrum of the reaction mixture showed a new species (blue triangles) with a signal above 9 ppm had formed, in addition to the unreacted **2e** (red triangles, Figure S105). Crystals obtained from the reaction revealed the product was **3** by X-ray crystallography (Figure S106).

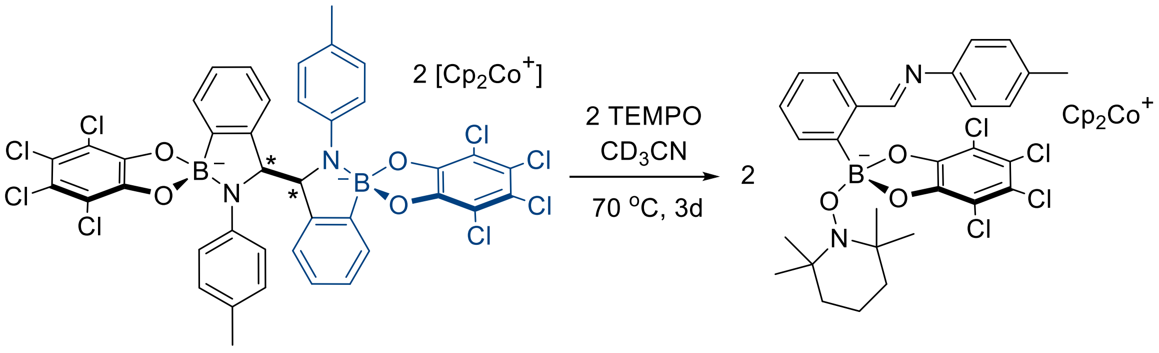

2e
Mixture of isomers

3

Scheme S5. Reaction of **2e** with TEMPO forming **3**.

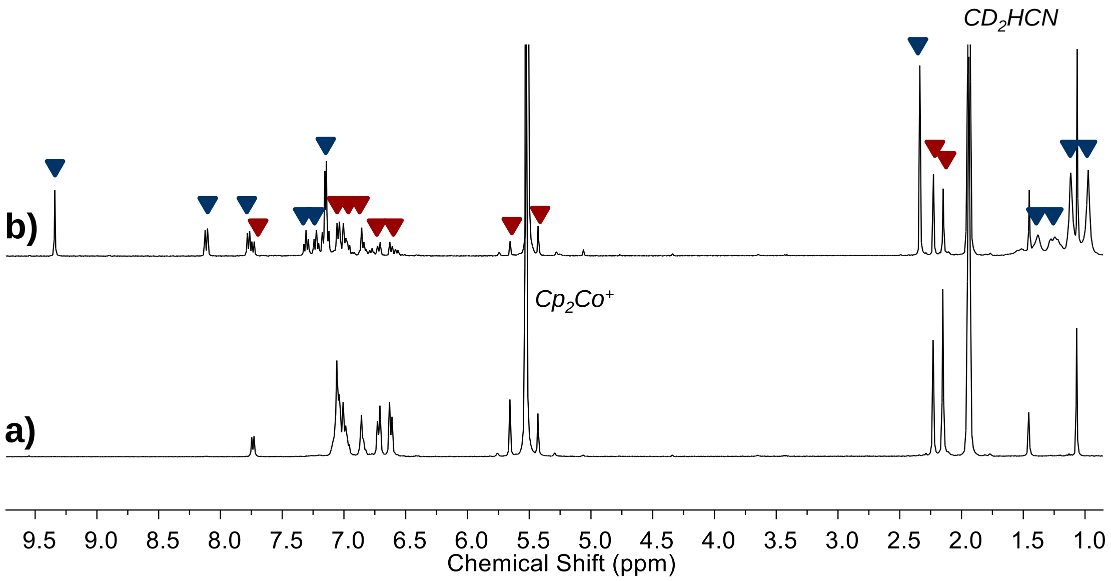

Figure S105. ^{1}H NMR spectra (400 MHz, CD$_3$CN) of the reaction of **2e** with TEMPO: a) spectrum of **2e** 5 mins after the addition of TEMPO and b) spectrum of the reaction mixture containing **2e** (red triangles) and **3** (blue triangles) after 3 days at 70 °C (numerous crystals of **3** were observed as part of the mixture).

12.1 X-Ray Structure of 3

3: Formula $C_{39}H_{40}BCl_4CoN_2O_3$, *M* 796.27, Monoclinic, space group P 21/n (#14), *a* 9.1792(3), *b* 22.4748(7), *c* 18.0279(5) Å, β 93.5580(10), *V* 3712.0(2) Å^3, D_c 1.425 g cm^{-3}, *Z* 4, crystal size 0.490 by 0.120 by 0.060 mm, colour pale yellow, habit block, temperature 180(2) Kelvin, λ(CuKα) 1.54178 Å, μ(CuKα) 6.594 mm^{-1}, T(SADABS)$_{min,max}$ 0.3686, 0.7531, 2θ_{max} 136.76, *hkl* range -10 11, -27 27, -21 21, *N* 35713, N_{ind} 6637(R_{merge} 0.0411), N_{obs} 5923(I > 2σ(I)), N_{var} 456, residuals* *R*1(*F*) 0.0408, *wR*2(F^2) 0.0893, GoF(all) 1.106, $\Delta\rho_{min,max}$ -0.345, 0.280 e$^-$ Å^{-3}.

*$R1 = \Sigma||F_o| - |F_c||/\Sigma|F_o|$ for $F_o > 2\sigma(F_o)$; $wR2 = (\Sigma w(F_o^2 - F_c^2)^2/\Sigma(wF_c^2)^2)^{1/2}$ all reflections

$w=1/[\sigma^2(F_o^2)+6.1808P]$ where $P=(F_o^2+2F_c^2)/3$

Crystals of **3** were obtained upon cooling to room temperature the reaction mixture described above.

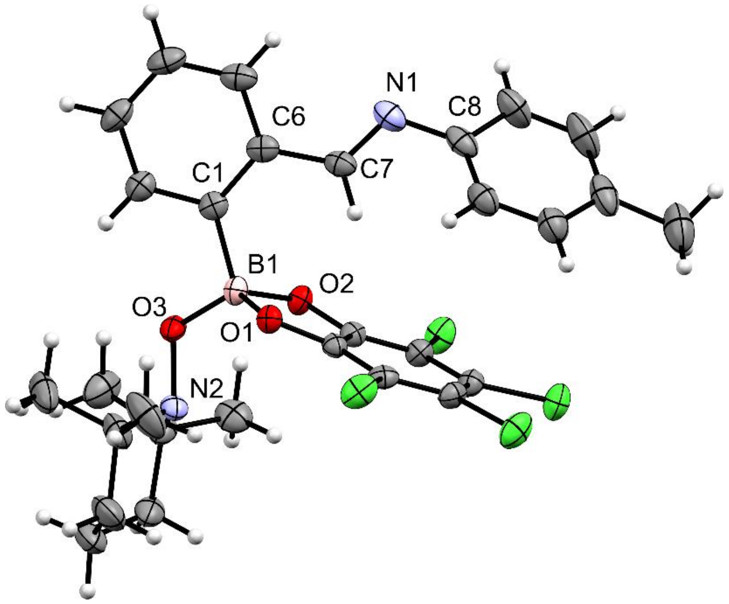

Figure S106. ORTEP diagram of the anionic portion of the structure of **3**. Thermal ellipsoids are drawn at the 50% probability level. Selected bond lengths (Å) and angles (º): B(1)-O(3) 1.430(3), B(1)-O(1) 1.529(3), B(1)-O(2) 1.548(3), B(1)-C(1) 1.630(4), N(1)-C(7) 1.272(3), N(2)-O(3) 1.465(3), C(6)-C(7) 1.468(4), O(1)-B(1)-O(2) 100.75(19), O(3)-B(1)-C(1) 106.6(2), O(1)-B(1)-C(1) 111.8(2), O(2)-B(1)-C(1)110.8(2), C(7)-N(1)-C(8) 116.3(3).

13 References

1. Nowell, H.; Barnett, S. A.; Christensen, K. E.; Teat, S. J.; Allan, D. R., *J. Synchrotron Radiat.* **2012,** *19*, 435-441.
2. Bruker-Nonius *APEX, SAINT and XPREP*, Bruker AXS Inc.: Madison, Wisconsin, USA, 2013.
3. Farrugia, L., *J. Appl. Crystallogr.* **2012,** *45*, 849-854.
4. Sheldrick, G., *Acta Crystallograph., Sect. A: Found. Adv.* **2015,** *71*, 3-8.
5. Palatinus, L.; Chapuis, G., *J. Appl. Crystallogr.* **2007,** *40*, 786-790.
6. Sheldrick, G., *Acta Crystallograph., Sect. C: Struct. Chem.* **2015,** *71*, 3-8.
7. Flack, H. D.; Bernardinelli, G., *Acta Crystallograph., Sect. A: Found. Adv.* **1999,** *55*, 908-915.
